# Longer Duration of Active Oil Biosynthesis during Seed Development Is Crucial for High Oil Yield—Lessons from Genome-Wide In Silico Mining and RNA-Seq Validation in Sesame

**DOI:** 10.3390/plants11212980

**Published:** 2022-11-04

**Authors:** Bhagwat Nawade, Ajay Kumar, Rasna Maurya, Rajkumar Subramani, Rashmi Yadav, Kuldeep Singh, Parimalan Rangan

**Affiliations:** 1 Division of Genomic Resources, ICAR-National Bureau of Plant Genetic Resources, PUSA Campus, New Delhi 110012, India; 2 Division of Germplasm Evaluation, ICAR-National Bureau of Plant Genetic Resources, PUSA Campus, New Delhi 110012, India; 3 Queensland Alliance for Agriculture and Food Innovation, University of Queensland, Brisbane, QLD 4072, Australia

**Keywords:** longer duration, early onset, sesame, oil biosynthesis, Kennedy pathway, *ACCase*, *KAS*, *SAD*, *FAD*, *LACS*, *DGAT*, *LPAAT*

## Abstract

Sesame, one of the ancient oil crops, is an important oilseed due to its nutritionally rich seeds with high protein content. Genomic scale information for sesame has become available in the public databases in recent years. The genes and their families involved in oil biosynthesis in sesame are less studied than in other oilseed crops. Therefore, we retrieved a total of 69 genes and their translated amino acid sequences, associated with gene families linked to the oil biosynthetic pathway. Genome-wide in silico mining helped identify key regulatory genes for oil biosynthesis, though the findings require functional validation. Comparing sequences of the *Si*SAD (stearoyl-acyl carrier protein (ACP)-desaturase) coding genes with known SADs helped identify two *Si*SAD family members that may be palmitoyl-ACP-specific. Based on homology with lysophosphatidic acid acyltransferase (LPAAT) sequences, an uncharacterized gene has been identified as *SiLPAAT1*. Identified key regulatory genes associated with high oil content were also validated using publicly available transcriptome datasets of genotypes contrasting for oil content at different developmental stages. Our study provides evidence that a longer duration of active oil biosynthesis is crucial for high oil accumulation during seed development. This underscores the importance of early onset of oil biosynthesis in developing seeds. Up-regulating, identified key regulatory genes of oil biosynthesis during early onset of seed development, should help increase oil yields.

## 1. Background

**Figure 1:**
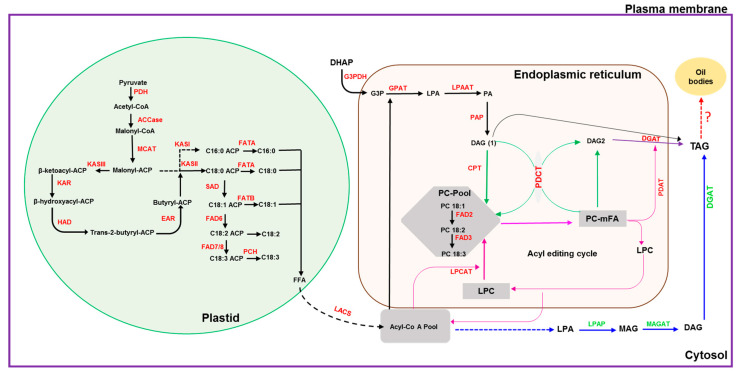
Schematic diagram depicting the oil biosynthesis pathway in plants (adapted from: [5,6,7,8,9,10]). The black dotted arrow indicates multiple steps in the conversion of FFA to acyl-CoA and transport; the blue dotted arrow indicates steps involved in the conversion of acyl-CoA to LPA; the red dotted arrow indicates conversion of TAG to oil bodies or lipid droplets. **ACCase:** acetyl-CoA carboxylase; **ACP:** acyl-carrier protein; **CPT:** CDP-choline:DAG cholinephosphotransferase; **DAG:** diacylglycerol; **DGAT:** diacylglycerol acyltransferase; **EAR:** enoyl-ACP reductase; **FAD2:** oleate desaturase; **FAD3:** linoleate desaturase; **FAD6:** oleate desaturase; **FAD7/8:** linoleate desaturase; **FATA:** fatty acid thioesterase A; **FATB:** fatty acid thioesterase B; **FFA:** free fatty acid; **G3PDH:** glycerol-3-phosphate dehydrogenase; **G3P:** glycerol-3-phosphate; **GPAT:** glycerol-3-phosphate acyltransferase; **HAD:** 3-hydroxyacyl-ACP dehydratase; **KASI:** 3-ketoacyl-ACP synthase I; **KASII:** 3-ketoacyl-ACP synthase II; **KASIII:** 3-ketoacyl-ACP synthase III; **KAR:** 3-ketoacyl-ACP reductase; **LACS:** long-chain acyl-CoA synthetase; **LPAAT:** lysophosphaditic acid acyltransferase; **LPCAT:** acyl-CoA:lysophosphatidylcholine acyltransferase; **LPAP:** lyso-phosphatidic acid phosphatase; **LPA:** lyso-phosphatidic acid; **LPC:** lyso-phosphatidylcholine; **MAG:** monoacylglycerol; **MAGAT:** monoacylglycerol acyltransferase; **MCAT:** malonyl Coenzyme A-ACP transacylase; **mFA:** PC-modified FA; **PAP:** phosphatic acid phosphohydrolase; **PA:** phosphatidic acid; **PC:** phosphatidylcholine; **PDH:** pyruvate dehydrogenase; **PCH:** palmitoyl-CoA hydrolase; **PDAT:** phospholipid:diacylglycerol acyltransferase; **PDCT:** PC:DAG cholinephosphotransferase; **SAD:** stearoyl-ACP desaturase; **TAG:** triacylglycerol.

Plants accumulate oil primarily in the form of triacylglycerols (TAG) [1]. Triacylglycerols have nutraceutical value and are the main source of edible oils for households and industrial applications. With the increasing human population, the global consumption for vegetable oils has increased by >50% over the past decade and is expected to double by 2040 [2,3]. The enzymatic reactions for TAG biosynthesis in plants are well established with the pathways for fatty acid (FA) biosynthesis in the plastid followed by TAG formation in the endoplasmic reticulum (ER) (Figure 1). For the final step in TAG biosynthesis, in addition to the reaction catalyzed by diacylglycerol O-acyl transferase (DGAT) (Figure 1), an acyl-CoA-independent reaction is catalyzed by phospholipid:diacylglycerol acetyltransferase (PDAT) ([4,5] and citations therein). Further, a cytosolic pathway has been identified in groundnut [6]. 

The first committed enzyme is acetyl-CoA carboxylase (ACCase), which acts as a control point over the carbon flux into FAs [8]. In plants, it is present in two functional forms, the heteromeric form in plastids and the homomeric form in the cytosol [11]. Heteromeric ACCase participates in FA synthesis, while the homomeric ACCase is involved in the synthesis of long-chain FAs and secondary metabolites [11]. There are four subunits present in heteromeric ACCase, encoded by *accC* for biotin carboxylase (BC), *accB* for biotin carboxyl carrier protein (BCCP), *accA* for α-carboxyl transferase, and *accD* for β-carboxyl transferase [12]. Of these four, only *accD* is chloroplast-encoded, while the others are nuclear encoded [8]. The malonyl-CoA-acyl carrier protein transacylase (MCAT) coding gene, *FabD*, is one of the key regulators in FA biosynthesis [13]. It aids in the formation of malonyl-ACP, the building block of FA synthesis, from malonyl-CoA [14]. Subsequently, *β*-ketoacyl-ACP synthase III (KASIII; encoding gene: *FabH*) catalyzes the condensation and transacylation of acetyl-CoA with malonyl-ACP to form 3-ketobutyryl-ACP. *β*-ketoacyl-ACP synthase I (KASI; *FabB*) elongates 3-ketobutyryl-ACP to palmitoyl-ACP in six condensation cycles. In the final elongation step, β-ketoacyl-ACP synthase II (KASII, *FabF*) converts palmitoyl-ACP to stearoyl-ACP in the plastid [15]. The acyl–ACP thioesterases (acyl-ACP TE; *FatA*, *FatB*) catalyze the FA chain termination through hydrolysis of the thioester bond of acyl–ACP to release the free FAs [9]. 

These free FAs are reactivated by a long-chain acyl-CoA synthetase (LACS) to form acyl-CoA esters and are exported to the ER [16]. FA desaturases (FAD) desaturate fatty acyl chains to form unsaturated fatty acids [17]. Plant FADs are of two types, soluble and membrane-bound desaturases. Stromal Δ9 acyl-ACP desaturases (AADs) introduce a double bond in saturated acyl chains to form cis-monoenes. The stearoyl-ACP-desaturase (SAD) is the archetype for this family and desaturates stearoyl-ACP (18:0) at C-9 to form oleic acid (18:1; omega-9). An AAD isoform, palmitoyl-ACP 19 desaturase (PAD), exhibits substrate specificity to palmitoyl-ACP, thereby producing palmitoleic acid (16:1; omega-7) [18]. The substrate-specific activity of SAD enzymes determines the ratio of omega-7 and omega-9 FAs in plants [19]. Membrane-bound desaturases are localized to the ER and plastid membranes, and they are grouped into several FAD subfamilies including Omega-3-FAD (fatty acid desaturase3 FAD3, FAD7, and FAD8) and Omega-6-FAD (FAD2 and FAD6) [20]. In ER, the sequential transfer of fatty acyl moieties to a glycerol-3-phosphate backbone generates TAG in four steps, also known as the Kennedy pathway [21]. Glycerol-3-phosphate is acylated by glycerol-3-phosphate acyltransferase (GPAT) to form lyso-phosphatidic acid (LPA) and further acylation of LPA by lysophosphatidic acid acyltransferase (LPAAT) to produce phosphatidic acid (PA) [1]. Subsequently, PA is hydrolyzed by phosphatidic acid phosphatase (PAP) to generate diacylglycerol (DAG). Plant PAPs are classified into two types: soluble-type PAPs called phosphatidate phosphohydrolases (PAH) dephosphorylate PA and membrane-bound PAPs called lipid phosphate phosphatases (LPP) dephosphorylate different lipid phosphates, non-specifically. In the final step, diacylglycerol acyltransferases (DGAT) convert DAG to TAG through the transfer of an acyl group [7] (Figure 1). 

Sesame (*Sesamum indicum* L., 2n = 2x = 26) is an ancient oilseed crop of the Pedaliaceae family. Sesame seeds are nutritionally rich and possess 50–60% oil, 25% protein, and antioxidants such as sesamolin and sesamin [22]. Sesame is a resilient crop with strong adaptation to environmental changes and is considered the ‘queen of oil seeds’ [23]. Sesame seed yield during 2019 was 6.5 million tons, as per FAO statistics (https://www.fao.org/faostat/en/#data; accessed on 28 September 2022). Understanding the genetic basis of oil biosynthesis and developing stable high oil yielding sesame varieties have been key objectives in sesame breeding in recent years [22]. To accelerate crop improvement, omics and molecular tools play key roles through the availability of high-throughput sequencing technologies. Sesame has gained increasing attention, and new genomic information is becoming available for the identification of genes and markers underlying the traits of interest, especially oil yield and productivity, with the help of omics data [24,25]. Here, we mined the existing genomic information for genes (and their translated amino acid sequences) and their families involved oil biosynthesis and subjected them to different bioinformatic tools to understand the characteristic features of the nucleotide and translated protein sequences. Our study identified the orthologs in sesame, on a homology basis, of genes that were previously unknown and also of genes associated with high oil yield with the potential to enhance oil yield in sesame.

## 2. Results

### 2.1. Physicochemical Properties of Retrieved Proteins

The translated amino acid sequences of genes involved in the oil biosynthesis pathway and their paralogs in sesame were mined from the NCBI database (Table S1). A total of seven genes that encode four subunits of heteromeric ACCase enzymes were found, including two for *SiaccB*, one for *SiaccC*, three for *SiaccA*, and one for chloroplastic *SiaccD* (Table S1). One *MCAT*, eight *KAS*, and thirteen *FAD* genes, including seven *SAD*, two *FAD2*, and one each of *FAD3*, *FAD6*, *FAD7*, and *FAD8*, were retrieved. Six *LACS*s, seventeen *GPATs*, six *LPAATs*, three *PAHs*, and three *DGATs* were retrieved in sesame (Table S1). Translated amino acid sequences (Seq_File_S1.docx) were used for the analysis. The analyzed protein sequences of sesame ranged between 271 amino acids for *Si*accB-2 (29.06 kDa) and 1004 (110.47 kDa) for SiPAH2 (Table S2). The predicted aliphatic index of the studied sesame proteins ranged from 72.20 to 121.27. The aliphatic index indicates the thermostability and half-life of a protein [26]. 

### 2.2. Subcellular Localization

The subcellular localization for the translated AA sequences was predicted in silico using the DeepLoc tool. For the nuclear-encoded subunits of ACCase, *Si*accB1, *Si*accB2, and *Si*accC were localized to the plastid while *Si*accA localized in the plastid membrane (Table S2). All the members of *Si*KASI and *Si*KASII were plastid localized (Table S2). 

The TMHMM tool predicted the existence of transmembrane helix domains in the FAD, GPAT, LPAAT, and DGAT families. In the case of membrane-bound FADs (*Si*FAD2-1, *Si*FAD2-2, *Si*FAD3, *Si*FAD6, *Si*FAD7, and *Si*FAD8), the number of transmembrane domains among proteins exhibited variability between a total of five transmembrane domains in *Si*FAD2-1 and only one in *Si*FAD7 (Figure S1). *Si*GPAT proteins were predicted to have two to four transmembrane domains (Figure S2). The TMHMM tool had predicted nine and two transmembrane domains for *Si*DGAT1-1 and *Si*DGAT-2, respectively (Figures S1–S3). 

### 2.3. Homology Relationship

**Figure 2:**
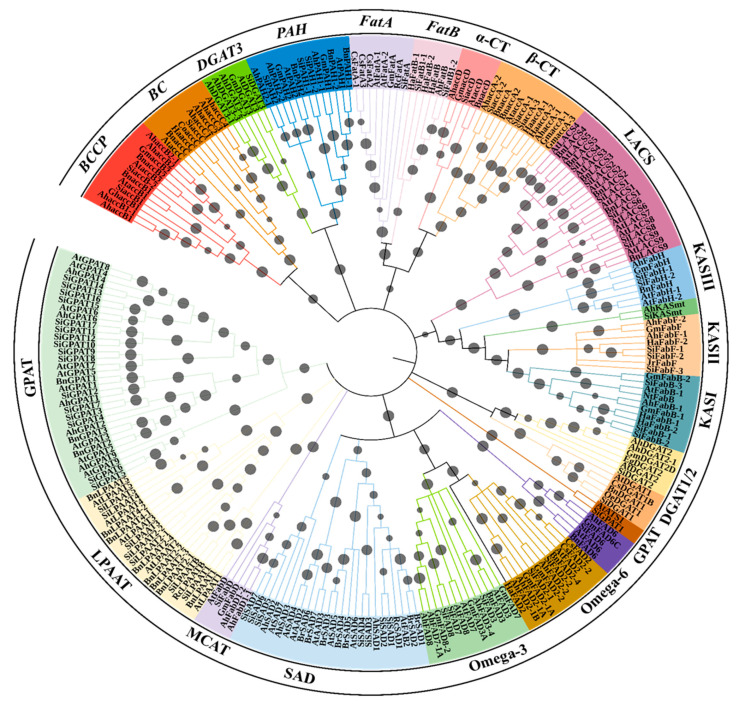
Circular dendrogram for the gene products involved in oil biosynthesis constructed using the N–J method with 10,000 bootstraps using MEGAv10 [27]. For accession number details of each gene product, please refer to Supplementary Table S1. Bootstrap values are represented by grey circles on the branches. The subfamily names of gene products are denoted on the outermost circle of the tree. Ah—*Arachis hypogaea*, At—*Arabidopsis thaliana*, Bn*—Brassica napus*, Br—*Brassica rapa*, Co—*Camellia oleifera*, Cs—*Camelina sativa*, Eg—*Elaeis guineensis*, Gh*—Gossypium hirsutum*, Gm—*Glycine max*, Ha—*Helianthus annuus*, Hi—*Handroanthus impetiginosus*, Ji—*Jatropha curcas*, Jr—*Juglans regia*, Muc—*Macfadyena unguis-cati*, Nt—*Nicotiana tabacum*, Oe—*Olea europaea*, Pf—*Perilla frutescens*, Rc—*Ricinus communis*, Sa—*Striga asiatica*, Sh—*Salvia hispanica*, Si—*Sesamum indicum*, Tm—*Tropaeolum majus*.

A tree was constructed based on an amino acid sequence alignment to understand the homology relationships between gene families of oil biosynthesis of sesame and other related oilseed crops (Seq_File_S1.docx). The gene products involved in oil biosynthesis were grouped into 11 distinct clades (Figure 2). All desaturase proteins formed a single clade with four subclades, in which one subclade consist of two groups, FAD2 in one and FAD3 and FAD7/8 in the second group. The second subclade possesses most of the SAD proteins excluding six SADs (*Si*SAD5, *Si*SAD6, *Si*SAD7, *At*SAD7, *Ah*SAD2, and *Ah*SAD3, that are grouped in third subclade). Lastly, the fourth subclade grouped all FAD6 proteins (Figure 2). KAS and LACS proteins formed a separate clade (Figure 2). All GPAT proteins were grouped into a single clade, except *Si*GPAT1 and *At*ATS1. In a clade containing FatA and FatB proteins, α-CT and β-CT subunits of ACCase were grouped in two distinct subclades (Figure 2). The other subunit proteins of ACCase proteins, BCCP and BC, were grouped in a different clade. Among the Kennedy pathway gene products, DGAT3 and PAH were more closely related and fall into one clade. Similarly, DGAT1 and DGAT2 forms a distinct clade (Figure 2). The protein identified by us as *Si*LPAAT-B on the basis of homology relationship with class-B LPAAT of *Ricinus communis* 410 (*Rc*LPAAT-B, Figure 2) is annotated in the NCBI protein database as 1-acyl-sn-glycerol-3-phosphate acyltransferase, (XP_020554192.1). 

### 2.4. Multiple Sequence Alignment 

Multiple sequence alignments of BCCP proteins indicated that the C-terminal region was conserved with a typical biotinyl domain (CIIEAMKLMNEIE) (Figure S4A), while sequence alignments of BC (accC) proteins indicated their highly conserved nature (Figure S4B). The N-terminal domain of α-CT proteins showed more conserved sites than the C-terminal one, whereas in the β-CT proteins, the C-terminal domain was more conserved (Figure S5). 

The specific amino acid residues that are conserved in the active sites of FabD proteins are present in *Si*FabD as Gln112, Ser197, Arg222, His310, and Gln359 were highly conserved in *Ah*FabD as Gln89, Ser174, Arg199, His287, and Gln336 [13,28] (Figure S6). An active site triad Cys-His-Asn and the motif GNTSAAS were found to be conserved in *Si*KASIII proteins (Figure S7). The seven catalytic sites’ residues (Cys, His, Thr, Thr, Lys, His, and Phe) and cation site residues [29] are highly conserved in the KASI and KASII proteins (Figure S8). The presence of two histidine boxes in the *Si*SAD genes is consistent with plastidial stearoyl-ACP desaturases containing EENRHG and DEKRHE boxes (Figure S9; Table 1). 

To identify specific *Si*SAD forms potentially possessing palmitoyl-ACP activity, sesame SAD sequences were aligned with well-characterized SADs, including SADs characterized as 18:0-ACP specific, *At*FAB2 [18], and *Rc*SAD1 [30]. The palmitoyl-ACP (16:0-ACP)-specific enzymes were characterized using sequence information from *At*AAD2 and *At*AAD3 in *Arabidopsis* [31] and *Muc*PAD in *Macfadyena unguis-cati* [32]. These studies reported that the substrate specificity of SAD is determined by eight amino acid residues from the region of the catalytic sites (Figure S9; Table 2). The comparison showed that 5 *Si*SAD members (*Si*SAD1-5) have the same amino acid residues at catalytic sites as *At*FAB2 and *Rc*SAD1 (except that *Si*SAD3 and *Si*SAD5 have isoleucine instead of leucine at the 142nd position), while *Si*SAD6 and *Si*SAD7 showed divergent residues in the aforesaid catalytic sites, suggesting that they might possess 16:0-ACP substrate specificity (Table 2). Sequence alignment of *Si*FatA and *Si*FatB with representative proteins identified four conserved residues necessary for thioesterase activity (Figure S10). All membrane-bound FAD proteins contain three conserved histidine motifs, H (X)3–4H, H (X)2–3HH, and H/Q (X)2–3HH. Eight conserved histidine residues from these boxes were reported to be important for di-iron center formation (Figure S11). 

Two conserved domains are typically present in the LACS family (Figure S12), an AMP-binding domain (involved in adenylate formation) and the ACS signature domain for catalytic activity [33]. Four conserved acyltransferase domains necessary for acyltransferase activity [34] were found in all SiGPAT proteins (Figure S13). Two protein motifs (NHX_4_D and EGTX) essential for LPAAT activity [35] were present in all LPAAT proteins (Figure S14). The catalytic motif DI/VDGT, characteristic of the Mg^2+^-dependent PA phosphatases, was identified in all PAH proteins (Figure S15). The sequences of DGAT subfamilies were distinct with no common features (Figures S16–S18).

### 2.5. Conserved Motif Analysis

Proteins having related functions contain highly conserved short (<20 amino acids) amino acid sequences recurring in fixed-length patterns called motifs. A motif may represent important biological features, such as protein-binding or targeting a particular subcellular location. Conserved motifs in all studied genes (Figures S19–S36) were identified using the MEME web tool.

### 2.6. Promoter Analysis

**Figure 3:**
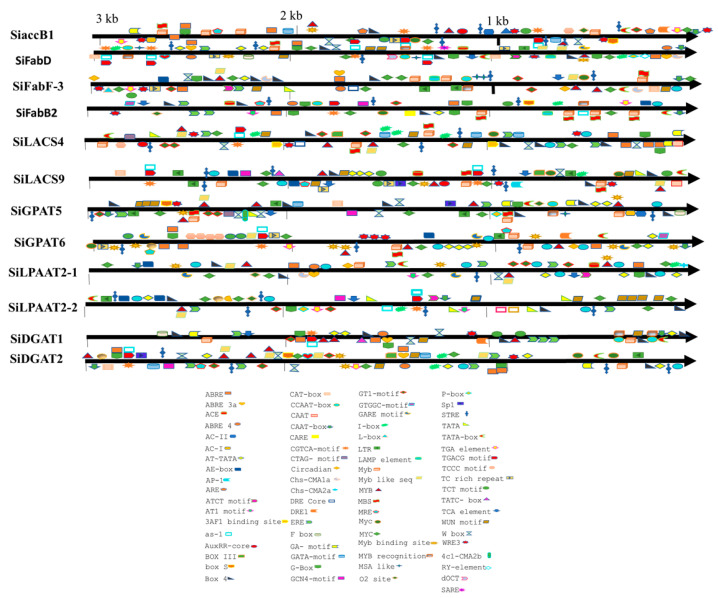
Distribution of *cis*-acting regulatory elements (CAREs) in the promoters of selected genes detected in the PlantCare tool.

**Figure 4:**
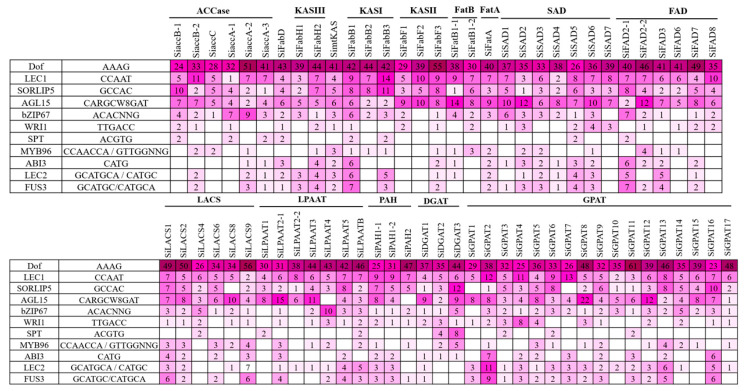
The number of binding sites identified using PLACE and PlantPAN 3.0 tools for each of the CARE (*cis*-acting regulatory elements) associated with oil accumulation (represented in a heat map).

Based on the homology relationships for genes associated with oil biosynthesis in model plants and genotypes exhibiting higher oil yield, we have identified 12 genes in sesame that could be potential targets for enhancement in the sesame oil yield (Table 3). The upstream sequences (3 kb from the start codon) for each of the 12 genes were subjected to promoter analysis using the PlantCARE database to predict the potential regulatory features [36]. A total of 79 CAREs (cis-acting regulatory elements) were detected from the upstream sequences of the aforementioned 12 genes potentially targeted to improve oil yield (Figure 3; Table S3). Different TFs associated with FA biosynthesis regulation and TAG accumulation are well documented, including DNA binding with one finger (Dof), LEAFY COTYLEDON1 (LEC1), LEC2, sequences over-represented in light-induced promoters 5 (SORLIP5), AGAMOUS-LIKE15 (AGL15), BASIC LEUCINE ZIPPER 67 (bZIP67), SPATULA (SPT), WRINKLED1 (WRI1), MYB96, ABSCISIC ACID INSENSITIVE3 (ABI3), and FUSCA3 (FUS3) [37,38]. Of these 11 TFs, binding sites for TFs DOF, SORLIP5, AGL15, and LEC1 were identified in the promoter regions of all the 12 genes studied (Figure 4). Although the number of DOF binding sites is over-represented in all these promoters studied, the literature suggests that not all the AAG motifs in plant promoters are targets for the DOF domain containing proteins and are variably regulated [39]. DOF4 and DOF11 positively regulate ACCase and LACS and, thereby, increase seed oil content in Arabidopsis, while this negatively regulates CRA1, associated with seed storage protein [40]. LEC1 and LEC2 positively regulate WRI1 and AGL15, respectively [41,42]. WRI1 in turn positively regulates TAG biosynthesis through DGAT1 [43]. Our transcriptome analysis also revealed the higher expression levels of WRI1 (SIN_1023649), specifically in high oil yielding genotypes. Binding sites for MYB96, a positive regulator of seed oil accumulation and activator of the DGAT1 gene in Arabidopsis seeds [44], was detected in the upstream sequences of the SiDGAT2 and SiDGAT3 genes in sesame (Figure 4), but not in the SiDGAT1 gene. It is reported that MYB96 binds to the promoter of ABI4 and activates its expression [45], and ABI4 has been shown to directly regulate DGAT1 expression in Arabidopsis [46].

### 2.7. Validation of In Silico Predicted Oil Biosynthesis Genes Using RNA-Seq

The key regulatory genes associated with oil biosynthesis (Table 3) were identified through genome-wide in silico mining approaches. Expression of these genes was validated through RNA-seq based differential expression studies using the publicly available transcriptome data for three genotypes (high oil yielding-ZZM4728, low oil yielding-ZZM3495, ZZM2161) at four developmental stages (10, 20, 25, and 30 days-post-anthesis: DPA) [24]. In total, 501 genes were found to be significantly differentially expressed in the high oil yielding sesame genotype (ZZM4728) when compared to the low oil yielding genotypes (ZZM3495 and ZZM2161) (Table S4). Further, of the 501 genes, 27 significantly differentially expressed genes were associated with the lipid biosynthesis (including lipid droplet biogenesis) pathway. From these, *DGAT*, *LPAAT*, *FAD*, *SAD*, *KCS* (β-ketoacyl-CoA synthase), *Oleosin*, *nsLTP* (non-specific lipid transfer protein), and *DIR1* (defective in induced resistance1) genes were selected manually.

**Figure 5:**
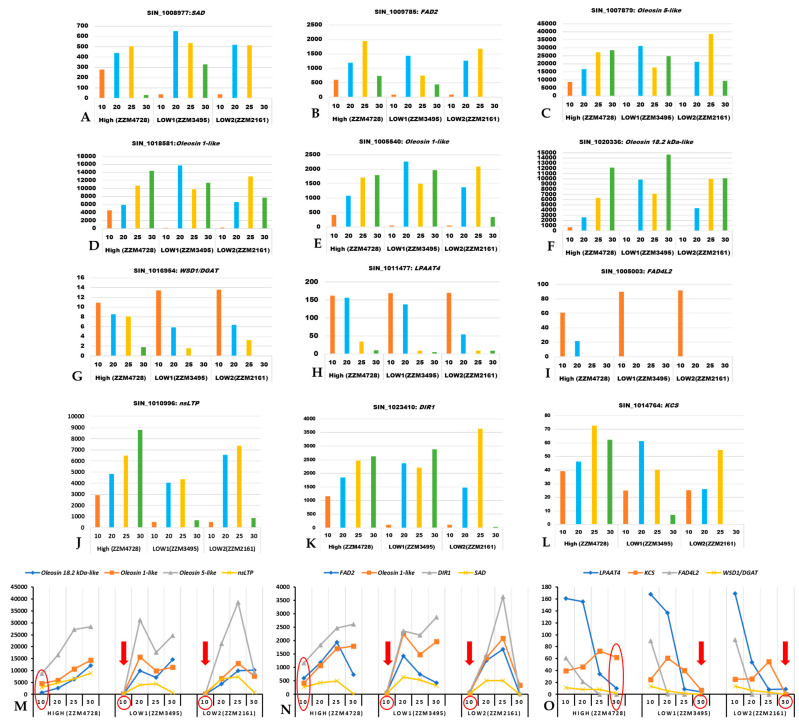
Expression profiles (in FPKM values, Y-axis) for key regulatory genes of oil biosynthesis in sesame using the transcriptome dataset of Wang et al. (2019). The X-axis represents the developmental stages in days post anthesis (DPA). (**A**): *Sad*; (**B**): *Fad2*; (**C**): *Oleosin 5-like*; (**D**): *Oleosin 1-like*; (**E**): *Oleosin 1-like*; (**F**): *Oleosin 18.2 kDa-like*; (**G**): *Dgat*; (**H**): *Lpaat4*; (**I**): *Fad4L2*; (**J**): *nsLTP*; (**K**): *DIR1*; (**L**): *KCS*; (**M**,**N**): Set of genes that are expressed significantly less at 10 DPA in both of the low oil yielding genotypes (indicated with a red colored down arrow at 10 DPA); (**O**): Set of genes that are significantly less expressed at 30 DPA in both of the low oil yielding genotypes (indicated with a red colored down arrow at 30 DPA).

The *SAD* gene showed higher expression at the early stage (10 DPA) in the high oil yielding genotype while its higher expression was found during middle stages (20 and 25 DPA) in the low oil yielding genotypes (Figure 5). Similar to *SAD* expression, *FAD2* gene expression also increases consistently from the early seed developmental stage (10 DPA) and is decreased at 30 DPA in the high oil yielding genotype, while, in case of low oil yielding genotypes, its expression level increased only during the middle developmental stage (20 DPA) (Figure 5). We also found genes associated with lipid droplet biogenesis (oleosin gene family) to be significantly more highly expressed in the high oil yielding genotypes, at 10 DPA. Interestingly, these oleosin genes exhibited higher expression levels in both high and low oil yielding genotypes during later developmental stages (25 and 30 DPA). The genes *WSD1*/*DGAT*, *LPAAT*, and *FAD4L2*exhibited higher expression levels during the early and middle developmental stages (10 and 20 DPA) in both high and low oil yielding genotypes. However, the expression level of these genes decreased towards later developmental stages (25 and 30 DPA) in low oil yielding genotypes while the expression pattern in high oil yielding genotypes is sustained at comparatively higher levels than the low oil yielding genotypes. Genes for lipid transfer proteins include *ns*LTP-encoding genes and *DIR1* [61,62]. The gene product of *KCS* catalyzes the first step of the very long chain FA biosynthesis [63,64]. These three genes, *nsLTP*, *DIR1*, and *KCS*, tended to show higher expression in the high oil content sesames genotype when compared to the low oil yielding genotypes (Figure 5J–L). Of these, expression pattern for *nsLTP1* and *DIR1* transcripts were lower at 10 DPA in low oil yielding genotypes when compared to high oil yielding genotypes, whereas *KCS* expression was found to be lower at 30 DPA in the low oil yielding genotypes than the high oil yielding genotype (Figure 5M–O). These results suggest that early onset of oil biosynthesis during seed development is important for higher oil yield, and this is regulated transcriptionally by the key regulatory genes studied here. In low oil yielding genotypes, the capacity for oil biosynthesis evidently initiates during the mid to late seed developmental stage, which may explain the low oil yield. Since the time duration between anthesis and capsule maturity is uniformly 30–45 days [24], early onset of oil biosynthesis during seed development results in the longer duration of active oil formation and, thereby, higher oil yield.

## 3. Discussion

With the availability of high-throughput technologies and the sesame genome datasets, various genes involved in oil biosynthesis have been mined from the publicly available sesame genome and RNA-seq datasets and were studied using various bioinformatics tools. The present report focused on studies pertaining to conserved sites, catalytic sites, domains and motifs, physicochemical properties, subcellular location, and their homology with genes of other oilseed crop and model plants. TAG biosynthesis in oilseeds can be summarized in three major steps (Figure 1): (i) biosynthesis of FA in plastids, (ii) desaturation of FA in plastid and ER, and (iii) TAG assembly in ER, where the free fatty acids are exported from the plastid to undergo stepwise acylation onto the glycerol backbone, forming TAG.

Plant de novo FA synthesis occurs in the stroma of the plastid in a series of reactions: initiation, elongation, and termination [5]. The heteromeric ACCase subunit genes have been characterized in different crop plants, including peanut [13], soybean [12], *Gossypium* species [48], and pea [65]. Overexpression of the *BCCP* gene modulates the oil content in seeds of *Arabidopsis* and cotton [47]. Additionally, overexpression of the *accD* gene through chloroplast transformation resulted in increased ACCase levels that led to higher oil production in transgenic tobacco [49].

### 3.1. Characterization of the In Silico Mined Genes of Oil Biosynthesis

An evaluation of the physicochemical properties of the amino acids of FA synthesis genes highlighted that, on an isoelectric point (pI) basis, most of the proteins were alkaline in nature except *Si*accB-2, *Si*accC, *Si*accD*, Si*KASIII, *Si*SAD, and FATB (Table S2). Similarly, the isoelectric points (pIs) of FAD genes from sunflower (pIs ranged from 6.24 to 9.61) and *Brassica napus* (7.8 to 9.5) showed alkaline nature [66,67]. The instability index is a measure of correlation with the in vivo half-life of a protein [68]. The aliphatic index is defined as a relative volume occupied by aliphatic side chains (alanine, valine, leucine, and isoleucine). Aliphatic amino acids are hydrophobic; therefore, a high aliphatic index indicates that a protein is thermo-stable over a wide temperature range and is regarded as a positive factor for the increase in thermostability of globular proteins [69]. The GRAVY value indicates the solubility of a protein, and a low GRAVY value indicates the hydrophilicity of the protein [69]. Most studied proteins have negative values revealing their hydrophilic nature (Table S2).

The catalytic residues are well conserved within KASI, KASII, and KASIII proteins (Figures S7 and S8). These residues are reported to each have a key function: cysteine (C) is a substrate-binding residue, two histidines (H) are involved in the decarboxylation, and two threonines (T) are involved in forming hydrogen bonds with the ACP phosphopantetheine moiety [15] (Figure S8). In the C-terminal region, a conserved Gly-rich GNTSAAS motif was also found, that has been reported to be involved in forming oxide anion free radical [15]. Hence, formed oxide anion free radicals participate in redox reactions leading to oxidative modifications. The plant KASIII enzyme-specific catalytic triad composed of Cys-His-Asn was conserved in all *Si*KASIII proteins [70] (Figure S7). Although the N-terminal domains of FatA and FatB showed high divergence, the specific catalytic residues were conserved (D268, N270, H272, and E306 in *Si*FatA; D282, N284, H286, and E320 in *Si*FatB-1, and D319, N321, H323, and E357 in *Si*FATB1-2) (Figure S10).

The FAD gene family is well characterized in oilseed crops, including 31 in peanut [71], 68 in rapeseed [66], 29 in soybean [72], 40 in sunflower [67], 12 in *B. juncea*, and 8 in black mustard [73]. SAD enzymes catalyze the first desaturation in the plant FA biosynthesis pathway. The members of the SAD family have been identified in oilseed crops, including peanut*, B. juncea*, *B. rapa*, *B. nigra*, olive, and camelina, and possess 3, 12, 7, 8, 3, and 3 *SAD* genes, respectively [74]. Among seven *At*SAD members, SSI2/FAB was characterized as a typical 18:0-ACP-specific acyl-ACP Δ^9^ desaturase [18], while two members from *At*SAD, *At*AAD2, and *At*AAD3 catalyze 16:0-ACPΔ^9^ desaturation [19]. The key amino acid residues located in the catalytic domain are predicted to shape a deep substrate-binding pocket for 18:0-ACP, while in 16:0-ACP specific enzymes, a shorter channel substrate-binding pocket was observed [75]. Based on the alignment of eight AA residues at the substrate-binding pocket, five *Si*SADs (*Si*SAD1-5) might be of 18:0-ACP specificity and two *Si*SADs (*Si*SAD6 and 7) possibly exhibit 16:0-ACP specificity (Table 2; Figure S9). Of the two *Si*SADs, *Si*SAD6 and 7, structural and functional association analysis of the amino acid residues (for amino acid positions refer to Table 2) reveals the presence of a bulky amino acid in *Si*SAD6 (W198), which is similar to *At*AAD2 (F224), *At*AAD3 (F216), and *Mu*PAD (W151), but not in *Si*SAD7. This observation favors *Si*SAD6 as a better candidate for PAD than *Si*SAD7. Since the orthologous genes from different species tend to group together rather than with the paralogous genes within a species, homology relationships (Figure 2) indicate *Si*SAD6 and *At*AAD2 and 3 would have evolved their catalytic specificity independently. Hence, the *Si*SAD6 is not as similar (with respect to orthologous relationship) as *Br*SAD6 with *At*AAD2 or *Br*SAD7 with *At*AAD3 (Figure 2). As reported in cotton (*GhA-SAD6* and *GhD-SAD8*) and Arabidopsis (*At*AAD2 and 3) for the preferential expression (of the respective gene copies) in the endosperm and aleurone tissues [19,31,75,76,77], the gene copies do exhibit tissue-specific selective expression patterns in addition to their specificity for 16:0-ACP or 18:0-ACP.

Membrane-bound FADs include omega-6-FADs (FAD2 and FAD6), omega-3-FADs (FAD3, FAD7 and FAD8), and palmitate desaturase (FAD4) [67]. FAD2 and FAD6 synthesize linoleic acid from oleic acid in plastids and ER, respectively [9]. In this study, three omega-6-FADs were retrieved from the sesame genome (Table S2). The presence of multiple copies of FAD2 is common in oilseed crops. For example, six FAD2 have been reported in peanut, five in soybean, six in safflower, three in sunflower, four in cotton, and two in flax [20]. Previously, FAD2 from sesame was isolated and characterized [78]. Furthermore, screening of the variants for *SiFAD2-1* from 705 accessions detected a mutation causing an amino acid change (R142H), which probably affects the desaturase activity, with mutant accessions accumulating extremely high levels of oleic acid (48%) in seed oil [25]. In the present study, a FAD2 member, designated as *SiFAD2-2*, was retrieved from the sesame genome (Table 1). The omega-3 desaturase genes (FAD3, FAD7, and FAD8) were found as single copies in sesame (Table S1).

The physicochemical properties of the FA desaturase proteins revealed that both *Si*SAD and *Si*FAD families were hydrophilic in nature. *Si*FAD proteins were predicted as alkaline proteins while *Si*SADs were slightly acidic (Table S2). The motifs identified using MEME software for the *Si*FAD genes were highly conserved within each subfamily (Figure S28), in accordance with a previous report [71]. Generally, soluble and membrane-bound desaturases were found to possess two and three histidine boxes, respectively (Table 1). The histidine boxes are essential for FAD catalytic activity [79]. The presence of conserved transmembrane domains is a typical characteristic of membrane-bound FADs [20]. The altered expression pattern of FAD family members is known to alter fatty acid profiles in various oil crops such as *Brassica* [80], cotton [81], and soybean [82]. The mechanism through which the FAD family members’ regulation alters the protein and oil content and yield is not known yet.

### 3.2. Identification of Key Regulatory Genes

The *MCAT* gene has been isolated and characterized in rapeseed, soybean, and peanut [13,83]. MCAT is a key enzyme that catalyzes the first committed step in the FA pathway, and its expression level is closely associated with increased storage oil content [50]. The total seed yield and oil content were increased in transgenic *Arabidopsis* plants overexpressing *MCAT* [50].

β-ketoacyl-acyl carrier synthases (KAS) catalyze chain-initiation, -elongation, and -condensation steps and are classified as KASIII, KASI, and KASII [14]. A total of eight *KAS* gene family members, including two *KASIII*, three *KASI*, and three *KASII*, were found in sesame (Table S2). In silico genome-wide analysis of the *KAS* gene family in flax (*Linum usitatissimum* L.) led to the identification of twelve genes consisting of four *KASIII*, six *KASI*, and two *KASII* [84]. *KAS* genes are reported to affect oil production. KASI-deficient mutant *Arabidopsis* seeds have been reported to produce significantly lower oil content [85]. Overexpression of *NtKASI-1* significantly enhanced oil accumulation in tobacco [15]. Virus-induced gene silencing in *Jatropha curcas* demonstrated that silencing of *JcKASII* significantly altered FA composition and TAG biosynthesis [51]. Statistically significant associations for the two major genes, *KASI* (*SiFabB-2*) and *SiDGAT2*, were reported to determine the unsaturated to saturated fat ratio [25]. No variability for *SiFabB*-2 at sequence level was found among the 705 sesame accessions studied [25].

Acyl-ACP thioesterases play an essential role and exhibit two distinct classes (FatA and FatB) [86]. FatA has higher specificity for unsaturated acyl groups, while FatB is more active in saturated acyl-ACPs. FatA and FatB grouped with α-CT and β-CT subunits of ACCase (Figure 2), which might be due to the presence of a conserved motif in these proteins (Figure S36). Genetic engineering of acyl-ACP thioesterases has been demonstrated to be effective for oil improvement [87]. Overexpression of *FatB1* from *Umbellularia californica* (California Bay laurel) and *MlFatB* from *Madhuca longifolia* in *Brassica juncea* increased the laurate levels by over 50% and stearate levels by 16-fold, respectively [87,88]. A genome-wide association study of 705 sesame accessions reported that the candidate genes underlying the variation in FA composition and oil content include *Si*FatB, *Si*FatA, KASII, KASI, and SAD [25]. Based on homology relationships, we identified them as *Si*FatB1-1, *Si*FatA, *Si*FabF-1, *Si*FabB-2, and *Si*SAD-1, respectively. Availability of the sequence information is much helpful in identifying, on a homology basis, the actual gene copy (among the gene copies in a genome) being transcriptionally regulated with tissue (space) and developmental stage (time) specificity. Hence, the evolutionary or comparative genomics tools are helpful in establishing a homology relationship and, thereby, predict the actual gene copy being specifically expressed with reference to space and time in an organism. Unraveling such biological information on a homology-basis leads to novel insights due to the exceptional conservation of synteny among ortholog blocks [89,90,91,92,93,94]. Adding such homology-based information through bioinformatic tools enriches our understanding on the expression patterns for the biological pathways, with reference to space and time, in the organism of study.

TAGs are the major form of energy storage in plants and contribute to many specific developmental stages of the plant [1]. Long-chain acyl-coenzyme A synthetase (LACS) catalyzes the formation of acyl-CoAs from free fatty acids, which is pivotal for TAG biosynthesis [33]. LACS enzymes esterify free fatty acids to fatty acyl-CoA thioesters [95]. These fatty acyl-CoA thioesters are utilized in many metabolic pathways for FA elongation, triacylglycerols, membrane lipids, wax, cutin, and suberin as well as in FA catabolism [33]. *Arabidopsis* contains one of the largest known LACS families, having nine *LACS* genes [95]. In sesame, we found six *LACS* genes. In comparison to *Arabidopsis*, three *LACS* types, *LACS3*, *LACS5*, and *LACS7*, were absent in sesame (Table S2). Likewise, substantial variation in 629 LACS homologs from 122 species was reported [96].

The functions of most of the LACS members in *Arabidopsis* are well characterized. *At*LACS9 functions redundantly with either *At*LACS1 or *At*LACS4 in seed TAG biosynthesis [16]. *At*LACS-6 and -7 localize in the peroxisome and are involved in *β*-oxidation. *At*LACS-1, -2, and -4 are involved in surface wax and cutin biosynthesis [16]. The overall functions of *At*LACS3 and *At*LACS5 are unknown yet [96]. In oilseed crops, LACS from sunflower*, Ha*LACS1 and *Ha*LACS2, showed high sequence homology with *At*LACS9 and *At*LACS8 and are essential in oil production [97]. *Bn*LACS2 from rapeseed is predominantly expressed during seed development and is involved in seed oil synthesis [98]. The specific gene homologs of *LACS* and *DGAT* in flax, *LuLACS8A* and *LuDGAT2*, respectively, have been reported to contribute in the enrichment of flaxseed oil with α-linolenic acid [99].

Plant GPATs are classified into three types, based on location, as membrane-bound, chloroplastic, and cytosolic [100]. In *Arabidopsis*, ten GPATs were extensively characterized, revealing their multifaceted functions [101]. Based on acyl-transfer specificity of *At*GPATs, they are categorized into sn-1- (*At*ATS1 and *At*GPAT9), GPATs that catalyze esterification of fatty acyl moiety from acyl-CoA (or -ACP) to the Sn-1 position of the G3P, and sn-2-GPAT (*At*GPAT1-8), which esterifies the Sn-2 position of the G3P [102]. Based on their functions, *At*GPATs are classified into three different groups, cutin (*At*GPAT4, 6, and 8), suberin (*At*GPAT5 and 7), and mitochondria-localized (*At*GPAT1-3) [103]. *At*GPAT1 was reported to be involved in tapetum differentiation and male fertility [104], while the functions of *At*GPAT2 and GPAT3 are yet unknown [100]. Plastidial *At*ATS1 and ER-localized *At*GPAT9 contribute to glycerolipid biosynthesis but not extracellular lipids [52]. GPAT family members in *Brassica* are well studied [105]. Similarly, in *Gossypium* species, 28, 27, 16, and 14 *GPAT* homologs were identified from *G. hirsutum*, *G. barbadense*, *G. arboreum*, and *G. raimondii*, respectively [106]. In sesame, *Si*GPAT10-*Si*GPAT17 represent the cutin group (Figure 2), while the suberin group contains *Si*GPAT8 and *Si*GPAT9. Among three mitochondrially targeted GPATs, *At*GPAT1 is found to have homology with *Si*GPAT2, while *At*GPAT-2 and -3 are grouped along with three *Si*GPATs (*Si*GPAT-3, -4, and -7) (Figure 2). *Si*GPAT1 groups with *At*ATS while *Si*GPAT5 and *Si*GPAT6 group with *At*GPAT9 (Figure 2). Therefore, it could be inferred that sesame has two GPATs (SiGPAT5 and SiGPAT6) that may be involved in seed TAG biosynthesis.

LPAAT incorporates FA at the sn-2 position of PA and is a crucial enzyme in membrane phospholipid and storage lipids biosynthesis [107]. In plants, LPAATs are classified into two classes, plastid LPAATs and ER LPAATs, with the ER LPAATs further classified into Class-A and Class-B [108]. In *Arabidopsis*, five LPAATs have been reported, one plastidial (*At*LPAAT1) and four (*At*LPAAT2–5) microsomal targeted [109]. Class-A LPAATs are ubiquitously present in all parts of most of the plants and show substrate specificity with C18 unsaturated FAs [107], whereas class-B LPAATs are expressed in seeds and are associated with acylation of unusual acyl-CoAs [110]. Among eight sesame LPAATs, one (*Si*LPAAT1) was identified to be plastid localized in this study and exhibits similarity with *Arabidopsis At*LPAAT1 (Figure 2). We have identified the protein annotated in the NCBI protein database as 1-acyl-sn-glycerol-3-phosphate acyltransferase (XP_020554192.1) as *Si*LPAATB because it was found to be homologous with class-B LPAAT of *Ricinus communis* (*Rc*LPAATB) (Figure 2). The microsomal LPAAT2/3 group includes *Si*LPAAT2-1 and *Si*LPAAT2-2, along with *Ah*LPAAT2 [53], *Bn*LPAAT2 of *B. napus* [54], and *At*LPAAT2 of *Arabidopsis* [110] (Figure 2). We retrieved *Si*LPAAT3 from the sesame genome, a probable ortholog of the male gametophyte isoenzyme *At*LPAAT3 characterized in *Arabidopsis* [110] (Figure 2). Overexpression of *LPAAT* genes has proven to increase seed oil content. Specifically, *LPAAT2* from rapeseed and peanut enhanced oil content by 13% and 7.4%, respectively, when transformed in *Arabidopsis* seeds [53,54]. The overexpression of yeast *SLC1* and *SLC1–1* genes (homologs of ER *At*LPAATs) in *Arabidopsis*, soybean, and rapeseed resulted in an 8–48% increase in seed oil content [111].

The dephosphorylation of PA to produce DAG catalyzed by PAPs is a committed step in TAG biosynthesis [112]. Transient expression of N-terminal green fluorescent protein (GFP) fused *At*PAH1 in tobacco leaves showed signals in cytosol and is predicted to become localized to the ER membrane to bind with PA [113], which is consistent with previous reports in yeast Pah1 and *Tetrahymena thermophila Tt*Pah1 [114]. A double mutant of the two *At*PAH genes (*At*PAH1 and 2) is affected in phosphatidylcholine production at the ER [113]. These two proteins have been reported as being involved in phospholipid homeostasis in the ER and in TAG synthesis, especially under N starvation [115].

DGATs are studied for enhancing oil production as they are one of the rate-limiting factors in plant storage lipid accumulation. Three structurally unrelated classes of DGATs were identified in plants: DGAT1, DGAT2, and DGAT3. DGAT1 and DGAT2 are associated predominantly with membranes, while DGAT3 is cytosolic [6,116]. Three members of the DGAT1 and four members of the DGAT2 family were found in soybean [60], and seven DGAT1, eight DGAT2 and three DGAT3 were identified in peanut [117].

The putative C-terminal ER retrieval motif is detectable in *Si*DGAT1 (–YYHDL) and in other plant DGAT1s [118]. These putative ER retrieval motifs possess hydrophobic amino acid residues [119]. The *Si*DGAT2 was found to have five conserved domains (Figure S17) as signature motifs within the DGAT2 subfamily. The Pfam analysis revealed that *Si*DGAT1 is a membrane-bound O-transferase (MBOAT) protein with nine transmembrane domains, consistent with the DGAT1 proteins of *Arabidopsis*, *B. napus*, castor, peanut, and soybean, as reported earlier [116]. In *Si*DGAT2, two transmembrane domains at the N-terminal of the protein were predicted (Figure S1), as in the other characterized plant DGAT2 proteins [120]. Overexpression of *DGAT1* genes from *Arabidopsis* and *Camelina sativa* in *Arabidopsis*, rapeseed, and *Camelina* led to a 5–25% increase in storage oil content [55,56,121]. Similar results were documented when *DGAT1* from *Tropaeolum majus* was transformed into *B. napus* [57] and *DGAT1* from *B. napus* and *Sesamum indicum* was transformed into *Arabidopsis* [58,122]. Overexpression of soybean and oil palm *DGAT2* in *Arabidopsis* also increased TAG biosynthesis [59,60].

### 3.3. Validation of the Identified Regulatory Genes through RNA-Seq Studies

Oil biosynthesis in plants is performed through de novo fatty acid biosynthesis, TAG assembly, and lipid droplet biogenesis ([5] and citations therein). *LPAAT* and *WSD1/DGAT* are known to regulate key steps of TAG or lipid biosynthesis in oil seed plants [123,124]. Higher expression levels of the genes involved in TAG biosynthesis such as LPAAT, GPAT, and DGAT were known to be associated with enhanced oil accumulation in the seeds of the oilseed crops [24,123,125]. SAD and FAD2 desaturate fatty acyl chains of unsaturated fatty acids. SAD desaturates stearoyl-ACP (18:0) to form oleic acid (18:1), and FAD2 catalyzes synthesis of linoleic acid (18:2) from oleic acid (18:1) [24]. The expression pattern of SAD and FAD2 corresponds with oleic acid (18:1) and linoleic acid (18:2) at the early and middle stages of developing seeds of sesame [126]. The oleosins have been linked to lipids’ biosynthesis/metabolism and the size of seed oil bodies [127,128]. The expression patterns observed for oleosins are consistent with their critical roles in oil body biogenesis.

Validation of the identified key regulatory genes for oil biosynthesis was completed using RNA-seq data. The transcriptome dataset used for this study was downloaded from publicly available datasetsfor the high and low oil yielding genotypes at four developmental stages [24]. Our results indicate that there are 27 genes (of 501 genes that are significantly differentially expressed between the high- and low-oil yielding genotypes) associated with oil biosynthesis and lipid droplet biogenesis (Table S4; Figure 5). These include those identified through our genome-wide in silico studies. In general, oil accumulation is said to increase rapidly during seed maturation [129]. Transcriptomic studies performed by Wang et al. (2019) underscore the importance of higher expression levels of oil biosynthetic genes at 30 DPA for increased oil accumulation in high oil yielding genotypes, compared to the low oil yielding genotypes [24]. In addition to this, our analysis using the same dataset suggests that early onset of higher expression levels for oil biosynthetic genes is also crucial for higher oil yield. Combining these two, early onset by 10 DPA and sustained expression until 30 DPA, the high oil yielding genotype accommodates a broader window of time for active oil accumulation when compared to the low oil yielding ones. Hence, for higher oil yield, in addition to expression patterns at 30 DPA, expression for genes of the oil biosynthesis pathway to be triggered during the early seed developmental stage (10 DPA) itself also seems to be a determining factor, as identified through the study. This helps in oil accumulating for a longer duration within the anthesis-capsule maturity window, thereby yielding higher oil content in the seeds at maturity.

## 4. Material and Methods

### 4.1. Sequence Retrieval

Using the available sesame genome sequence information [25,130], the gene family members involved in oil biosynthesis were retrieved. The sequence information for genes (and their translated amino acid sequences, Table S1 and Seq_File_S1) in oil biosynthesis in *Arabidopsis*, peanut, soybean, and other plant species was also retrieved. Transcriptome raw reads (90 bp paired-end sequencing using the Illumina Hiseq 2000 platform) of 12 samples representing different stage of seed development (10, 20, 25, and 30 DPA) for 1 high (ZZM4728) oil- and 2 low (ZZM3495 and ZZM2161) oil-yielding genotypes were downloaded from the GenBank SRA database [24] for validation studies.

### 4.2. Physicochemical Analysis

Retrieved translated amino acid sequences were analyzed using the ExPASy ProtParam tool to estimate the molecular weight (MW), isoelectric point (pI), grand average of hydropathy (GRAVY), aliphatic index, and instability index [131]. The subcellular localization was predicted using DeepLoc [132]. TargetP-2.0 and ChloroP 1.1 were used to predict signal peptides. The transmembrane structures were predicted using TMHMM-2.0 (http://www.cbs.dtu.dk/services/; accessed on 28 September 2022).

### 4.3. Phylogenetic Analysis and Conserved Motifs Screening

The conserved domains for the retrieved proteins were also identified from the data reported in the NCBI conserved domain database [133]. Domain analysis of the retrieved proteins was performed using the Pfam database [134]. Retrieved sequences were also verified using a simple modular architecture research tool (SMART, http://smart.embl.de; accessed on 28 September 2022), an annotation resource [135]. Multiple sequence alignments (MSA) of the retrieved protein sequences were performed using the Clustal [136] with default parameters. Sequence analyses were conducted in MEGAv10 [27] with a bootstrap value set to 10,000. The MEME v4.12.0 [137] server was utilized to identify the conserved amino acid motifs (Table S1) with the following setting: the maximum number of motifs—ten and minimum motif width—six.

### 4.4. Analysis of Cis-Acting Regulatory Elements

The retrieved candidate promoter regions (3 kb upstream) were used to predict the potential *cis*-acting regulatory elements (CAREs) using the PlantCare tool (http://bioinformatics.psb.ugent.be/webtools/plantcare/html/; accessed on 28 September 2022). Function annotation of identified CAREs was retrieved from the PlantCare database (Lescot et al. 2002).

### 4.5. Analysis of CAREs for Oil Related Transcription Factors Binding Sites (TFbs)

A total of 11 TFs were identified to be involved in regulating seed oil accumulation [37,38,40,138]. Potential TF binding sites in all retrieved promoters sequences were identified using PLACE [36] (http://www.dna.affrc.go.jp/PLACE/; accessed on 28 September 2022) and PlantPAN 3.0 [139] (http://plantpan.itps.ncku.edu.tw/index.html; accessed on 28 September 2022).

### 4.6. RNA-Seq Analysis between High and Low Oil Content Yielding Sesame Genotypes

The fastq files of raw reads [24] were processed to check the quality parameters using FastQC [140]. The adapter, low-quality sequences, were trimmed using sickle (v1.33) [141]. The reads were subjected to a quality check using FastQC post-trimming. The filtered high-quality reads were mapped against the reference sesame genome [130] using TopHat (v2.1.1) [142] with default parameters. A reference-guided assembly of the transcriptome data from all 12 samples was performed using Cufflinks (v2.1.1) [143], and a consensus assembly was generated using Cuffmerge. The differentially expressed genes were identified using cuffdiff [144] with default parameters. The transcripts exhibiting differences of at least two-fold with FDR value ≤ 0.05 were considered to be significantly differentially expressed. The transcriptome database (cDNA sequences) for sesame genotype *Zhongzhi-*13 [130] was downloaded and functionally annotated using omicsbox (v1.4.11) [145]. All significantly differentially expressed gene IDs were searched in the annotated file to assign the functionality of the unknown differentially expressed genes.

## 5. Conclusions

We report here on the predicted physicochemical properties, subcellular locations, conserved sites, and homology relationships for the gene products involved in oil biosynthesis in sesame, using the available genome information. Of eight SAD members, which are known as stearoyl-(acyl-carrier-protein) 9-desaturases, two members were predicted to be possibly 16:0-ACP specific (Table 2). Similarly, *Si*LPAAT1, previously uncharacterized, has been functionally characterized by its homology to LPAAT1 from *Arabidopsis* (Figure 2). Moreover, *Si*LPAATB, previously annotated as 1-acyl-sn-glycerol-3-phosphate acyltransferase, is a class-B LPAAT, identified through a homology relationship with *Ricinus communis Rc*LPAATB (Figure 2). The genome-wide in silico mining revealed key regulatory genes associated with the oil biosynthesis pathway (Table 3). Validation of these genes was performed through RNA-seq approaches using the publicly available transcriptome dataset [24]. Our validation studies underscored the requirement to trigger the early onset of oil biosynthesis during seed development to have a longer duration of oil accumulation, resulting in higher oil yield. This is especially required when the anthesis-capsule maturity window period does not vary much. Therefore, identifying genotypes with early onset (prior 10 DPA) and sustained expression patterns until 30 DPA for the genes involving oil biosynthesis would help enhance oil yield in the oilseed crop sesame.

## Figures and Tables

**Table 1:** Conserved histidine-rich boxes for the FAD family members identified in sesame.

Protein Name	Histidine Box-1		Histidine Box-2		Histidine Box-3		Membrane Nature
	Conserved Motif	AA Position	Conserved Motif	AA Position	Conserved Motif	AA Position	
*Si*SAD-1	EENRHG	175–180	DEKRHE	261–266	-	-	soluble
*Si*SAD-2	EENRHG	175–180	DEKRHE	261–266	-	-	soluble
*Si*SAD-3	EENRHG	169–174	DEKRHE	255–260	-	-	soluble
*Si*SAD-4	EENRHG	169–174	DEKRHE	255–260	-	-	soluble
*Si*SAD-5	EENRHG	160–165	DEKRHE	246–251	-	-	soluble
*Si*SAD-6	EENRHG	159–164	DEKRHE	245–250	-	-	soluble
*Si*SAD-7	EENRHG	166–171	DEKRHE	252–257	-	-	soluble
*Si*FAD2-1	HECGH	105–109	HRRHH	141–145	HVTHH	315–319	membrane-bound
*Si*FAD2-2	HECGH	105–109	HRRHH	141–145	HVAHH	315–319	membrane-bound
*Si*FAD3	HDCGH	123–127	HKTHH	159–163	HVIHH	326–330	membrane-bound
*Si*FAD6	HDCAH	167–171	HDRHH	203–207	HIPHH	363–367	membrane-bound
*Si*FAD7	HDCGH	167–171	HRTHH	203–207	HVIHH	370–374	membrane-bound
*Si*FAD8	HDCGH	161–165	HRTHH	197–201	HVIHH	364–368	membrane-bound

**Table 2:** Comparison of the amino acid residues of the catalytic sites of *Si*SADs with cell highlights showing a different AA when compared to *Rc*SAD1 and *At*FAB2 sequences.

*Rc*	*At*			*Mu*	*Si*						
SAD1	FAB2	AAD2	AAD3	PAD	SAD1	SAD2	SAD3	SAD4	SAD5	SAD6	SAD7
M147	M152	T157	T149	M147	M147	M147	M141	M141	M132	T131	M138
L148	L153	L158	L150	L148	L148	L148	I142	L142	I133	I132	I139
T150	T155	T160	T152	T150	T150	T150	T144	T144	T135	A134	T141
L151	L156	L161	L153	W151	L151	L151	L145	L145	L136	C135	C142
P212	P217	S222	S214	P212	P212	P212	P206	P206	P197	V196	I203
T214	T219	F224	F216	T214	T214	T214	T208	T208	T199	W198	L205
G221	G226	G231	G223	G221	G221	G221	G215	G215	G206	T205	T212
F222	F227	F232	F224	F222	F222	F222	F216	F216	F207	F206	F213

***Rc:** Ricinus communis; **At:** Arabidopsis thaliana; **Mu:** Macfadyena unguis-cati; **Si:** Sesamum indicum.*

**Table 3:** List of orthologs in sesame identified from genes reported in model plants as associated with improving oil content.

S. No	Gene Name	Genes from Model Plants	Effect on Oil Accumulation	Reference	Orthologs in *S indicum*
1	*ACCase*	Gh*accB1* (*Gossypium hirsutum*)	Overexpression driven by the seed specific AGP promoter improved cotton oil content by 9.19 to 21.92%	[47,48]	*SiaccB1*
		*Nt*accD (*Nicotiana tabacum*)	Chloroplast transformation with modified *acc*D operon increased seed and oil yield	[49]	*SiaccD*
2	*MCAT*	*AtFabD* (*Arabidopsis*)	Overexpression of *AtFabD* driven by the promoter of the senescence-associated 1 (SEN1) gene increased by 15–20% storage oil content of *Arabidopsis*	[50]	*SiFabD*
3	*KASI*	*NtFabB* (*Nicotiana tabacum*)	*NtKASI-1* overexpression enhanced oil accumulation	[15]	*SiFabB-2*
4	*KASII*	*Jc*FabF (*Jatropha curcas*)	Virus-induced gene silencing of *JcKASII* significantly altered TAG biosynthesis	[51]	*SiFabF-3*
5	*LACS*	*AtLACS4* and *AtLACS9* (*Arabidopsis*)	TAG content of the *lacs4 lacs9* double mutant seeds reduced by 27% compared to wild type seeds	[16]	*SiLACS4* and *SiLACS9*
6	*GPAT*	At*GPAT9* (*Arabidopsis*)	Knockdown of *AtGPAT9* resulted in 26% to 44% reduced seed oil content	[52]	*SiGPAT5* and *SiGPAT6*
7	*LPAAT*	*Ah*LPAAT2 (*Arachis hypogea*)	Seed-specific overexpression of *AhLPAT2* in *Arabidopsis* increased oil content by 7.4% in transgenic plants	[53]	*SiLPAAT2-1* and *SiLPAAT2-2*
		*Bn*LPAAT2-2 and *Bn*LPAAT2-4 (*Brasica napus*)	Overexpression of rapeseed *LPAAT* genes in *Arabidopsis* resulted in transgenic seeds with 13% increased TAG content	[54]	
8	*DGAT1*	AtDGAT1	Overexpression of At*DGAT1* under the control of a seed-specific promoter in *Arabidopsis* and canola increased seed oil content by 28 and 16%, respectively	[55]	*SiDGAT1*
		CsDGAT1B (*Camelina sativa*)	~24% increase in seed oil content in transgenic *C. sativa* overexpressing *CsDGAT1B*	[56]	
		*TmDGAT1* (*Tropaeolum majus*)	Overexpression of embryo-specific *TmDGAT1* increased the storage oil content in transgenic *Arabidopsis* and rapeseed by ~8 and ~15%, respectively	[57]	
		BnDGAT1 (*Brasica napus*)	Seed-specific napin promoter drived overexpression in *Arabidopsis* and rapeseed increased oil content by ~5 and 14%, respectively	[58]	
9	*DGAT2*	*EgDGAT2* (*Elaeis guineensis*)	Hetrologously expressed *EgDAGT2* in mutant yeast H1246 restored TAG biosynthesis and overexpression of *EgDAGT2* in *Arabidopsis* increased the content of polyunsaturated FAs	[59]	*SiDGAT2*
		*GmDGAT2D* (*Glycine max*)	Overexpression of *Gm*DGAT2D in *Arabidopsis* increased TAG biosynthesis by 5.7%	[60]	

## Data Availability

All data pertaining to the study reported here are provided in the supplementary information.

## Supplementary Materials

The following supporting information can be downloaded at: https://www.mdpi.com/article/10.3390/plants11212980/s1, Figures S1–S36: ‘Fig_S1-S36_revAug2022.pptx’; Sequence file S1: ‘Seq_File_S1.docx’; Table S1: ‘Table_S1.docx’; Table S2: ‘Table_S2.docx’; Table S3: ‘Table_S3.xlsx’; Table S4: ‘Table_S4.xlsx’;

## References

[1] Bates P.D., Stymne S., Ohlrogge J. (2013). Biochemical pathways in seed oil synthesis. Curr. Opin. Plant Biol..

[2] Lu C., Napier J.A., Clemente T.E., Cahoon E.B. (2011). New frontiers in oilseed biotechnology: Meeting the global demand for vegetable oils for food, feed, biofuel, and industrial applications. Curr. Opin. Biotechnol..

[3] Bruinsma J. (2017). World Agriculture: Towards 2015/2030-An FAO Perspective.

[4] Banaś W., Sanchez Garcia A., Banaś A., Stymne S. (2013). Activities of acyl-CoA: Diacylglycerol acyltransferase (DGAT) and phospholipid: Diacylglycerol acyltransferase (PDAT) in microsomal preparations of developing sunflower and safflower seeds. Planta.

[5] Rangan P., Maurya R., Singh S. (2022). Can omic tools help generate alternative newer sources of edible seed oil?. Plant Direct.

[6] Saha S., Enugutti B., Rajakumari S., Rajasekharan R. (2006). Cytosolic triacylglycerol biosynthetic pathway in oilseeds. Molecular cloning and expression of peanut cytosolic diacylglycerol acyltransferase. Plant Physiol..

[7] Bhatt-Wessel B., Jordan T.W., Miller J.H., Peng L. (2018). Role of DGAT enzymes in triacylglycerol metabolism. Arch. Biochem. Biophys..

[8] Nikolau B.J., Ohlrogge J.B., Wurtele E.S. (2003). Plant biotin-containing carboxylases. Arch. Biochem. Biophys..

[9] Ohlrogge J., Browse J. (1995). Lipid biosynthesis. Plant Cell.

[10] Bates P.D., Browse J. (2012). The significance of different diacylgycerol synthesis pathways on plant oil composition and bioengineering. Front. Plant Sci..

[11] Sasaki Y., Nagano Y. (2004). Plant acetyl-CoA carboxylase: Structure, biosynthesis, regulation, and gene manipulation for plant breeding. Biosci. Biotechnol. Biochem..

[12] Reverdatto S., Beilinson V., Nielsen N.C. (1999). A multisubunit acetyl coenzyme A carboxylase from soybean. Plant Physiol..

[13] Li M.-J., Li A.-Q., Xia H., Zhao C.-Z., Li C.-S., Wan S.-B., Bi Y.-P., Wang X.-J. (2009). Cloning and sequence analysis of putative type II fatty acid synthase genes from *Arachis hypogaea* L.. J. Biosci..

[14] White S.W., Zheng J., Zhang Y.-M., Rock C.O. (2005). The structural biology of type II fatty acid biosynthesis. Annu. Rev. Biochem..

[15] Yang T., Xu R., Chen J., Liu A. (2016). β-Ketoacyl-acyl carrier protein synthase I (KASI) plays crucial roles in the plant growth and fatty acids synthesis in tobacco. Int. J. Mol. Sci..

[16] Jessen D., Roth C., Wiermer M., Fulda M. (2015). Two activities of long-chain acyl-coenzyme A synthetase are involved in lipid trafficking between the endoplasmic reticulum and the plastid in Arabidopsis. Plant Physiol..

[17] Dong C.-J., Cao N., Zhang Z.-G., Shang Q.-M. (2016). Characterization of the fatty acid desaturase genes in cucumber: Structure, phylogeny, and expression patterns. PLoS ONE.

[18] Lightner J., Wu J., Browse J. (1994). A mutant of Arabidopsis with increased levels of stearic acid. Plant Physiol..

[19] Bryant F.M., Munoz-Azcarate O., Kelly A.A., Beaudoin F., Kurup S., Eastmond P.J. (2016). ACYL-ACYL CARRIER PROTEIN DESATURASE2 and 3 are responsible for making omega-7 fatty acids in the Arabidopsis aleurone. Plant Physiol..

[20] Dar A.A., Choudhury A.R., Kancharla P.K., Arumugam N. (2017). The FAD2 gene in plants: Occurrence, regulation, and role. Front. Plant Sci..

[21] Kennedy E.P. (1961). Biosynthesis of complex lipids. Fed. Proc..

[22] Dossa K., Diouf D., Wang L., Wei X., Zhang Y., Niang M., Fonceka D., Yu J., Mmadi M.A., Yehouessi L.W. (2017). The emerging oilseed crop *Sesamum indicum* enters the “Omics” era. Front. Plant Sci..

[23] Bedigian D. (2004). History and lore of sesame in Southwest Asia. Econ. Bot..

[24] Wang L., Zhang Y., Li D., Dossa K., Wang M.L., Zhou R., Yu J., Zhang X. (2019). Gene expression profiles that shape high and low oil content sesames. BMC Genet..

[25] Wei X., Liu K., Zhang Y., Feng Q., Wang L., Zhao Y., Li D., Zhao Q., Zhu X., Zhu X. (2015). Genetic discovery for oil production and quality in sesame. Nat. Commun..

[26] Ikai A. (1980). Thermostability and aliphatic index of globular proteins. J. Biochem..

[27] Kumar S., Stecher G., Li M., Knyaz C., Tamura K. (2018). MEGA X: Molecular evolutionary genetics analysis across computing platforms. Mol. Biol. Evol..

[28] Keatinge-Clay A.T., Shelat A.A., Savage D.F., Tsai S.-C., Miercke L.J., O’Connell J.D., Khosla C., Stroud R.M. (2003). Catalysis, specificity, and ACP docking site of Streptomyces coelicolor malonyl-CoA: ACP transacylase. Structure.

[29] Von Wettstein-Knowles P., Olsen J.G., McGuire K.A., Henriksen A. (2006). Fatty acid synthesis: Role of active site histidines and lysine in Cys-His-His-type β-ketoacyl-acyl carrier protein synthases. FEBS J..

[30] Lindqvist Y., Huang W., Schneider G., Shanklin J. (1996). Crystal structure of delta9 stearoyl-acyl carrier protein desaturase from castor seed and its relationship to other di-iron proteins. EMBO J..

[31] Troncoso-Ponce M.A., Barthole G., Tremblais G., To A., Miquel M., Lepiniec L., Baud S. (2016). Transcriptional activation of two delta-9 palmitoyl-ACP desaturase genes by MYB115 and MYB118 is critical for biosynthesis of omega-7 monounsaturated fatty acids in the endosperm of Arabidopsis seeds. Plant Cell.

[32] Cahoon E.B., Shah S., Shanklin J., Browse J. (1998). A determinant of substrate specificity predicted from the acyl-acyl carrier protein desaturase of developing cat’s claw seed. Plant Physiol..

[33] Wu T., Fu Y., Shi Y., Li Y., Kou Y., Mao X., Liu J. (2020). Functional characterization of long-chain acyl-CoA synthetase gene family from the oleaginous alga *Chromochloris zofingiensis*. J. Agric. Food Chem..

[34] Mañas-Fernández A., Li-Beisson Y., Alonso D.L., García-Maroto F. (2010). Cloning and molecular characterization of a glycerol-3-phosphate O-acyltransferase (GPAT) gene from *Echium* (Boraginaceae) involved in the biosynthesis of cutin polyesters. Planta.

[35] Koerbes A.P., Kulcheski F.R., Margis R., Margis-Pinheiro M., Turchetto-Zolet A.C. (2016). Molecular evolution of the lysophosphatidic acid acyltransferase (LPAAT) gene family. Mol. Phylogenetics Evol..

[36] Higo K., Ugawa Y., Iwamoto M., Korenaga T. (1999). Plant cis-acting regulatory DNA elements (PLACE) database: 1999. Nucleic Acids Res..

[37] Kumar N., Chaudhary A., Singh D., Teotia S. (2020). Transcriptional regulation of seed oil accumulation in *Arabidopsis thaliana*: Role of transcription factors and chromatin remodelers. J. Plant Biochem. Biotechnol..

[38] Niu Y., Zhang G., Wan F., Zhang Y.-M. (2020). Integration of RNA-Seq profiling with genome-wide association study predicts candidate genes for oil accumulation in soybean. Crop Pasture Sci..

[39] Mehrotra R., Jain V., Shekhar C., Mehrotra S. (2014). Genome wide analysis of *Arabidopsis thaliana* reveals high frequency of AAAGN7CTTT motif. Meta Gene.

[40] Wang H.W., Zhang B., Hao Y.J., Huang J., Tian A.G., Liao Y., Zhang J.S., Chen S.Y. (2007). The soybean Dof-type transcription factor genes, *GmDof4* and *GmDof11*, enhance lipid content in the seeds of transgenic Arabidopsis plants. Plant J..

[41] Mu J., Tan H., Zheng Q., Fu F., Liang Y., Zhang J., Yang X., Wang T., Chong K., Wang X.-J. (2008). *LEAFY COTYLEDON1* is a key regulator of fatty acid biosynthesis in Arabidopsis. Plant Physiol..

[42] Baud S., Mendoza M.S., To A., Harscoët E., Lepiniec L., Dubreucq B. (2007). WRINKLED1 specifies the regulatory action of LEAFY COTYLEDON2 towards fatty acid metabolism during seed maturation in Arabidopsis. Plant J..

[43] Behera J.R., Rahman M.M., Bhatia S., Shockey J., Kilaru A. (2021). Functional and predictive structural characterization of WRINKLED2, a unique oil biosynthesis regulator in avocado. Front. Plant Sci..

[44] Lee H.G., Kim H., Suh M.C., Kim H.U., Seo P.J. (2018). The MYB96 transcription factor regulates triacylglycerol accumulation by activating DGAT1 and PDAT1 expression in Arabidopsis seeds. Plant Cell Physiol..

[45] Lee K., Lee H.G., Yoon S., Kim H.U., Seo P.J. (2015). The Arabidopsis MYB96 transcription factor is a positive regulator of ABSCISIC ACID-INSENSITIVE4 in the control of seed germination. Plant Physiol..

[46] Kong Y., Chen S., Yang Y., An C. (2013). ABA-insensitive (ABI) 4 and ABI5 synergistically regulate DGAT1 expression in Arabidopsis seedlings under stress. FEBS Lett..

[47] Cui Y., Liu Z., Zhao Y., Wang Y., Huang Y., Li L., Wu H., Xu S., Hua J. (2017). Overexpression of heteromeric GhACCase subunits enhanced oil accumulation in upland cotton. Plant Mol. Biol. Report..

[48] Cui Y., Zhao Y., Wang Y., Liu Z., Ijaz B., Huang Y., Hua J. (2017). Genome-wide identification and expression analysis of the biotin carboxyl carrier subunits of heteromeric acetyl-CoA carboxylase in Gossypium. Front. Plant Sci..

[49] Madoka Y., Tomizawa K.-I., Mizoi J., Nishida I., Nagano Y., Sasaki Y. (2002). Chloroplast transformation with modified accD operon increases acetyl-CoA carboxylase and causes extension of leaf longevity and increase in seed yield in tobacco. Plant Cell Physiol..

[50] Jung S.H., Kim R.J., Kim K.J., Lee D.H., Suh M.C. (2019). Plastidial and mitochondrial malonyl CoA-ACP malonyltransferase is essential for cell division and its overexpression increases storage oil content. Plant Cell Physiol..

[51] Ye J., Qu J., Bui H.T.N., Chua N.H. (2009). Rapid analysis of *Jatropha curcas* gene functions by virus-induced gene silencing. Plant Biotechnol. J..

[52] Shockey J., Regmi A., Cotton K., Adhikari N., Browse J., Bates P.D. (2016). Identification of Arabidopsis GPAT9 (At5g60620) as an essential gene involved in triacylglycerol biosynthesis. Plant Physiol..

[53] Chen S., Lei Y., Xu X., Huang J., Jiang H., Wang J., Cheng Z., Zhang J., Song Y., Liao B. (2015). The peanut (*Arachis hypogaea* L.) gene AhLPAT2 increases the lipid content of transgenic Arabidopsis seeds. PLoS ONE.

[54] Maisonneuve S., Bessoule J.-J., Lessire R., Delseny M., Roscoe T.J. (2010). Expression of rapeseed microsomal lysophosphatidic acid acyltransferase isozymes enhances seed oil content in Arabidopsis. Plant Physiol..

[55] Jako C., Kumar A., Wei Y., Zou J., Barton D.L., Giblin E.M., Covello P.S., Taylor D.C. (2001). Seed-specific over-expression of an Arabidopsis cDNA encoding a diacylglycerol acyltransferase enhances seed oil content and seed weight. Plant Physiol..

[56] Kim H., Park J.H., Kim D.J., Kim A.Y., Suh M.C. (2016). Functional analysis of *diacylglycerol acyltransferase1* genes from Camelina sativa and effects of CsDGAT1B overexpression on seed mass and storage oil content in *C. sativa*. Plant Biotechnol. Rep..

[57] Xu J., Francis T., Mietkiewska E., Giblin E.M., Barton D.L., Zhang Y., Zhang M., Taylor D.C. (2008). Cloning and characterization of an acyl-CoA-dependent diacylglycerol acyltransferase 1 (DGAT1) gene from Tropaeolum majus, and a study of the functional motifs of the DGAT protein using site-directed mutagenesis to modify enzyme activity and oil content. Plant Biotechnol. J..

[58] Weselake R.J., Shah S., Tang M., Quant P.A., Snyder C.L., Furukawa-Stoffer T.L., Zhu W., Taylor D.C., Zou J., Kumar A. (2008). Metabolic control analysis is helpful for informed genetic manipulation of oilseed rape (*Brassica napus*) to increase seed oil content. J. Exp. Bot..

[59] Jin Y., Yuan Y., Gao L., Sun R., Chen L., Li D., Zheng Y. (2017). Characterization and functional analysis of a type 2 diacylglycerol acyltransferase (DGAT2) gene from oil palm (*Elaeis guineensis* Jacq.) mesocarp in Saccharomyces cerevisiae and transgenic *Arabidopsis thaliana*. Front. Plant Sci..

[60] Chen B., Wang J., Zhang G., Liu J., Manan S., Hu H., Zhao J. (2016). Two types of soybean diacylglycerol acyltransferases are differentially involved in triacylglycerol biosynthesis and response to environmental stresses and hormones. Sci. Rep..

[61] Liang Y., Huang Y., Chen K., Kong X., Li M. (2022). Characterization of non-specific lipid transfer protein (nsLtp) gene families in the Brassica napus pangenome reveals abundance variation. BMC Plant Biol..

[62] Alotaibi S.S., Elseehy M.M., Aljuaid B.S., El-Shehawi A.M. (2020). Transcriptome analysis of jojoba (*Simmondsia chinensis*) during seed development and liquid wax ester biosynthesis. Plants.

[63] Javelle M., Vernoud V., Rogowsky P.M., Ingram G.C. (2011). Epidermis: The formation and functions of a fundamental plant tissue. New Phytol..

[64] Samuels L., Kunst L., Jetter R. (2008). Sealing plant surfaces: Cuticular wax formation by epidermal cells. Annu. Rev. Plant Biol..

[65] Nováková E., Zablatzká L., Brus J., Nesrstová V., Hanáček P., Kalendar R., Cvrčková F., Majeský Ľ., Smýkal P. (2019). Allelic diversity of acetyl coenzyme A carboxylase accD/bccp genes implicated in nuclear-cytoplasmic conflict in the wild and domesticated pea (*Pisum* sp.). Int. J. Mol. Sci..

[66] Xu L., Zeng W., Li J., Liu H., Yan G., Si P., Yang C., Shi Y., He Q., Zhou W. (2019). Characteristics of membrane-bound fatty acid desaturase (FAD) genes in *Brassica napus* L. and their expressions under different cadmium and salinity stresses. Environ. Exp. Bot..

[67] Li J., Liu A., Najeeb U., Zhou W., Liu H., Yan G., Gill R.A., Yun X., Bai Q., Xu L. (2021). Genome-wide investigation and expression analysis of membrane-bound fatty acid desaturase genes under different biotic and abiotic stresses in sunflower (*Helianthus annuus* L.). Int. J. Biol. Macromol..

[68] Heidari P., Ahmadizadeh M., Izanlo F., Nussbaumer T. (2019). In silico study of the CESA and CSL gene family in *Arabidopsis thaliana* and *Oryza sativa*: Focus on post-translation modifications. Plant Gene.

[69] Kyte J., Doolittle R.F. (1982). A simple method for displaying the hydropathic character of a protein. J. Mol. Biol..

[70] González-Mellado D., von Wettstein-Knowles P., Garcés R., Martínez-Force E. (2010). The role of β-ketoacyl-acyl carrier protein synthase III in the condensation steps of fatty acid biosynthesis in sunflower. Planta.

[71] Chi X., Zhang Z., Chen N., Zhang X., Wang M., Chen M., Wang T., Pan L., Chen J., Yang Z. (2017). Isolation and functional analysis of fatty acid desaturase genes from peanut (*Arachis hypogaea* L.). PLoS ONE.

[72] Chi X., Yang Q., Lu Y., Wang J., Zhang Q., Pan L., Chen M., He Y., Yu S. (2011). Genome-wide analysis of fatty acid desaturases in soybean (*Glycine max*). Plant Mol. Biol. Report..

[73] Xue Y., Jiang J., Yang X., Jiang H., Du Y., Liu X., Xie R., Chai Y. (2020). Genome-wide mining and comparative analysis of fatty acid elongase gene family in *Brassica napus* and its progenitors. Gene.

[74] Ahmadizadeh M., Rezaee S., Heidari P. (2020). Genome-wide characterization and expression analysis of fatty acid desaturase gene family in *Camelina sativa*. Gene Rep..

[75] Liu B., Sun Y., Xue J., Mao X., Jia X., Li R. (2019). Stearoyl-ACP Δ9 desaturase 6 and 8 (GhA-SAD6 and GhD-SAD8) are responsible for biosynthesis of palmitoleic acid specifically in developing endosperm of upland cotton seeds. Front. Plant Sci..

[76] Kryuchkova-Mostacci N., Robinson-Rechavi M. (2016). Tissue-specificity of gene expression diverges slowly between orthologs, and rapidly between paralogs. PLoS Comput. Biol..

[77] Kryuchkova-Mostacci N., Robinson-Rechavi M. (2017). A benchmark of gene expression tissue-specificity metrics. Brief. Bioinform..

[78] Jin U.-H., Lee J.-W., Chung Y.-S., Lee J.-H., Yi Y.-B., Kim Y.-K., Hyung N.-I., Pyee J.-H., Chung C.-H. (2001). Characterization and temporal expression of a ω-6 fatty acid desaturase cDNA from sesame (*Sesamum indicum* L.) seeds. Plant Sci..

[79] Shanklin J., Whittle E., Fox B.G. (1994). Eight histidine residues are catalytically essential in a membrane-associated iron enzyme, stearoyl-CoA desaturase, and are conserved in alkane hydroxylase and xylene monooxygenase. Biochemistry.

[80] Scarth R., Tang J. (2006). Modification of Brassica oil using conventional and transgenic approaches. Crop Sci..

[81] Liu F., Zhao Y.-P., Zhu H.-g., Zhu Q.-H., Sun J. (2017). Simultaneous silencing of GhFAD2-1 and GhFATB enhances the quality of cottonseed oil with high oleic acid. J. Plant Physiol..

[82] Clemente T.E., Cahoon E.B. (2009). Soybean oil: Genetic approaches for modification of functionality and total content. Plant Physiol..

[83] Qu C., Jiang H., Zhang L. (2014). Cloning and enzymatic activity analysis of the malonyl-CoA: Acyl carrier protein transacylase in *Brassica napus* cultivars with different oil content. Am. J. Plant Sci..

[84] You F.M., Li P., Kumar S., Ragupathy R., Li Z., Fu Y.-B., Cloutier S. (2014). Genome-wide identification and characterization of the gene families controlling fatty acid biosynthesis in flax (*Linum usitatissimum* L). J Proteom. Bioinform.

[85] Wu G.-Z., Xue H.-W. (2010). Arabidopsis β-ketoacyl-[acyl carrier protein] synthase I is crucial for fatty acid synthesis and plays a role in chloroplast division and embryo development. Plant Cell.

[86] Salas J.N.J., Ohlrogge J.B. (2002). Characterization of substrate specificity of plant FatA and FatB acyl-ACP thioesterases. Arch. Biochem. Biophys..

[87] Bhattacharya S., Sinha S., Das N., Maiti M.K. (2015). Increasing the stearate content in seed oil of *Brassica juncea* by heterologous expression of MlFatB affects lipid content and germination frequency of transgenic seeds. Plant Physiol. Biochem..

[88] Eccleston V.S., Cranmer A.M., Voelker T.A., Ohlrogge J.B. (1996). Medium-chain fatty acid biosynthesis and utilization in *Brassica napus* plants expressing lauroyl-acyl carrier protein thioesterase. Planta.

[89] Koonin E.V. (2005). Orthologs, paralogs, and evolutionary genomics. Annu. Rev. Genet..

[90] Fujimoto M.S., Suvorov A., Jensen N.O., Clement M.J., Bybee S.M. (2016). Detecting false positive sequence homology: A machine learning approach. BMC Bioinform..

[91] McGimpsey S. (2019). The Twilight Zone of Nucleotide Homology. Ph.D. Thesis.

[92] Pearson W.R. (2013). An introduction to sequence similarity (“homology”) searching. Curr. Protoc. Bioinform..

[93] Movahedi S., Van de Peer Y., Vandepoele K. (2011). Comparative network analysis reveals that tissue specificity and gene function are important factors influencing the mode of expression evolution in Arabidopsis and rice. Plant Physiol..

[94] Kille B., Balaji A., Sedlazeck F.J., Nute M., Treangen T.J. (2022). Multiple genome alignment in the telomere-to-telomere assembly era. Genome Biol..

[95] Shockey J.M., Fulda M.S., Browse J.A. (2002). Arabidopsis contains nine long-chain acyl-coenzyme a synthetase genes that participate in fatty acid and glycerolipid metabolism. Plant Physiol..

[96] Ayaz A., Saqib S., Huang H., Zaman W., Lü S., Zhao H. (2021). Genome-wide comparative analysis of long-chain acyl-CoA synthetases (LACSs) gene family: A focus on identification, evolution and expression profiling related to lipid synthesis. Plant Physiol. Biochem..

[97] Aznar-Moreno J.A., Venegas Caleron M., Martínez-Force E., Garcés R., Mullen R., Gidda S.K., Salas J.J. (2014). Sunflower (*Helianthus annuus*) long-chain acyl-coenzyme A synthetases expressed at high levels in developing seeds. Physiol. Plant..

[98] Ding L.-N., Gu S.-L., Zhu F.-G., Ma Z.-Y., Li J., Li M., Wang Z., Tan X.-L. (2020). Long-chain acyl-CoA synthetase 2 is involved in seed oil production in *Brassica napus*. BMC Plant Biol..

[99] Xu Y., Holic R., Li D., Pan X., Mietkiewska E., Chen G., Ozga J., Weselake R.J. (2018). Substrate preferences of long-chain acyl-CoA synthetase and diacylglycerol acyltransferase contribute to enrichment of flax seed oil with α-linolenic acid. Biochem. J..

[100] Bai Y., Shen Y., Zhang Z., Jia Q., Xu M., Zhang T., Fang H., Yu X., Li L., Liu D. (2021). A GPAT1 Mutation in Arabidopsis Enhances Plant Height but Impairs Seed Oil Biosynthesis. Int. J. Mol. Sci..

[101] Chen X., Snyder C.L., Truksa M., Shah S., Weselake R.J. (2011). sn-Glycerol-3-phosphate acyltransferases in plants. Plant Signal. Behav..

[102] Jayawardhane K.N., Singer S.D., Weselake R.J., Chen G. (2018). Plant sn-glycerol-3-phosphate acyltransferases: Biocatalysts involved in the biosynthesis of intracellular and extracellular lipids. Lipids.

[103] Yang W., Simpson J.P., Li-Beisson Y., Beisson F., Pollard M., Ohlrogge J.B. (2012). A land-plant-specific glycerol-3-phosphate acyltransferase family in Arabidopsis: Substrate specificity, sn-2 preference, and evolution. Plant Physiol..

[104] Zheng Z., Xia Q., Dauk M., Shen W., Selvaraj G., Zou J. (2003). Arabidopsis AtGPAT1, a member of the membrane-bound glycerol-3-phosphate acyltransferase gene family, is essential for tapetum differentiation and male fertility. Plant Cell.

[105] Wang J., Singh S.K., Geng S., Zhang S., Yuan L. (2020). Genome-wide analysis of glycerol-3-phosphate O-acyltransferase gene family and functional characterization of two cutin group GPATs in *Brassica napus*. Planta.

[106] Cui Y., Ma J., Liu G., Wang N., Pei W., Wu M., Li X., Zhang J., Yu J. (2019). Genome-wide identification, sequence variation, and expression of the glycerol-3-phosphate acyltransferase (GPAT) gene family in *Gossypium*. Front. Genet..

[107] Kim H.U., Park M.-E., Lee K.-R., Suh M.C., Chen G.Q. (2020). Variant castor lysophosphatidic acid acyltransferases acylate ricinoleic acid in seed oil. Ind. Crops Prod..

[108] Frentzen M. (1998). Acyltransferases from basic science to modified seed oils. Lipid/Fett.

[109] Kim H.U., Huang A.H. (2004). Plastid lysophosphatidyl acyltransferase is essential for embryo development in Arabidopsis. Plant Physiol..

[110] Kim H.J., Silva J.E., Iskandarov U., Andersson M., Cahoon R.E., Mockaitis K., Cahoon E.B. (2015). Structurally divergent lysophosphatidic acid acyltransferases with high selectivity for saturated medium chain fatty acids from *Cuphea* seeds. Plant J..

[111] Rao S.S., Hildebrand D. (2009). Changes in oil content of transgenic soybeans expressing the yeast SLC1 gene. Lipids.

[112] Nakamura Y. (2017). Plant phospholipid diversity: Emerging functions in metabolism and protein–lipid interactions. Trends Plant Sci..

[113] Eastmond P.J., Quettier A.-L., Kroon J.T., Craddock C., Adams N., Slabas A.R. (2010). Phosphatidic Acid Phosphohydrolase 1 and 2 regulate phospholipid synthesis at the endoplasmic reticulum in *Arabidopsis*. Plant Cell.

[114] Pillai A.N., Shukla S., Rahaman A. (2017). An evolutionarily conserved phosphatidate phosphatase maintains lipid droplet number and endoplasmic reticulum morphology but not nuclear morphology. Biol. Open.

[115] Yoshitake Y., Sato R., Madoka Y., Ikeda K., Murakawa M., Suruga K., Sugiura D., Noguchi K., Ohta H., Shimojima M. (2017). Arabidopsis phosphatidic acid phosphohydrolases are essential for growth under nitrogen-depleted conditions. Front. Plant Sci..

[116] Gao H., Gao Y., Zhang F., Liu B., Ji C., Xue J., Yuan L., Li R. (2021). Functional characterization of an novel acyl-CoA: Diacylglycerol acyltransferase 3-3 (CsDGAT3-3) gene from *Camelina sativa*. Plant Sci..

[117] Zheng L., Shockey J., Bian F., Chen G., Shan L., Li X., Wan S., Peng Z. (2017). Variant amino acid residues alter the enzyme activity of peanut type 2 diacylglycerol acyltransferases. Front. Plant Sci..

[118] Chi X., Hu R., Zhang X., Chen M., Chen N., Pan L., Wang T., Wang M., Yang Z., Wang Q. (2014). Cloning and functional analysis of three diacylglycerol acyltransferase genes from peanut (*Arachis hypogaea* L.). PLoS ONE.

[119] McCartney A.W., Dyer J.M., Dhanoa P.K., Kim P.K., Andrews D.W., McNew J.A., Mullen R.T. (2004). Membrane-bound fatty acid desaturases are inserted co-translationally into the ER and contain different ER retrieval motifs at their carboxy termini. Plant J..

[120] Shockey J.M., Gidda S.K., Chapital D.C., Kuan J.-C., Dhanoa P.K., Bland J.M., Rothstein S.J., Mullen R.T., Dyer J.M. (2006). Tung tree DGAT1 and DGAT2 have nonredundant functions in triacylglycerol biosynthesis and are localized to different subdomains of the endoplasmic reticulum. Plant Cell.

[121] Lu C.L., de Noyer S.B., Hobbs D.H., Kang J., Wen Y., Krachtus D., Hills M.J. (2003). Expression pattern of diacylglycerol acyltransferase-1, an enzyme involved in triacylglycerol biosynthesis, in *Arabidopsis thaliana*. Plant Mol. Biol..

[122] Wang Z., Huang W., Chang J., Sebastian A., Li Y., Li H., Wu X., Zhang B., Meng F., Li W. (2014). Overexpression of SiDGAT1, a gene encoding acyl-CoA: Diacylglycerol acyltransferase from *Sesamum indicum* L. increases oil content in transgenic Arabidopsis and soybean. Plant Cell Tissue Organ Cult..

[123] Liu F., Xia Y., Wu L., Fu D., Hayward A., Luo J., Yan X., Xiong X., Fu P., Wu G. (2015). Enhanced seed oil content by overexpressing genes related to triacylglyceride synthesis. Gene.

[124] Abdullah H.M., Akbari P., Paulose B., Schnell D., Qi W., Park Y., Pareek A., Dhankher O.P. (2016). Transcriptome profiling of *Camelina sativa* to identify genes involved in triacylglycerol biosynthesis and accumulation in the developing seeds. Biotechnol. Biofuels.

[125] Zhou W., Song S., Dossou S.S.K., Zhou R., Wei X., Wang Z., Sheng C., Zhang Y., You J., Wang L. (2022). Genome-wide association analysis and transcriptome reveal novel loci and a candidate regulatory gene of fatty acid biosynthesis in sesame (*Sesamum indicum* L.). Plant Physiol. Biochem..

[126] Zhang Y.-P., Zhang Y.-Y., Thakur K., Zhang F., Hu F., Zhang J.-G., Wei P.-C., Wei Z.-J. (2021). Integration of miRNAs, Degradome, and Transcriptome Omics Uncovers a Complex Regulatory Network and Provides Insights Into Lipid and Fatty Acid Synthesis During Sesame Seed Development. Front. Plant Sci..

[127] Quettier A.-L., Eastmond P.J. (2009). Storage oil hydrolysis during early seedling growth. Plant Physiol. Biochem..

[128] Parthibane V., Rajakumari S., Venkateshwari V., Iyappan R., Rajasekharan R. (2012). Oleosin is bifunctional enzyme that has both monoacylglycerol acyltransferase and phospholipase activities. J. Biol. Chem..

[129] Li X., Xiao L., Wu G. (2008). Accumulation pattern of fatty acids during the seed development of sesame (*Sesamum indicum* L.). Chin. J. Oil Crop Sci..

[130] Wang L., Xia Q., Zhang Y., Zhu X., Zhu X., Li D., Ni X., Gao Y., Xiang H., Wei X. (2016). Updated sesame genome assembly and fine mapping of plant height and seed coat color QTLs using a new high-density genetic map. BMC Genom..

[131] Gasteiger E., Hoogland C., Gattiker A., Wilkins M.R., Appel R.D., Bairoch A., Walker J. (2005). Protein identification and analysis tools on the ExPASy server. The Proteomics Protocols Handbook.

[132] Almagro Armenteros J.J., Sønderby C.K., Sønderby S.K., Nielsen H., Winther O. (2017). DeepLoc: Prediction of protein subcellular localization using deep learning. Bioinformatics.

[133] Lu S., Wang J., Chitsaz F., Derbyshire M.K., Geer R.C., Gonzales N.R., Gwadz M., Hurwitz D.I., Marchler G.H., Song J.S. (2020). CDD/SPARCLE: The conserved domain database in 2020. Nucleic Acids Res..

[134] Mistry J., Chuguransky S., Williams L., Qureshi M., Salazar G.A., Sonnhammer E.L., Tosatto S.C., Paladin L., Raj S., Richardson L.J. (2021). Pfam: The protein families database in 2021. Nucleic Acids Res..

[135] Letunic I., Bork P. (2018). 20 years of the SMART protein domain annotation resource. Nucleic Acids Res..

[136] Madeira F., Park Y.M., Lee J., Buso N., Gur T., Madhusoodanan N., Basutkar P., Tivey A.R., Potter S.C., Finn R.D. (2019). The EMBL-EBI search and sequence analysis tools APIs in 2019. Nucleic Acids Res..

[137] Bailey T.L., Boden M., Buske F.A., Frith M., Grant C.E., Clementi L., Ren J., Li W.W., Noble W.S. (2009). MEME SUITE: Tools for motif discovery and searching. Nucleic Acids Res..

[138] Liu B., Hua C., Song G., Wu M., Cui R., Zhang A., Liu Y., Huang L., Yan A., Ali I. (2017). The SPATULA transcription factor regulates seed oil content by controlling seed specific genes in *Arabidopsis thaliana*. Plant Growth Regul..

[139] Chow C.-N., Lee T.-Y., Hung Y.-C., Li G.-Z., Tseng K.-C., Liu Y.-H., Kuo P.-L., Zheng H.-Q., Chang W.-C. (2019). PlantPAN3. 0: A new and updated resource for reconstructing transcriptional regulatory networks from ChIP-seq experiments in plants. Nucleic Acids Res..

[140] Andrews S. (2010). FastQC: A quality control tool for high throughput sequence data. Babraham Bioinformatics.

[141] Joshi N., Fass J. (2011). Sickle: A Sliding-Window, Adaptive, Quality-Based Trimming Tool for FastQ Files, Software Version 1.33. https://github.com/najoshi/sickle.

[142] Trapnell C., Pachter L., Salzberg S.L. (2009). TopHat: Discovering splice junctions with RNA-Seq. Bioinformatics.

[143] Trapnell C., Williams B.A., Pertea G., Mortazavi A., Kwan G., Van Baren M.J., Salzberg S.L., Wold B.J., Pachter L. (2010). Transcript assembly and quantification by RNA-Seq reveals unannotated transcripts and isoform switching during cell differentiation. Nat. Biotechnol..

[144] Trapnell C., Roberts A., Goff L., Pertea G., Kim D., Kelley D.R., Pimentel H., Salzberg S.L., Rinn J.L., Pachter L. (2012). Differential gene and transcript expression analysis of RNA-seq experiments with TopHat and Cufflinks. Nat. Protoc..

[145] Bioinformatics B., Valencia S. (2019). OmicsBox-Bioinformatics made easy. https://www.biobam.com/omicsbox/.

