# Supplementary material for: Longer Duration of Active Oil Biosynthesis during Seed Development Is Crucial for High Oil Yield—Lessons from Genome-Wide In Silico Mining and RNA-Seq Validation in Sesame
Source: Plants (Basel). 2022 Nov 4;11(21):2980. doi: 10.3390/plants11212980 (PMC9657858; doi:10.3390/plants11212980)
Supplement: Supplementary file 1 [file plants-11-02980-s001.zip › Fig_S1-S36_revAug2022.pptx]

## Slide 1
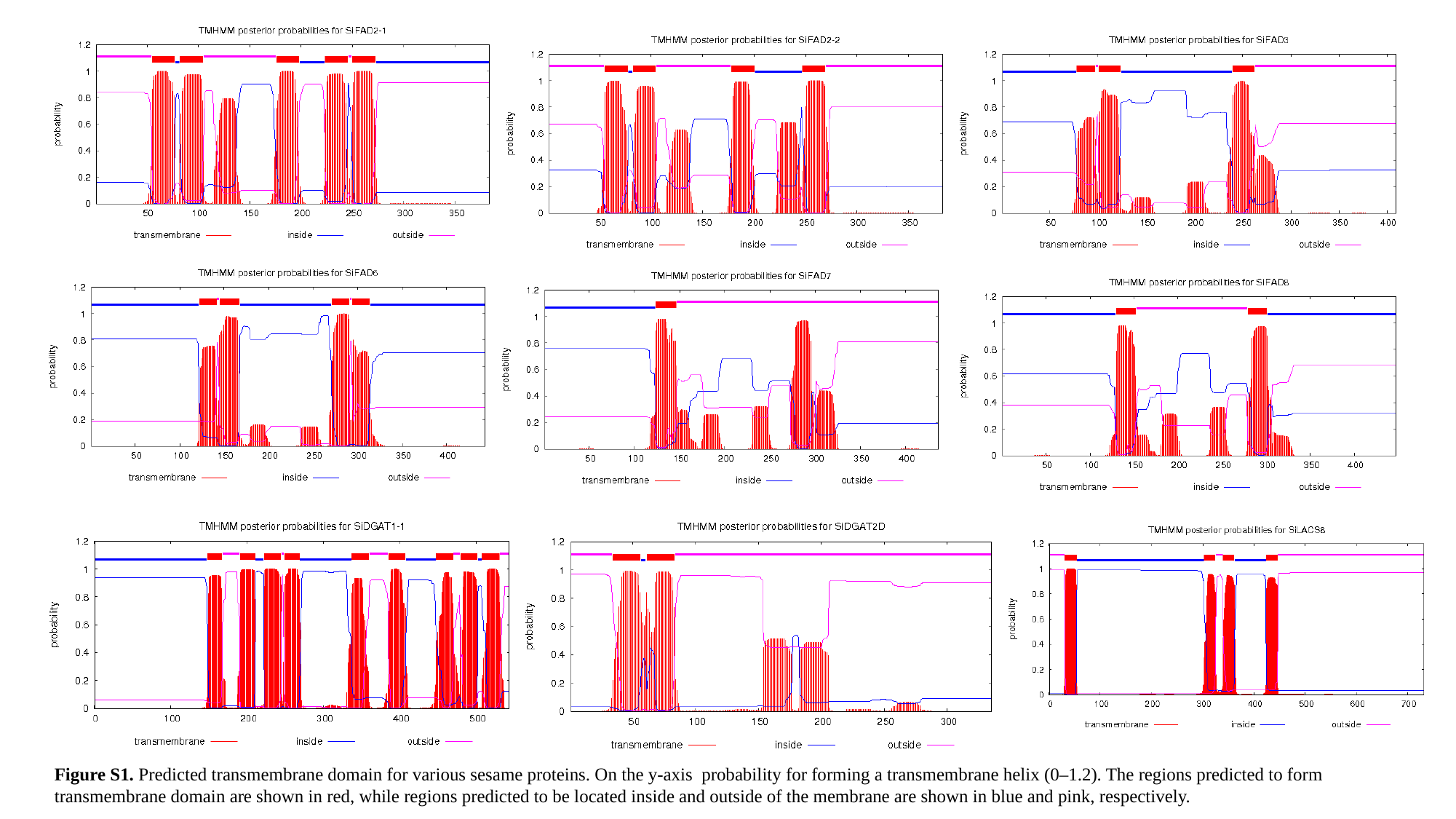

Figure S1. Predicted transmembrane domain for various sesame proteins. On the y-axis probability for forming a transmembrane helix (0–1.2). The regions predicted to form transmembrane domain are shown in red, while regions predicted to be located inside and outside of the membrane are shown in blue and pink, respectively.

## Slide 2
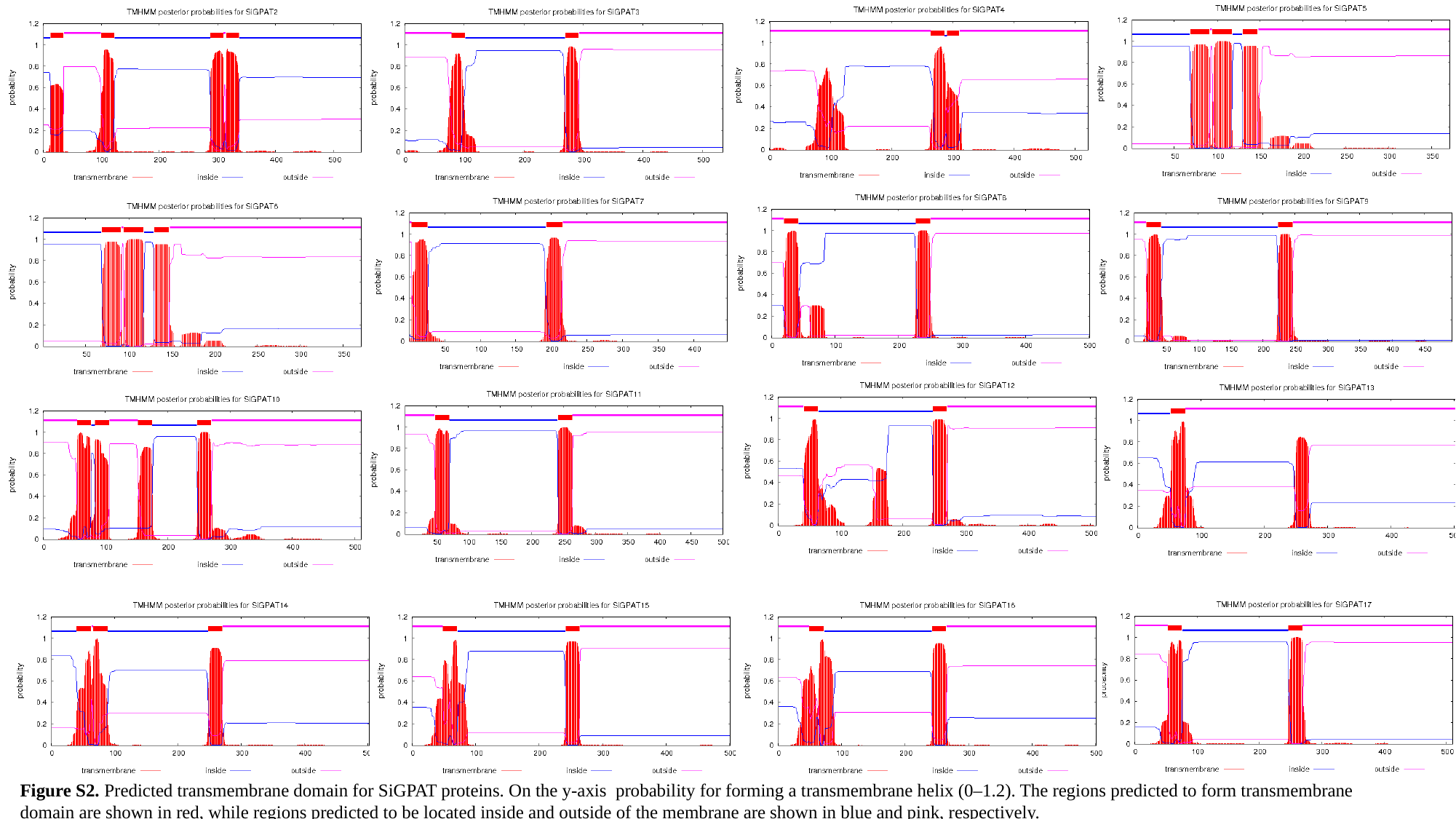

Figure S2. Predicted transmembrane domain for SiGPAT proteins. On the y-axis probability for forming a transmembrane helix (0–1.2). The regions predicted to form transmembrane domain are shown in red, while regions predicted to be located inside and outside of the membrane are shown in blue and pink, respectively.

## Slide 3
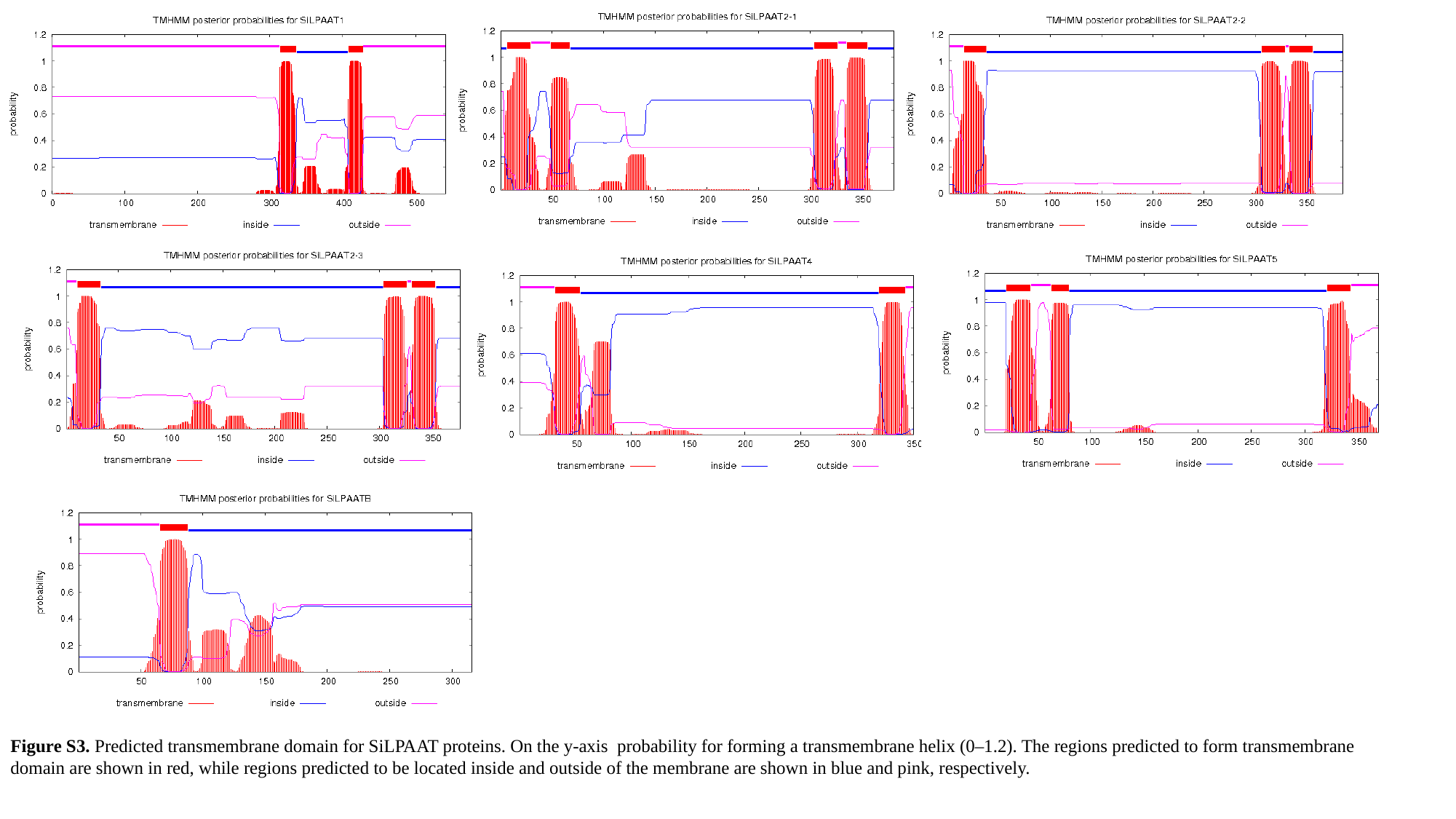

Figure S3. Predicted transmembrane domain for SiLPAAT proteins. On the y-axis probability for forming a transmembrane helix (0–1.2). The regions predicted to form transmembrane domain are shown in red, while regions predicted to be located inside and outside of the membrane are shown in blue and pink, respectively.

## Slide 4
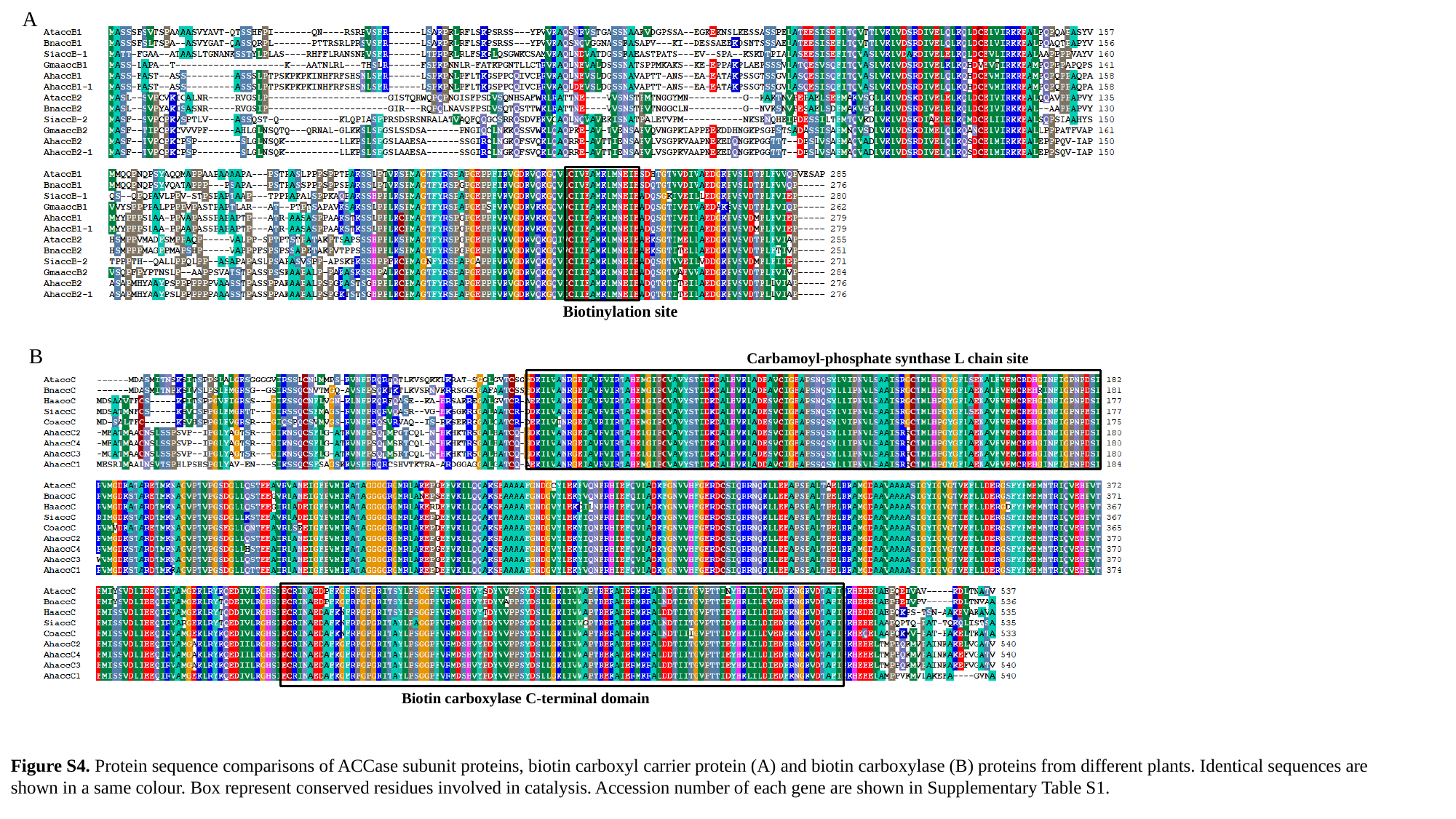

A
Biotinylation site
B
Carbamoyl-phosphate synthase L chain site
Biotin carboxylase C-terminal domain
Figure S4. Protein sequence comparisons of ACCase subunit proteins, biotin carboxyl carrier protein (A) and biotin carboxylase (B) proteins from different plants. Identical sequences are shown in a same colour. Box represent conserved residues involved in catalysis. Accession number of each gene are shown in Supplementary Table S1.

## Slide 5
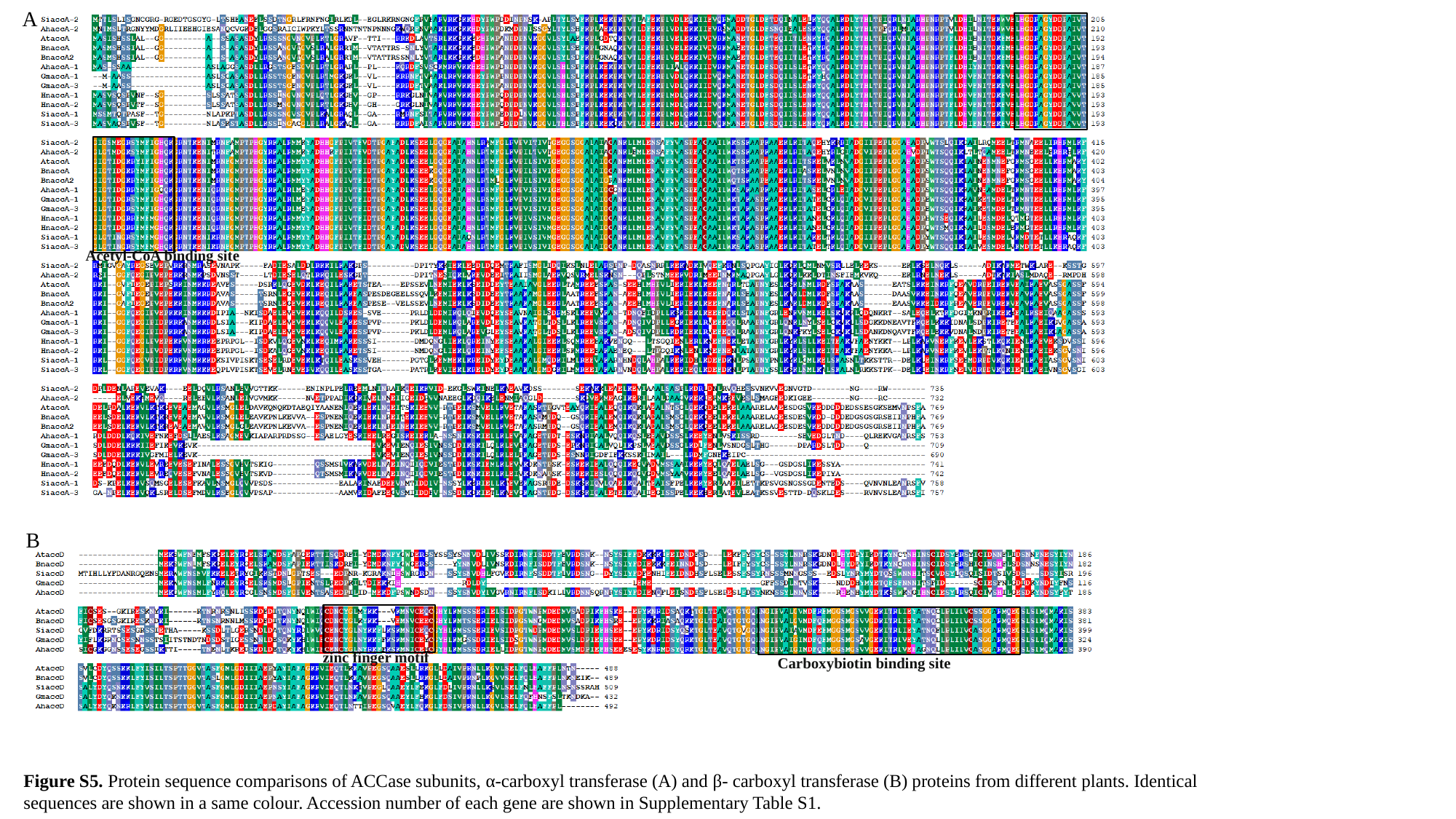

A
Acetyl-CoA binding site
B
zinc finger motif
Carboxybiotin binding site
Figure S5. Protein sequence comparisons of ACCase subunits, α-carboxyl transferase (A) and β- carboxyl transferase (B) proteins from different plants. Identical sequences are shown in a same colour. Accession number of each gene are shown in Supplementary Table S1.

## Slide 6
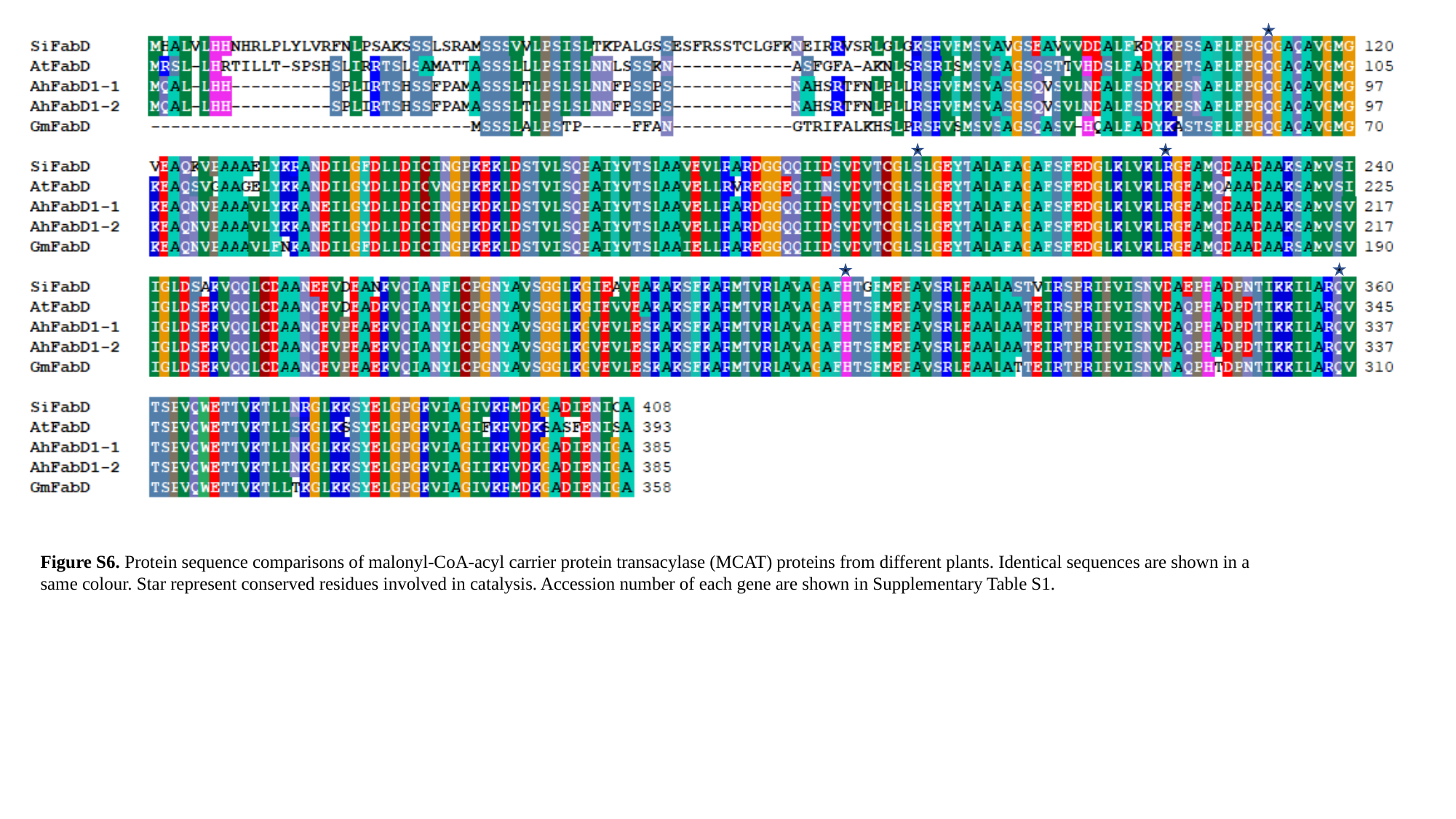

Figure S6. Protein sequence comparisons of malonyl-CoA-acyl carrier protein transacylase (MCAT) proteins from different plants. Identical sequences are shown in a same colour. Star represent conserved residues involved in catalysis. Accession number of each gene are shown in Supplementary Table S1.

## Slide 7
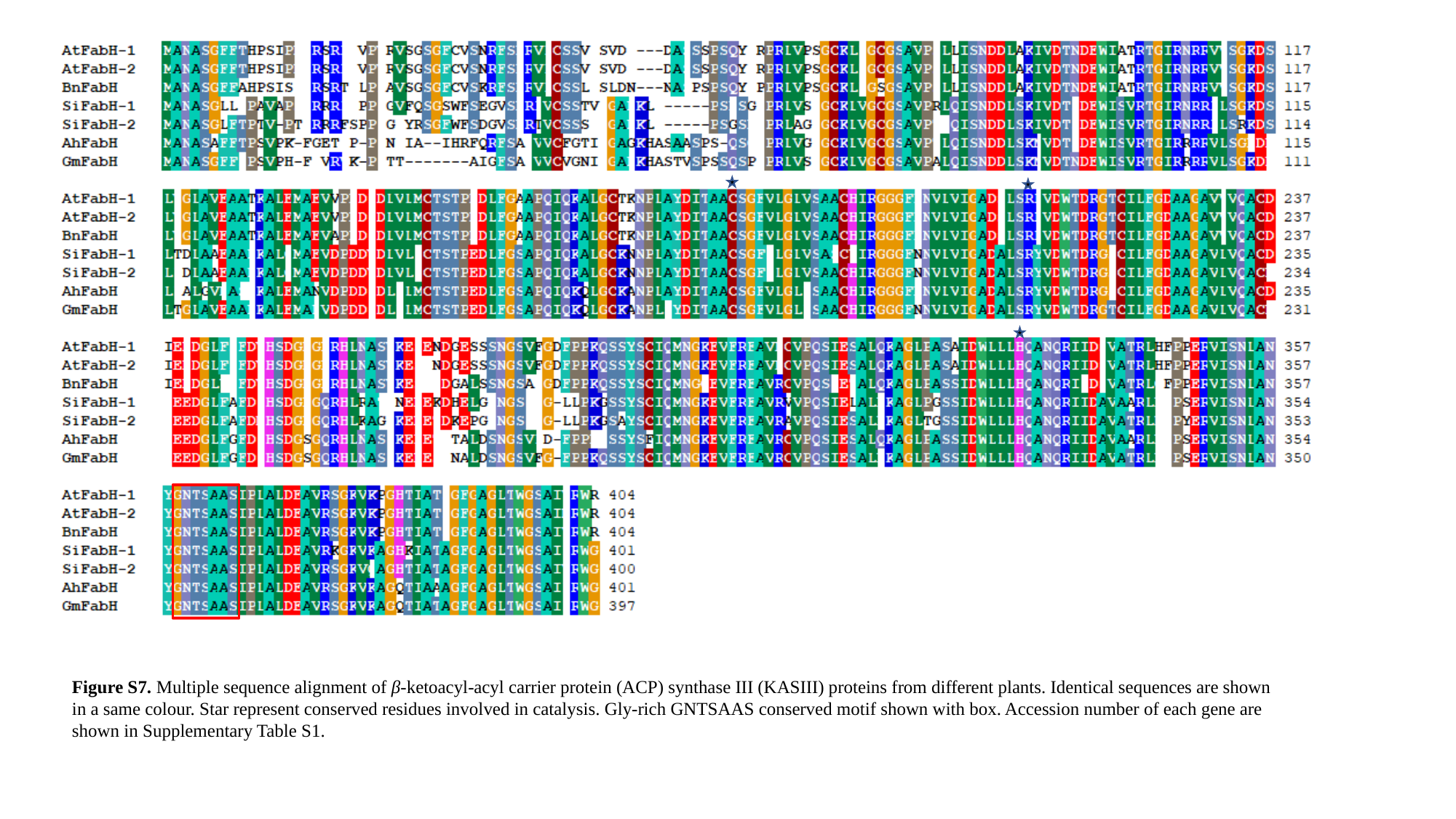

Figure S7. Multiple sequence alignment of β-ketoacyl-acyl carrier protein (ACP) synthase III (KASIII) proteins from different plants. Identical sequences are shown in a same colour. Star represent conserved residues involved in catalysis. Gly-rich GNTSAAS conserved motif shown with box. Accession number of each gene are shown in Supplementary Table S1.

## Slide 8
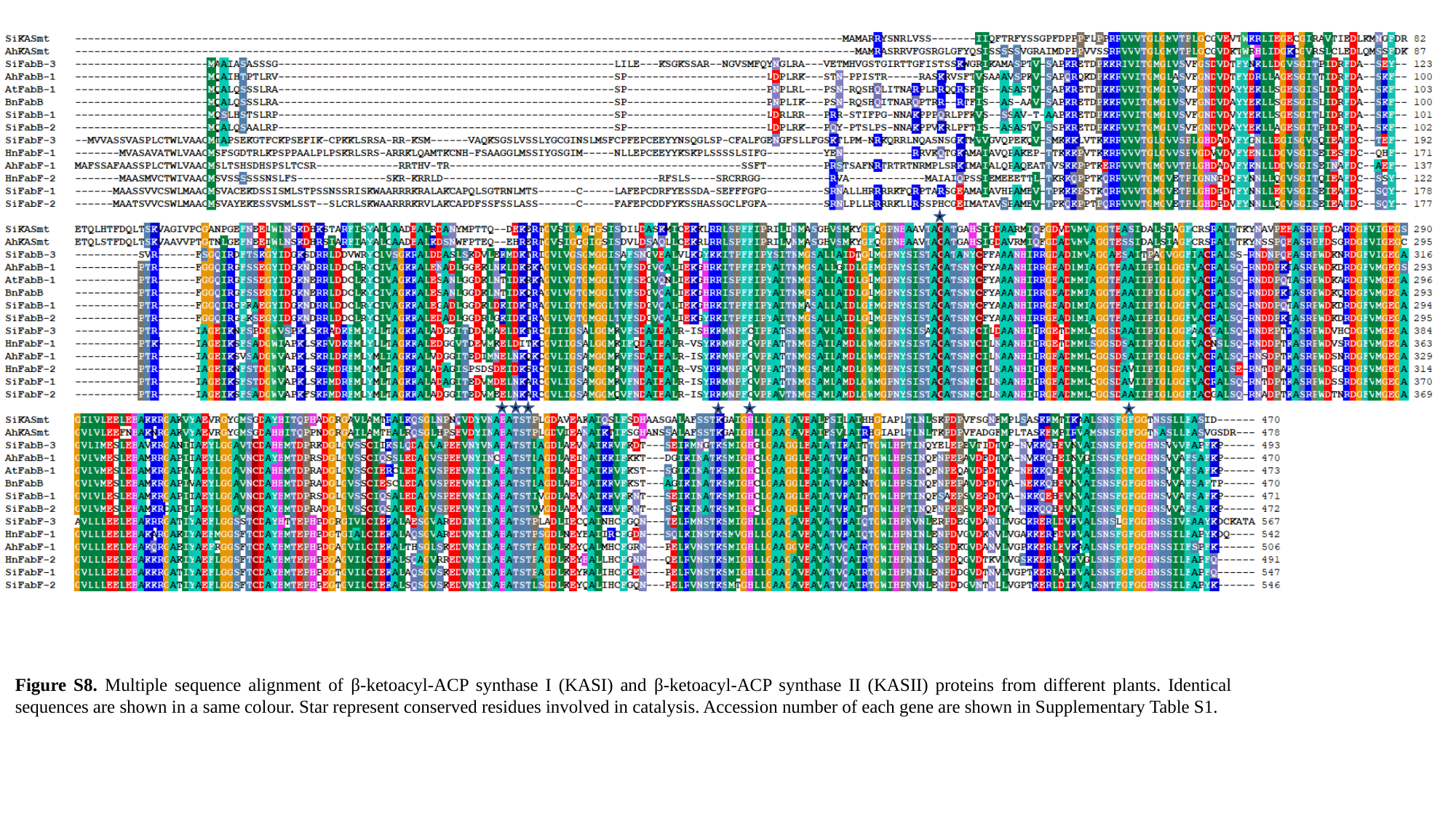

Figure S8. Multiple sequence alignment of β-ketoacyl-ACP synthase I (KASI) and β-ketoacyl-ACP synthase II (KASII) proteins from different plants. Identical sequences are shown in a same colour. Star represent conserved residues involved in catalysis. Accession number of each gene are shown in Supplementary Table S1.

## Slide 9
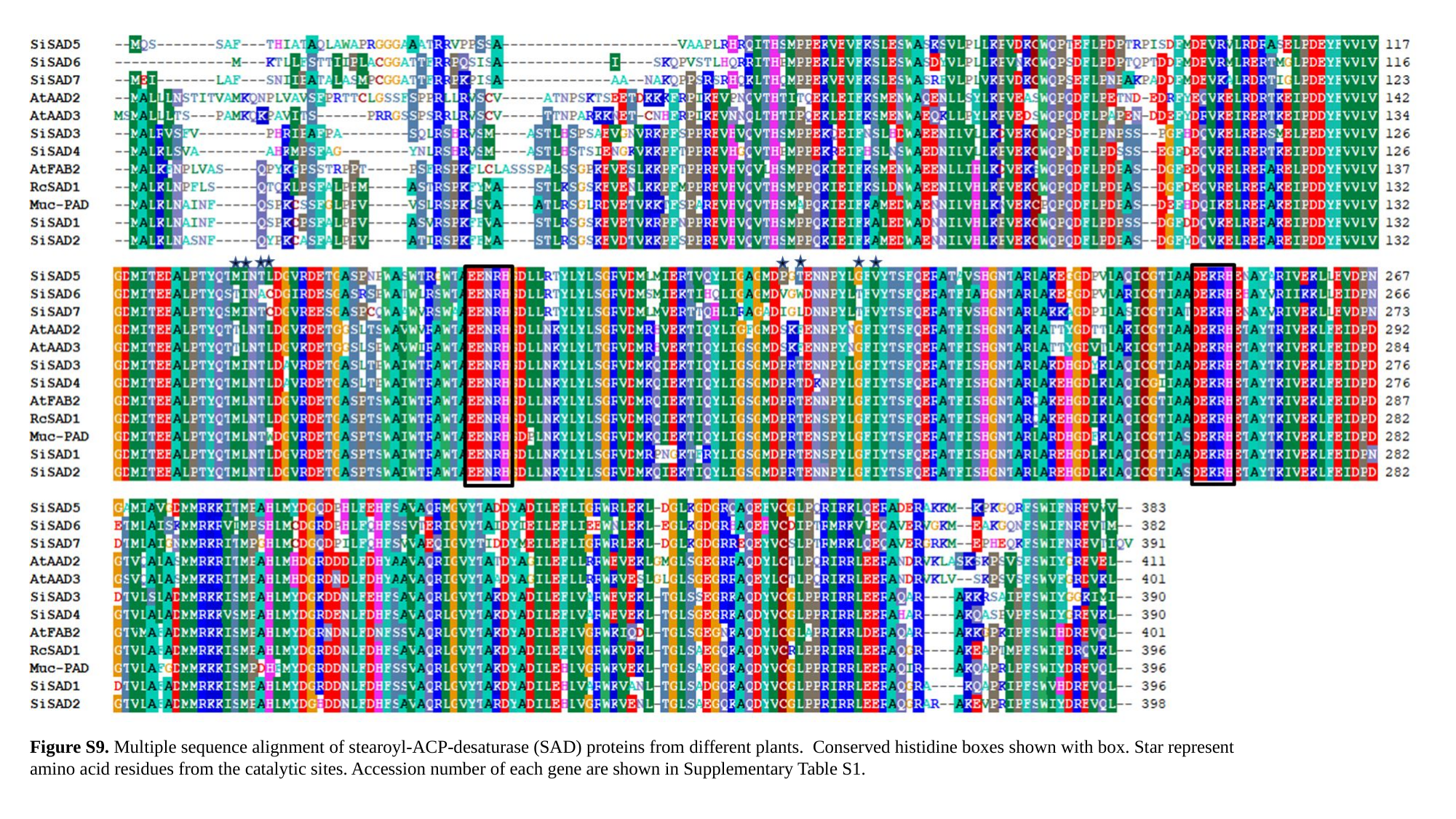

Figure S9. Multiple sequence alignment of stearoyl-ACP-desaturase (SAD) proteins from different plants. Conserved histidine boxes shown with box. Star represent amino acid residues from the catalytic sites. Accession number of each gene are shown in Supplementary Table S1.

## Slide 10
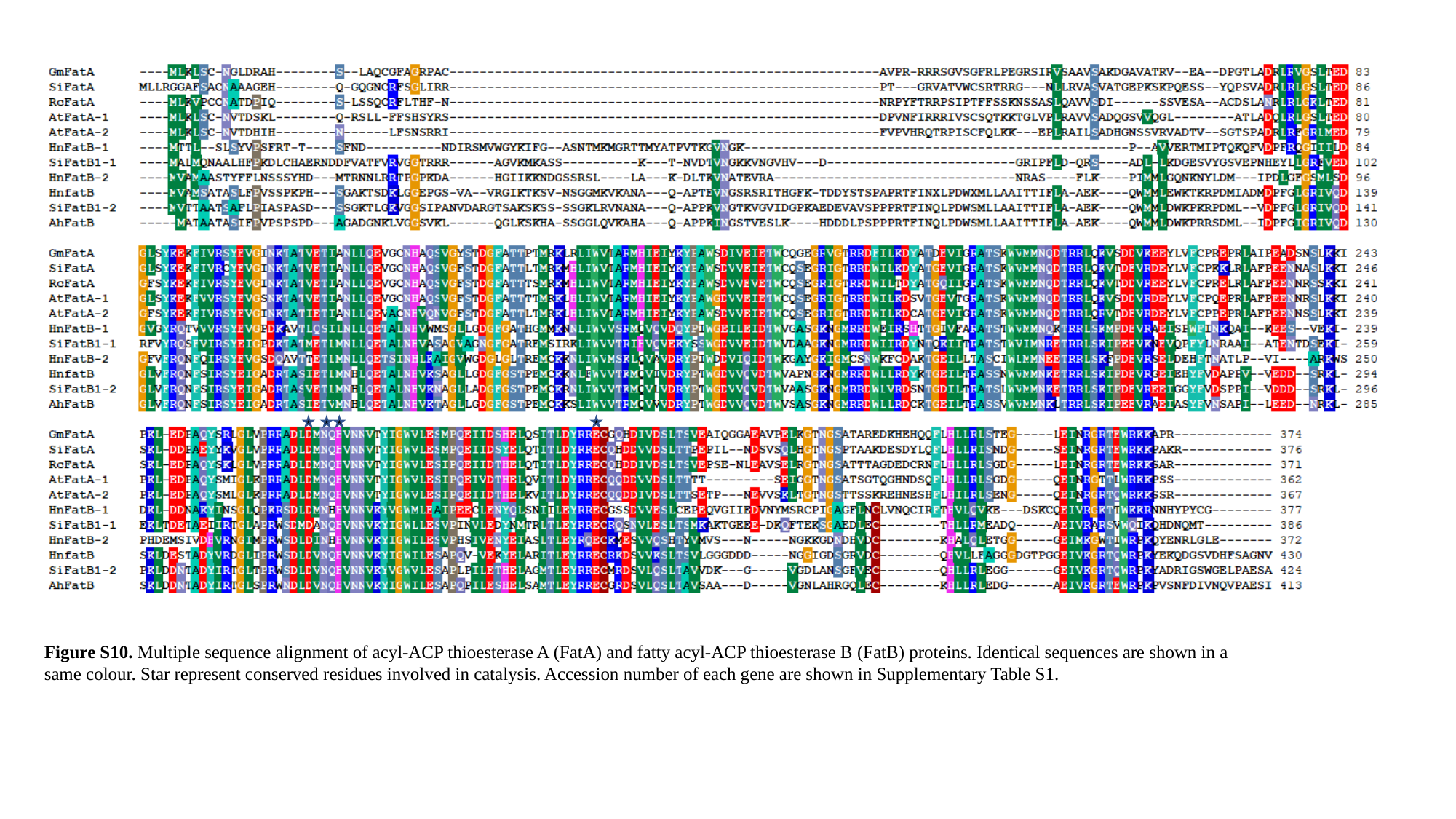

Figure S10. Multiple sequence alignment of acyl-ACP thioesterase A (FatA) and fatty acyl-ACP thioesterase B (FatB) proteins. Identical sequences are shown in a same colour. Star represent conserved residues involved in catalysis. Accession number of each gene are shown in Supplementary Table S1.

## Slide 11
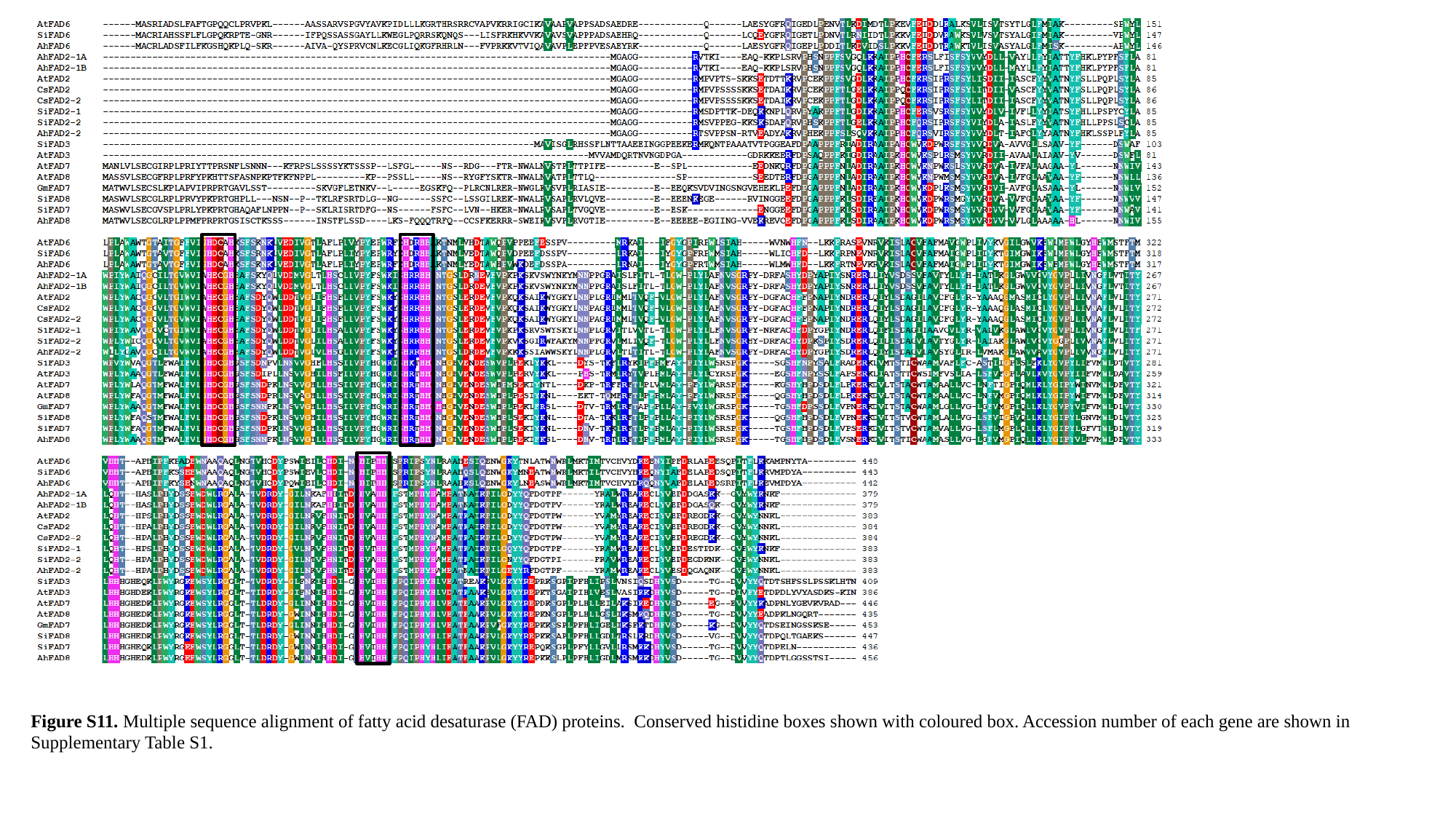

Figure S11. Multiple sequence alignment of fatty acid desaturase (FAD) proteins. Conserved histidine boxes shown with coloured box. Accession number of each gene are shown in Supplementary Table S1.

## Slide 12
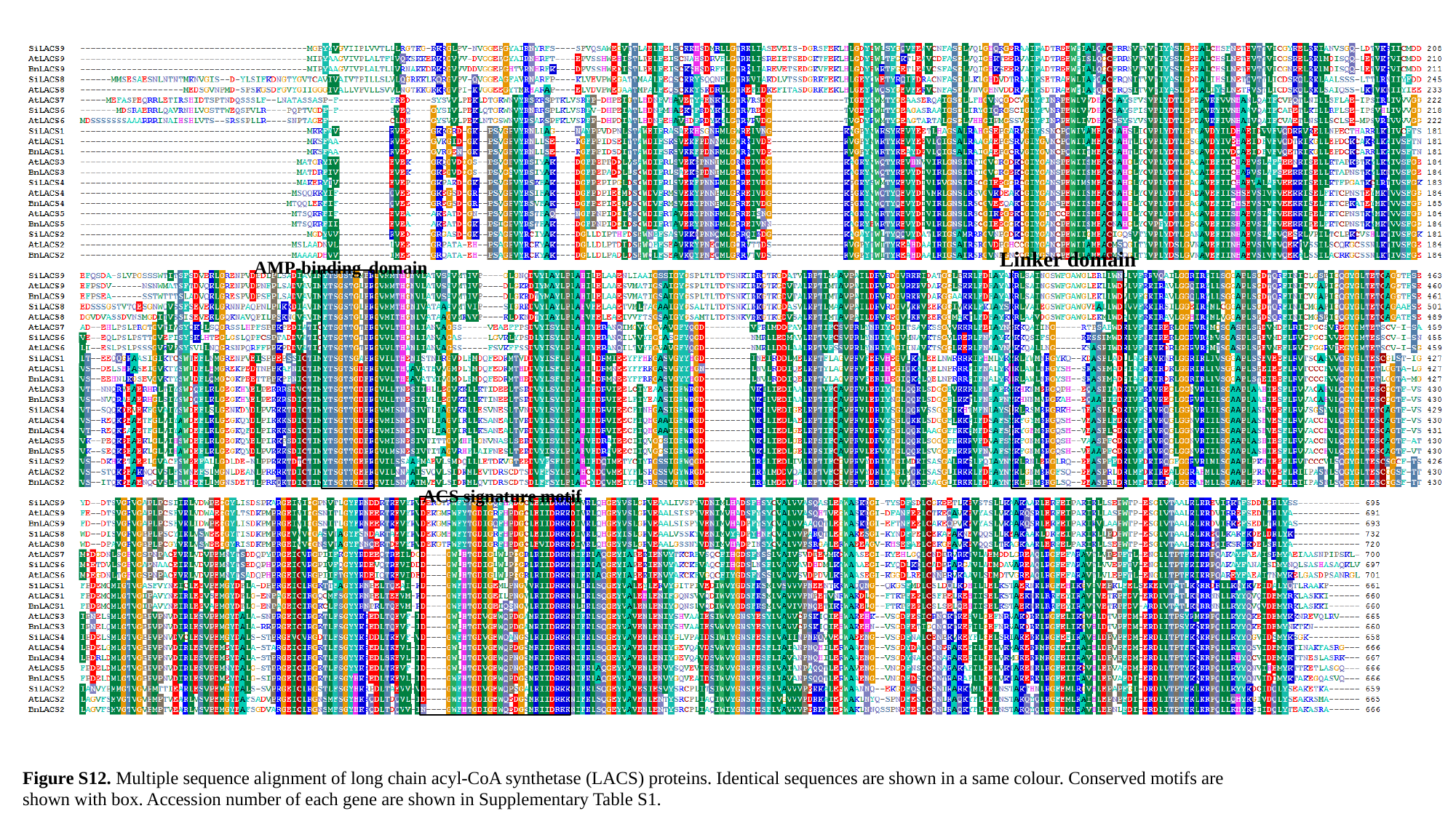

#
AMP-binding domain
Linker domain
ACS signature motif
Figure S12. Multiple sequence alignment of long chain acyl-CoA synthetase (LACS) proteins. Identical sequences are shown in a same colour. Conserved motifs are shown with box. Accession number of each gene are shown in Supplementary Table S1.

## Slide 13
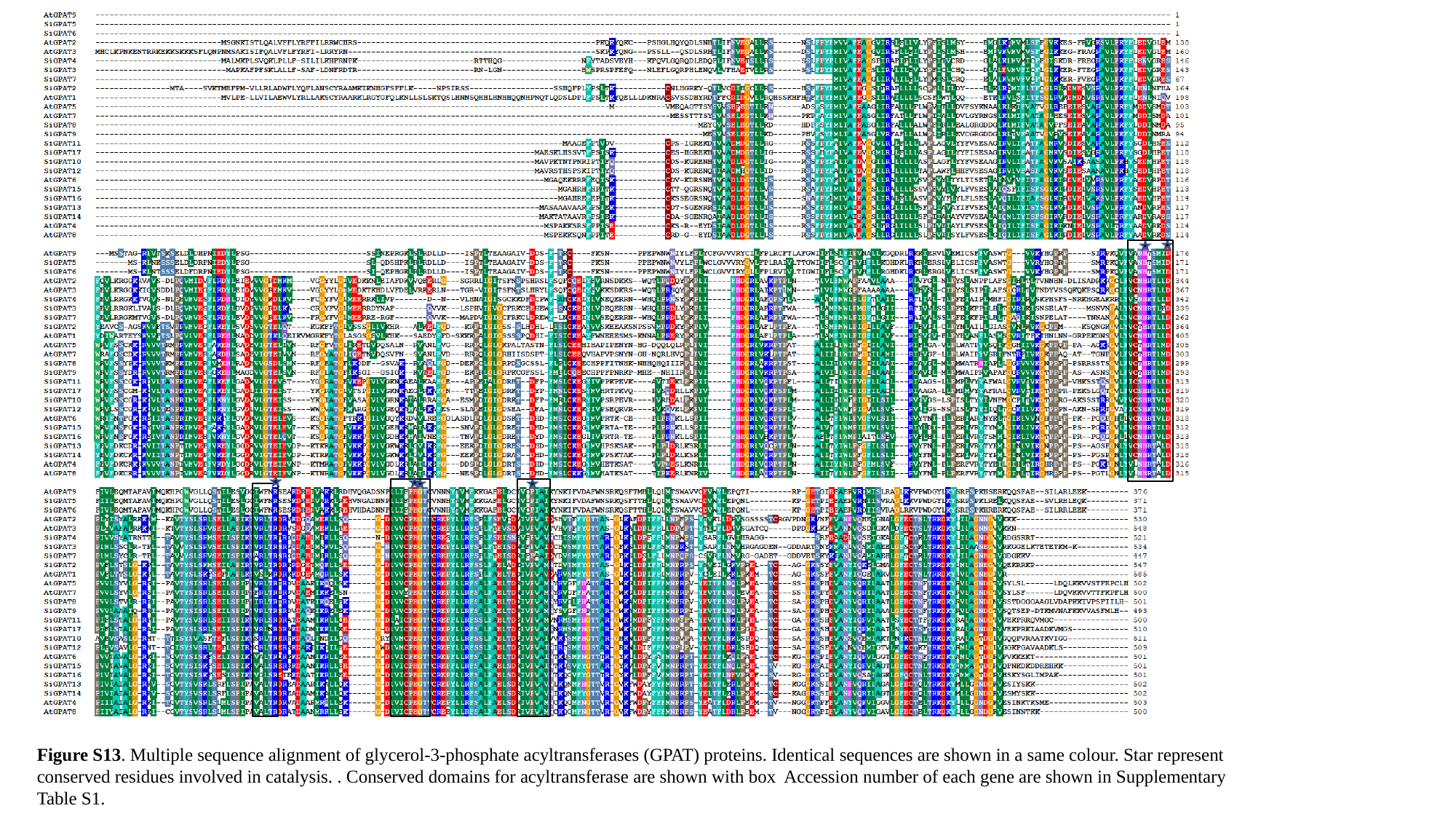

Figure S13. Multiple sequence alignment of glycerol-3-phosphate acyltransferases (GPAT) proteins. Identical sequences are shown in a same colour. Star represent conserved residues involved in catalysis. . Conserved domains for acyltransferase are shown with box Accession number of each gene are shown in Supplementary Table S1.

## Slide 14
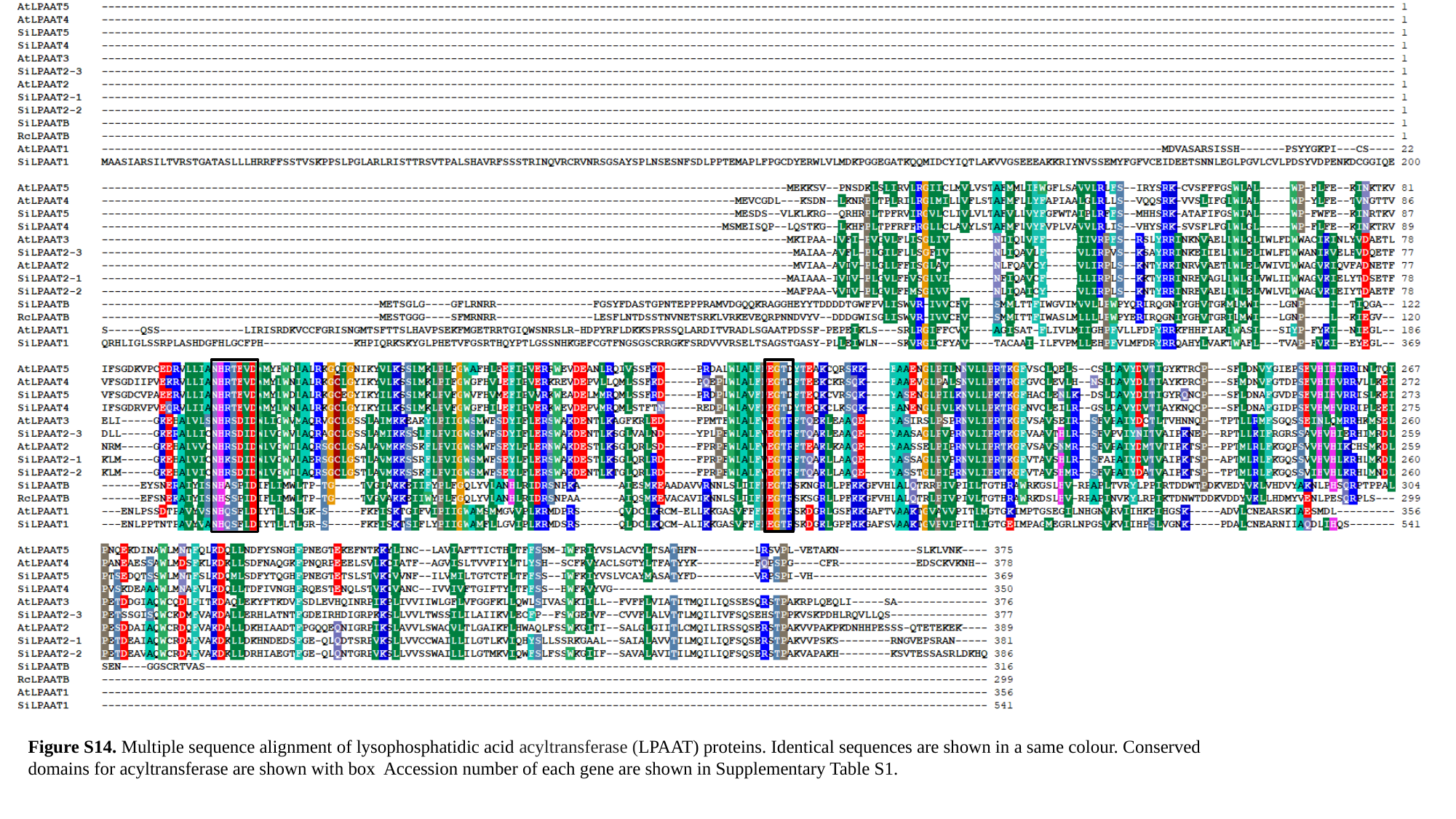

Figure S14. Multiple sequence alignment of lysophosphatidic acid acyltransferase (LPAAT) proteins. Identical sequences are shown in a same colour. Conserved domains for acyltransferase are shown with box Accession number of each gene are shown in Supplementary Table S1.

## Slide 15
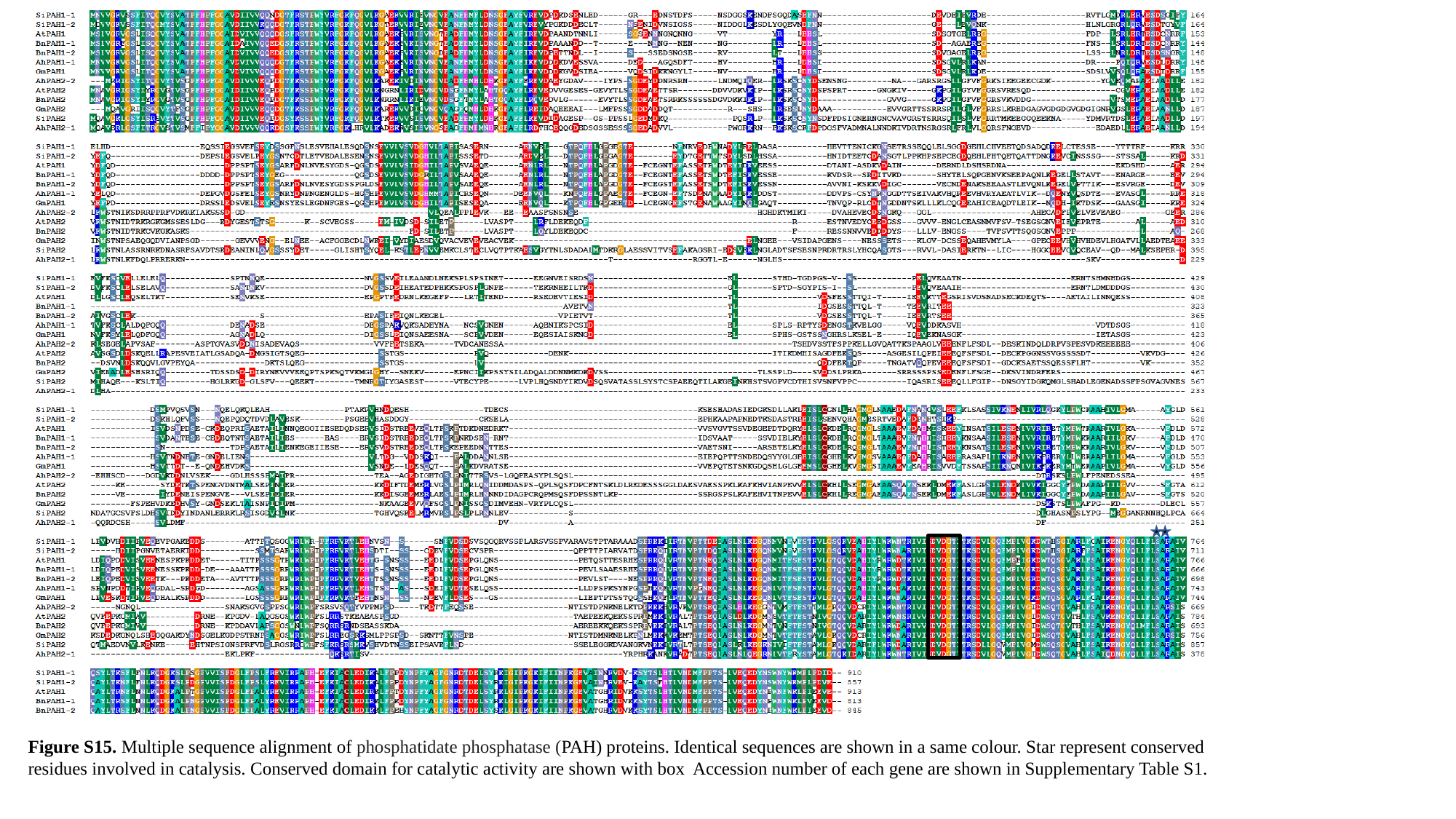

Figure S15. Multiple sequence alignment of phosphatidate phosphatase (PAH) proteins. Identical sequences are shown in a same colour. Star represent conserved residues involved in catalysis. Conserved domain for catalytic activity are shown with box Accession number of each gene are shown in Supplementary Table S1.

## Slide 16
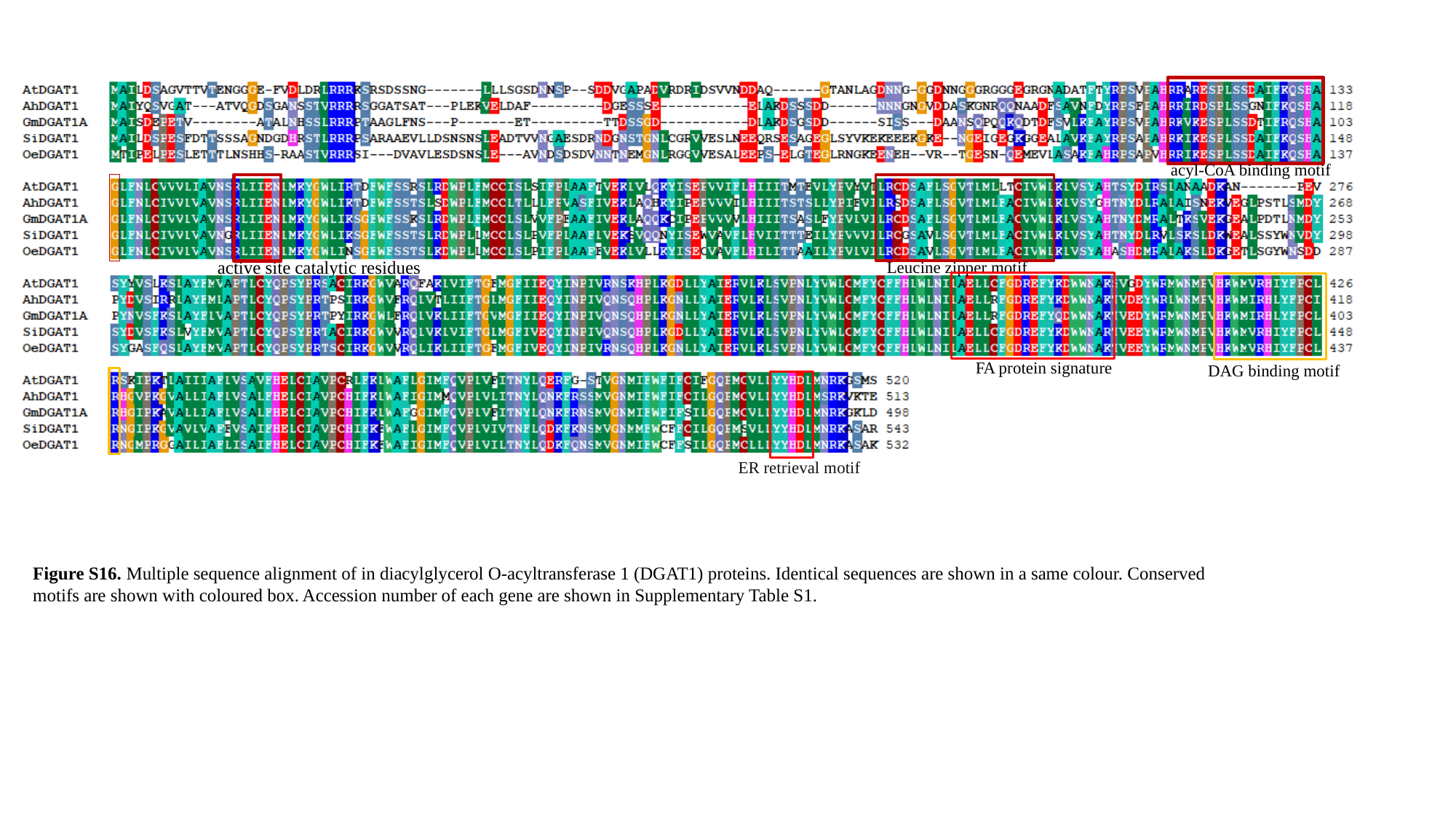

acyl-CoA binding motif
active site catalytic residues
Leucine zipper motif
FA protein signature
DAG binding motif
ER retrieval motif
Figure S16. Multiple sequence alignment of in diacylglycerol O-acyltransferase 1 (DGAT1) proteins. Identical sequences are shown in a same colour. Conserved motifs are shown with coloured box. Accession number of each gene are shown in Supplementary Table S1.

## Slide 17
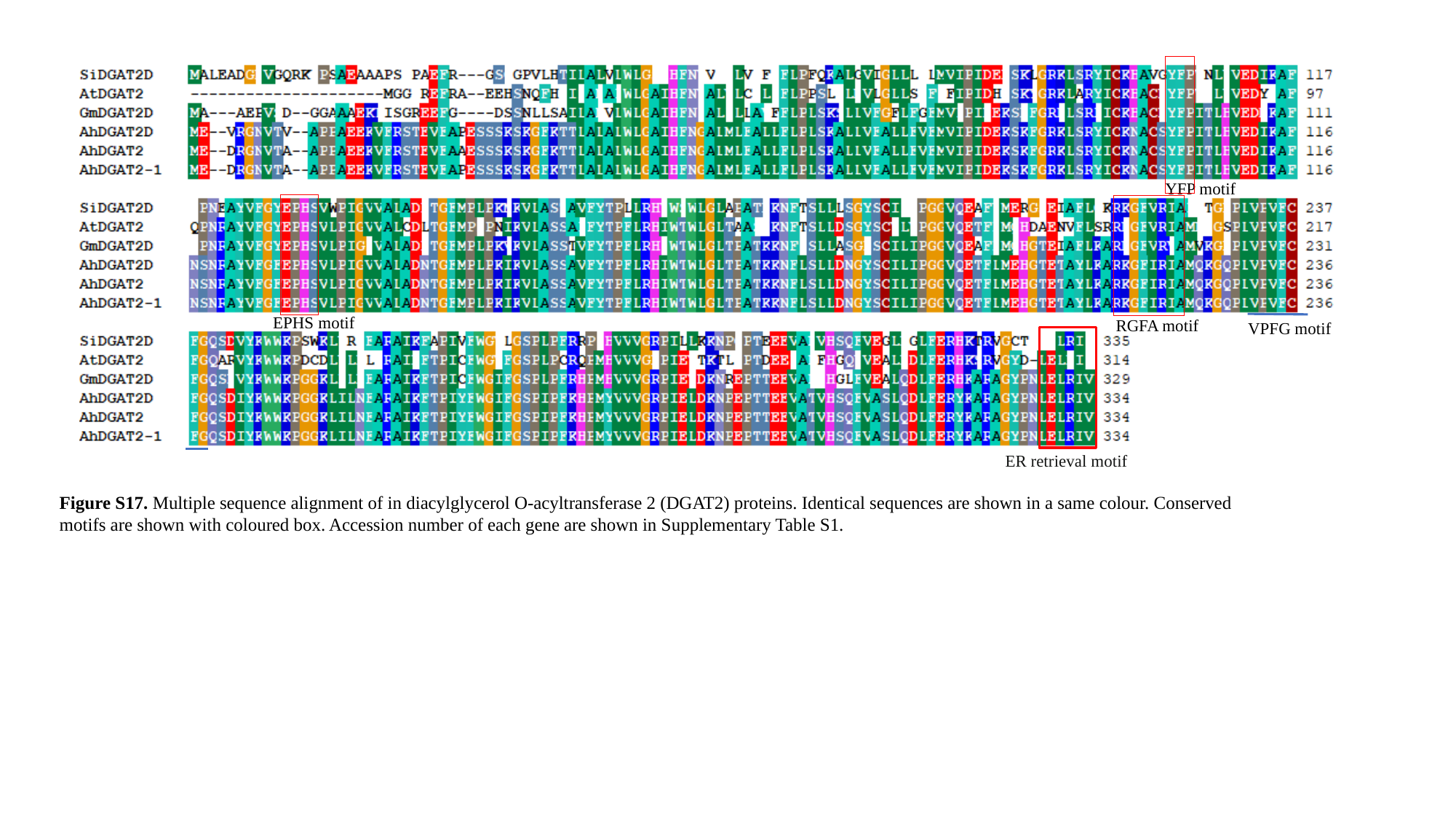

YFP motif
EPHS motif
RGFA motif
VPFG motif
ER retrieval motif
Figure S17. Multiple sequence alignment of in diacylglycerol O-acyltransferase 2 (DGAT2) proteins. Identical sequences are shown in a same colour. Conserved motifs are shown with coloured box. Accession number of each gene are shown in Supplementary Table S1.

## Slide 18
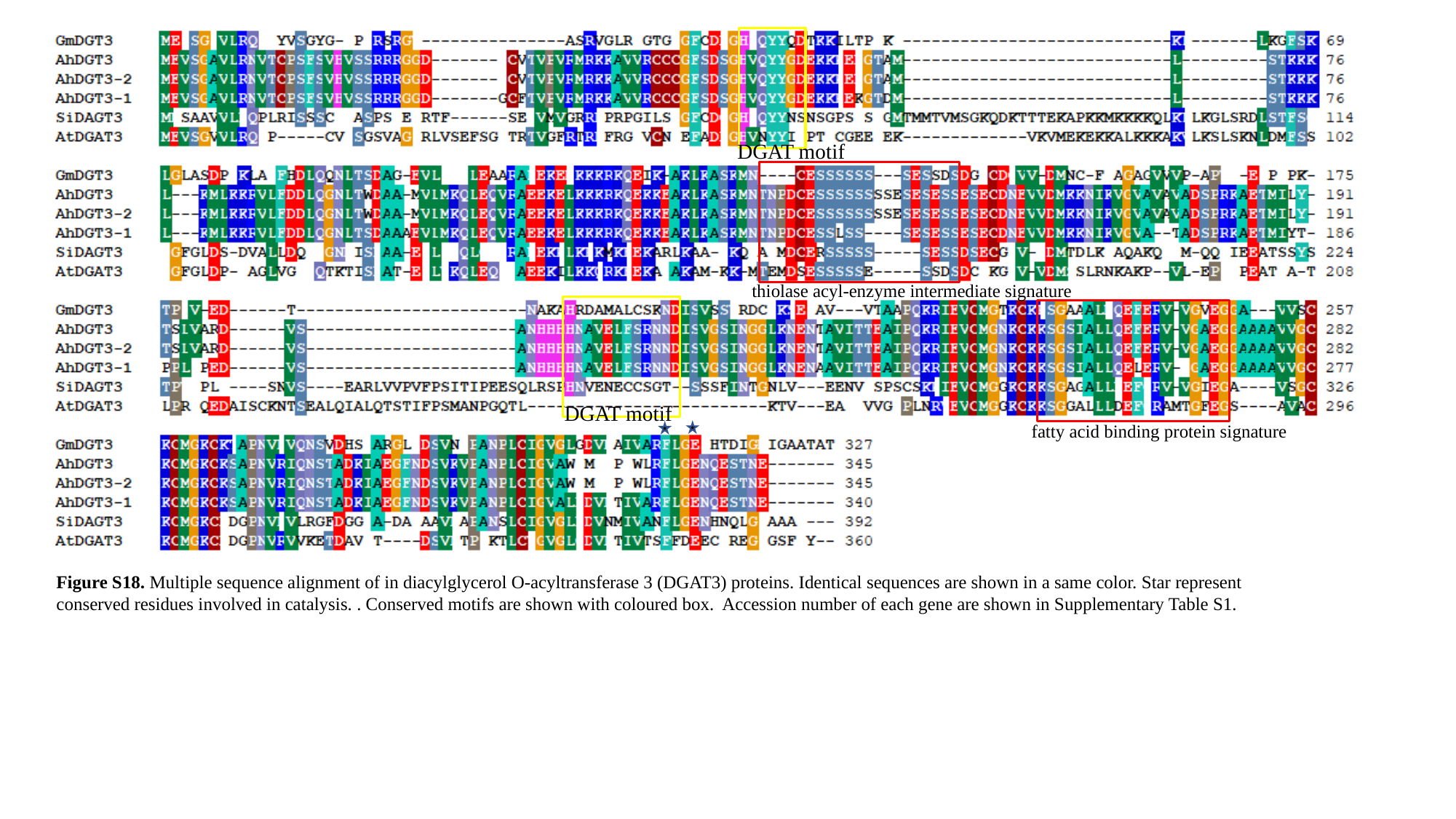

DGAT motif
thiolase acyl-enzyme intermediate signature
DGAT motif
fatty acid binding protein signature
Figure S18. Multiple sequence alignment of in diacylglycerol O-acyltransferase 3 (DGAT3) proteins. Identical sequences are shown in a same color. Star represent conserved residues involved in catalysis. . Conserved motifs are shown with coloured box. Accession number of each gene are shown in Supplementary Table S1.

## Slide 19
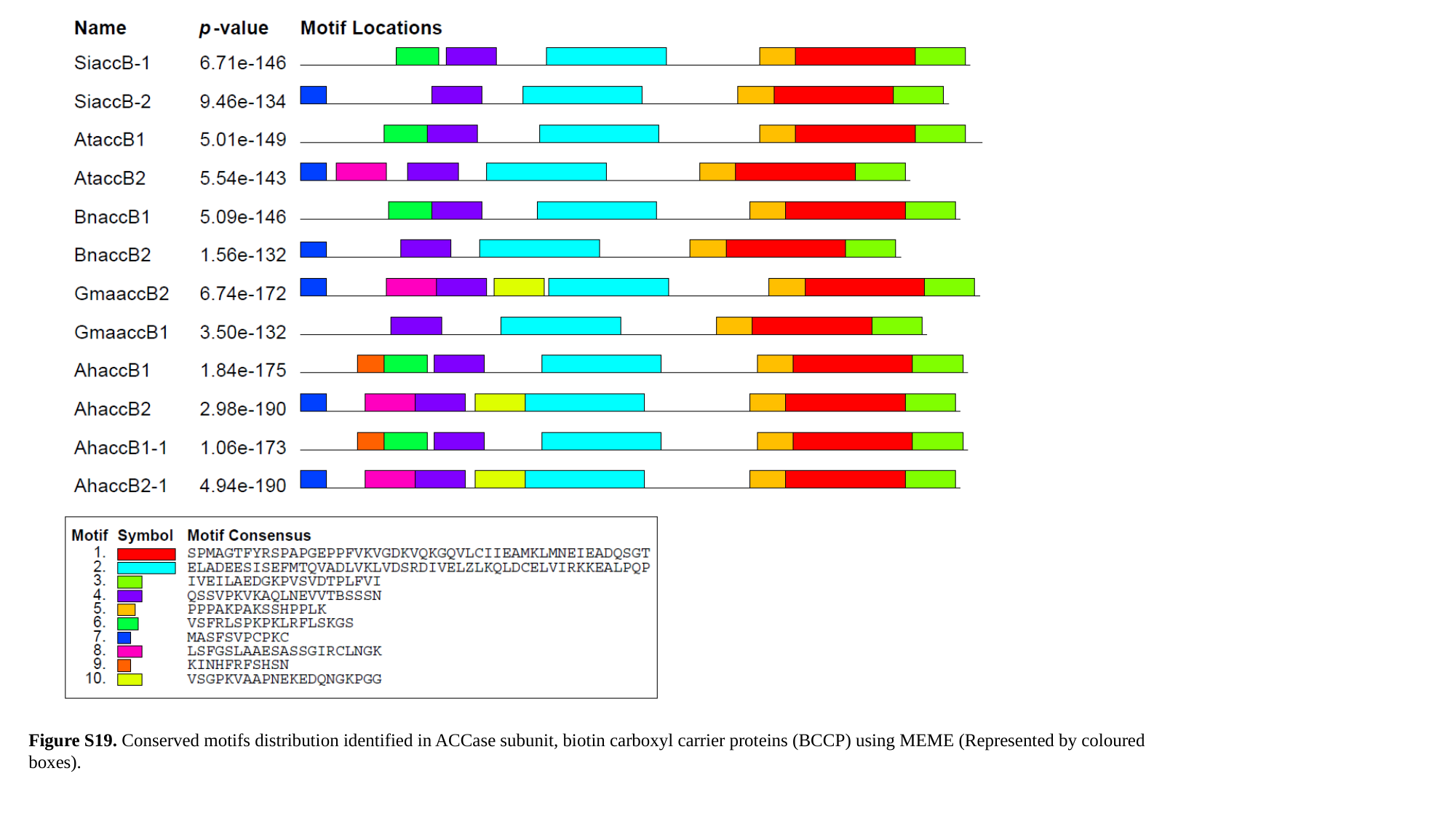

Figure S19. Conserved motifs distribution identified in ACCase subunit, biotin carboxyl carrier proteins (BCCP) using MEME (Represented by coloured boxes).

## Slide 20
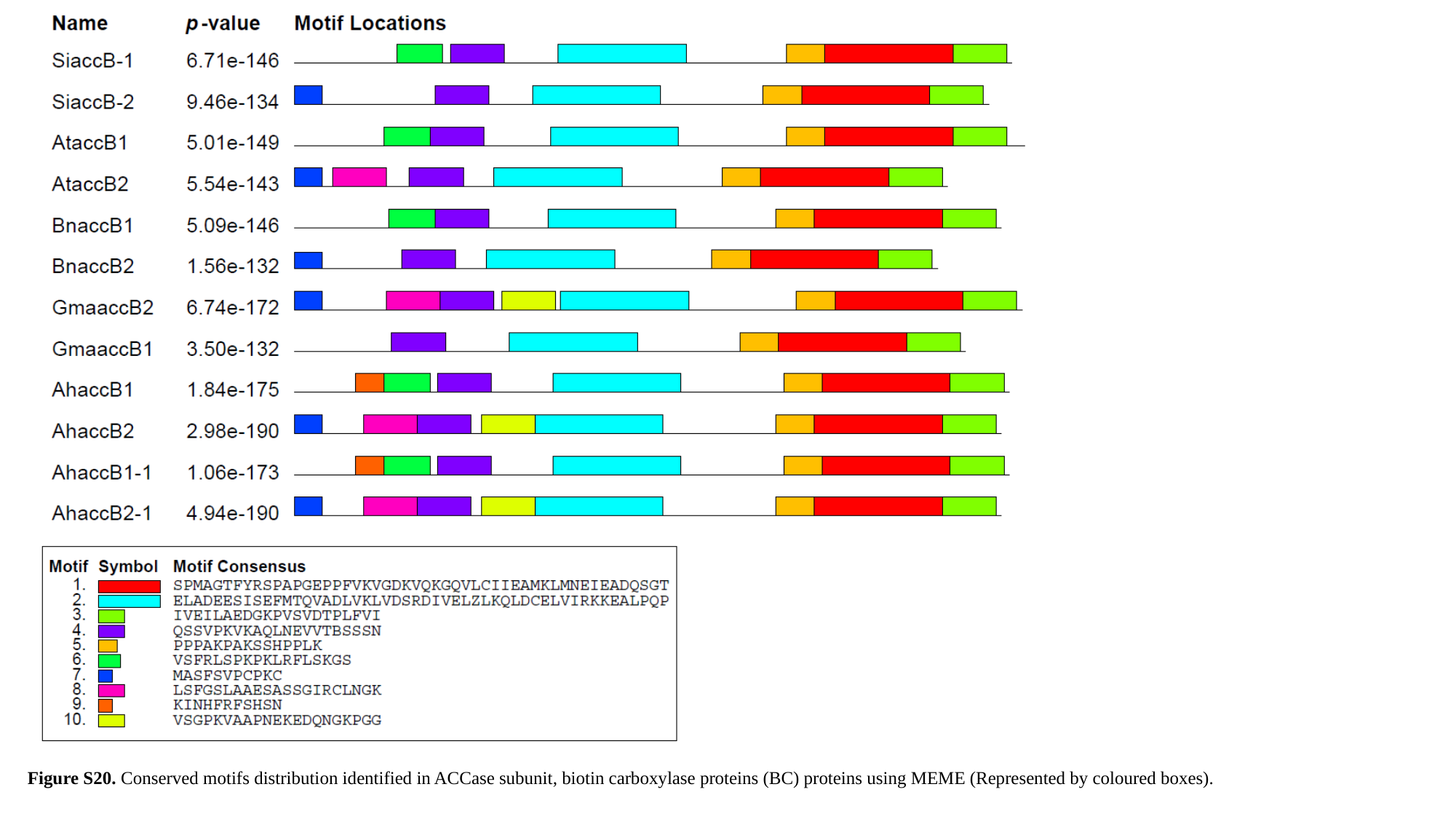

Figure S20. Conserved motifs distribution identified in ACCase subunit, biotin carboxylase proteins (BC) proteins using MEME (Represented by coloured boxes).

## Slide 21
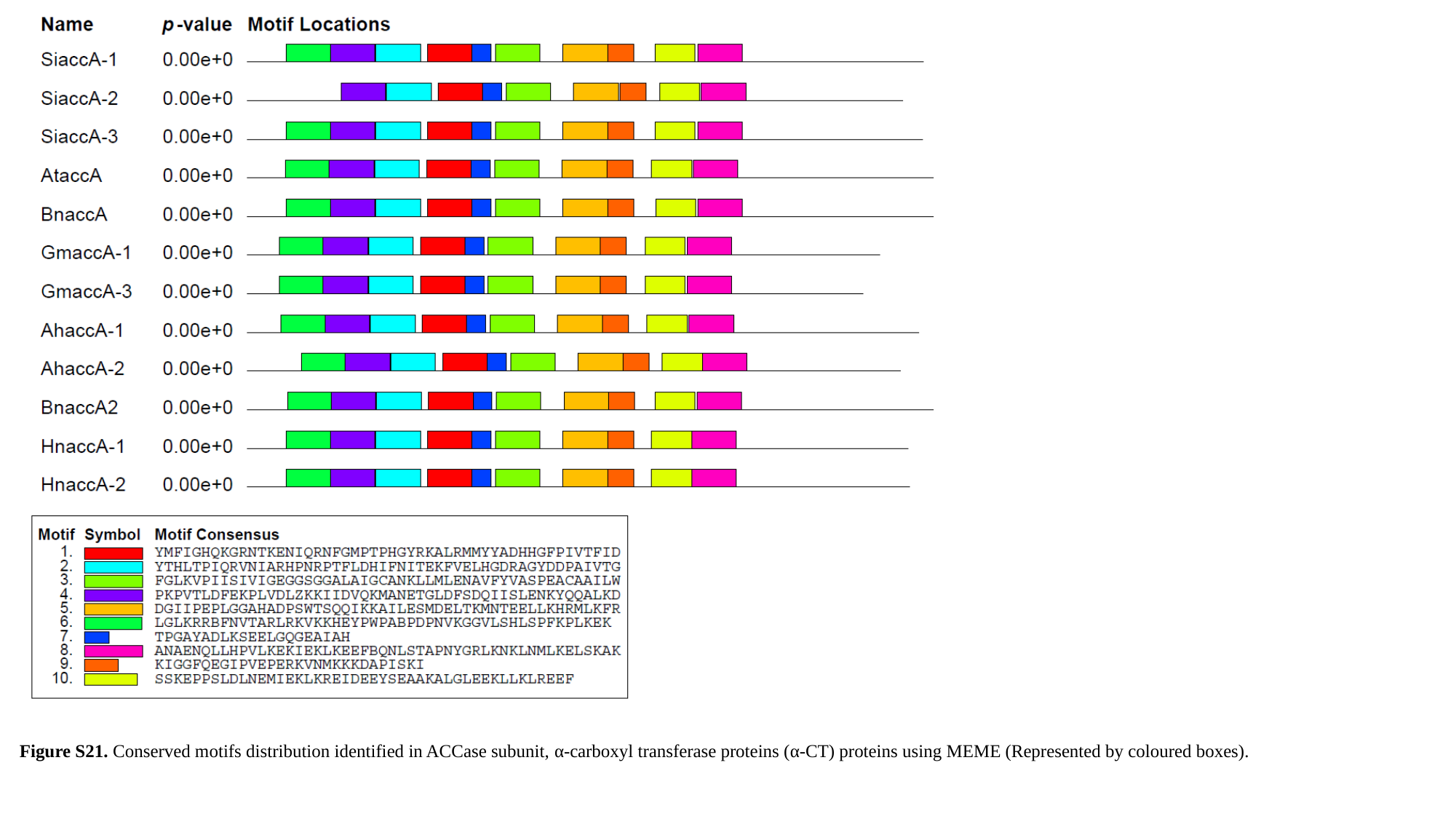

Figure S21. Conserved motifs distribution identified in ACCase subunit, α-carboxyl transferase proteins (α-CT) proteins using MEME (Represented by coloured boxes).

## Slide 22
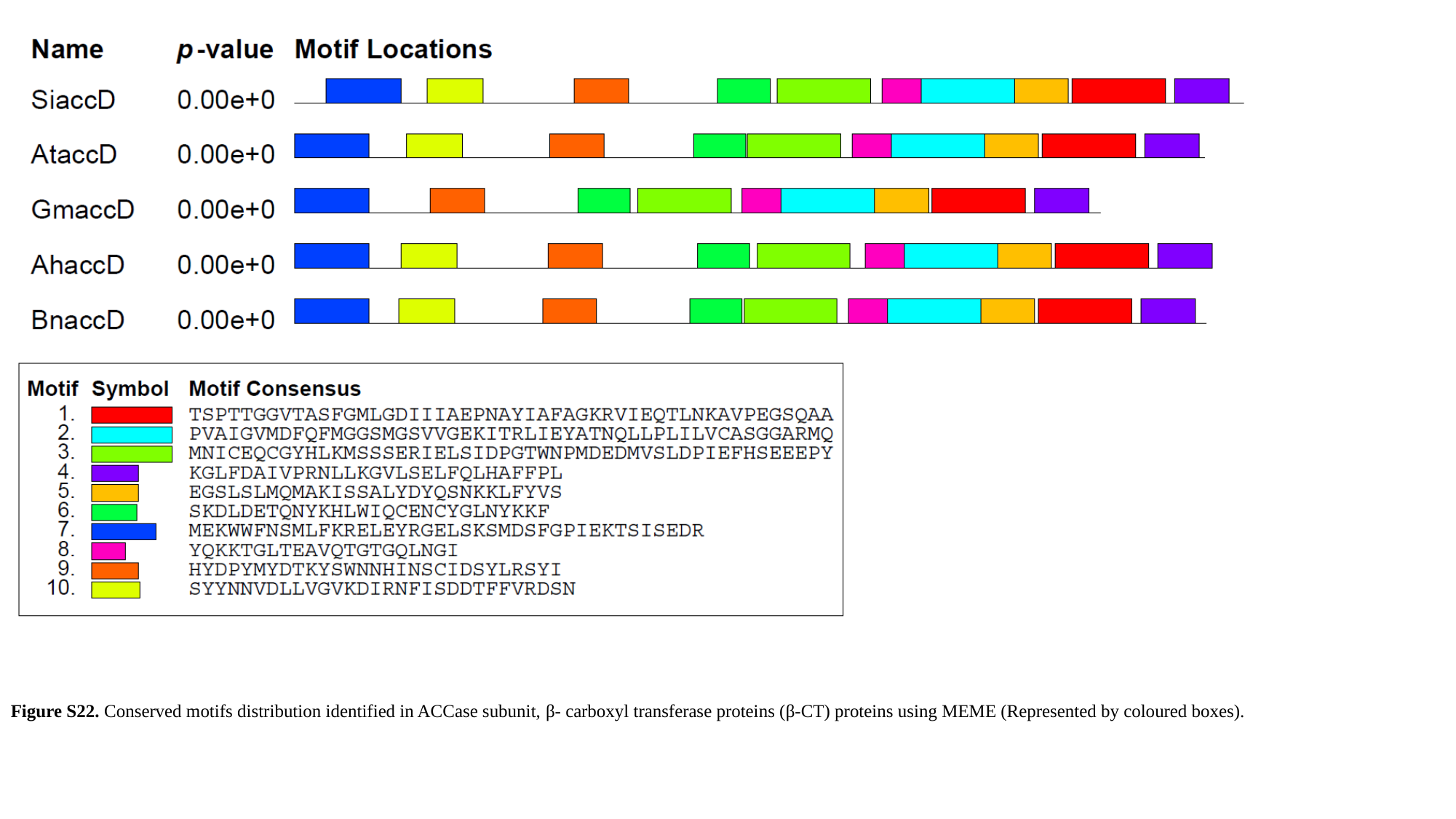

Figure S22. Conserved motifs distribution identified in ACCase subunit, β- carboxyl transferase proteins (β-CT) proteins using MEME (Represented by coloured boxes).

## Slide 23
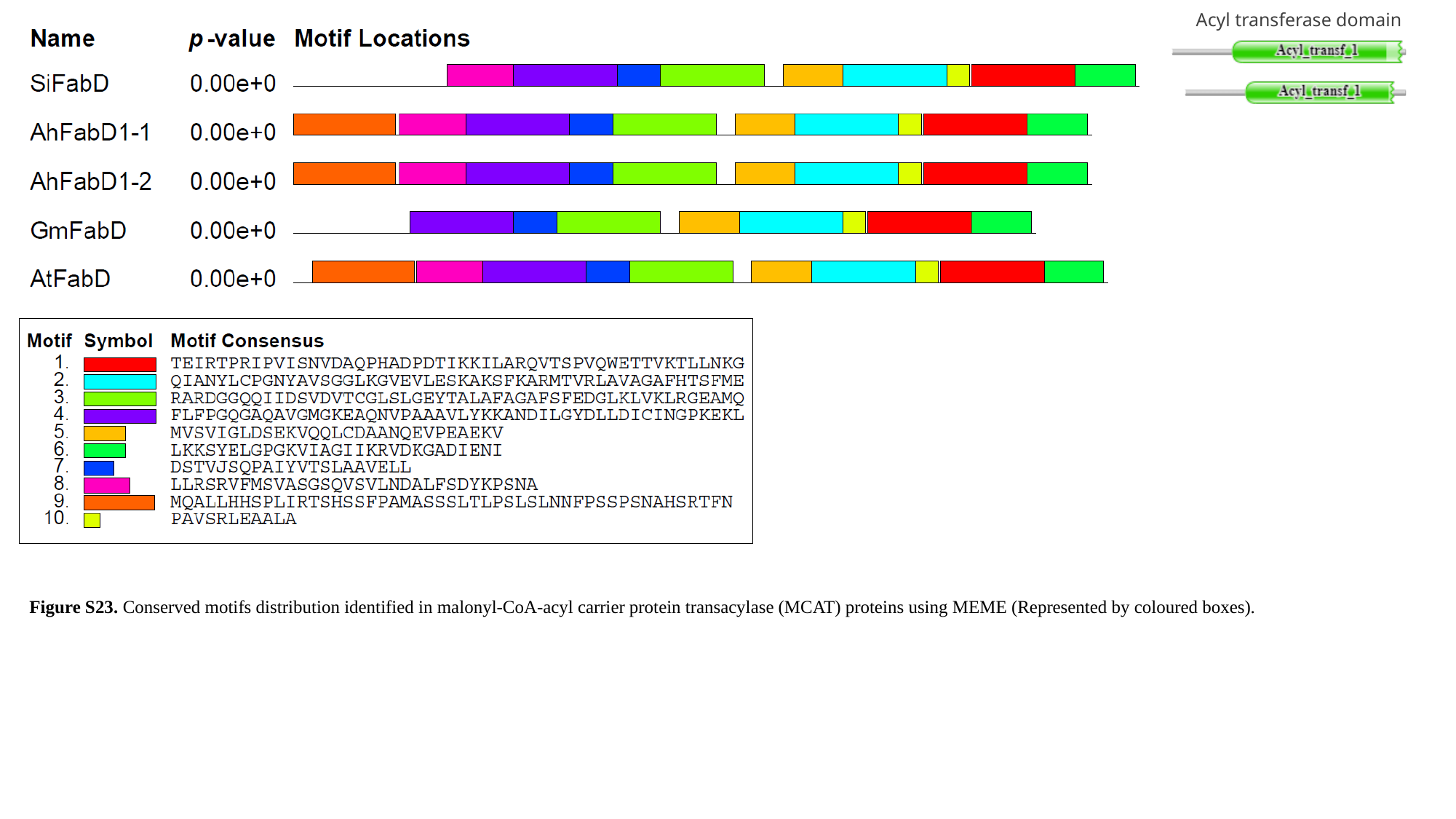

Acyl transferase domain
Figure S23. Conserved motifs distribution identified in malonyl-CoA-acyl carrier protein transacylase (MCAT) proteins using MEME (Represented by coloured boxes).

## Slide 24
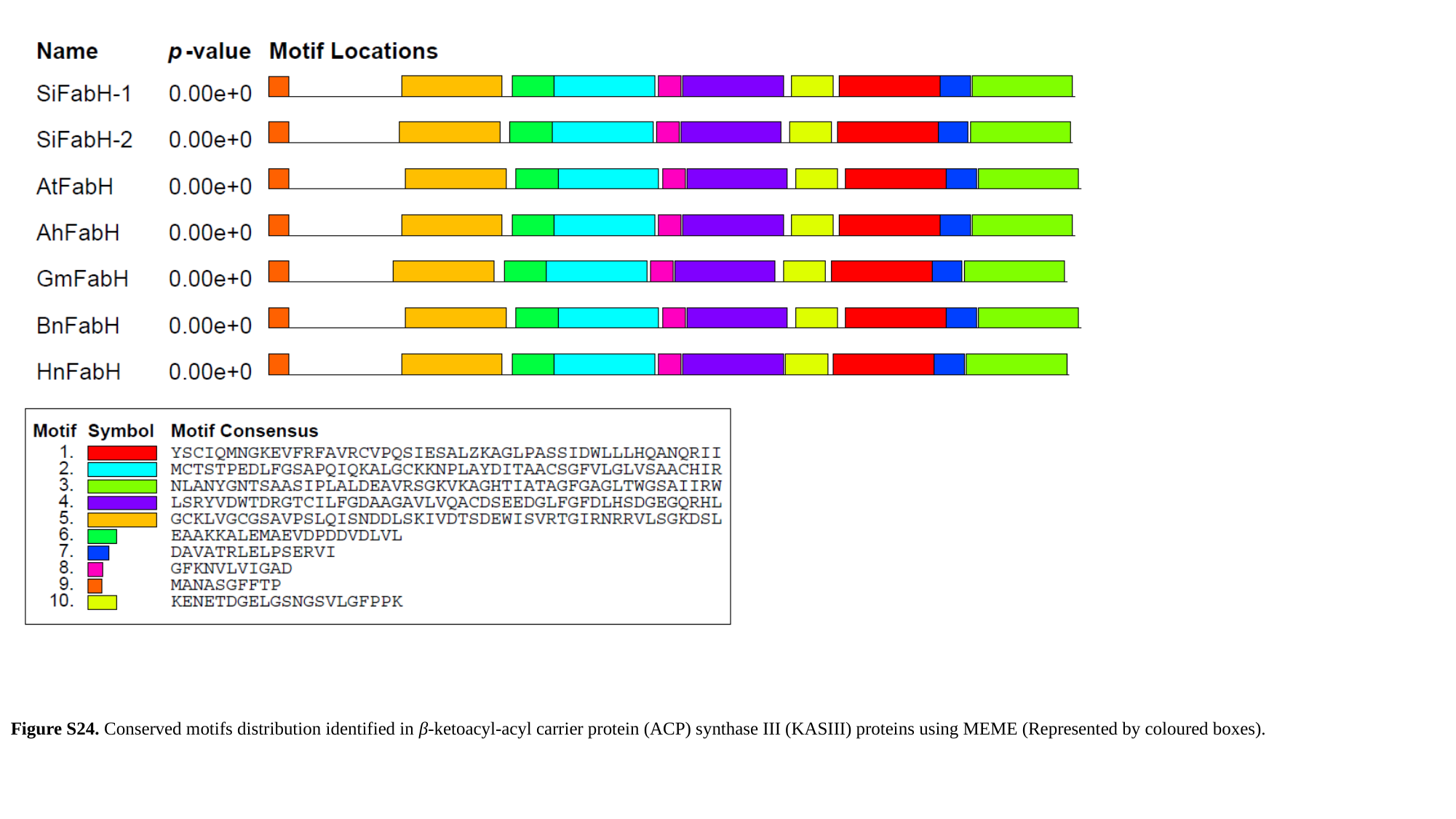

Figure S24. Conserved motifs distribution identified in β-ketoacyl-acyl carrier protein (ACP) synthase III (KASIII) proteins using MEME (Represented by coloured boxes).

## Slide 25
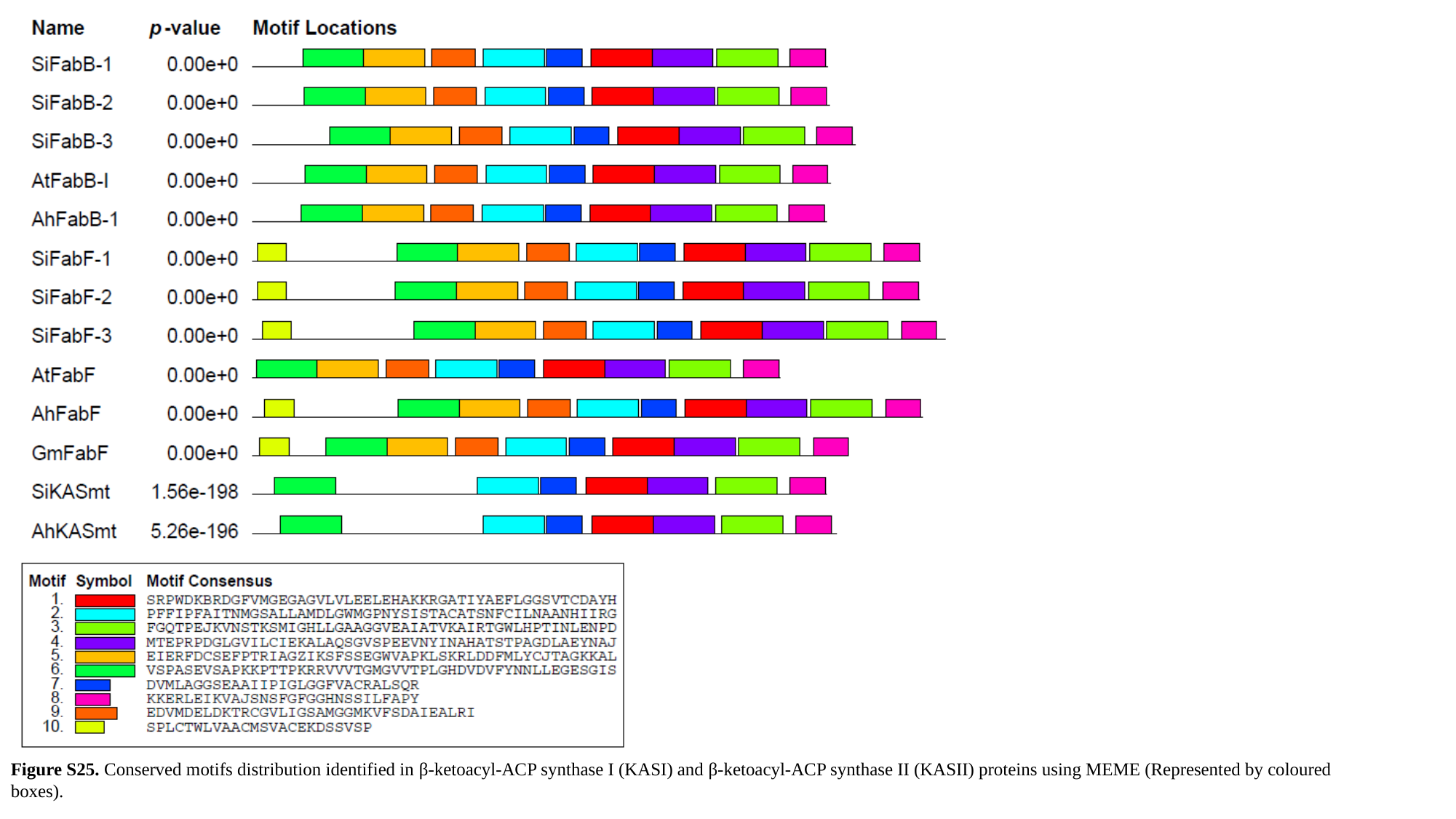

Figure S25. Conserved motifs distribution identified in β-ketoacyl-ACP synthase I (KASI) and β-ketoacyl-ACP synthase II (KASII) proteins using MEME (Represented by coloured boxes).

## Slide 26
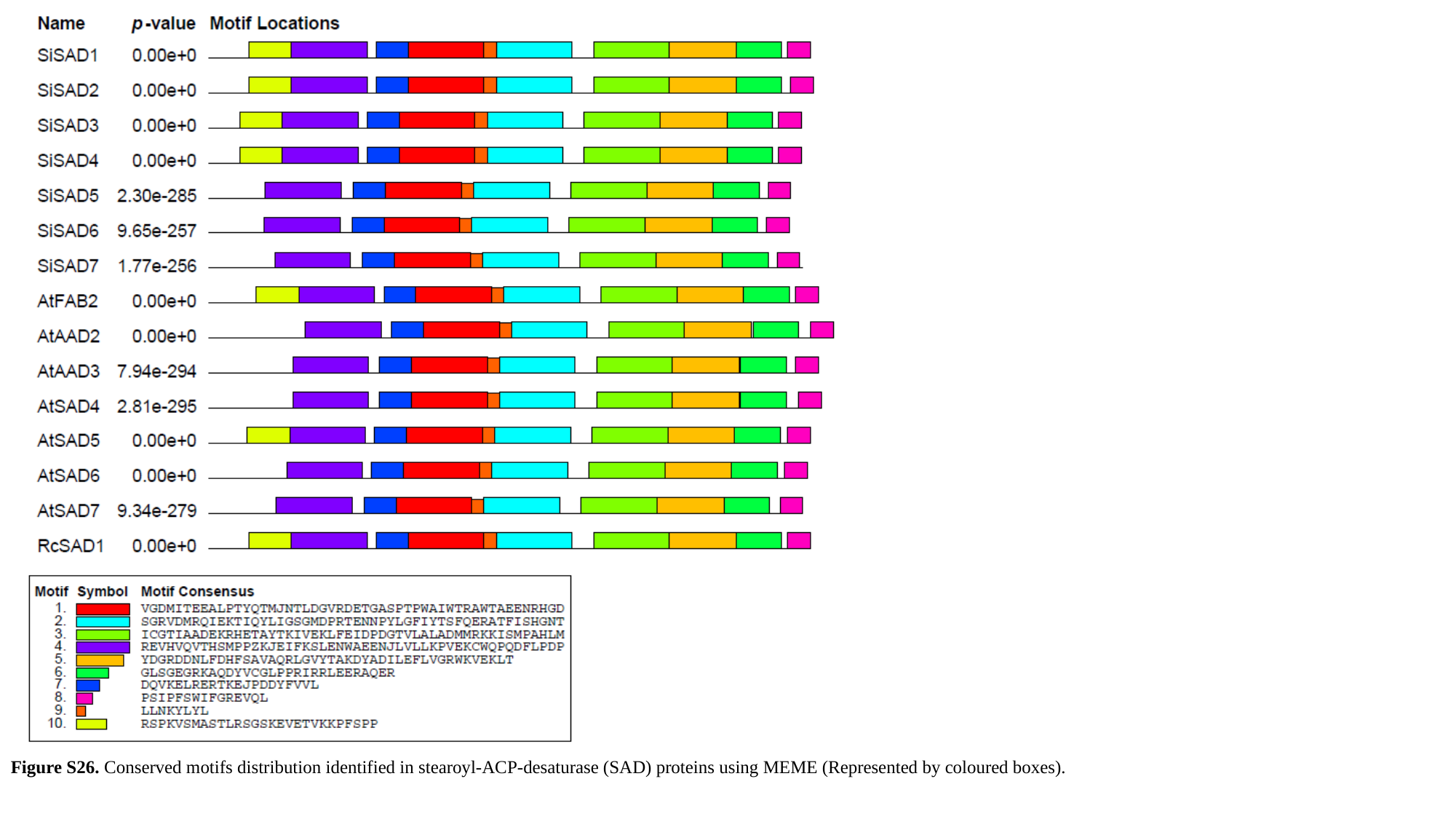

Figure S26. Conserved motifs distribution identified in stearoyl-ACP-desaturase (SAD) proteins using MEME (Represented by coloured boxes).

## Slide 27
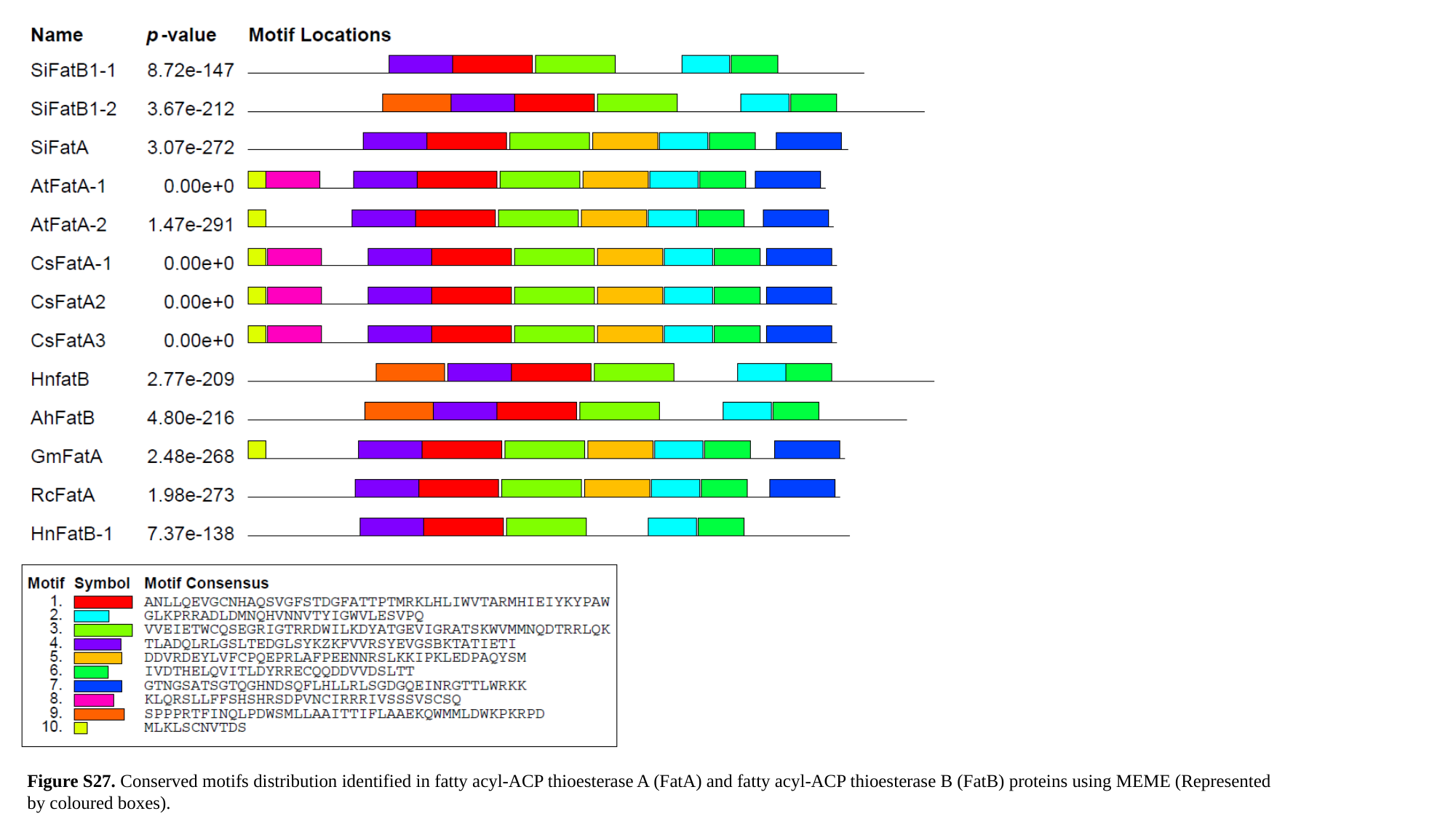

Figure S27. Conserved motifs distribution identified in fatty acyl-ACP thioesterase A (FatA) and fatty acyl-ACP thioesterase B (FatB) proteins using MEME (Represented by coloured boxes).

## Slide 28
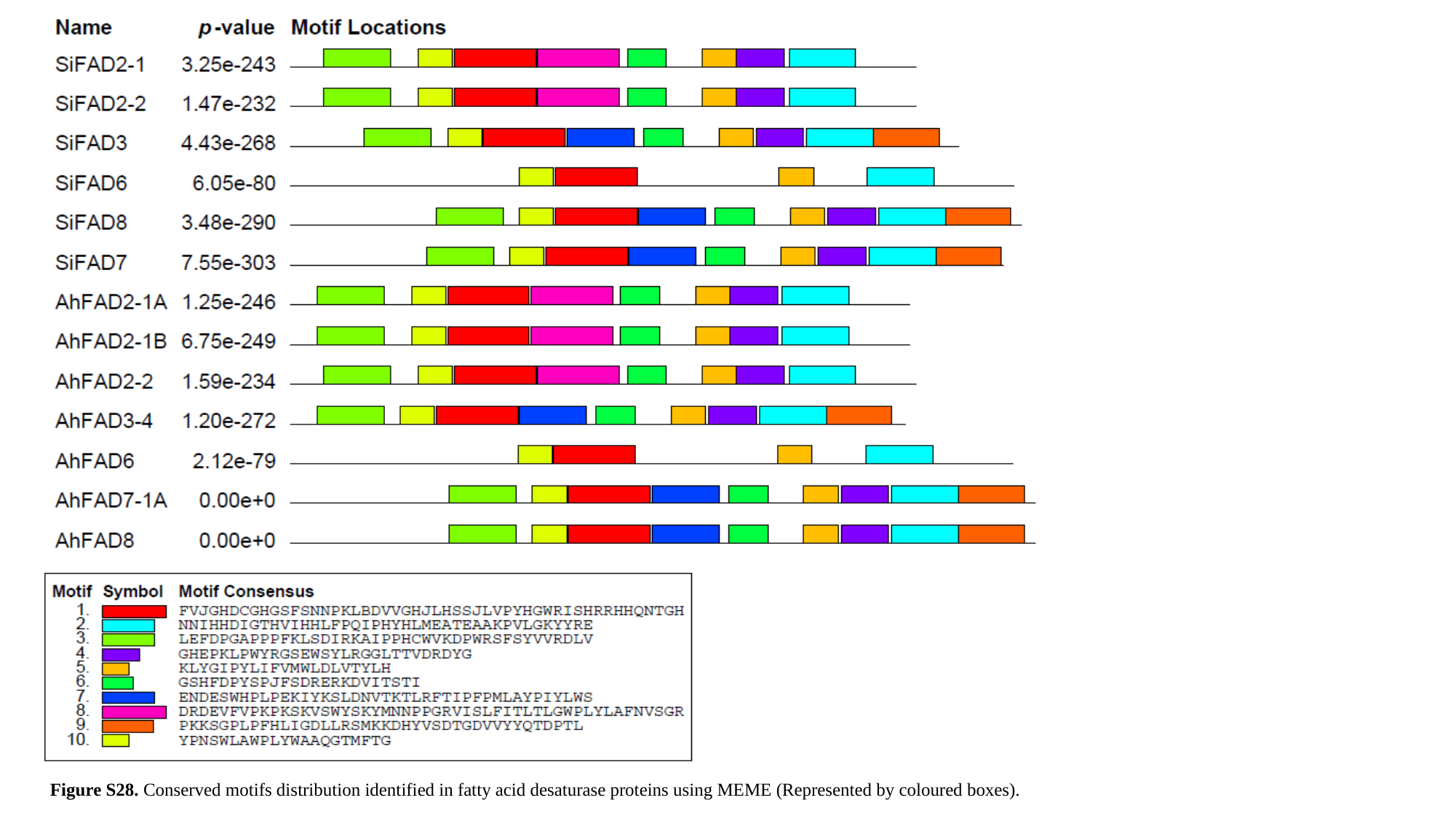

Figure S28. Conserved motifs distribution identified in fatty acid desaturase proteins using MEME (Represented by coloured boxes).

## Slide 29
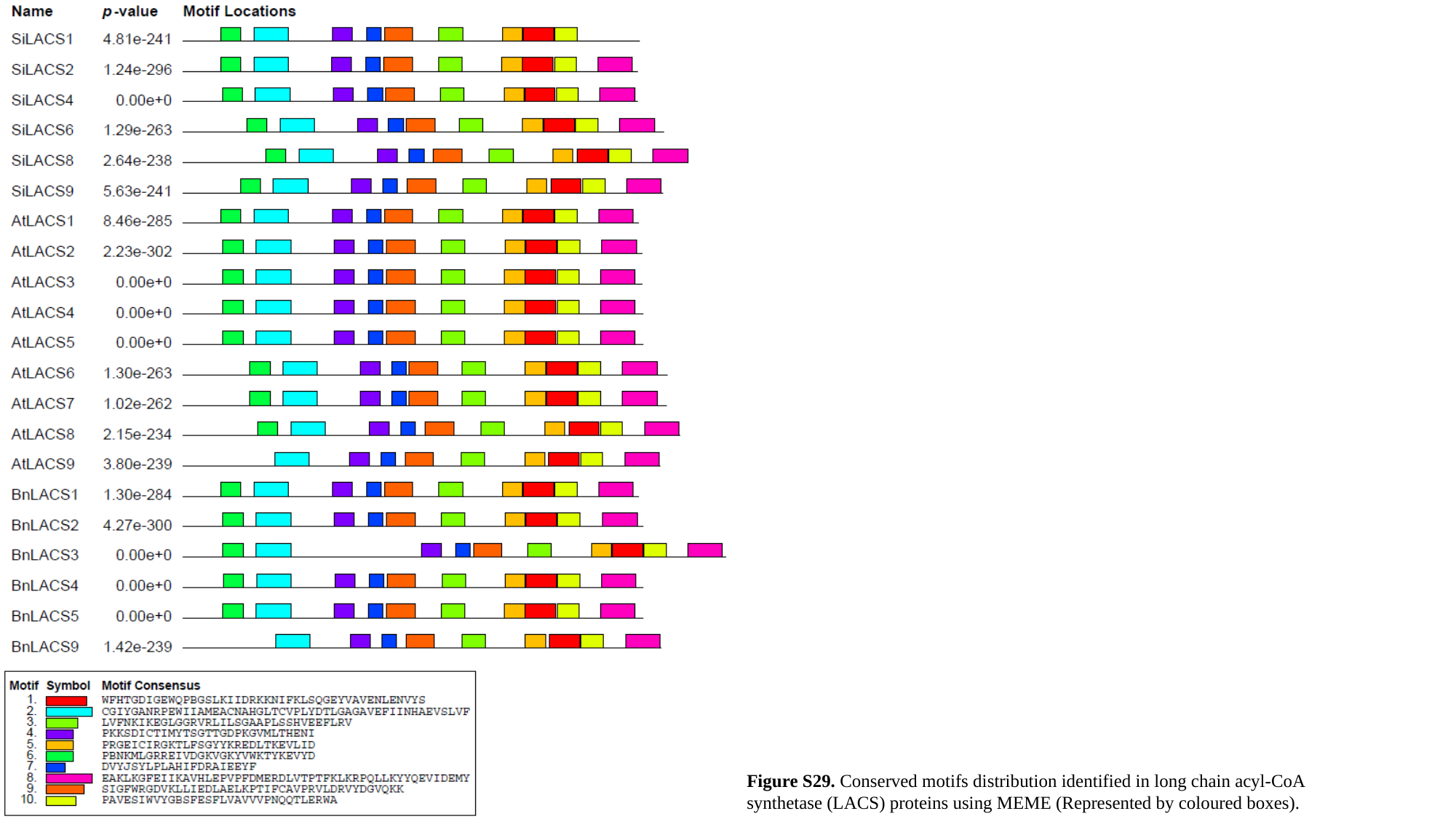

Figure S29. Conserved motifs distribution identified in long chain acyl-CoA synthetase (LACS) proteins using MEME (Represented by coloured boxes).

## Slide 30
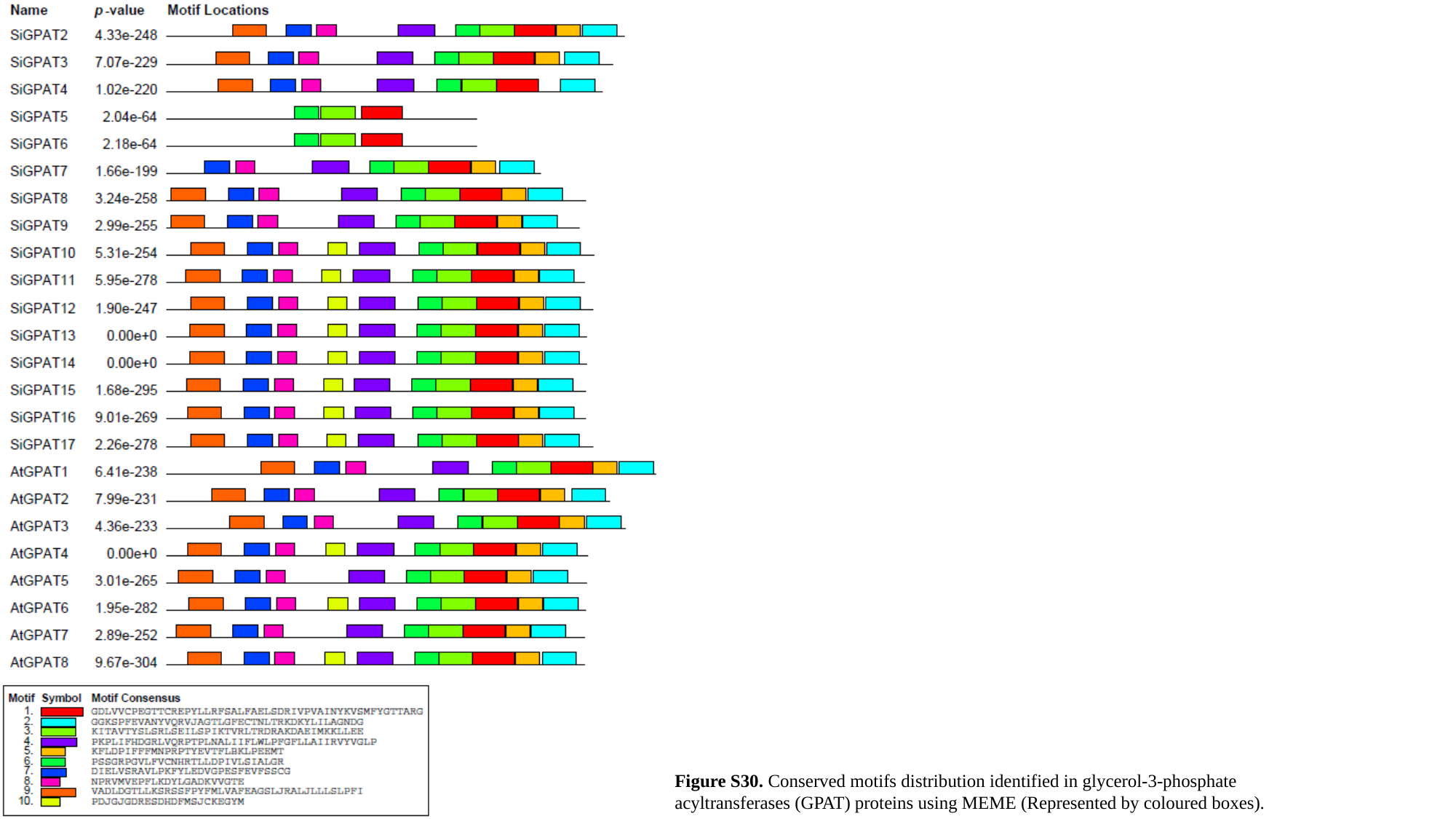

Figure S30. Conserved motifs distribution identified in glycerol-3-phosphate acyltransferases (GPAT) proteins using MEME (Represented by coloured boxes).

## Slide 31
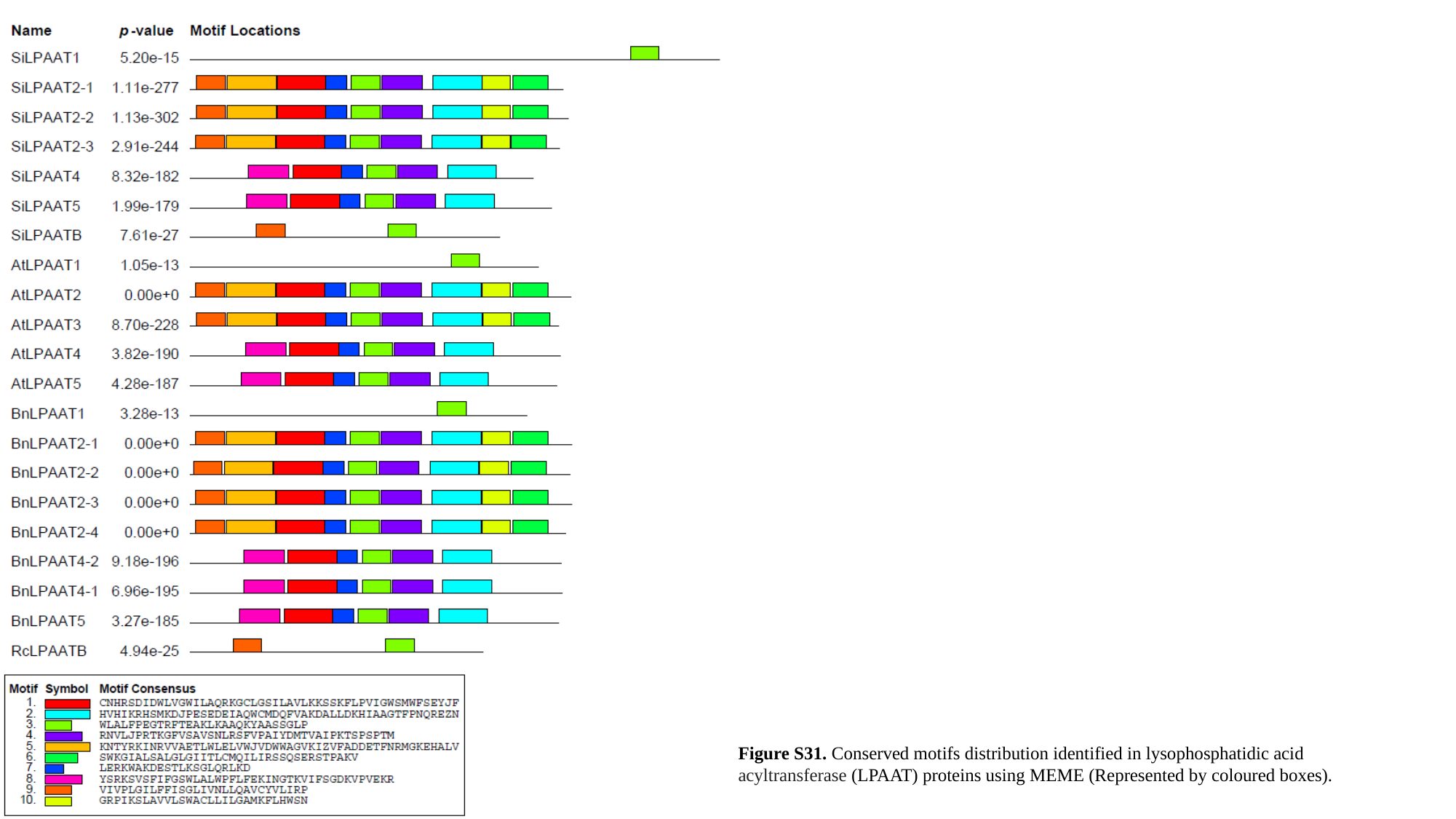

Figure S31. Conserved motifs distribution identified in lysophosphatidic acid acyltransferase (LPAAT) proteins using MEME (Represented by coloured boxes).

## Slide 32
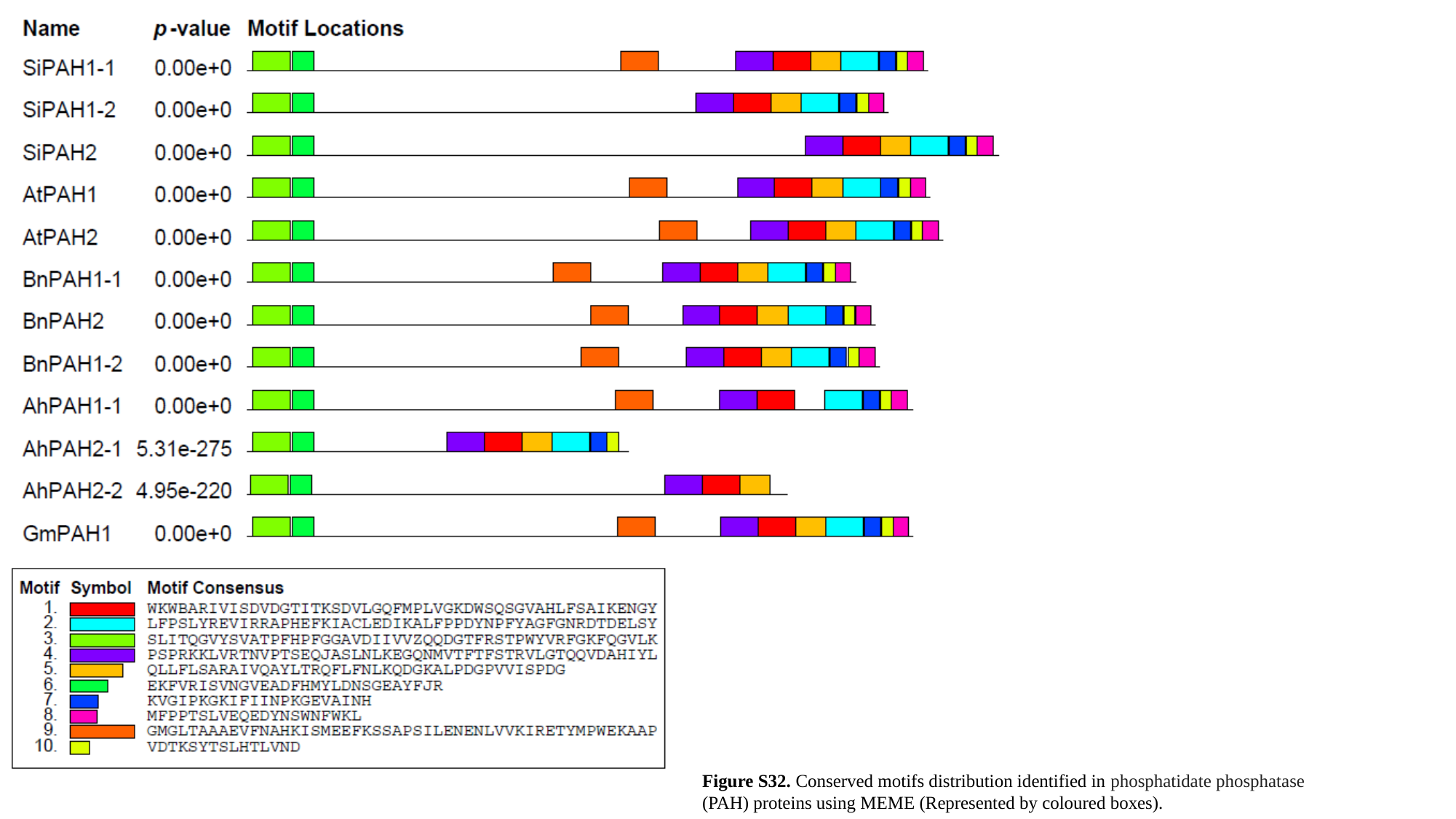

Figure S32. Conserved motifs distribution identified in phosphatidate phosphatase
(PAH) proteins using MEME (Represented by coloured boxes).

## Slide 33
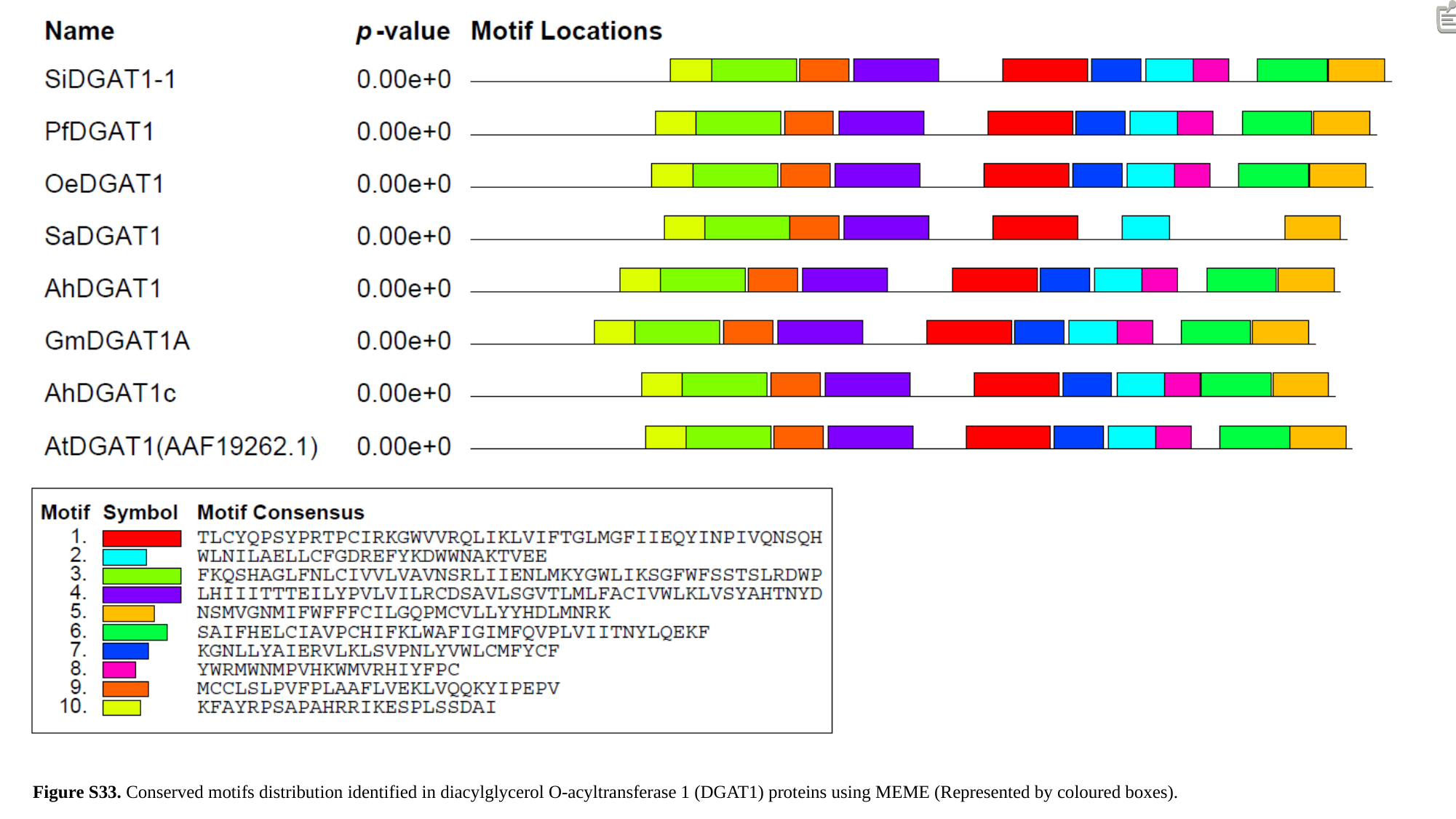

Figure S33. Conserved motifs distribution identified in diacylglycerol O-acyltransferase 1 (DGAT1) proteins using MEME (Represented by coloured boxes).

## Slide 34
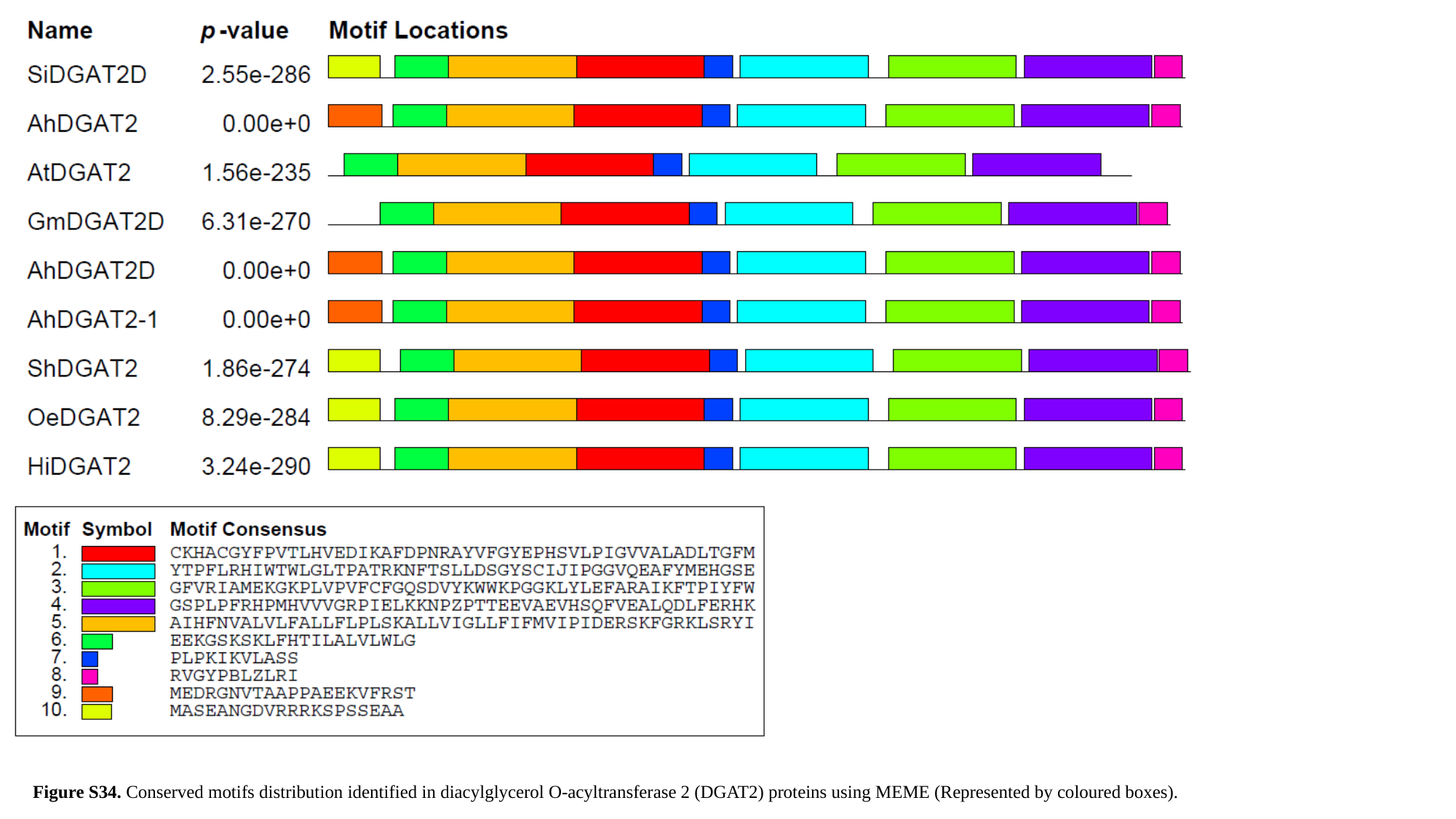

Figure S34. Conserved motifs distribution identified in diacylglycerol O-acyltransferase 2 (DGAT2) proteins using MEME (Represented by coloured boxes).

## Slide 35
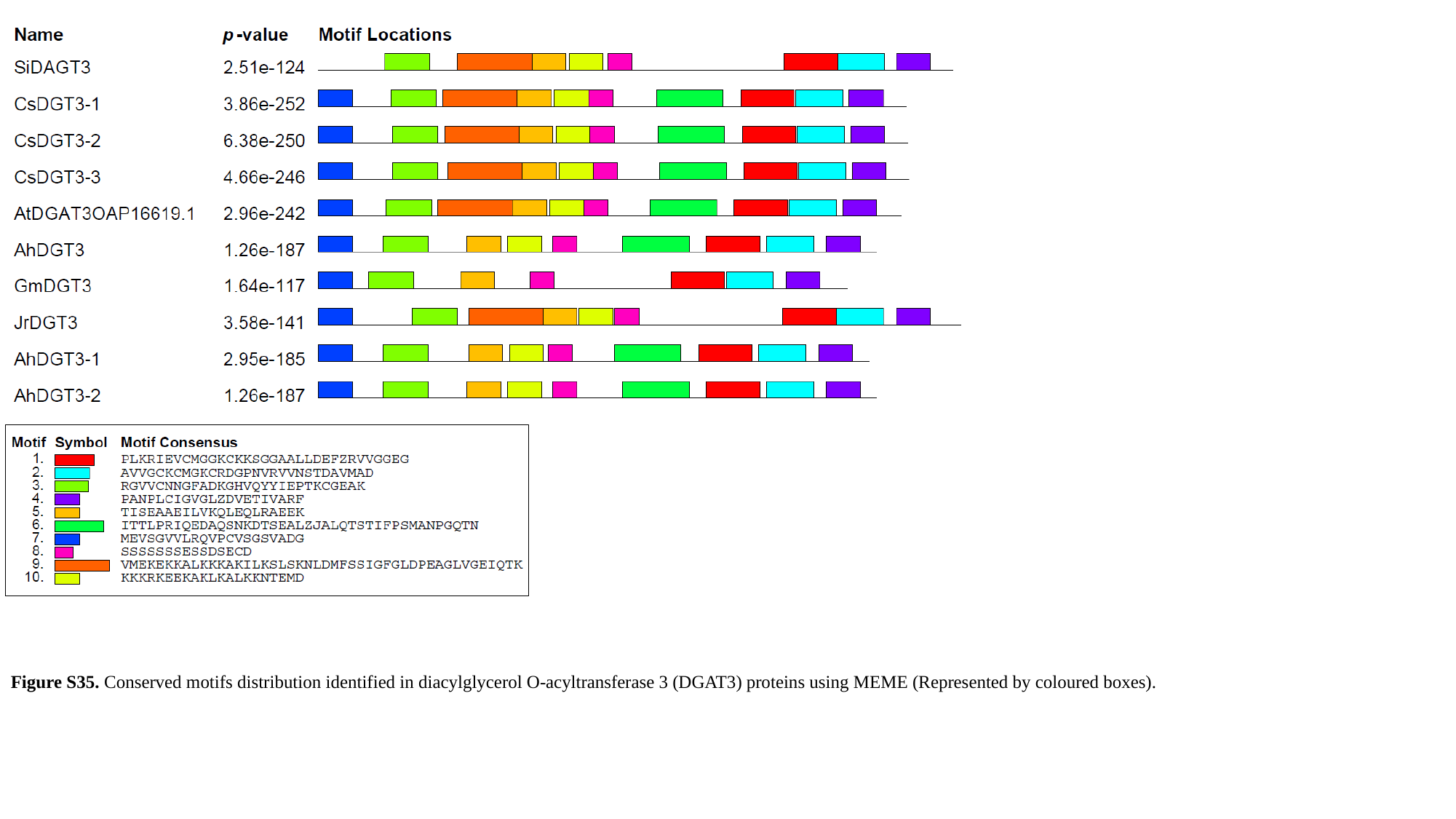

Figure S35. Conserved motifs distribution identified in diacylglycerol O-acyltransferase 3 (DGAT3) proteins using MEME (Represented by coloured boxes).

## Slide 36
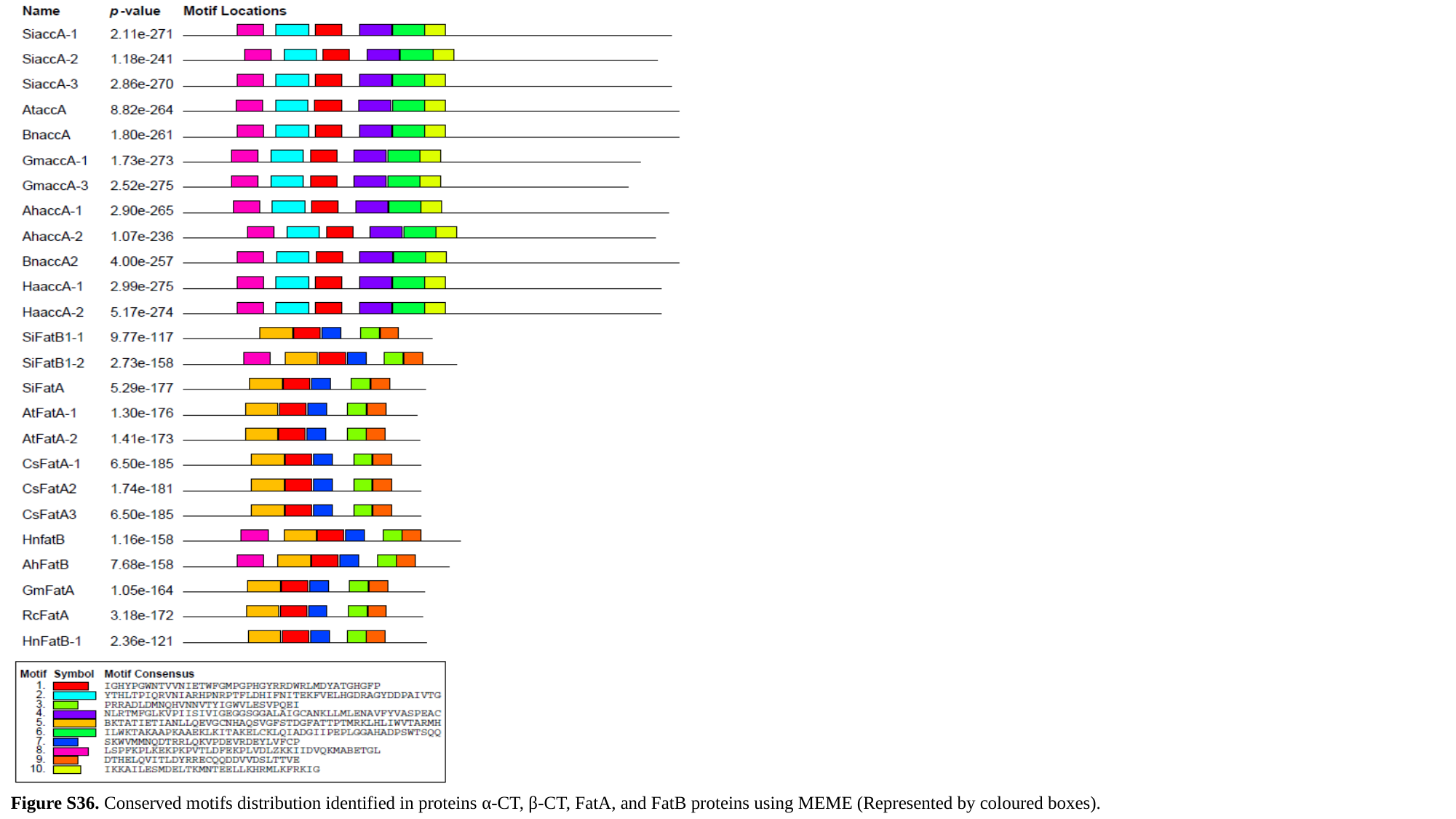

Figure S36. Conserved motifs distribution identified in proteins α-CT, β-CT, FatA, and FatB proteins using MEME (Represented by coloured boxes).
