# Supplementary material for: Longer Duration of Active Oil Biosynthesis during Seed Development Is Crucial for High Oil Yield—Lessons from Genome-Wide In Silico Mining and RNA-Seq Validation in Sesame
Source: Plants (Basel). 2022 Nov 4;11(21):2980. doi: 10.3390/plants11212980 (PMC9657858; doi:10.3390/plants11212980)
Supplement: Supplementary file 1 [file plants-11-02980-s001.zip › Seq_File_S1.pdf]

Seq File S1: Full length amino acid sequence details for the genes and gene families used in this study for *in silico* analysis.

>SiFabD1 Malonyltransferase [*Sesamum indicum*] XP_011080770
MHALVLHHNHRLPLYLVRFNLPSAKSSSLSRAMSSSVVLPSISLTKPALGSSESFRSSTCLGFKNEIRRVS
RLGLGKSRVFMSVAVGSEAVVVDDALFKDYKPSSAFLFPGQGAQAVGMGVEAQKVPAAAELYKRANDILGF
DLLDICINGPKEKLDSTVLSQPAIYVTSLAAVEVLRARDGGQQIIDSVDVTCGLSLGEYTALAFAGAFSFE
DGLKLVKLRGEAMQDAADAAKSAMVSIIGLDSAKVQQLCDAANEEVDEANKVQIANFLCPGNYAVSGGLKG
IEAVEAKAKSFKARMTVRLAVAGAFHTGFMEPAVSRLEAALASTVIRSPRIPVISNVDAEPHADPNTIKKI
LARQVTSPVQWETTVKTLLNRGLKKSYELGPGKVIAGIVKRMDKGADIENICA

>SiSAD6 Palmitoyl-ACP Δ9 desaturase (PAD)[*Sesamum indicum*] XP_011100275.1
MKTLLFSTTIIPLACGGATTFRRPQSISAISKQPVSTLHQRRITHPMPPEKLEVFKSLESWASDYVLPLL
KPVNKCWQPSDFLPDPTQPTDDFMDEVRMLRERTMGLPDEYFVVLVGDMITEEALPTYQSTINACDGIRD
ESGASRSPWATWLRSWTAEENRHGDLLRTYLYLSGRVDMSMIEKTIHQLIGAGMDVGWDNNPYLTFVYTS
FQERATFIAHGNTARLAKEGGDPVLARICGTIAADEKRHEHAYVRIIKKLLEIDPNETMLAISKMMRKRV
IMPSHLMCDGRDPHLFQHFSSVTERIGVYTAIDYIEILEFLIEEWNLEKLEGLKGDGRHAQEHVCDIPTR
MRKVLEQAVERVGKMEAKGQNFSWIFNREVTM

>SiSAD7 Palmitoyl-ACP Δ9 desaturase (PAD)[*Sesamum indicum*] XP_011100276.1
MEILAFSNIIPATALASMPCGGATTFRRPKPISAAANAKQPPSRSRHQKLTHQMPPEKVEVFKSLESWAS
RFVLPLVKPVDKCWQPSEFLPNPAKPADDFMDEVKALRDRTIGLPDEYFVVLVGDMITEEALPTYQSMIN
TCDGVREESGASPCQWAAWVRSWAAEENRHGDLLRTYLYLSGRVDMLMVERTTQHLIRAGADIGLDNNPY
LTFVYTSFQERATFVSHGNTARLAKKAGDPILASICGTIATDEKRHENAYVRIVEKLLEVDPNDTMLAIG
NMMRKRITMPGHLMCDGQDPILFQHFSVVAEQIGVYTIDDYMEILEFLIGRWRLEKLDGLKGDGRREQEY
VCSLPTRMRKLQEQAVERGRKMEPHEQKFSWIFNREVTIQV

>SiLPAAT1 1-acyl-sn-glycerol-3-phosphate acyltransferase 1 [*Sesamum indicum*] XP_011084281.2
MAASIARSILTVRSTGATASLLLHRRFFSSTVSKPPSLPGLARLRISTTRSVTPALSHAVRFSSSTRINQ
VRCRVNRSGSAYSPLNSESNFSDLPPTEMAPLFPGCDYERWLVLMDKPGGEGATKQQMIDCYIQTLAKVV
GSEEEAKKRIYNVSSEMYFGFVCEIDEETSNNLEGLPGVLCVLPDSYVDPENKDCGGIQEQRHLIGLSSR
PLASHDGFHLGCFPHKHPIQRKSKYGLPHETVFGSRTHQYPTLGSSNHKGEFCGTFNGSGSCRRGKFSRD
VVVRSELTSAGSTGASYPLLEIWLNSKVRGICFYAVTACAAIILFVPMLLEHPFVLMFDRYRRQAHYLVA
KTWAFLTVAPFVKIEYEGLENLPPTNTPAVYVANHQSFLDIYTLLTLGRSFKFISKTSIFLYPIIGWAMF
LLGVIPLKRMDSRSQLDCLKQCMALIKKGASVFFFPEGTRSKDGKLGPFKKGAFSVAAKTGVPVIPITLI
GTGEIMPAGMEGRLNPGSVKVIIHPSLVGNKPDALCNEARNIIAQDLIHQS

>SiLPAATB B-class lysophosphatidate acyltransferase [*Sesamum indicum*] XP_020554192.1
METSGLGGFLRNRRFGSYFDASTGPNTEPPPRAMVDGQQKRAGGHEYYTDDDDTGWFPVLISWVRIVVCF
VSMMLTTFIWGVIMVVLLPWPYQRIRQGNIYGHVTGKMLMWILGNPITIQGAEYSNERAIYISNHASPID
IFLIMWLTPTGTVGIAKKEIIFYPLFGQLYVLANHLRIDRSNPKAAIESMKEAADAVVRNNLSLIIFPEG
TRSKNGRLLPFKKGFVHLALQTRRPIVPIILTGTHRAWRKGSLHVRPAPLTVRYLPPIRTDDWTPDKVED
YVKLVHDVYAKNLPHSQRPTPPALSENGGSCRTVAS

>*SiaccB1* Biotin carboxyl carrier protein of acetyl-CoA carboxylase 1 [*Sesamum indicum*] XP_011077154.1
MATTFGAAATAASLTGNANKSSTYLPLASRHFFLRANSNKVSFRLTPRPKLRLFSKGLQSGWKCSAMVKA
QLNDVATDGSSKAEASTPATSEVSPAKSKDTPIALASEESISEFITQVASLVKLVDAKDIVELELKQLDC
EVLIRKKEALAAPPPPVAYVQSQPQPAVLPPVSTPSPAPTAAPTPPPAPALSPPKAQPAKSSHPPLKSPM
AGTFYRSPAPGEPPFVKVGDKVQKGQVLCIIEAMKLMNEIEADQSGKIVEILLEDGKPVSVDTPLFVIEP

>*SiaccB-2* Biotin carboxyl carrier protein of acetyl-CoA carboxylase 2 [*Sesamum indicum*] XP_011072841.1
MASFSVPCPKVSPTLVASSQSTQKLQPIASFPRSDSRSNRALATVAQFQQGCSRRQSDVFKVCAQLNQVA
VEKISNATPALETVPMNKSENQHEIPDESSILTFMTQVKDLVKLVDSKDIAELELKQMDCELIIRKKEAL

SQPSIAAHYSTPPPTHQALLPPQLPPASAPAPASLPSAPASVSPPAPSKPKSSHPPFKCPMAGNFYRSPA
PGAPPFVKVGDKVQKGQVICIIEAMKLMNEIEADQSGTVVEILVDDGKPVSVDMPLFIIEP
>Si*accC* Biotin carboxylase 2 [*Sesamum indicum*] XP_011070473.1
MDSATMNFCSKSVCSPPGLFMGRTTGIRSSQCSFMAGSRVNFPRQRVQASRVGHKSGKRGGALAATCRDD
KILVANRGEIAVRVIRTAHEMGIPCVAVYSTIDKDALHVKLADESVCIGEAPSNQSYLVIPNVLSAAISR
GCTMLHPGYGFLSENAVFVEMCREHGINFIGPNPESIRIMGDKSTARDTMKNAGVPTVPGSDGLLKSTEE
AVRLADEIGYPVMIKATAGGGGRGMRLAKEPDEFVKLLQQAKTEAAAAFGNDGVYLEKFIQNPRHIEFQV
LADKYGNVVHFGERDCSIQRRNQKLLEEAPSPALTPELRKAMGDAAVAAAASIGYIGVGTIEFLLDERGS
FYFMEMNTRIQVEHPVTEMISSVDLIEEQIRVARGEKLRYTQEDIVLRGHSIECRINAEDAFKNFRPGPG
RITAYLPAGGPFVRMDSHVYPDYVVPPSYDSLLGKLIVWGPTRERAIERMKRALNDTIITGVPTTIDYHK
LILDIEDFKNGKVDTAFIPKHEEELAAPQPTQPATTQKQLISTSA

>Si*accA-1* *A*cetyl-coenzyme A carboxylase carboxyl transferase subunit alpha [*Sesamum indicum*] XP_011077598.1
MSSMTQTPASFTGNLAPKPTASDLLRSSSNGVSGVPLKALGRAQLGARMRNFSITARVRKVKKHEYPWPE
DPDLNVKGGVLSHLSPFKPLKEKPKPVTLDFEKPLMDLQKKIIDVQKMANETGLDFSDQIISLENKYQQA
LKDLYTHLTPIQRVNIARHPNRPTFLDHVFNITEKFVELHGDRAGYDDPAVVTGLGTINGRSYMFMGHQK
GRNTKENIKRNFGMPTPHGYRKALRMMYYADHHGFPIITFIDTPGAYADLKSEELGQGEAIAQNLRTMFG
LKVPIVSIVMGEGGSGGALAIGCANKLLMLENAVFYVASPEACAAILWKSAKASPKAAEKLKITAKELTK
LQIADGIIPEPLGGAHADPYWTSQQIKTAILESMDELVKMDTETLLKHRAQKFRKLGGFQEGVPIDPKRK
VNMKKKDKSIVPISKTSESELRDEVEKLKTQILEASKSSVEHPGTGLKEMMEKLKREIDYEYDEAAKALG
MQDKILMLREEVAKARNHNDQLAHPALKEKIDHLKDEFDKNLPSAPNYPNLKYKLGMLKELSKASNLTKK
STTRDELKLEINKRFSEFMDRPDVKQKIETLKAEIASSGVSNIDSKPELKEKVSQMSGELESEFKAVLNS
MGLQVVPSDSEALAKINAFDEEVNMTIDDLVNSSYLKERIELLKTEVEKAGSRPDEDSKSKIQVLQAEIK
QAITEAISFPELKEKYERLAAEILETTKPSVGSNGSSGDENTEDSQVNVNLEANRSFV
>Si*accA-2* Acetyl-coenzyme A carboxylase carboxyl transferase subunit alpha [*Sesamum indicum*] XP_020551382.1
MTTLSLISGNCGRGRGEDTGSGYGLTSHFANEFLSSDTNGRLFRNFNGIRLKDLEGLRKRNGNGFRVFAR
VRKGKKHDYPWPDDINPNSKAPLTYLSYFKPLKEKPKPVTLAFEKPLVDLEQKIIEVQRMADDTGLDFTD
QINALELKYQQALKDLYTHLTPIQRLNIARHPNRPTVLDHILNITEKWVELHGDRAGYDDPAIVTGIGSM
EGRSYMFIGHQKGRNTKENIMRNFAMPTPHGYRKALRMMKYADHHGFPIVTFVDTPGAFADLKSEELGQG
EAIAHNLRAMFGLRVPVITIVTGEGGSGGALAIACANKLLMLENSAFYVASPEACAAILWKSSKAAPKAA
EKLKITAQEHYKRRIADGIIPEPLGGAHADPVWTSLQIKDAILRQMEELTRMNAEELIRHRMLKFRMIGV
GAYREGSEVEPVRKRNMRASEVNAPKFADIESALDDLRKKILKAKGPSDPITYKKIEKLEEDLDQEMTKA
FISMGLIDQIKSLNLELARSPNPDQASNRRLKEKADKIVQEFKRNLSQPGAYIGLKQKLQMLNMVSRLLE
LKEKSEKLKSELNQKLSADIKAKMETWKLAREKSSTGDPLDENLAREVEVAKEELDQVLRSANLHVVGTT
KKENINPLPELREEMLNINRAIKQEIKKVIDEKGLSWKINELKAEAVKDSSSEKAKKLEAELKEVIAAAL
SASPLKDRLDNLRVQHESSVNKVVEGNVGTDNGRW
>Si*accA-3* *A*cetyl-coenzyme A carboxylase carboxyl transferase subunit alpha [*Sesamum indicum*] XP_011094964.1
MASVAQSPVSFTGNLASKSTASDLLKSSINGACGLPLRALGKAQLGAKRRDFAISARVRKVKKHDYPWPE
DPDPNVKGGVLTHLSPFKPLKEKQKPVTLDFEKPLMDLQKKIIDVQKMANETGLDFSDQIISLENKYQKA
LKDLYTHLTPIQRVNIARHPNRPTFLDHIFNITEKFVELHGDRAGYDDPAVVTGLGTINGRSYMFMGHQK
GRNTKENIKRNFGMPTPHGYRKALRMMYYADHHGFPIVTFIDTPGAYADVKSEELGQGEAIAHNLRTMFG
LKVPIVSIVIGEGGSGGALAIGCANKLLMLENAVFYVASPEACAAILWKSAKASPKAAEKLKITATELTK
LQIADGVIPEPLGGAHADPYWTSQQIKTAIVESMDELVKMDTETLLKHRAQKFRKLGGFQEGIPIDPKRK
VNMKKKEQPLVPISKTSEVELRNEVERVKQQILEASKSSTGAPATRLKEVIEKLKRELDYEYDEAAKALG
MDGKILMMREEIAKARNVNDQLAHPALKEKIEQLKDEFDKNLPTAPNYSSLKSKLNMLKALSKALNLRKK
STPKDELKIEINKRFNELVDRPDVKQKIETLKAEIVNSGVSGIGANPELKEKVQKLSRELDSEFMDVLKS
FGLQVVPSAPAAMVKIDAFEEGVSMIIDDFVNSSDLKDKIETLKAEVGKAGNTPDGDSKSKIQALETEIK
QAIIEGISSPELKEKHERLATEVLEATKSSVESTTDDQSKLDESRVNVSLEANRSFI
>Si*accD* *A*cetyl-CoA carboxylase beta subunit [*Sesamum indicum*] YP_004935675.1
MTIHLLYFDANRGQENSMERWWFNSMVFKKELERRYGIKKSTDNLGPTSESEDPNRKGRAKNIHSWRGRD
NSSYSNVDHLFGVKDIRNFSSDDTFLVRDSNGDYYSIYFDFENHIFEIDNDHSFLSELESSFSSYRNSSS
MNNGSSSEDSLYNRYMYDTQSSWNNHITSCVDSYLQSQISIDTSIVSDSSDSYISRCVFDKRRTSSESGR

SSIETHAKSSDLTLGERSNDLDATQNYRDLWVQCENCYGLNYKKFLKSKMNICEQCGYHLKMSSSERIEV
SIDPGTWDPMDEDMVSLDPIEFHSEEEPYKDRIDSYQSKTGLTEAVQTGVGQLNGIPVAIAVMDFEFMGG
SMGSVVGEKITRLIEYATNQFLPLIIVCASGGARMQEGSLSLMQMAKISSALYDYQSNKKLFYVSILTSP
TTGGVTASFGMLGDIIIAEPNSYIAFAGKRVIEQTLNKTVPEGLQAAEYLFEKGLFDLIVPRNLLKSVLS
ELFNLHAFFPLNSNSSRAH

>SiFabH-1 3-oxoacyl-[acyl-carrier-protein] synthase 3 [*Sesamum indicum*]
XP_011069912.1
MANASGLLSPAVAPSVRRRLNPPIGVFQSGSWFSEGVSRRIVCSSTVQGAEKLSPSKSGVPRLVSRGCKLV
GCGSAVPRLQISNDDLSKIVDTSDEWISVRTGIRNRRILSGKDSLTDLAAEAAKKALQMAEVDPDDVDLVL
LCTSTPEDLFGSAPQIQKALGCKNNPLAYDITAACSGFILGLVSASCYIRGGGFNNVLVIGADALSRYVDW
TDRGSCILFGDAAGAVLVQACDSEEDGLFAFDLHSDGEGQRHLRATMNENEKDHELGTNGSLLGLLPKGSS
YSCIQMNGKEVFRFAVRVVPQSIELALEKAGLPGSSIDWLLLHQANQRIIDAVAARLELPSERVISNLANY
GNTSAASIPLALDEAVRKGKVKAGHKIATAGFGAGLTWGSAIIRWG
>SiFabH-2 3-oxoacyl-[acyl-carrier-protein] synthase 3 [*Sesamum indicum*]
XP_011079297
MANASGLFTPTVPTVRRRFSPPLGIYRSGFWFSDGVSRRTVCSSSTQGAEKLSPSGSRVPRLAGKGCKLVG
CGSAVPTVQISNDDLSKIVDTSDEWISVRTGIRNRRILSRKDSLIDLAAEAAKKALQMAEVDPDDVDLVLL
CTSTPEDLFGSAPQIQKALGCKNNPLAYDITAACSGFILGLVSAACHIRGGGFNNVLVIGADALSRYVDWT
DRGSCILFGDAAGAVLVQACNSEEDGLFAFDMHSDGEGQRHLKAGIKENETDKEPGTNGSLVGLLPKGSAY
SCIQMNGKEVFRFAVRAVPQSIESALEKAGLTGSSIDWLLLHQANQRIIDAVATRLDVPYERVISNLANYG
NTSAASIPLALDEAVRSGKVQAGHTIATAGFGAGLTWGSAIVRWG

>SiFabB-1 3-oxoacyl-[acyl-carrier-protein] synthase I [*Sesamum indicum*]
XP_011085258.1
MQSLHSTSLRPSPLDRLRRPRRSTIFPGNNAKPPPQRLPFKVSSSAVTAAPKRETDPKKRVVITGMGLVSV
FGNDVDVYYEKLLSGESGITLIDRFDASKFPTRFGGQIRGFKAEGYIDGKNDRRLDDCLRYCIVAGKKALE
GADLGGDKLDKIDKIRAGVLIGTGMGGLTVFSDGVQALIEKGHRKITPFFIPYAITNMASALLAIDLGFMG
PNYSISTACATSNYCFYAAANHIRRGEADLMIAGGTEAAIIPIGLGGFVACRALSQRNDDPQTASRPWDKD
RDGFVMGEGAGVLVLESLEHAMKRGAPIIAEYLGGAVNCDAYHMTDPRSDGLGVSSCIQSALEDAGVSPEE
VNYINAHATSTIVGDLAEVNAIKKVFKNTSEIKINATKSMIGHCLGAAGGLEAIATVKAITTGWLHPTINQ
FSAEPSVEFDTVANKKQEHEVNVAISNSFGFGGHNSVVAFSAFKP
>SiFabB-2 3-oxoacyl-[acyl-carrier-protein] synthase I [*Sesamum indicum*]
XP_011096538.1
MQALQSAALRPSPLDPLRKPQYPTSLPSNNAKPPVKRLPFTISASASTVSSPKRETDPKKRVVITGMGLVS
VFGNDVDAYYEKLLAGESGITPIDRFDASKFPTRFGGQIRGFKSEGYIDGKNDRRLDDCLRYCIVAGKKAL
EDADLGGDRLGKIDKIRAGVLVGTGMGGLTVFSDGVQALIEKGYRKITPFFIPYAITNMGSALLAIDLGLM
GPNYSISTACATSNYCFYAAANHIRRGEADLMIAGGTEAAIIPIGLGGFVACRALSQRNDDPKTASRPWDK
DRDGFVMGEGAGVLVMESLEHAMKRDAPIIAEYLGGAVNCDAYHMTDPRADGLGVSSCIQSALEDAGVSPE
EVNYINAHATSTVVGDLAEVNAIKKVFKNTSGIKINATKSMIGHCLGAAGGLEAIATVKAITTGWLHPTIN
QFNPEPSVEFDTVANKKQQHEVNVAISNSFGFGGHNSVVAFSAFKP
>SiFabB-3 3-oxoacyl-[acyl-carrier-protein] synthase I [*Sesamum indicum*]
XP_011082304.1
MAAIASASSSGLILEKSGKSSARNGVSMFQYNGLRAVETMHVGSTGIRTTGFISTSSKWGRIKAMASPTVS
APKRETDPKKRIVITGMGLVSVFGSDVDTFYNKLLDGVSGITPIDRFDASEYSVRFSGQIRDFTSKGYIDG
KSDRRLDDVWRYCLVSGKRALDDASLSKDVLEKMDKTRIGVLVGSGMGGISAFSNGVEALVLKGYKKITPF
FIPYSITNMGSALLAIDTGLMGPNYSISTACATANYCFFAAANHIRRGDADIMVAGGAESAITPAGVGGFI
ACRALSSRNDNPQEASRPWDKNRDGFVIGEGAGVLIMESLEHAVKRGANIIAEYLGGAVTCDAHHMTDPRK
DGLGVSSCIIKSLQDAGVAPEEVNYVNAHATSTLAGDLAEVNAIKKVFKDTSEIKMNGTKSMIGHGLGAAG
GLEAIATIKAITTGWLHPTINQYELEPEVTIDTVPNVKKQHEVNVAISNSFGFGGHNSVVVFAPFKP

>SiFabF-1 3-oxoacyl-[acyl-carrier-protein] synthase II, [*Sesamum indicum*]
XP_011093876.1
MAASSVVCSWLMAACMSVACEKDSSISMLSTPSSNSSRISKWAARRRKRALAKCAPQLSGTRNLMTSCLAF
EPCDRFYESSDASEFFFGFGSRNALLHRRRRKFQRPTARSGEAMAIAVHPAMEVTPKKKPSTKQRRVVVTG
MGVETPLGHDPDTFYNNLLEGVSGISEIEAFDCSQYPTRIAGEIKSFSTDGWVAPKLSKRMDRFMLYMLTA

GKKALADAGITEDVMDELNKARCGVLIGSAMGGMKVFNDAIEALRISYRKMNPFCVPFATTNMGSAMLAMD
LGWMGPNYSISTACATSNFCILNAANHIIRGEADMMLCGGSDAVIIPIGLGGFVACRALSQRNTDPTKASR
PWDSSRDGFVMGEGAGVLLLEELEHAKKRGATIYAEFLGGSFTCDAYHMTEPHPEGTGVILCIEKALAQSG
VSKEDVNYINAHATSTPAGDLKEYKALIHCFGENPELRVNSTKSMIGHLLGAAGAVEAVATVQAIRTGWIH
PNINLENPDDGVDTNVLVGPTKERLAIKVALSNSFGFGGHNSS
ILFAPFQ

>SiFabF-2 3-oxoacyl-[acyl-carrier-protein] synthase II [*Sesamum indicum*] XP_011078136.1
MAATSVVCSWLMAACMSVAYEKESSVSMLSSTSLCRLSKWAARRRKRVLAKCAPDFSSFSSLASSCFAFEP
CDDFYKSSHASSGCLFGFASRNLPLLRRRRKLLRSSPHCGEIMATAVSPAMEVTPKQKPPTPQRRVVVTGM
GVETPLGHDPDVFYNNLLQGVSGISEIEAFDCSQYPTRIAGEIKSFSADGWVAPKFSKRMDRFMLYMLTAG
KKALADGGITEDVMEELNKARCGVLIGSAMGGMQVFNDAIEALRISYRKMNPFCVPFAVTNMGSAMLAMDL
GWMGPNYSISTACATSNFCILNAANHIIRGEADMMLCGGSDAAIIPIGLGGFIACGALSQRNADPTKASRP
WDTNRDGFVMGEGAGVLLLEELEHAKKRGATIYAEFLGGSFTCDAYHMTEPHPEGTGVILCIEKALSQSGV
SKEDVNYINAHATSTLSGDLKEYQALIHCFGQNPELRVNSTKSMTGHLLGAAGAVEAVATVQAIRTGWIHP
NVNLENPDDGVNTNLLVGPTKERLDIKVALSNTFGFGGHNSSILFAPYK

>SiFabF-3 3-oxoacyl-[acyl-carrier-protein] synthase II [*Sesamum indicum*] XP_011072620.1
MVVASSVASPLCTWLVAACMTAPSEKGTFCKPSEFIKCPKKLSRSARRKSMVAQKSGSLVSSLYGCGINSL
MSFCPFEPCEEYYNSQGLSPCFALFGENGFSLLFGSKTLPMNRKQRRLNQASNSGKTMVVGVQPEKQVSMK
KKLVTKKRRVVVTGLGVVSPLGHDADVFYGNLLEGISGVSQIEAFDCTEFPTRIAGEIKNFSPDGWVSPKL
SKRADKFMLYLLTAGKKALADGGITDDVMAELDKTRCGIIIGSALGGMKVFSDAIEALRISHKKMNPFCIP
FATSNMGSAVLAIDLGWMGPNYSISAACATSNFCILDAANHIIRGETDMMLCGGSDAAIIPIGLGGFAACQ
ALSQRNDEPTKASRPWDVHCDGFVMGEGAAVLLLEELEHAKRRGATIYAEFLGGSSTCDAYHTTEPHPDGR
GIVLCIEKALAESGVAREDINYINAHATSTPLADLIECQAINHCFGQNTELRMNSTKSMIGHLLGAAGAVE
AVATVKAIQTGWIHPNVNLERPDEGVDANILVGCKRERLDVKVALSNSLGFGGHNSSIVFAAYKDCKATA

>SiKASmt 3-oxoacyl-[acyl-carrier-protein] synthase mitochondrial [*Sesamum indicum*] XP_011077001.1
MAMARRYSNRLVSSIIQFTRFYSSGPFDPPPFLPPRRVVVTGLGMVTPLGCGVEVTWKRLIEGECGIRAVT
IEDLKMNGFDRETQLHTFDQLTSKVAGIVPCGANPGEFNEELWLNSKDHKSTARFISYALCAADEALRDAN
YMPTTQDEKERTGVSIGAGTGSISDILDASKMICEKKLRRLSPFFIPRILINMASGHVSMKYGFQGPNHAA
VTACATGAHSIGDAARMIQFGDVDVMVAGGTEASIDALSIAGFCRSRALTTKYNAVPEEASRPFDCARDGF
VIGEGSGILVLEELEHAKKRGAKVYAEVRGYGMSGDAYHITQPHADGRGAVLAMTRALKQSGLNPNQVDYV
NAHATSTPLGDAVEAKAIQSLFSDHAASGALAFSSTKGAIGHLLGAAGAVEALFSILAIHHGIAPLTLNLS
KPDPVFSGNFMPLSASKKMTIKAALSNSFGFGGTNSSLLFASID

>SiSAD1 Acyl-[acyl-carrier-protein] desaturase [*Sesamum indicum*] NP_001291335.1
MALKLNAINFQSPKCPSFALPPVASVRSPKFFVASTLRSGSKEVETVKRPFNPPREVHVQVTHSMPPQKI
EIFKALEDWADNNILVHLKPVEKCWQPQDFLPDPSSDGFDDQVKELRERAKEIPDDYFVVLVGDMITEEA
LPTYQTMLNTLDGVRDETGASPTSWAIWTRAWTAEENRHGDLLNKYLYLSGRVDMRPNGKTFRYLIGSGM
DPRTENSPYLGFIYTSFQERATFISHGNTARLAREHGDLKLAQICGTIAADEKRHETAYTKIVEKLFEID
PNDTVLAFADMMRKKISMPAHLMYDGRDDNLFDHFSSVAQRLGVYTAKDYADILEHLVARWKVANLTGLS
ADGQKAQDYVCGLPPRIRRLEERAQGRAKQAPKIPFSWVHDREVQL

>SiSAD2 Stearoyl-[acyl-carrier-protein] 9-desaturase [*Sesamum indicum*] XP_011091536.1
MALKLNASNFQYPKCASFALPPVATIRSPKFFMASTLRSGSKEVDTVKKPFSPPREVHVQVTHSMPPQKI
EIFKAMEDWAENNILVHLKPVEKCWQPQDFLPDPASDGFYDQVKELRERAREIPDDYFVVLVGDMITEEA
LPTYQTMLNTLDGVRDETGASPTSWAIWTRAWTAEENRHGDLLNKYLYLSGRVDMKQIEKTIQYLIGSGM
DPRTENNPYLGFIYTSFQERATFISHGNTARLAREHGDLKLAQICGTIASDEKRHETAYTKIVEKLFEID
PDGTVLAFADMMRKKISMPAHLMYDGHDDNLFDHFSAVAQRLGVYTARDYADILEHLVGRWKVENLTGLS
AEGQKAQDYVCGLPPRIRRLEERAQGRARAKEVPRIPFSWIYDREVQL

>SiSAD3 Stearoyl-[acyl-carrier-protein] 9-desaturase [*Sesamum indicum*] XP_011084689.1
MALRVSFVPHRIPAFPASQLRSHRVSMASTLHSPSAEVGNVRKPFSPPREVHVQVTHSMPPEKQEIFNSL
HDWAEENILVLLKDVEKCWQPSDFLPNPSSPGFHDQVKELRERSMELPEDYFVVLVGDMITEEALPTYQT
MINTLDAVRDETGASLTPWAIWTRAWTAEENRHGDLLNKYLYLSGRVDMKQIEKTIQYLIGSGMDPRTEN

NPYLGFIYTSFQERATFISHGNTARLAKDHGDYKLAQICGTIAADEKRHETAYTKIVEKLFEIDPDDTVL
SLADMMRKKISMPAHLMYDGKDDNLFEHFSAVAQRLGVYTAKDYADILEFLVARWEVEKLTGLSSEGRKA
QDYVCGLPPRIRRLEERAQARAKKRSAIPFSWIYGGKIMI

>SiSAD4 Stearoyl-[acyl-carrier-protein] 9-desaturase [*Sesamum indicum*] XP_011077116.1
MALKLSVAAHKMPSFAGYNLRSHRVSMASTLHSTSIENGKVKKPFTPPREVHGQVTHPMPPEKREIFHSL
NSWAEDNILVLLKPVEKCWQPNDFLPDSSSEGFDEQVKELRERTKEIPDDYFVVLVGDMITEEALPTYQT
MLNTLDAVRDETGASLTPWAIWTRAWTAEENRHGDLLNKYLYLSGRVDMKQIEKTIQYLIGSGMDPRTDK
NPYLGFIYTSFQERATFISHGNTARLAKEHGDLKLAQICGIIAADEKRHETAYTKIVEKLFEIDPDGTVL
ALADMMRKKVSMPAHLMYDGRDENLFDHFSAVAQRLGVYTAKDYADILEFLVARWEVEKLTGLSGEGRKA
QDYVCGLPPRIRRLEERAHARAKQASPVPFSWIYGREVKL

>SiSAD5 Stearoyl-[acyl-carrier-protein] 9-desaturase [*Sesamum indicum*] XP_011074783.1
MQSSAFTHIATAQLAWAPRGGGAAATRRVPPSSAVAAPLRHRQITHSMPPEKVEVFKSLESWASKSVLPL
LKPVDKCWQPTEFLPDPTRPISDFMDEVRVLRDRASELPDEYFVVLVGDMITEDALPTYQTMINTLDGVR
DETGASPNPWASWTRGWTAEENRHGDLLRTYLYLSGRVDMLMIERTVQYLIGAGMDPGTENNPYLGFVYT
SFQERATAVSHGNTARLAKEGGDPVLAQICGTIAADEKRHENAYARIVEKLLEVDPNGAMIAVGDMMRKK
ITMPAHLMYDGQDPHLFEHFSAVAQRMGVYTADDYADILEFLIGRWRLEKLDGLKGDGRQAQEFVCGLPQ
RIRKLQERADERAKKMKPKGQRFSWIFNREVVV

>SiFatB1-1 Fatty acyl-ACP thioesterase B [*Sesamum indicum*] (XP_011073093)
MALMQNAALHFPKDLCHAERNDDFVATFVRVGGTRRRAGVKMKASSKTNVDTVNGKKVNGVHVDGRIPFLDQRSADLLK
DGESVYGSVEPNHEYLLGRFVEDRFVYRQSFVIRSYEIGPDKTATMETLMNLLQETALNHVASAGVAGNGFGATREMSI
RKLIWVVTRIHVQVEKYSSWGDVVEIDTWVDAAGKNGMRRDWIIRDYNTQKIITRATSTWVIMNRETRRLSKIPEEVKN
EVQPFYLNRAAIATENTDSEKIEKLTDETAEIIRTGLAPRWSDMDANQHVNNVKYIGWLLESVPINVLEDYNMTRLTLE
YRRECRQSNVLESLTSMKAKTGEEEDKQFTEKSGAEDLECTHLLRMEADQAEIVRARSVWQIKQHDNQMT

>SiFatB1-2 Fatty acyl-ACP thioesterase B [*Sesamum indicum*] (XP_011078334)
MVTTAATSAFLPIASPASDSSGKTLGKVGGSIPANVDARGTSAKSKSSSSGKLRVNANAQAPPKVNGTKVGVIDGPKAE
DEVAVSPPPRTFINQLPDWSMLLAAITTIFLAAEKQWMMLDWKPKRPDMLVDPFGLGRIVQDGLVFRQNFSIRSYEIGA
DRTASVETLMNHLQETALNHVKNAGLLADGFGSTPEMCKRNLIWVVTKMQVLVDRYPTWGDVVQVDTWVAASGKNGMRR
DWLVRDSNTGDILTRATSLWVMMNKETRRLSKIPDEVREEIGGYFVDSPPIVDDDSRKLPKLDDNTADYIRTGLTPRWS
DLDVNQHVNNVKYVGWVLESAPLPILETHELAGMTLEYRRECMRDSVLQSLTAVVDKGVGDLANSGFVECQHLLRLEGG
GEIVKGRTQWRPKYADRIGSWGELPAESA

>SiFatA Fatty acyl-ACP thioesterase A [*Sesamum indicum*] (XP_011089673)
MLLRGGAFSACNAAAGEHQGQGNCRFSGLIRRPTGRVATVWCSRTRRGNLLRVASVATGEPKSKPQESSYQPSVADRLR
LGSLTEDGLSYKEKFIVRCYEVGINKTATVETIANLLQEVGCNHAQSVGFSTDGFATTLTMRKMHLIWVTARMHIEIYK
YPAWSDVVEIETWCQSEGRIGTRRDWILKDYATGEVIGRATSKWVMMNQDTRRLQKVTDEVRDEYLVFCPKKLRLAFPE
ENNASLKKISKLDDPAEYYKVGLVPRRADLDMNQHVNNVTYIGWVLESMPQEIIDSYELQTITLDYRRECQHDDVVDSL
TTPEPILNDSVSQLHGTNGSPTAAKDESDYLQFLHLLRISNDGSEINRGRTEWRKKPAKR

>*SiFAD2-1* Oleic acid desaturase [*Sesamum indicum*] AAX11454.1
MGAGGRMSDPTTKDEQKKNPLQRVPYAKPPFTLGDIKKAIPPHCFERSVSRSFSYVVYDLVIVFLLYYIA
TSYFHLLPSPYCYLAWPIYWAVQGCVCTGIWVIAHECGHHAFSDYQWLDDTVGLILHSALLVPYFSWKYS
HRRHHSNTGSLERDEVFVPKPKSRVSWYSKYLNNPLGRVITLVVTLTLGWPLYLLFNVSGRPYNRFACHF
DPYGPIYNDRERLQIFISDAGIIAAVCVLYRVALVKGLAWLVCVYGVPLLIVNGFLVLITFLQHTHPSLP
HYDSSEWDWLRGALATVDRDYGVLNKVFHNITDTHVTHHLFSTMPHYHAMEATKAIKPILGQYYQFDGTP
FYKAMWREAKECLYVEPDESTPDKGVFWYKNKF

>*SiFAD2-2* Delta(12)-fatty-acid desaturase [*Sesamum indicum*] XP_011075145.1
MGAGGRMSVPPEGKKSKSDAFQRVPHSKPPFTLGELKKAIPPHCFQRSIPRSFSYVIYDLAIASLFYYVA
TNYFHLLPPSLSCLAWPLYWICQGCVLTGVWVIAHECGHHAFSDYQWLDDTVGLILHSALLVPYFSWKYS
HRRHHSNTGSLERDEVFVPKVKSGIRWFAKYMNNPPGRVLMLIVQFTLGWPLYLLFNVSGRHYDRFACHY
DPKSPIYSDRERLQILISDAGVLAVTYGLYRLAIAKGLAWLVCVYGGPLLVVNAFLVLITYLQHTHPALP
HYDSSEWDWLRGALATVDRDYGILNTVFHNITDTHVAHHLFSTMPHYHAMEATKAIKPILGNYYQFDGTP
IFKAVWREAKECIYVEPDEGDKNKGVFWYNNKL

>SiFAD3 Acyl-lipid omega-3 desaturase [*Sesamum indicum*] XP_011080789.1
MAVISGLRHSSFLNTTAAEEINGGPEEKERMKQNTPAAATVTPGGEAFDPAAPPPFRIADIRAAIPAHCW

VKDPWRSFSYVVQDVAAVVGLLSAAVYFDSWAFWPVYWVAQGTLFWAIFVLGHDCGHGSFSDNPVLNNVV
GHFLHSSILVPYHGWRISHKTHHQNHGHVENDESWVPLPEKLYKKLDYSTKFLRYKIPFPMFAYPIYLWS
RSPGKSGSHFNPYSALFRADERKLVMTSTICWAAMVAFLFCASTIIGPLSLFKLYGVPYLIFVMWLDTVT
YLHHHGHEQKLPWYRGKEWSYLRGGLTTVDRDYGLFNKIHHDIGTHVIHHLFPQIPHYHLVEATREAKRV
LGKYYREPRKSGPIPFHLIPSLVNSIQSDHYVSDTGDVVYYQTDTSHFSSLPSSKLHTN
>SiFAD6 Omega-6 fatty acid desaturase [*Sesamum indicum*] XP_011079423.1
MACRIAHSSFLFLGPQKRPTEGNRIFPQSSASSGAYLLKWEGLPQRRSKQNQSLISFRKHKVVKAVAVSV
APPPADSAEHRQQLCQEYGFRQIGETLPDNVTLRNIIDTLPKKVFEIDDVKAWKSVLVSVTSYALGIFMI
AKVPWYLLPLAWAWTGTAVTGFFVIGHDCAHKSFSRNKLVEDIVGTLAFLPLIYPYEPWRYKHDRHHAKT
NMLVEDTAWQPVDPEEFDSSPVLRKAIIYGYGPFRPWMSIAHWLICHFDLKKFRPNEVNRVKISLACVFA
FMAIGWPLIIYKTGIMGWIKFWLMPWLGYHFWMSTFTMVHHTAPHIPFKSSEEWNAAQAQLNGTVHCDYP
SWIEVLCHDINVHIPHHISPRIPSYNLRAAHQSLQENWGKYMNEATWNWRLMKTILTVCHVYHKEQNYIA
FDELAPEDSQPITFLKRVMPDYA
>SiFAD7 Omega-3 fatty acid desaturase [*Sesamum indicum*] NP_001306619.1
MASWVLSECGLRPLPRVYPKPRTGHPLLNSNPTKLRFSRTDLGNGSSFCLSSGILREKNWALRVSAPLRV
LQVEEEEENKEGERVINGGEEFDPGAPPPFKLSDIREAIPKHCWVKDPWRSMGYVVRDVAVVFGLAAVAA
YFNNWVVWPLYWFAQSTMFWALFVLGHDCGHGSFSNDPKLNSVVGHILHSSILVPYHGWRISHRTHHQNH
GHVENDESWHPLSEKIYKNLDTATKKLRFTLPFPLLAYPIYLWSRSPGKQGSHFHPDSDLFVPNEKKDVI
TSTVCWTAMLALLVGLSFVIGPVQLLKLYGIPYLGNVMWLDLVTYLHHHGHEDKLPWYRGKEWSYLRGGL
TTLDRDYGWINNIHHDIGTHVIHHLFPQIPHYHLIEATEAAKPVLGKYYREPKKSAPLPFHLLGDLTRSL
KRDHYVSDVGDVVYYQTDPQLTGAEKS
>SiFAD8 Omega-3 fatty acid desaturase [*Sesamum indicum*] XP_011076709.1
MASWVLSECGVSPLPRLYPKPRTGHAQAFLNPPNPSKLRISRTDFGNSFSFCLVNHKERNWALRVSAPLT
VGQVEEESKENGGEEFDPGAPPPFKLSDIRAAIPNHCWVKDPWRSMSYVVRDVVVVFGLAAVAAYFNNWA
VWPLYWFAQGTMFWALFVLGHDCGHGSFSNDPKLNSVVGHLLHSSILVPYHGWRISHRTHHQNHGHVEND
ESWHPLSEKIYKNLDNVTKKLRFTLPFPMLAYPIYLWSRSPGKTGSHFHPDSDLFVPSERKDVITSTVCW
TAMVALLVGLSFLMGPLQLLKLYGIPYLGFVTWLDLVTYLHHHGHEQKLPWYRGEEWSYLRGGLTTLDRD
YGWINNIHHDIGTHVIHHLFPQIPHYHLIEATEAAKPVLGKYYREPQKSGPLPFYLLGVLLRSMKKDHYV
SDTGDVVYYQTDPELN

>SiLACS1 Long chain acyl-CoA synthetase 1 [*Sesamum indicum*] XP_011079524.1
MKKFAVKVEEGKKGRDGKPSVGPVYRNLLAGNAYPPVDPNLSTAWEIFRASAEKHSGNRMLGWRELVNGK
WGPYVWKSYKEVYEETLHAGSALRAHGSEPGARVGIYSSNCPQWIVAMEACNAHSLICVPLYDTLGTGAV
DYILDHAEIDVVFVQDKKVKELLNPECTHARRLKLIVCFTSLTEEQQGKAASIGIKTCSWTEFLNMGREN
PVEISPPEPSSICTIMYTSGTSGAPKGVILTHENISTNIRGVDLFMDQFEDKMTVDDVYISFLPLAHILD
RMIEEYFFHKGASVGYYHGDINEIRDDLMELKPTFLAGVPRVFERVHEGVLKALEELNWRRRKIFHMLYK
YKLYWMNRGYKQKDASPLADLLAFGKVKNRLGGRIRLIVSGGAPLSSEVEEFLRVTSCAFVVQGYGLTES
CGLSTIGFPDEMCMIGTVGASFVYNEICLEEVPEMGYDPLADPPRGEICLRGKTTFAGYYKNPELTQEAI
KDGWFHTGDIGEMLPNGVVKIIDRKKNLIKLSQGEYVALEYLEKVYGITPIVEDIWVYGDSFKSMLVSVV
VPNEENTKKWAQQNGQKGSFMDLCSLDQLKDHILLELKSTAERNKLKGFEHIKGVIVEPKLFELSEKELV
TATLKKRRDRLLKNYKVEIDALYQTLRAAKP
>SiLACS2 Long chain acyl-CoA synthetase 2 [*Sesamum indicum*] XP_011088325.1
MGDYVVKVEDGRGASDGKPSAGPVYRCIYAKDGLLDIPTHFDSPWNFFSASVKKNPNNQMLGRRQIKDGK
AGAYTWLTYQQVYDATLKIGSAMRRRGVNPGDKCGIYGANCPEWIIGMEACIGQSVTYVPLYDTLGANAV
EFIINHAEVSIAFVQESKLVPILTCLPKCVSHLKTIVSFGKVSDKHKETAEELGVACFSWEEFALLGDLD
ENLPPKKKTDTCTIMYTSGTTGEPKGVILSSAAFMAEVLSMDQLLLETDKVGTEEDVYFSFLPLAHIFDQ
IMETYCIYRGSSIGFWQGDIRFLIEDLIVLKPTIFCGVPRVYDRIYTGIMDKISASGALRKSLFQIAYNY
KLRNLEKGLRQEEASPFFDKLVFDKIKQGFGGRVRLMLSGAAPLPKHIEEFLRVTCCCVLSQGYGLTESC
SGCFTSIANVYPMMGTVGVPMTTIEARLESVPEMGYDALSSVPRGEICLRGSTLFSGYHKRPDLTAEVVV
DGWFHTGDVGEWQPSGALKIIDRKKNIFKLSQGEYVAVESIESVYSRCPLITSIWVYGNSFESFLVAVVV
PEKKALEEWAANNQEKGDFQSLCSNIKARKYMLDELNSTAKTHNLRGFEMLRGVHLEPAPFGIDRDLVTP
TFKLKRPQLLKYYKDCIDQLYSEAKETKA

>SiLACS4 Long chain acyl-CoA synthetase 4 [*Sesamum indicum*] XP_011101042.1
MAKERYTVEVEPGKPAKDGKPSVGPVYRSKFAKDGFPEPIPGLDSCWDIFRLSVEKFPNNRMLGRREIVD
GKPGKYSWMTYKEVYDIVLKVGNSIRSCGIEEGGRCGIYGANSPEWIMSMEACNAHGLYCVPLYDTLGAG
AVEFIICHAEVALAFVEEKKISELLKTFPGATKCLRTIVSFGKVTSQQKDEVEKFGVAIYSWDEFLSLGE
NKDYDLPVKKKTDICTIMYTSGTTGDPKGVMISNNSIVTLIAGVKRLLESVNESLTVNDVYLSYLPLAHI
FDRVIEECFINHGASIGFWRGDVKLLVEDIGELKPTIFCAVPRVLDRIYSGLQQKVSSGGFIKHTMFNLA
YSLKLRSMRKGRKHTEASPLCDKIVFSKVKQGLGGNVRLILSGAAPLASHVEEFLRVVSGSYVLQGYGLT
ETCAGTFVSIPDELSMLGTVGPPVPNVDVCLESVPEMGYDALSSTPRGEVCVRGDTLFSGYYKRDDLTKE
VFVDGWFHTGDVGEWQANGSLKIIDRKKNIFKLSQGEYVAVENLENIYGLVPAIDSIWIYGNSFESFLVA
IINPNKQVVEQWAEENGVSGDFNALCENPKVKEYFLGELSRIAKEKKLKGFEFIKAVHLDPVPFDMERDL
ITPTFKKKRPQLLKYYQGVIDSMYKSGK
>SiLACS6 Long chain acyl-CoA synthetase 6[*Sesamum indicum*] XP_011090746.1
MDSRAERRLQAVRNHLVGSTTWEQSPVLRPQPTVGDFFSVEQGYSIVLPEKLQTGKWNVYRNRRSPLKLV
SRFVDHPEIATLHDNFMHAEKTFRDYKYLGTRVREDGTVGEYKWITYGEAGASRAAIGSGLIRYGIQKGS
CIGLYFVNRPEWLVVDHACSAYSFISVPLYDTLGPDAVKYIVNHAAVQAIFCAPETLKILLRFLSEIPSV
HLIVVVGGVEEQLPSLPSTTGVEFISYSKLHTEGLGSLQPFCSPTADDVATICYTSGTTGTPKGVVLTHG
NLIANVAGASLGVKLYPSDLYISYLPLAHIYERANQILVVYFGGASGFYQGDNMKLLEDMAVLKPTVFCS
VPRLYNRIYAGVMNAVKTSGVLRERLFNAAYKAKKQSIFSGKKSSPMWDKLVFNKIKEKLGGRVRYMVSG
ASPLSPEVMDFLRVCFGCIVVEGYGMTESSCVISNMDETDVLSGHVGAPNAACEIKLVDVPEMNYTSEDQ
PHPRGEICVRGPIVFQGYYKDEVQTREVIDDDGWLHTGDIGLWLPGGRLKIIDRKKNIFKLAQGEYIAPE
KIENVYAKCKFVAQCFIHGDSLNSFLVAVVAVDHDMLKAWAAAEGIKYQDLKQLCADPRARGAVLADMDA
VAREAQLRGFEFAKAVTLVVEPFTVENGLLTPTFKIKRPQAKAYFANAISDMYNQLSASHASAQKLV
>SiLACS8 Long chain acyl-CoA synthetase 8 [*Sesamum indicum*] XP_011085413.1
MMSESAESNLNTNTMKNVGISDYLSIFKDNGTYGVTCAVIVAIVTPILLSLVLQGRKKLKQRGVPVQVGG
EAGFAVRNARFPKLVEVPWEGATTMAALFEQSCKRYSQQNFLGTRKVIAKDLVTSSDGRKFEKLHLGEYQ
WETYRQIFDRACNFASGLLKLGHDVDTRAAIFSETRAEWLIAFQACFRQNITVVTIYASLGDDALIHSLN
ETQVTTLICDSKQLKKLAALSSSLTTLRNIIYFDDEDSSSGSTVTQESGNWQVSSFSEVEKLGRNNPAQP
NLPIKKDIAVIMYTSGSTGLPKGVMMTHGNIVATAAAVMTVIPSIGRKDVYLAYLPLAHVFELAAETVML
TAGAAIGYGSALTLTDTSNKIKKGTKGDASVLKPTLMAAVPAILDRVRDGVVKKVEEKGGIVKKLFKIAY
SRRLVAIEGSWFGAWGVEALLWDIVVFKKIRSILGGEIRFMLCGGAPLSGDTQRFINICMGAPIGQGYGL
TETCAGAAFSEWDDISVGRVGPPLPSCYIKLVSWEEGGYTISDKPMPRGEVVVGGASVTAGYFSNDAKTA
EVYKVDEKGMHWFYTGDIGRFHPDGCLEIIDRKKDIVKLQHGEYISLGKVEAALVSSKYVENIMVYADPY
HNFCVALVVPARQTLEGWAKESGIKYNDFPELCEKAEAKNEVQQSLLKEAKAAKLDKFEIPAKIKLLPDP
WTPESGLVTAALKLKREQLKAKFKDELDKLYK
>SiLACS9 Long chain acyl-CoA synthetase 9 [*Sesamum indicum*] XP_011069534.1
MGPYYVGVIIPLVVTLLLRGTKGKKRGLPVNVGGEPGYAIRNYRFSSPVQSAWEGVTTLAELFELSCKKH
SDMRLLGTRKLIASEVEISDGRSFEKLHLGDYEWLSYGQVFEAVCNFASGLVQLGHQKGERAAIFADTRE
EWFIALQACFRRNVSVVTIYASLGEEALCHSFNETEVTTVICGYKELKKIANVSGQLDTVKRIICMDDEF
QSDASLVPGSSSWTITSFSDVEKLGRENPVDPDLPLSADIAVIMYTSGSTGMPKGVMMTHGNVLATVSAV
MTIVPGLGNQDVYLAYLPLAHILELAAENLIAAIGSSIGYGSPLTLTDTSNKIKRGTKGDATVLRPTLMA
AVPAILDRVRDGVRKKIDATGGLPKKLFDLAYARRLSAINGSWFGAWGLERLLWNLLVFRKVQAILGGRI
RFILSGGAPLSGDTQRFINICLGSPIGQGYGLTETCAGGTFSEYDDTSVGRVGAPLPCSIIKLVDWPEGG
YLISDSPKARGEIVIGGPNVTLGYFKNDDKTKEVYKVDERGTRWFYTGDIGQFHADGCLEIIDRKKDIVK
LQHGEYVSLGKVEAALIVSPYVDNIMLHADSFHSYCVALVVASQASLEAWASKQGITYSDFSDLCGKEET
LKEVSTSLLKAAKAAKLEKFEIPAKIKLLSEPWTPESGLVTAALKLKREVIRKTFSDDLGKLYSS

>SiGPAT1 Glycerol-3-phosphate acyltransferase [*Sesamum indicum*] XP_011069706.1
MIFSTTSSTTLFTCNTTPRVSASPSTSRAACLLKSSCGGASRRSRSSFLVLCASKLSEAIDGKKSSTSST
VAAAPVFASSSAYDQDEISAPRTFLDARTEQDLLLGIQKEAEAGRLPINVAQGMEELYHNYRNAVFHSGH
PKAHEIVLSNMAVALDRIFMDVRDPFQFSPHHKAIREPFDYYLFGQKYISPLLDFRNSFLGNSSLFDEME
KKLQQGENVILISNHQTEADPAIISLLLESTNPFIAENMICVAGDRVVTDPLCKPFSMGRNLLCVYSKKH
MNDDPELIDMKRRANTKSLKEMAMLLRVGSKVIWIAPSGGRDRPDPVTKEWYPAPFDASSVDNMRRLVEH
AGVPGHIYPLAILCYDVMPPPAQVEKEIGEKRVISFHGVGLSVAPKISYQEITATLEDPEEAKNAFSELL

YSSVCEQYDVLRSAIHGKQGLAASTPTVSLSQPWE
>SiGPAT2 Glycerol-3-phosphate acyltransferase 1[*Sesamum indicum*] XP_011082911.1
MTASVKTMEFPMVLLRLADWFLYQFLANSCYRAAMKIKNHGFSFFLKNPSIRSSSSHQFPLYPSLTKCNL
HGRKYQTLVCDIHGCLLRSHSFFPYFMLVAFEGGSIFRAFLLLLSCPFLLILDYELKLRAMIFLTFCGLR
LKDMESVSRAVLPKFYLENLNFHAYEAVCSAGSKVVFTSVPRVMVEGFLKEYLSVDSVVGTELQTKGKFF
TGLVSSSGLLVKHRALKELFGDKRPDIGIGSSSLHDHLLISLCKEAYVVSKEEAKSNPSSVMPRDKYPKP
LVFHDGRLAFLPTPFATLSMFMWLPIGILLAVFRLFVGICLPYNLAILLGIASGVNLKVKGCPPMKSQNG
KGVLYVCTHRTLLDPVFLSTSLGKPLTAVTYSLSKMSEILAPIRTVRLTRDRKRDGETMQKLLSEGDLVV
CPEGTTCREPYLLRFSSLFAELADEIVPVAMNTDVTMFYGTTASGLKCLDPIFFLMNPRPSYSVEILGKV
PRELTCAGGKSSYEVANYIQKELGMALGFECTSLTRRDKYLMLAGNEGVVQEKRRKP
>SiGPAT3 probable glycerol-3-phosphate acyltransferase 3 [*Sesamum indicum*] XP_011094070.1
MAPKAFPFSKLALLFSAFLDNFRDTRRNLGNHWSPRSPFEFQNLEFLGQRPHLENQVLVFHAETVLLRSS
SLFPYFMLVAFEAGSLIRALILFVLYPFICLCHQDLALEVMVFICFLGLKKERFTEGRAVLPKFFLEDVG
RESFEVLRRGKLIVAISDLPQVMVESFLKDFLDVDFVVGRDLKACRGYFVGLMEERRDYNAFQVVKLSPH
VIGVGCFRKCFEHEWFSNCKEIYLVDEQERRNWHQLPRDLYPKPLIFHDGRLAFRPTFWATLAMFMWLPM
GFTLGIIRTIVVLSSLPFEIKFPILHLTGVRIKISNSELATMSNVNGALYVCNHRTLFDPLMLSSCLRTP
LTAVVYSLSRVSEILSPIKTVRLTRNRGQDAELMSKVLSQGNLVVCPEGTTCREPYLLRFSPLFTEISDN
IFPVAIDCHVSMFYGTTARGFKSLDPLFFSMNPRSTYSARFLNMVHRGAGDENGDDARTSNYKMANLVQS
MIAEELGFSCTKLTRKDKYLILAANEGVVRKGGELKTETETKMK
>SiGPAT4 Probable Glycerol-3-phosphate acyltransferase 3 [*Sesamum indicum*] XP_011077903.1
MALMKPLSVQKLPLLFSILILKHFRNPKRTTHQGNFYTADSVEYHKFQVLGQRQDLEDQFLIFNVETSLL
RSSSLFPYFLLVAFEAGSPIRAFIFLILYPFITVCRDGLALKLMVMICFFGISKDRFREGRAVLPKFFLE
NVGRESFEVLRRGKKTVGVSNLPHVMVESFLRDHLDIDYVVGKDLKVFCGYFVGLMEERRKLIVPDNVLH
NAIGISGCKKDFDCPWFAHCKEIYLVNEQERRNWHQLPRNSYPKPLIFHDGRLAFQPSFLAALAMFMWLP
LGFTLAIIRTLIALTLPFEIAIPLMHFIGIRIRVSKPHSFSNRKHGEAKRRLYVSNHKTLLDPIVVSYAT
RNTTLTAVTYSLSRMSEIISPIKTVRLTRHRDQDAELMHKLLSQNDLVVCPEGTTCRESYLLRFSPLFSE
ISNEVFPVAVNCHISMFYGTTARGLKFLDPFFFLMNPWPSYSARFLGVIHRAGGSRFEAADLVQSEIGKA
LGFTCTKLTRKDKYLILAGNDGVVRDGSRRT
>SiGPAT5 Glycerol-3-phosphate acyltransferase 3-like [*Sesamum indicum*] XP_011088689.1
MSKLNKSSSELDLDRPNIEDYLPSGSLQDSHPKLRLRDLLDISPTLTEAAGAIVDDSFTRCFKSNPPEPW
NWNVYLFPLWCLGVVVRYGVLFPLRAIVLTTGWIIFLSCYFPVHFLLKGHDKLRKRLERSLVELICSFFV
ASWTGVVRYHGPRPSMRPKQVFVANHTSMIDFIILEQMTAFAVIMQKHPGWVGLLQSTILESLGCIWFNR
SESKDREIVARKLREHVNGADNNPLLIFPEGTCVNNHYTVMFKKGAFELGCTVCPIAIKYNKIFVDAFWN
SRKQSFTTHLLQLMTSWAVVCDVWYLEPQNLKPGETPIEFAERVRNIISVRAGLKKVPWDGYLKYSRPSP
KLRELKQQSFAESVLRHLEQK
>SiGPAT6 Glycerol-3-phosphate acyltransferase 3-like [*Sesamum indicum*] XP_011092934.1
MSKLNTSSSELDFDRPNIEDYLPSGSIQEPHGKLRLRDLLDISPTLTEAAGAIVDDSFTRCFKSNPPEPW
NWNIYLFPLWCLGVVIRYGLLFPLRVIVLTIGWIIFLSCYFPVHFLLRGHDKLRKRLERGLVELICSFFV
ASWTGVVKYHGPRPSMRPKQVFVANHTSMIDFIVLEQMTAFAVIMQKHPGWVGLLQSTILESLGCIWFNR
SESKDREIVAKKLREHVHDADNNPLLIFPEGTCVNNHYTVMFKKGAFELGCTVCPIAIKYNKIFVDAFWN
SRKQSFTTHLLQLMTSWAVVCDVWYLEPQNLKPGETPIEFAERVRDIISVRAGLRKVPWDGYLKYSRPSP
KHRERKQQSFAESILRRLEEK
>SiGPAT7 Probable glycerol-3-phosphate acyltransferase 3 [*Sesamum indicum*] XP_011094147.2
MLVAFEAGGLIRALILFLLYPMISLCRQELALKVMVFVCFLGLKKERFVEGRAVLPKFFLEDVGRESFEV
LRRGKMTVGVSDLPQVMVESFLRDYLEVDFVVGRELKVFRGYFVGLMEERRERGFQVVKMAPDVIGIGCF
RKCLDREWFLNCKEIYLVSEQERRNWHQLPRDLYPKPLIFHDGRLAFRPTFWATLSMFMWLPIGFTLGII
RAIVALSSLPFEIKFPILHLTGAQIKISNPELATTNNANGALYVCNHRTLLDPLMLSYCLRTPLTAVVYS
LSRVSEIISPIKTARLTRNRGQDAELMHKLLSQGNLVVCPEGTTCREPYLLRFSPLFTEISDRIFPAAID
YDVSMFYGTTARGFKSLDSLFLLMNPQPSCSVRFLNMVRGGADETGDDVETSKYKTANLVQAMIAEELGF
TCTKLTRKDKYLILAGNEGIVGDGKKV

>SiGPAT8 Glycerol-3-phosphate acyltransferase 5-like [*Sesamum indicum*] XP_011085137.1
MEYCVVSELEGTLLKDHDPFSYFMLIAFEASGLIRFALLLALWPFIRLLEALGRGDDGLKLMIFVATAGV
PFSEIEAVARAVLPKFYLDDINMDAWRVFSSYPKRVVVTKMPKIMVERFVMEHLRADHVVGSDLSMNRFG
FATGFLKDDFGSVAKRVAQLFGDDEKPCLGLGRPDSGCSSLFLPLCKEECHPPFITNHKNHHQHQIIIRP
LPVIFHDGRLAKRPTPSTALLIIILWVPLGIILAAIRIAVGLMLPMWATRHIAPLLGGRVVVKGNPPPPPS
RRSSTSGVLFVCTHRTLMDPVVLSTVLRRRIPAVTYSISRLSEILSPIPTIRLTRIRDVDATKIKQELEK
GDLVVCPEGTTCREPFLLRFSALFAELTDQIVPVAMNYRVGLFHATTARGWKAMDPIFFFMNPRPVYEVT
FLNQLPVEATCSAGKSPHDVANYVQRILAATLGFECTNFTRKDKYRVLAGNDGNVSSTDGGGAAGLVDAF
KKIVPSFIILH
>SiGPAT9 Glycerol-3-phosphate acyltransferase 5-like [*Sesamum indicum*] XP_011076807.1
MESVVSELEGTLLKDPHVFSYFMLIAFEASGLVRFAFLLALWPLIRLLEVCGRGDDGLRLTVFAATVGVP
VSEIEAVARAVLPKFYLDDINMRAWRVFSSYDRRVVVTRMPRIMVERFGKEHLMAGDVVGTELSVNRFGL
ATGFIKSGIGSIGKKVTELFGDEKPRLGLGRPKCGFSSLFMPLCQEECHPPFPNRKPMHENHIIRPLPVI
FHDGRLVKRPTPSAALVILLWIPLGILLAAIRIAVGLMLPMWAIPRVAPAFGGRVVVKGTPPPPASASNS
GVLFVCTHRTLMDPVVLAAAIQRRIPAVTYSISRLSEILSPIPTVRLTRNRDVDAQKIKRELEKGDLVVC
PEGTTCREPFLLRFSALFAELTDRIVPVAMNYSVGFFHPTTARGWKAMDPIFFFMNPRPIYEVTFLNQLP
VEATCSAGKSPHDVANYVQRILAAALGFECTNFTRKDKYRVLAGNDGTVSQTSEPDTKMVNAFKKVVASF
MLH
>SiGPAT10 Glycerol-3-phosphate 2-O-acyltransferase 6-like [*Sesamum indicum*] XP_011073520.1
MAVPKTNTPNRIPTIFQCDSKGRENHTVVADLDGTLLIGRSSFPYFALIAFDVGGILRLLLLLLASPLAG
FLYYFVSEAAGIRVLIFATFAGVKVSAIKSAASAVLPKHYSEDMHPETWRVFSSCGRKCVLTANPRIMVE
PFLKNYLGVDVVLGTEISSYKGIATGFVASAGVLVGRNKAIALRRAFGAESMPDIGIGDRKTDFPFMRLC
KERYIVPSRPEVRPVRHDALPKPVIFHDGRLVQKPTPLMALLIIILWFPIGIILSILRVLVGSLSPISLTY
YIVNFMGCPITVKGTPPRGAKSSSTRRGVVFVCSHRTVLDAVFVSASLGRMTTTISYSVASFTEFLSPIK
TSRLTRERTKDAQLINDILDQGRYLVMCPEGTTCREPYLLRFSSLFAELTDQIVPVAIVVKTSMFHGTTA
RGHKWLDPFFFFMNPVPAYEVTFLNKLSPDQTCSAGKSSHEVANSVQEMIAKTLNYKCTNLTRKDKYRAL
AGTDGLVGQQPVRAATKVIGG
>SiGPAT11 Glycerol-3-phosphate 2-O-acyltransferase 6-like [*Sesamum indicum*] XP_011086432.1
MAAGEFPTVDVCPSIGREKDTVVADMDGTLLRGRSSFPYFALVAFEVGGVLRLLFLLLLAPLAGVLYYFV
SESAGIRVLIFATFAGMRVSDIESVARAVLPKFYSGDLHSESWRVFSSCGKRCVLTANPRIMVEAFLKEF
LGADMVLGTEVSTYGGRATGFVKEPGVLVGKNKAEALKAAFGEAPPEIALGDRHTDFPFMSLCKEGYIVP
PKPEVKAVTIEKLPKPIIFHDGRLVQKPTPFLALLTILWIPVGFLLACLRIAAGSLLPMPLVYYAFWALG
VRVTVKGTPPPPVHKSSTQSGVLFICSHRTLLDPIFLSTALGRPIPAVTYSVSRLSEIISPIKTVRLSRD
RATDAAMIKKLLQEGDLAICPEGTTCREPFLLRFSSLFAELTDELVPVAMVNRMSMFHGTTARGWKGMDP
FYFFMNPSPAYEVTFLNKLPRELTCGAGKSSHEVANYIQRVIAATLSYECTTFTRKDKYRALAGNDGTVV
EKPRRQVMGC
>SiGPAT12 Glycerol-3-phosphate 2-O-acyltransferase 6-like [*Sesamum indicum*] XP_011091226.1
MAVRSTHSPSKIPTIYQCDSKGRENQTIAADMHGTLLIDRSLFPYFALIAFDVGGILRLLLLLLTAPLAW
FLHHFVSESAGIRVLVFASFAGVKVSEIESAANAVLPKHYSEDLHPETWRVLSSCGRKCVLTESPRIMVE
PFLKNYLGVDLVLGTEISSWWGVATGFMARGGVLVGEQKAMALRKAFESSLAPEIGIGDSEADFAFMNLC
KEKYIVPSEQRVRPVKQVELPKPVIFHDGRLVQKPSPLIALLIIVWFPIGVLLSVSRVLIGSNSPISLFY
YIIQLTGCKILVKGTPPPNAKNSRRTGVAFICSHKTVMDPLFVSAVLGRNTTCISYSVSRLTEFLSPIRN
CRLTRERSKDAKIIKDILEEGWDLVMCPEGTTCREPYLLRFSSLFAELTDEIVPVAINIRTSMFHGSTAR
GRKWLDIFFFFMNPLPVYEITFLDKLSPDQTCSAGKSSFDVANNVQEMIGTVLKFKCTKFTRKDKYRMLA
GTDGLVGGKPGAVAADKLS
>SiGPAT13 Glycerol-3-phosphate 2-O-acyltransferase 4 [*Sesamum indicum*] XP_011092551.1
MASAAAVAARFPSIEKCDTSGENRRSIAADLDGTLLISRSSFPYFMLVAIEAGSLLRGLILLLSFPLIAV
AYIFVSESLAIQMLIYISYSGLKVRDIELVSRAVLPRFYANDVRPESYEVFDKCDRKVIITANPTIMVEP
FVKEYLGGDKVVGTEIEVDPKTKKATGFVKKPGVLVGKWKKLGVLKEFGEEKPDIGIGDRESDHDFMSIC
KEGYMVHPSKSAKPLPLDRLKSRLIFHDGRLVQCPTPLNALITYIWLPFGFILSLIRVYFNLPLPERIVR
YTYPMLGINLVIKGNPPPPPSAGSPGNLYVCNHRTALDPIVIAIALGRKVSCVTYSVSKLSRFLSPIPAV

ALTRDREADAARIKALLQKGDLVVCPEGTTCREPYLLRFSALFAELSDRIVPVAVNLKQNMFHGTTVRGV
KFWDAYFYFMNPRPTYEITFLERLPEEMTCKGGGKSSIEVANHVQRILAGALGFECTQLTRKDKYMLLGG
NDGKVESIYSKK
>SiGPAT14 Glycerol-3-phosphate 2-O-acyltransferase 4-like [*Sesamum indicum*] XP_011093132.1
MAKTATAAVRFPSIEKCDASGENRQAIAADLDGTLLISRSSFPYFMLVAIEAGSLLRGLILLLSFPIIAI
AYVFVSEALAIQMLIYISFSGIKVRDIELVSRAVLPRFYARDVRAESYEVFDKCKRKVIITANPTIMVEP
FVKEFLGGDKVIGTEIEVDPKTRKATGYVKKPGVLVGKWKKLGVLKEFGEEKPDIGIGDRASDHDFMSIC
KEGYMVPPSKTAKPLPLDRLKSRLIFHDGRLVQRPTPLNALITYIWLPFGFLLSLIRVYFNLPLPERIVR
YTYPMLGINLVIKGNPPPPPSPGSPGNLYVCNHRTALDPIVIAIALGRKVSCVTYSVSKLSRFLSPIPAV
ALTRDREADAAMIKALLQKGDLVVCPEGTTCREPYLLRFSALFAELSDRIVPVAVNIKQNMFYGTTVRGV
KFWDAYFYFMNPRPTYELTFLERLPEEMTCKAGGKSSIEVANHVQRILAGTLGFECTQLTRKDKYMLLGG
NDGKVESMYSKK

>SiGPAT15 Glycerol-3-phosphate 2-O-acyltransferase 6-like [*Sesamum indicum*] XP_011077573.2
MGAHRHFHPITKCTTQGRSNQTVAADLDGTLLVSRSAFPYFMLVALEAGSLIRALLLLSSVPFVYLVYLF
VSESLAIQSFIFISFSGLKIRDIELVSRSVLPKFYSEDVHPETWKVFNSFGKRYIVTANPRIMVEHFAKS
YLGADKVLGTELEVTKSGRATGFVKKPGVLVGEHKKMAIVKEFGSNVPDLGLGDRETDHDFMSICKEGYM
VPRTATEPLPRNKLLSPIIFHEGRLVQRPTPLVALVTFLWMPIGFVLSVIRIYLDIPLPERIVRYTYMML
GIKLIVKGTPPPPPSPGRGGVLFVCNHRTILDPVVTAVALGRKISCVTYSISKFSELISPIKAVALSRER
EKDAANIKRLLEEGDLVICPEGTTCREPFLLRFSALFAELSDRIVPVAINTKQSVFYGTTARGYKVLDPY
FVFMNPRPTYEITFLNQLPEELTVKGGKSAIEVANYIQRVLAGTLGFECTNLTRKDKYIMMAGTDGRVQP
NKDKDDREHKK
>SiGPAT16 Glycerol-3-phosphate 2-O-acyltransferase 6-like [*Sesamum indicum*] XP_011094988.1
MGAHREFEPITKCKSSEGRSNQTVAADLDGTLLVSSNAFPYLMLVALEAGSLIRALFLLASVPFVYFLYL
FLSESLAVQILIFIAFSGLKIRDVELVAKSVLPKFYAEDVHPETWRVFNSFGKRYIVTANPRIMVEHFVK
NYLGVDKVLGTELQVTKSGRATGFVKKPGVLVGDHKKMAIVNEFGTNVPDLGLGDRETDYDFMSICKEGL
IVPRTRTEPLPRNKLLSPIIFHEGRLVHKPTPLVAFLTFLWMPIAITLSFVRVFLHIPLPERIVRYTSMM
LGIKLIVKGTPPPPPRPGQGGRLLVCNHRTVLDPLVTAVALGRKITCVTYSISKFSESISPIKTVGLSRE
TEKDAATIKRLLEEGDLVICPEGTTCREPFLLRFSALFAELSDRIVPVAVNTKQCVFYGTTTRGYKFLDP
FFVFMNPRPTYEITFLNEVPREFSVRGGKSGIEVANYVQSAIAGKLGFQCTNLTRKDKYAMMAGTDGRVH
SKYSGLIMPAK
>SiGPAT17 Glycerol-3-phosphate 2-O-acyltransferase 6-like [*Sesamum indicum*] XP_011098557.1
MAESKLHSSVTFPSISKCESHGREKHTVVADMDGTLLIGRSSFHYFALVAFEVGGMLRLLFLLLASPLAG
ILYYFISESAGIRVLIFATFAGMKVSDIESVGRAVLPKFYSGDLHPETWRVFSSCGMKCVLTANPRIMVE
AFLKEYLGADLIIGTEISTYKGRATGLVTSPGILVGKNKAEGLRKAFGNTTPEIGIGDRKTDYPFMNLCK
ESYMVHRTPKVQPVSQDRLLKPIVFHDGRLVQKPSPLMALLIIILWIPIGFILACIRIAAGALLPMPMVYY
AFRALGVRVTIKGTPPPRPEKSLGQTGVLFVCSHRSLLDPIFLSTALGRAIPAVTYSLSRLSEIISPIKT
VRLNRDRAKDADMIKKLLKEGDLVICPEGTTCREPFLLRFSALFAELTDELVPVAMSNRMSMFHGTTARG
WKGMDPFYFFMNPSPAYEVTFLNKLPGDLTCGAGKSSHEVANYIQRMIAATLSYECTSFTRKDKYRALAG
NDGNVVEKPRKIAADKVMGS

>SiLPAAT2-1 1-acyl-sn-glycerol-3-phosphate acyltransferase 2 [*Sesamum indicum*] XP_011075205.1
MAIAAAIVIVPLGVLFFVSGLVINFIQAVCFLLIRPLSKKTYRRINREVAGLLWLGLVWLIDWWAGVKIE
LYTDSETFKLMGKEHALVICNHKSDIDWLVGWVLAERSGCLGSTLAVMKKSSRFLPVIGWSMWFSEYLFL
ERSWAKDESTLKSGLQRLRDFPRPFWLALFVEGTRFTQAKLLAAQEYASSAGLPVPRNVLIPRTKGFVTA
VSHLRSFAPAIYDVTVAIPKTSPAPTMLRLFKGQSSVVHVHLKRHLMKDLPETDEAIAQWCRDAFVAKDK
LLDKHNDEDSFGEQLQDTSRPVKSLLVVCCWAILLILGTLKVIQHYSLLSSRKGAALSAIALAVVTILMQ
ILIQFSQSERSTPAKVVPSKSRNGVEPSRAN
>SiLPAAT2-2 1-acyl-sn-glycerol-3-phosphate acyltransferase 2-like [*Sesamum indicum*] XP_011078878.1
MAFPAAVVIVPLGVLFFMSGLVVNLIQAICYVLIRPLSKNTYRRINREVAELLWLELVWLVDWWAGVKIE

IYTDAETFKLMGKEHALVICNHRSDIDWLVGWILAQRSGCLGSTLAVMKKSSKFLPVIGWSMWFSEYLFL
ERSWAKDENTLKTGLQRLRDFPRPFWLALFVEGTRFTQAKLLAAQEYASSTGLPIPRNVLIPRTKGFVTA
VSHMRSFVPAIYDATVAIPKTSPTPTMLRLFKGQSSVIHVHLKRHLMNDLPETDEAVAQWCRDAFVAKDK
LLDRHIAEGTFGEQLQNTGRPVKSLLVVSSWAILLILGTMKVIQWFSLFSSWKGIIFSAVALAVITILMQ
ILIQFSQSERSTPAKVAPAKHKSVTESSASRLDKHQ
>SiLPAAT3 1-acyl-sn-glycerol-3-phosphate acyltransferase 2 [*Sesamum indicum*] XP_011080283.1
MAIAAAVFLPLGILFLLSGFIVNLIQAVLFVLIRPVSKSAYRRINKEIIELLWLELIWLFDWWANIKVEL
FVDQETFDLLGKERALLICNHRSDIDWLVGWVLAQRAGCLGSSLAMIKKSSLFLPVIGWSMWFSDYIFLE
RSWAKDENTLKSGLVALNDYPLPFWLALFVEGTRFTQAKLEAAQEYAASAGLPVPRNVLIPRTKGFVAAV
THLRSFVPVIYNITVAIPKNEPRPTLLKIFRGRSSAVHVHIERHIMRDLPETSSGISQWCKDMFVAKDAL
LERHLATNTFGDEIRHDIGRPKKSLLVVLTWSSILILAIIKVLECFPFSWGEIVFCVVFLALVTTLMQIL
IVFSQSEHSTPPKVSKPDHLRQVLLQS
>SiLPAAT4 Probable 1-acyl-sn-glycerol-3-phosphate acyltransferase 4 [*Sesamum indicum*] XP_011074489.1
MSMEISQPLQSTKGLKHFPLTPFRFFRGLLCLAVYLSTAFMFLVYFVPLVAVVLRLISVHYSRKSVSFLF
GLWLGLWPFLFEKINKTRVIFSGDRVPVEQRVLIIANHRTEVDWMYLWNLALRKGCLGYIKYILKSSLMK
LPVFGWGFHILEFIPVERKWEVDEPVMRQMLSTFTNREDPLWLAVFPEGTDYTEQKCLKSQKFANENGLP
VLKNVLLPKTRGFNVCLEILRGSLDAVYDVTIAYKNQCPSFLDNAFGIDPSEVHMHVRRIPLEEIPVSKD
EAAAWLMNAFVLKDQLLTDFIVNGHFRQESTENQLSTVKCVANCIVVIVFTGIFTYLTFFSSHWFKVYVG
>SiLPAAT5 Probable 1-acyl-sn-glycerol-3-phosphate acyltransferase 5 [*Sesamum indicum*] XP_011088344.1
MESDSVLKLKRGQRHRPLTPFRVIRGVLCLIVLVLTAFVLLVYFGFWTAIPLRFFSMHHSRKATAFIFGS
WIALWPFWFEKINRTKVVFSGDCVPAEERVLLIANHRTEVDWMYLWDLALRKGCEGYIKYILKSSLMKLP
VFGWVFHVMEFIPVVRKWEADELMMRQMLSSFRDPRDPLWLAVFPEGTDFTEQKCVRSQKYASENGLPIL
KNVLLPKTKGFHACLENLKDSLDAVYDITIGYRQNCPSFLDNAFGVDPSEVHIHVRRISLKEIPTSEDQT
SSWLMNTFSLKDQMLSDFYTQGHFPNEGTETSLSTVKCVVNFILVMILTGTCTFLTFFSSIWFKIYVSLV
CAYMASATYFDVRPSPIVH

>SiPAH1-1 Phosphatidate phosphatase 1 [*Sesamum indicum*] XP_011077414.2
MNVVGKVSSFITQGVYSVATPFHPFGGAVDIIVVQQNDGTFRSTPWYVRFGKFQGVLKGAEKVVRIEVNG
VEANFHMFLDNSGEAYFVREVDPDKDSENLEDGREDNSTDFSNSDGGSKENDFSGQDASEFNNDEVDEIE
VRDERVTLGMDRLERVESDSGIFYELHDEQSSIEGSVEFSEYDSSGFNSLESVEHALESQDSNSEVVLVS
VDGHVLTAPISASERNAENVELGTPQFHLGPGEGTENFNRVEDPWNADYLRELDASAHEVTTENICKGNS
ETRSSEQQLELSGGDGEHLCHVEETQDSADQDKELCTESSEYTTTRFKRREVFKSCVELLELELQSPTNN
QENVGSSVEILEAANDLNEKSPLSPSINETEEGNVEISRDSNELSTHDTGDPGSVSSPELQVEAATNERN
TSHMNHDGSDSMPVQSVSNNQELQKQLEAHPTAKRVHNDQESHTDECSKSESHADASIEDGKSDLLAKLE
ISLCGNLLHAGMGLNAAEDAFNANCVSLEEFKLSASSIVKNENLIVRLQGKYLPWCKAAHIVLGMAAYGL
DLPVDVHDIIPVEQEVPGAKEDDSATTPTQSGGWRLWRPFRRVKTLEHNVSNSSNFVDSDSVSQGQRVSS
PLARSVSSPVARAVSTPTARAAADSPRKKIIRTNVPTTDEIASLNLKEGQNMVNFVFSTRVLGSQKVEAH
IYLWKWNTRIVISDVDGTITKSDVLGQFMPLVGKDWTHSGIARLFCAIKENGYQLLFLSARAIVQSYLTK
SFLFNLKQDGKSLPSGPVVISPDGLFPSLFREVIRRAPHEFKIACLEDIKALFPPDYNPFYAGFGNRDTD
ELSYRKIGIPKGKIFIINPKGEVAINNRVDVKSYTSLHTLVNDMFPPTSLVEQEDYNSWNYWKMPLPDID
>SiPAH1-2 Phosphatidate phosphatase 1 [*Sesamum indicum*] XP_011094787.1
MDVVGKVSSFITQGMYSVATPFHPFGGAVDIIVVKQQDGTFRSTPWYVRFGKFQGVLKGTEKVVRIEVNG
VEANFNMYLDNSGEAYFVREVYPGKDDDECLTNSENIDVNSIGSSNIDDGLKESDLYGQEVNDFNNGELE
VQNKHLNLGRGRLQRVESDTGYVFYEFQDEPSLEGSVELPEYGSNTCDTLETVEDALESENSNSEVVLVS
VDGHILTAPISSSETDAEDVELDTPQFHLGPGAGTEEYDTGETTWTSDYLSDLHSSAHNIDTEETCDANS
GTLPPKHPSEPCEGDQEHLFHTQETQATTDNGKEVCINSSSGSTSSALKRDDVFKSCLELSELAVQSANT
NKVDVGSSDEIHEATEDPHKKSPGSPLGNPETEKRNHEILTKDGLSPTDSGYPISISLPEVQVEAAIHER
NTLDMDDDGSDSKHLQFVSSNQEPQDQTDVDLAVESKPSGEEVHASDDGYCKSELAEPHKAAPAPNEDTK
SDASTRPEISLSENVQHAGMESRTVEDAFDPNHTSIKPHDIIPGNVETAERKIDDSSMTSARWRLWPIPF
RRVKTLEHSDTISSCDEVFVDSECVSPRQPPTTPIARVATDSPRKQIIRTNVPTTDQIASLNLKEGQNMV
NFVFSTRVLGSQKVEAHIYLWKWNARIVISDVDGTITKSDVLGQFMPLVGKDWTHSGIARFFSAIKENGY
QLLFLSARAIVQAYLTKNFLFNLKQDGKSLPDGPVVISPDGLFPSLYREVIRRAPHEFKIACLEDIKALF
PPDYNPFYAGFGNRDTDELSYRKIGIPKGKIFIINPKGEVAINHRVEVKAYTSFHTLVNDMFPPTSLVEQ

EDYNSWNYWKMPLPDVE
>SiPAH2 Phosphatidate phosphatase 2 [*Sesamum indicum*] XP_020550995.1
MQAVGKLGSYISRSVYTVSGPFHPFGGAVDIIVVEQPDGSYKSSPWYVRFGKFQGVLKTKEKVVSISVNG
VEADFHMYLDHKGEAFFLKEVDIDAGESPGSPPSSLGEDMDKQPQSRLPLKSKSCNYNSDFPDSIGNERN
GNCVAVGRSTSRRSQILSLVFGRRTMKEEGGQEEKNAYDMVRTDSLERAEIAADLLDLKWSTNLASSRNR
KDNASRFSAVDTSKDEANINLQVGSSSYDETGLISHTSYQELKSTLEENVVEMKCLSTECLVQTPTKAES
VGYTNLSDADALMTDKRGLAESSVITVSEFAKAGSRIEDSVEKLNGLADTSFSESNPRDRTRSLYHCQAS
GTSRVVLDASIERKTNLICHGGCEEVCVQCEAVQDMALKSEPERDMIHAQEKSLTIQHGLRKGDGLSFVQ
EEKTTMNRGTIYGASESTVTECYPELVPLHQSNDYIKDVDSQSVATASSLSYSTCSPAEEQTILAKGEIN
KHSTSVGPVCDTHISVSNFVPPCIQASRISEEEQLLFGIPDNSGYIDGKQMGLSHADLEGENADSSFPSG
VAGVNESNDATGCSVFSLDHSVIDDYINDANLERRKLRSISGDVCLNKTGHVQSKELMRMVRSLPSLPLR
NNLEVSDLGHASNPSLYPGMEGGANRNNHQLPCAQTMAEDVNVLKENKEEHTNPSIGNSPRFVDSLRGSR
RGWPFSFKRSRSMKVSHVDTNSSEIPSAVKFLNDSSELEGGKDVANGKVNKKIVRTLTPTSEQLASLKLK
EGKNIVIFTFSTAMLGKQQVDARIFLWRWDARIVISDVDGTITRSDLLGQVMPLVGMDWSQSGVAHLFSA
IKENGYQLLFLSARAISQSFHTRQFLFNIKQDGKALPDGPVVISPDGLFPSLFREVVRRAPHEFKIACLE
DIKALFPGDRNPFYAGFGNRDTDEFSYLKVGIPKGKIFIINPKGEIVVNRRVDTKSYLSLHALVHGMFPT
MLSSEQEDFNSWNYWKLPPPAIDV
>SiDGAT1 Diacylglycerol O-acyltransferase 1 [*Sesamum indicum*] NP_001291334.1
MAILDSPESFDTTSSSAGNDGDHRSTLRRRPSARAAEVLLDSNSNSLEADTVVNGAESDRNDGNSTGNLC
GRVVESLNEEQRSESAGEGLSYVKEKEEEKGKENGEIGEGKGGEALAVKFAYRPSAPAHRKIKESPLSSD
AIFKQSHAGLFNLCIVVLVAVNGRLIIENLMKYGWLIKSGFWFSSTSLRDWPLLMCCLSLPVFPLAAFLV
EKSVQQNYISEWVAVFLHVIITTTEILYPVVVILRCGSAVLSGVTLMLFACIVWLKLVSYAHTNYDLRVL
SKSLDKWEALSSYWNVDYSYDVSFKSLVYFMVAPTLCYQPSYPRTACIRKGWVVRQLVKLVIFTGLMGFI
VEQYINPIVQNSQHPLKGDLLYAIERVLKLSVPNLYVWLCMFYCFFHLWLNILAELLCFGDREFYKDWWN
ARTVEEYWRMWNMPVHKWMVRHIYFPCLRNGIPKGVAVLVAFFVSAIFHELCIAVPCHIFKFWAFLGIMF
QVPLVIVTNFLQDKFKNSMVGNMMFWCFFCILGQPMSVLLYYHDLMNRKASAR
>SiDGAT2 Diacylglycerol O-acyltransferase 2D [*Sesamum indicum*] XP_011098009.1
MALEADGDVGQRKSPSAEAAAPSTPAEFRGSQGPVLHTILALVLWLGSVHFNVVVVLVSFIFLPFQKALG
VIGLLLILMVIPIDERSKLGRKLSRYICKHAVGYFPVNLYVEDIKAFDPNEAYVFGYEPHSVWPIGVVAL
ADHTGFMPLPKMKVLASTAVFYTPLLRHVWSWLGLAPATRKNFTSLLLSGYSCIIVPGGVQEAFYMERGS
EIAFLQKRKGFVRIAIETGKPLVPVFCFGQSDVYKWWKPSWKLYREFARAIKFAPIVFWGVLGSPLPFRR
PLHVVVGRPILLKKNPQPTEEEVAEVHSQFVEGLEGLFERHKTRVGCTDVQLRIL
>SiDGAT3 Diacylglycerol O-acyltransferase 3,[*Sesamum indicum*] XP_011086024.1
MDSAAVVLKQPLRISSSCYSASPSKELRTFSESVMVGRRRPRPGILSSGFCDQGHLQYYNSNSGPSSSRG
MTMMTVMSGKQDKTTTEKAPKKMKKKKQLKLLKGLSRDLSTFSQMGFGLDSDVALLDQVKGNMISEAAEL
LLEQLQKLRADEKKLKRKMKEEKARLKAANKQSAHMDCERSSSSSSESSDSECGEVIDMTDLKRAQAKQS
IMQQVIEEATSSYSTPVMPLESNVSEARLVVPVFPSITIPEESQLRSPHNVENECCSGTSSSFINTGNLV
EENVSSPSCSKKIEVCMGGKCKKSGAGALLEEFQRVVGIEGAVSGCKCMGKCRDGPNVKVLRGFDGGVAD
ADAAVRAPANSLCIGVGLEDVNMIVANFLGENHNQLGLAAAS

>AtaccB1 Acetyl-CoA Carboxylase biotin-containing subunit [*Arabidopsis thaliana*] AAC49114.1
MASSSFSVTSPAAAASVYAVTQTSSHFPIQNRSRRVSFRLSAKPKLRFLSKPSRSSYPVVKAQSNKVSTG
ASSNAAKVDGPSSAEGKEKNSLKESSASSPELATEESISEFLTQVTTLVKLVDSRDIVELQLKQLDCELV
IRKKEALPQPQAPASYVMMQQPNQPSYAQQMAPPAAPAAAAPAPSTPASLPPPSPPTPAKSSLPTVKSPM
AGTFYRSPAPGEPPFIKVGDKVQKGQVLCIVEAMKLMNEIESDHTGTVVDIVAEDGKPVSLDTPLFVVQP
VESAP

>AtaccB2 Biotin carboxyl carrier protein 2 [*Arabidopsis thaliana*] NP_568316.1
MASLSVPCVKICALNRRVGSLPGISTQRWQPQPNGISFPSDVSQNHSAFWRLRATTNEVVSNSTPMTNGG
YMNGKAKTNVPEPAELSEFMAKVSGLLKLVDSKDIVELELKQLDCEIVIRKKEALQQAVPPAPVYHSMPP
VMADFSMPPAQPVALPPSPTPTSTPATAKPTSAPSSSHPPLKSPMAGTFYRSPGPGEPPFVKVGDKVQKG
QIVCIIEAMKLMNEIEAEKSGTIMELLAEDGKPVSVDTPLFVIAP
>BnaccB1 Biotin carboxyl carrier protein of acetyl-CoA carboxylase 1, [*Brassica napus*] XP_013649844.1
MASSSFSLTSPAASVYGATQASSQRPLPTTRSRLPRSVSFRLSAKPKLRFLSKPSRSSYPVVKAQSNQVG
GNASSKASAPVKIDESSAEEKDSNTSSSAELATEESISEFLTQVTTLVKLVDSRDIVELQLKQLDCELVI
RKKEALPQAQTPAPYVMMQQPNQPSYVQATAPPPPSAPAPSTPASSPPPSPPSPAKSSLPTVKSPMAGTF
YRSPGPGEPPFIKVGDKVQKGQVLCIVEAMKLMNEIESDQTGTVVDIVAEDGKPVSLDTPLFVVQP
>BnaccB2 Biotin carboxyl carrier protein of acetyl-CoA carboxylase 2, [*Brassica napus*] XP_013728269.1
MASLSVPYAKISASNRRVGSIPGIRRQPQLNAVSFPSDVSQTQSTTWKLRATTNEVVSNSTPVTNGGCLN
GNVKSNVPESAELSDFMAKVSGLLKLVDSRDIVELELKQLDCEIVIRKKEALAAPPAPVYHSMPPPMAGF
PMAPSHPVAPPPFSPSPSSAPETAKPVTPPSSSHPPLKSPMAGTFYRSPGPGEPPFVKVGDKVQKGQVVC
IIEAMKLMNEIEAEKSGTITELLAEDGKPVSVDTPLFTIVP
>GmaccB2 Biotin carboxyl carrier protein subunit [*Glycine max*] AAG44776.1
MASFTIPCPKCVVVPFAHLGLNSQTQQRNALGLKKSLSFGSLSSDSAPNGIQCLNKKQSSVWKLQAQPKE
AVTVENSAPVQVNGPKIAPPEEKDDHNGKPSGPSTSADASSISAFMNQVSDLVKLVDSKDIMELQLKQAN
CELVIRKKEALLPPPATFVAPVSQPFPYPTNSLPAAPPSVATSTPASSPSSKAAPALPPAKASKSSHPAL
KCPMAGTFYRSPAPGEPPFVKVGDKVQKGQVICIIEAMKLMNEIEADQSGTVAEVVAEDGKPVSVDTPLF
VIVP
>GmaccB1 Biotin carboxyl carrier protein subunit [*Glycine max*] AAG44776.1
MASSLAPATKAATNLRLTHSLRFSPKPNNLRFATKPGNTLLCTRVKAQLNEVALDSSSNATSPPMKAKSK
EEPPAKPLAEPSSSVLATQESVSQFITQVASLVKLVDSRDIVELKLKQHDVEVTIRKKEAMPQPPPAPQP
SVVYSPPPPALPPPPVPASTPAPTLARATPTPTSAPAVKSAKSSLPPLKSPMAGTFYRSPAPGEPSFVKV
GDKVKKGQVVCIIEAMKLMNEIEADQSGTIVEIVAEDAKSVSVDTPLFVIQP
>AhaccB1 Biotin carboxyl carrier protein of acetyl-CoA carboxylase,[*Arachis hypogaea*] XP_025649626.1
MASSFASTASSASSSLPTPSKPKPKINHFRFSHSNLSFRLSPKPNLPFLTKGSPPCQIVCPRVKAQLNEV
SLDGSSNAVAPTTANSEAEATAKPSSGTSSGVLASQESISQFITQVASLVKLVDSRDIVELQLKQHDCEV
MIRKREAMPQPQPPAQPAMYYPPPSLAAPPVAPASSPAPAPTPATRAASASPPAAKSTKSSLPPLKCPMA
GTFYRSPGPGEPPFVKVGDKVKKGQVLCIIEAMKLMNEIEADQSGTIVEILAEDGKPVSVDMPLFVIEP
>AhaccB2 Biotin carboxyl carrier protein of acetyl-CoA carboxylase 2, [*Arachis hypogaea*] XP_025681786.1
MASFTVPCPKCPSPSLGLNSQKLLKPSLSFGSLAAESASSGIRCLNGKQFSVQKLQAQRREAVTTIENSA
PVLVSGPKVAAPNEKEDQNGKPGGTTTDPSLVSAFMAQVADLVKLVDSRDIVELQLKQSDCELMIRKKEA
LEPPPQVIAPASAPMHYAAYPSPPPPPPPVAASSTPASSPPAKAAPALPSPGKASTSGHPPLKCPMAGTF
YRSPAPGEPPFVKVGDKVQKGQVICIIEAMKLMNEIEADQTGTITEILAEDGKPVSVDTPLLVIAP
>AhaccB1-1 Biotin carboxyl carrier protein 1-1 [*Arachis hypogaea*] ACO53609.1
MASSFASTASSASSSLPTPSKPKPKINHFRFSHSNLSFRLSPKPNLPFLTKGSPPCQIVCPRVKAQLDEV
SLDGSSNAVAPTTANSEAEATAKPSSGTSSGVLASQESISQFITQVASLVKLVDSRDIVELQLKQHDCEV

MIRKREAMPQPQPPAQPAMYYPPPSLAAPPAAPASSPAPAPTPATRAASASPPAAKSTKSSLPPLKCPMA
GTFYRSPGPGEPPFVKVGDKVKKGQVLCIIEAMKLMNEIEADQSGTIVEILAEDGKPVSVDMPLFVIEP
>AhaccB2-1 Biotin carboxyl carrier protein 2-1 [*Arachis hypogaea*] ACO53611.1
MASFTVPCPKCPSPSLGLNSQKLLKPSLSFGSLAAESASSGIRCLNGKQFSVQKLQAQRREAVTTIENSA
PVLVSGPKVAAPNEKEDQNGKPGGTTTDPSLVSAFMAQVADLVKLVDSRDIVELQLKQSDCELMIRKKEA
LEPPSQVIAPASAPMHYAAYPSLPPPPPPAAASSTPASSPPAKAAPALPSPGKTSTSGHPPLKCPMAGTF
YRSPAPGEPPFVKVGDKVQKGQVICIIEAMKLMNEIEADQTGTITEILAEDGKPVSVDTPLLVIAP
>Gh*accB1* Biotin carboxyl carrier protein of acetyl-CoA carboxylase [*Gossypium hirsutum*] ABU41516.1.
MASSLSTTSSAAFTSVAKTTTALPNSTNLQLSIVSFRFSSRRNLRFFSKSLQHGQNSKTVVKAQLNEVAI
DGSSNASAAPTIKSGAPTAEAKDAKTSSDVSPLAMATEESIAEFLNQVSSLVKLVDSRGIVELQLKQLDC
ELVIRKKEALPQPPSAAPVVMMQSPSQLPVMPPPPSVPALPAGQASAAPTPAPSLAASKSAKSSLPPLKC
PMARTFYRSPAPGEPPFVKVGDKVQKGQVLCIIEAMKLMNEIEADQSGTMVEILAEDGKAVSVDMPLFVI
EP
>AtaccC Acetyl Co-enzyme a carboxylase biotin carboxylase subunit [*Arabidopsis thaliana*] NP_198386.1
MDASMITNSKSITSPPSLALGKSGGGGVIRSSLCNLMMPSKVNFPRQRTQTLKVSQKKLKRATSGGLGVT
CSGGDKILVANRGEIAVRVIRTAHEMGIPCVAVYSTIDKDALHVKLADEAVCIGEAPSNQSYLVIPNVLS
AAISRGCTMLHPGYGFLSENALFVEMCRDHGINFIGPNPDSIRVMGDKATARETMKNAGVPTVPGSDGLL
QSTEEAVRVANEIGFPVMIKATAGGGGRGMRLAKEPGEFVKLLQQAKSEAAAAFGNDGCYLEKFVQNPRH
IEFQVLADKFGNVVHFGERDCSIQRRNQKLLEEAPSPALTAELRKAMGDAAVAAAASIGYIGVGTVEFLL
DERGSFYFMEMNTRIQVEHPVTEMIYSVDLIEEQIRVAMGEKLRYKQEDIVLRGHSIECRINAEDPFKGF
RPGPGRITSYLPSGGPFVRMDSHVYSDYVVPPSYDSLLGKLIVWAPTREKAIERMKRALNDTIITGVPTT
INYHKLILDVEDFKNGKVDTAFIVKHEEELAEPQEIVAVKDLTNATV
>CoaccC Biotin carboxylase [*Camellia oleifera*] ACS14663.1
MDSALTFCKSVHSPPGLFVGRSRGIQSPQCSVMVGSRVNFPRQSVRVAQISRKSEKRGGALCATCRDEKI
LVINRGEIAVRIIRTAHEMGIPCVAVYSTIDKDALHVKLADESVCIGEAPSSQSYLVIPNVLSAAISRGC
TMLHPGYGFLAENAVFVEMCREHGINFIGPNPDSIRVMVDKATARETMKNAGVPTVPGSEGLLQNTEEAV
RLSEEIGFPVMIKATAGGGGRGMRLAKEPDEFVKLLQQAKSEAAAAFGNDGVYLEKYIQNPRHIEFQVLA
DKFGNVVHFGERDCSIQRRNQKLLEEAPSPALTPELRKAMGDAAVAAAASIGYIGVGTVEFLLDERGSFY
FMEMNTRIQVEHPVTEMISSVDLIEEQIRVAMGEKLRYKQEDIVLRGHSIECRINAEDAFKNFRPGPGRI
TAYLPSGGPFVRMDSHVYPDYVVPPSYDSLLGKLIVWAPTRERAIERMKRALNDTIIIGVPTTIDYHKLI
LDVEDFKNGKVDTAFIPKHEQELAAPQKMVPATPAKELTKATA
>AhaccC2 Biotin carboxylase 2, [*Arachis hypogaea*] XP_025636875.1
MEATMAACNSLSSPSVPIPGLYAGTSRGIKNSQCSFLGATKVNFPSQTMPGTCQLNHKHKTRSGALHATC
QGDKILVANRGEIAVRVIRTAHELGIPCVAVYSTIDKDALHVKLADESVCIGEAPSSQSYLLIPNVLSAA
ISRRCTMLHPGYGFLAENAVFVEMCREHGINFIGPNPDSIRVMGDKSTARDTMKNAGVPTVPGSDGLLQS
TEEAIRLANEIGFPVMIKATAGGGGRGMRLAKEPGEFVKLLQQAKSEAAAAFGNDGVYLEKYIQNPRHIE
FQVLADKYGNVVHFGERDCSIQRRNQKLLEEAPSPALTPELRKAMGDAAVAAAASIGYIGVGTVEFLLDE
RGSFYFMEMNTRIQVEHPVTEMISSVDLIEEQIRVAMGAKLRYKQEDIILRGHSIECRINAEDAFKGFRP
GPGRITAYLPSGGPFVRMDSHVYPDYVVPPSYDSLLGKLIVWAPTREKAIERMKRALDDTIITGVPTTIE
YHKLILDIEDFRNGKVDTAFIPKHEEELTMPPQKMVPAINKAKELVGATV
>AhaccC1 Biotin carboxylase 1 [*Arachis hypogaea*] XP_025700754.1
MESRIMAALNSVTSPHLPSHSPGLYAVENSIKSSQCSFSAGSKKVSFPRQRCSHVTKTRAARDGGAGGAL
GATCQAEKILVANRGEIAVRVIRTAHEMGIPCVAVYSTIDKDALHVKLADDAVCIGEAPSSQSYLLIPNV
LSAAISRRCTMLHPGYGFLAENAVFVEMCREHGINFIGPNPDSIRVMGDKSTARDTMKKAGVPTVPGSDG
LLQTTEEAIRLANEIGFPVMIKATAGGGGRGMRLAKEPDEFVKLLQQAKSEAAAAFGNDGVYLEKYVQNP
RHIEFQVLADKYGNVVHFGERDCSIQRRNQKLLEEAPSPALTPELRKAMGDAAVAAAASIGYIGVGTVEF
LLDERGSFYFMEMNTRIQVEHPVTEMISSVDLIEEQIRVAMGEKLRYKQEDIVLRGHSIECRINAEDAFK
GFRPGPGRITAYLPSGGPFVRMDSHVYPDYVVPPSYDSLLGKLIVWAPTREKAIERMKRALDDTIITGVP
TTIDYHKLILDIEDFKNGKVDTAFIPKHEEELAMPPVKMVLAKEFAGVNA
>AhaccC4 Biotin carboxylase 4 [*Arachis hypogaea*] ACO53619.1
MEATMAACNSLSSPSVPIPGLYAGTSRGIKNSQCSFLGATKVNFPSQTMSRTCQLNHKHKTRSGALHATC
QGDKILVANRGEIAVRVIRTAHELGIPCVAVYSTIDKDALHVKLADESVCIGEAPSSQSYLLIPNVLSAA
ISRRCTMLHPGYGFLAENAVFVEMCREHGINFIGPNPDSIRVMGDKSTARDTMKNAGVPTVPGSDGLLHS
TEEAIRLANEIGFPVMIKATAGGGGRGMRLAKEPGEFVKLLQQAKSEAAAAFGNDGVYLEKYIQNPRHIE

FQVLADKYGNVVHFGERDCSIQRRNQKLLEEAPSPALTPELRKAMGDAAVAAAASIGYIGVGTVEFLLDE
RGSFYFMEMNTRIQVEHPVTEMISSVDLIEEQIRVAMGAKLRYKQEDIILRGHSIECRINAEDAFKGFRP
GPGRITAYLPSGGPFVRMDSHVYPDYVVPPSYDSLLGKLIVWAPTREKAIERMKRALDDTIITGVPTTIE
YHKLILDIEDFRNGKVDTAFIPKHEEELTMPPQKMVPAINKAKEFVGATV
>AhaccC3 Biotin carboxylase 3 [*Arachis hypogaea*] ACO53618.1
MGATMAACNSLSSPSVPIPGLYAGTSRGIKNSQCSFLGATKVNFPSQTMSRTCQLNHKHKTRSGALHATC
QGDKILVANRGEIAVRVIRTAHELGIPCVAVYSTIDKDALHVKLADESVCIGEAPSSQSYLLIPNVLSAA
ISRRCTMLHPGYGFLAENAVFVEMCREHGINFIGPNPDSIWVMGDKSTARDTMKNAGVPTVPGSDGLLQS
TEEAIRLANEIGFPVMIKATAGGGGRGMRLAKEPGEFVKLLQQAKSEAAAAFGNDGVYLEKYIQNPRHIE
FQVLADKYGNVVHFGERDCSIQRRNQKLLEEAPSPALTPELRKAMGDAAVAAAASIGYIGVGTVEFLLDE
RGSFYFMEMNTRIQVEHPVTEMISSVDLIEEQIRVAMGAKLRYKQEDIILRGHSIECRINAEDAFKGFRP
GPGRITAYLPSGGPFVRMDSHVYPDYVVPPSYDSLLGKLIVWAPTREKAIERMKRALDDTIITGVPTTIE
YHKLILDIEDFRNGKVDTAFIPKHEEELTMPPQKMVPAINKAKEFVGATV
>BnaccC Biotin carboxylase [*Brassica napus*] ADI79335.1
MDASMITNPKSIVSLPSLFMGRSGGSIRSSQCNVTMGQAVSFPSQKTKTLKVSRNVKRRSGGGGAFAATC
SSGDKILVANRGEIAVRVIRTAHEMGIPCVAVYSTIDKDALHVKLADEAVCIGEAPSNQSYLLIPNVLSA
AISRGCTMLHPGYGFLAENALFVEMCREHRINFIGPNPDSIRVMGDKSTARETMKNAGVPTVPGSDGLLQ
STEEGVRLANEIGYPVMIKATAGGGGRGMRLANEPSEFVKLLQQAKSEAAAAFGNDGVYLEKYVQNPRHI
EFQILADKFGNVVHFGERDCSIQRRNQKLLEEAPSPALTPELRKAMGDAAVAAAASIGYIGVGTVEFLLD
ERGSFYFMEMNTRIQVEHPVTEMIYSVDLIEEQIRVAMGEKLRYTQDEIVLRGHSIECRINAEDPFKGFR
PGPGRITSYLPSGGPFVRMDSHVYPDYVAPPSYDSLLGKLIVWAPTRERAIERMKRALNDTIITGVPTTI
EYHKLILEVEDFKNGKVDTAFIPKHEEELAEPHEIVPVKDLTNVAA
>HaaccC Biotin carboxylase 2 [*Helianthus annuus*] XP_021986207.1
MDSAAVTFCSKPITSPPGVFIGRSSGIKSSQCNFLVGNKLNFPKQRFQASEKAHRSAKREGALGVTCRAE
KILVANRGEIAVRVIRTAHELGIPCVAVYSTIDKDALHVKLADESVCIGEAPSNQSYLLIPNVLSAAISR
GCTMLHPGYGFLAENAVFVEMCREHGINFIGPNPDSIRVMGDKATARDTMKNAGVPTVPGSDGLLQSTEE
GIRLADEIGFPVMIKATAGGGGRGMRLAKERDEFVKLLQQAKSEAAAAFGNDGVYLEKCILNPRHIEFQV
LADKYGNVVHFGERDCSIQRRNQKLLEEAPSPALTPELRKAMGDAAVAAAASIGYIGVGTIEFLLDERGD
FYFMEMNTRIQVEHPVTEMISSVDLIEEQIRVAMGEKLRYTQDDIVLRGHSIECRINAEDAFKNFRPGPG
RITSYLPSGGPFVRMDSHVYTDYVVPPSYDSLLGKLIVWAPTREKAIERMKRALDDTIITGVPTTIEYHK
LILDIEDFKNGKVDTAFIPKHEDELAEPQKPSTSNAAKEVAKAVA
>At*accA* Carboxyltransferase alpha subunit [*Arabidopsis thaliana*] BAD93727.1
MASISHSSLALGGASSASASDYLRSSSNGVNGVPLKTLGRAVFTTIRRKDLAVTSRLKKGKKFEHPWPAN
PDPNVKGGVLSYLAEFKPLGDTQKPVTLDFEKPLVELEKKIVDVRKMANETGLDFTEQIITLENKYRQAL
KDLYTHLTPIQRVNIARHPNRPTFLDHIHNITDKFMELHGDRAGYDDPAIVTGIGTIDGKRYIFIGHQKG
RNTKENIMRNFGMPTPHGYRKALRMMYYADHHGFPIVTFIDTPGAYADLKSEELGQGEAIANNLRTMFGL
KVPILSIVIGEGGSGGALAIGCANKMLMLENAVFYVASPEACAAILWKTSKAAPEAAEKLRITSKELVKL
NVADGIIPEPLGGAHADPSWTSQQIKIAINENMNEFGKMSGEELLKHRMAKYRKIGVFIEGEPIEPSRKI
NMKKREAVFSDSRKLQGEVDKLKEQILKAKETSTEAEPSSEVLNEMIEKLKSEIDDEYTEAAIAVGLEER
LTAMREEFSKASSEEHLMHPVLIEKIEKLKEEFNTRLTDAPNYESLKSKLNMLRDFSRAKAASEATSLKK
EINKRFQEAVDRPEIREKVEAIKAEVASSGASSFDELPDALKEKVLKTKGEVEAEMAGVLKSMGLELDAV
KQNQKDTAEQIYAANENLQEKLEKLNQEITSKIEEVVRTPEIKSMVELLKVETAKASKTPGVTEAYQKIE
ALEQQIKQKIAEALNTSGLQEKQDELEKELAAARELAAEESDGSVKEDDDDDEDSSESGKSEMVNPSFA
>Bn*accA* Carboxyltransferase alpha subunit isoform 2 [*Brassica napus*] ACN65504.1
MASMSHSSIALGGASASASDYLRSSANGVTGVSLRALGRRTMVTATTRSSNLYVTARLKKGKKFDHPWPA
NPDPNVKGGVLSYLSEFKPLGNAQKPVTLDFERPLVELEKKIVDVRKMAEETGLDFTEQIITLETKYRQA
LKDLYTHLTPIQRVNIARHPNRPTFLDHIHNITDKFMELHGDRAGYDDPAIVTGIGTIDGKRYMFIGHQK
GRNTKENIMRNFGMPTPHGYRKALRMMYYADHHGFPIVTFIDTPGAYADLKSEEKGQGEAIANNLRTMFG
LKVPILSIVIGEGGSGGALAIGCANKMLMLENAVFYVASPEACAAILWQTSKAAPEAAEKLRIASKELVN
LNVADGIIPEPLGGAHADPSWTSQQIKIAINENMNEFGKMSGEELLKHRMAKYRKIGVFIEGEPVEPEKK
INMKRRDAVASTSRNLEGEVEKLREQILKAKEASPESDEGEELSSQVLKEMIEKLKSDIDEEYTKAAKAM
GLEERLAATREEFSKANAEEHLMHPVLIERIEKLKEEFNARLSEAPNYESLKAKLDMLKDFSRAKAASDA
ASVKKEINKRFQEAVERPEVREKVEAVKAEVASSGASSFEELSDELKEKVLKTKREVEAEMAVVLKSMGL
ELEAVKPNLKEVVAESPNENIQEKIEKLNREITEKIEEVVRTPEIKSMVELLKVETAKASQMPDQGSQKI

EALEMQIKQKIADALSMSGLQEKQEELEKELAAARELAGEESDESVKDDDDDEDGSGSGRSEIINPHFA
>Gm*accA-1* Carboxyl transferase alpha subunit [*Glycine max*] AAF80496.1
MAASSASLSGASASDLLRSSTSGFNGVPLRTMGKGKLVLKRRNFTVAARLRKVKKHEYPWPPNPDPNVKG
GVLSHLSLFKPLKEKPKPVTLDFERPLVDLQKKIIDVQKMANETGLDFSDQILSLETKYHQALKDLYTHL
TPIQRVNIARHPNRPTFLDHVFNITEKFVELHGDRAGYDDPAIVTGLGTIDGRSYMFIGHQKGRNTKENI
QRNFGMPTPHGYRKALRLMEYADHHGFPIVTFIDTPGAYADLKSEELGQGEAIAHNLRSMFGLKVPVISI
VIGEGGSGGALAIGCANKLLMLENAVFYVASPEACAAILWKTAKASPKAAEKLKITATELCKLQIADGVI
PEPLGGAHADPEWTSQQIKKAIKETMDELTKMNTEELLKHRMLKFRKIGGFQEGIPIDPKRKANMKKRDL
SIAKIPDAELEVEVEKLKQQVLEAKESSPVPPKLDLDEMLKQLAREVDLEYSEAVKATGLTDSLLKLREE
VSKANADNQIVDPLLEGKIEKLRVEFEQQLRAAPNYGRLQNKLNYLSELCKVKLLSDGKKDNEAVTFKQE
LKKKIDNALSDPKIRETFEALKAEIKGVGASSASDLDDELKKKIIEFIKEVKEVKEVKEVIENQIESLVN
SSDDIKSKILQLKLEVPKAGETPDSEPKNRIGALVQLIKPSLVEAVDSSGLKDLFENLVSNDGSLTHGDP
ARDSLTDDQ
>Gm*accA-3* Carboxyl transferase alpha subunit [*Glycine max*] AAF89549.1
MAASSASLSGASASDLLRSSTSGFNGVPLRTLGKGKLVLKRRDFTVAAKLRKVKKHEYPWPANPDPNVKG
GVLSHLSLFKPLKEKPKPVTLDFEKPLVDLQKKIIDVQKMANETGLDFSDQILSLENKYQQALKDLYTHL
TPIQRVNIARHPNRPTFLDHVFNITEKFVELHGDRAGYDDPAIVTGLGTIDGRSYMFIGHQKGRNTKENI
QRNFGMPTPHGYRKALRLMEYADHHGFPIVTFIDTPGAYADLKSEELGQGEAIAHNLRSMFGLKVPVISI
VIGEGGSGGALAIGCANKLLMLENAVFYVASPEACAAILWKTAKASPKAAEKLKITATELCKLQIADGVI
PEPLGGAHADPEWTSQQIKKAIKETMDELTKMNTEELLKHRMLKFRKIGGFQEGIPIDPKRKANMKKRDL
SIAKIPDAELEVEVEKLKQQVLEAKESSPVPPKLDLDEMLKQLAREVGLEYSEAVKATGLTDSLLKLREE
VSKANADSQIVDPLLKDKIEKLRVEFEQQLRAAPNYGRLQNKFKYLSELCKVKLLSDANKDNQAVTFKQE
LEKKVDNALNDPKIRETFEALKAEIKGAGASSASDLDDELKKKIVGFMIELKEVKEVKEVIENQIESLVN
SSDDIKSKILQLKLELPKAGETPDSESNNRIGDFIFKKSSKIIMAILLRDMFGNFKEIPC
>AhaccA-1 Acetyl-coenzyme A carboxylase carboxyl transferase subunit alpha, [*Arachis hypogaea*] XP_025695444.1
MASSSAAASLAGGSASDLLRGSTSGFSGVPLRTLGRARLPLKQRDFSVSCKMRKVKKHEHPWPDNPDPNV
KGGVLSHLSPFKPLKEKPKPVTLDFEKPLIALQKKIIDVRKMANETGLDFSDQILSLETKYQQALKDLYT
HLTPIQRVNIARHPNRPTFLDHIYNITDKFVELHGDRAGYDDPAIVTGIGTIDGRRYMFIGQQKGRNTKE
NIQRNFGMPTPHGYRKALRLMEYADHHGFPIVTFIDTPGAYADLKSEELGQGEAIAHNLRSMFGLKVPVI
SIVIGEGGSGGALAIGCGNKLLMLENAVFYVASPEACAAILWKSAKAAPKAAEKLRITASELCRLEIADG
VIPEPLGGAHADPSWTSQQIKKAVNEAMDELTKMNTEELLRHRMLKFRKIGGFQEGIPVEPKKKINMKKK
DIPIANKISDAELEVEVEKLKQQILDSKESSVEPRLDLDDMIKQLQIEVDQEYSEAVNAIGLSDRMSKLK
EEVVKANTDNQFIDPLLKSKIEKLKEEFDQKLSTAPNFGRLENKVNMLKELSKVKRLQDQNKRTSALEQE
LKTKFDGIMKNPRIKEKYEALKSEIQAAGASSSRDLDDDLKQKIVEFNKEFDSLLAESLKSAGMEVKIAP
ARPRDSSGESAELGYESKIEELREGISKEIEKLANSSNIKSKIELLKLEVAKAGETPDTESKNRIAALVQ
QIKQSLEEAVDSSSLKEEYENLVSKISSRDSEVEDGLTNDQLREKVGANRSFS
>Ah*accA*-2 Acetyl-coenzyme A carboxylase carboxyl transferase subunit alpha [*Arachis hypogaea*] XP_025649321.1
MNTMSLTRGNYYMDGRLIIEEHGIESANQCVGKDFLGGSRAICIWPKYLTSSRNNTNTNPNNNGKWQRFN
VAAKIRKGKKHDYPWPDKMDPNISSGYLTYLSHFKPLAEKPKPVTLDFEKPLVDLEKKIIEVRSMADDTG
LDFSNQIEALESKYQQALKDLYTHLTPFQRLMIARHPNRPTVLDHILNITEKWVELHGDRAGYDDPAIVT
GLGTMDGKSYMFIGHQKGRNTKENITRNFAMPTPHGYRKALRMMKYADHHKFPIITFVDTPGAYADLKSE
ELGQGEAIAHNLRTMFGLKVPILTVVTGEGGSGGALAIACANKLFMLENSAFYVASPEACAAILWKSSKA
APKAAEKLRITAQEHYRLGIADGVIPEPLGGAHVDPTWTSQQIKLTLTQAMEELTKMNEEELFRHRHLKF
RSIGGFQEGIPVEPERKRNMKPSDVNSSTLTDIESELQTLRKQILESKGPTDPITNESIQKLVKEVDEEI
TKAIISMGLAEKVQSVRMELSKNSNQPLSTNMEEKVDRIMEEINMKMAQPGAYLGLKQKLKKLDTINSFI
EMKVKQEKLRNELNEKLSADTKAKIASLMDAQERMPDHELVEKAMEVQRELEEVLKSANLEIVGVMKKNV
ETPPADIKQKIVELNNEIIGEIDRVVNAEEGLKDQIKELENMIAQGLDSKDVEKMEAGIKERILAALDAA
GVKEKIERMKEEVESLSMAGFEDKIGEENGRC
>BnaccA2 Carboxyltransferase alpha subunit [*Brassica napus*] ACT83681.1
MASMSHSSIALGGASASASDYLRSSANGVTGVSLRALGRRTMVTATTRSSNNLYVTARLKKGKKFDHPWP
ANPDPNVKGGVLSYLSDFKPLGNAQKPVTLDFERPLVELEKKIVDVRKMAEETGLDFTEQIITLETKYRQ
ALKDLYTHLTPIQRVNIARHPNRPTFLDHIHNITDKFMELHGDRAGYDDPAIVTGIGTIDGKRYMFIGHQ
KGRNTKENIMRNFGMPTPHGYRKALRMMYYADHHGFPIVTFIDTPGAYADLKSEEKGQGEAIANNLRTML
GLKVPILSIVIGEGGSGGALAIGRANKMLMLENAVFYVASPEACAAILWQTSKAAPEAAEKLRITSKELV

NLNVADGIIPEPLGGAHADPSWTSQQIKIAINENMNEFGKMSGEELLKHRMAKYRKIGAFIEGEPVEPEK
KINMKRRDAVASTSRNLEGEVEKLREQILKAKEASPESEVELSSEVLNEMIEKLKSDIDEEYTKAAKAMG
LEERLAATREEFSKANAEEHLMHPVLIERIEKLKEEFNARLSEAPNYESLKAKLDMLKDFSRAKAASEAA
SVKKEIDKRFREAVERPEVREKVEAVKAEVASSGASSFEELSDELKEKVLKTKREAEAEMAVVLKSMGLG
LEAVKPNLKEVVAESPNENIQEKLEKLNREINEKIEEVVRTPEIKSMVELLKVETAKASRMPDQGSQKIE
ALEMQIKQKIADALSMSGLQEKQEELEKELAAARELAGEESDESVKEDDDDDEDGSGSGRSEIINPHFA
>Ha*accA-1* Acetyl-coenzyme A carboxylase carboxyl transferase subunit alpha [*Helianthus annuus*] QGZ98789.1
MASVSQSPVNFSGSLSSATSASDLLRSSINGVNGVPLQTLGKGHVGPRRKGLNIVAKVRKVKKHEYPWPQ
DPDPNVKGGVLSHLSPFKPLKEKPKPVTLEFEKPLMDLQKKIIDVQKMANETGLDFSDQIISLENKYQQA
LKDLYTHLTPIQRVNIARHPNRPTFLDHVFNITDKFVELHGDRAGYDDPAVVTGIGTIDGRRFMFMGHQK
GRNTKENIQRNFGMPTPHGYRKALRMMYYADHHGFPIVTFIDTPGAFADLKSEELGQGEAIAHNLRTMFG
LKVPIVSIVMGEGGSGGALAIGCANKLLMLENAVFYVASPEACAAILWKTAKASPKAAEKLKITANELCK
LQIADGIIPEPLGGAHADPHWTSEQIKKAILESMDELQTMGTEELLRHRMLKFRKIGGFQEGLPVEPEKK
VNMKRKEEPRPGLISDKVLQGEVNKLKEQIMKAKESSSIDMDQNGLIEKLQREINYEFSEAAKALGIEEK
LSQMREEFAKVENGQLTSGQIENLERLKNEFNEKLETAPNYGRLKYKLSLLKEITEAKVFAENYKKTLPL
KAKVNEKFKEVLEKSTLKQKIENLKAEVEKSDVSSIEEFDQDLKEKVLEVREEVESEFINALESSGVHVT
SKIGQSSMSLVKAKVDELNAEINQIIQDVIESTPDLKSKIEMLKLEVVKDKNTPSKESKEKIEALQQQIK
EGVADVMSSAAIKEKYEQLQAELAELSGGSDGSLIKESSYA
>Ha*accA-2* Acetyl-coenzyme A carboxylase carboxyl transferase subunit alpha, [*Helianthus annuus*] XP_022010319.1
MASVSQSPVTFSGSLSSATSASDLLRSSMNGVNGVPLKTLGKGHVGHGRKGLNIVAKVRKVKKHEYPWPQ
DPDPNVKGGVLSHLSPFKPLKEKPKPVTLEFEKPLMDLQKKIIDVQKMANETGLDFSDQIISLENKYQQA
LKDLYTHLTPIQRVNIARHPNRPTFLDHVFNITDKFVELHGDRAGYDDPAVVTGIGTIDGRRFMFMGHQK
GRNTKENIQRNFGMPTPHGYRKALRMMYYADHHGFPIVTFIDTPGAFADLKSEELGQGEAIAHNLRTMFG
LKVPIVSIVMGEGGSGGALAIGCANKLLMLENAVFYVASPEACAAILWKTAKASPKAAEKLKITANELCK
LQIADGIIPEPLGGAHADPQWTSMQIKNAILDSMDELEKMGTEELLRHRMLKFRKIGGFQEGLPVDPEKK
VNMKRKEEPRPGLISDKALQGEVNKLKEQILKAKETSSINMDQNGLIEKLQREINYEFSEAAKALGIEEK
LSKMREEFAKAENEQLTPGQIKNLENLKNEFNENLATAPNYGRLKYKLSLLKEITEAKTFAENYKKALPL
KAKVNEKFKEVLEKPTLKQNIENIKAEIEKSGVSNIEEFDQELKEKVLEVREQVESEFVNALESSGVHVT
SKVDQTSMSMIKAKVDELNAEINQIIQDVIESTPDLKNKIEILKLEIVKDKNAPSKESKEKIESLQQQIK
QGVGDVMSYAAVKEKYEELQAELAELSGVGSDGSLIKEPIYA

>AtaccD Acetyl-CoA carboxylase beta subunit [*Arabidopsis thaliana*] NP_051068.1
MEKSWFNFMFSKGELEYRGELSKAMDSFAPGEKTTISQDRFIYDMDKNFYGWDERSSYSSSYSNNVDLLV
SSKDIRNFISDDTFFVRDSNKNSYSIFFDKKKKIFEIDNDFSDLEKFFYSYCSSSYLNNRSKGDNDLHYD
PYIKDTKYNCTNHINSCIDSYFRSYICIDNNFLIDSNNFNESYIYNFICSESGKIRESKNYKIRTNRNRS
NLISSKDFDITQNYNQLWIQCDNCYGLMYKKVKMNVCEQCGHYLKMSSSERIELSIDPGTWNPMDEDMVS
ADPIKFHSKEEPYKNRIDSAQKTTGLTDAVQTGTGQLNGIPVALGVMDFRFMGGSMGSVVGEKITRLIEY
ATNQCLPLILVCSSGGARMQEGSLSLMQMAKISSVLCDYQSSKKLFYISILTSPTTGGVTASFGMLGDII
IAEPYAYIAFAGKRVIEQTLKKAVPEGSQAAESLLRKGLLDAIVPRNLLKGVLSELFQLHAFFPLNTN
>GmaccD Beta-carboxyltransferase subunit [*Glycine max*] AAA80643.1
MEKWWFNSMLFNRKLEYRCELSKSMDSLGPIENTSLREDPKILTDIEKKIHRDLDYLEMEGFFSSDLNTV
SKNDDDHYMYETQFSFNNNITSFIDSCIESFNLGDIDKYNDIYFNSYIFLKGRNCSESNNSSTSIITSTN
DTNDSDSTIGESSNNLDESQKYKHLWLECENCYGLNYKKFFKSKMNICEYCGYHLKMGSSDRIELSIDSG
TWNPMDEDMVSLDPIEFHSEEEPYKDRIDSYQRKTGLTEAVQTGTGQLNGIPVAIGIMDFQFMGGSMGSA
VGEKITRLVEYATNQLLPLILVCASGGARMQEGSLSLIQMAKISSALYDYQKNKKLFYVSILTSPTTGGV
TASFGMLGDIIIAEPNAYIAFAGKRVIEQTLNKAVPEGSQAAEYLFHKGLFDSIVPRNLLKGVLSELFQF
HNSFSLTKNDKA
>AhaccD Beta-carboxyltransferase 1 [*Arachis hypogaea*] ACO53622.1
MEKGWFNSMLFYRQLEYRCGLSNSMDSFGPVENTSASEDPILIDMEKDFPSWNDSDNSSYSNVDYLVGVR
NIRNFLSDKILLVRDNNSQRNRYSIYFDIENQFLEISNDPSFLSEPESLFDSYNKNSSYLNNVSKRHENH
YMYDTKSSWKNGIHNCIESYLRSQICIVSHILGESDKYNDSYFYTSICGKGGNSSESEGSSIKTTITNEN
LTKREDSKDLDETQKYKHLWIECENCYGLNYRKFFKSKMNICEHCGYHLKMSSSDRIELLIDPGTWNPMD
EDMVSMDPIEFHSEEESESYKNRMDSYQRKTGLTEAVQTGTGQLNGIPVAIGIMDFQFMGGSMGSVVGEK

ITRLVEHAGNQLLPLILVCASGGARMQEGSLSLMQMAKISSALYEYQKNKRLFYVSILTSPTTGGVTASF
GMLGDIIIAEPDAYIAFAGKRVIEQTLNTTIPEGSQVAEYLFQKGLFDSIVPRNLLKGVLSELFQLHAFF
PL
>BnaccD Acetyl CoA carboxylase carboxyltransferase (beta subunit) [*Brassica napus*] CAA90747.1
MEKSWFNLMFSKGELEYRGELSKAMDSFAPIEKTTISKDRFIYDMDKNFYGWGERSSYYNNVDLLVNSKD
IRNFISDDTFFVRDSNKNSYSIYFDIEKKKFEINNDLSDLEIFFYSYCSSSYLNNRSKGDNDLHYDPYIK
DTKYNCNNHINSCIDSYFRSHICINSHFLSDSNNSNESYIYNFICSESGSGKIRESKNDKIRTNSNRNNL
MSSKDFDITKNYNQLWIQCDNCYGLKYKKVEMNVCEECGHYLKMTSSERIELSIDPGSWNGMDEDMVSAD
PIKFHSREEPYKKRIASAQKKTGLTDAIQTGTGQLNGIPVALGVMDFQFMGGSMGSVVGDKITRLIEYAT
NQCLPLILVCSSGGARMQEGSLSLMQMAKISSVLCDYQSSKKLFYISILTSPTTGGVTASLGMLGDIIIA
EPYAYIAFAGKRVIEQTLKKAVPEGSQAAESLLRKGLLDAIVPRNPLKGVVSELFQLHAFFPLNKNEIK
>*Nt*accD Acetyl-CoA carboxylase beta subunit [*Nicotiana tabacum*]
MTIHLLYFHANRGQENSMERWWFNSMLFKKEFERRCGLNKSMGSLGPIENTNEDPNRKVKNIHSWRNRDN
SSCSNVDYLFGVKDIRNFISDDTFLVSDRNGDSYSIYFDIENHIFEIDNDHSFLSELESSFYSYRNSNYR
NNGFRGEDPYYNSYMYDTQYSWNNHINSCIDSYLQSQICIDTSIISGSENYGDSYIYRAVCGGESRNSSE
NEGSSRRTRTKGSDLTIRESSNDLEVTQKYRHLWVQCENCYGLNYKKFLKSKMNICEQCGYHLKMSSSDR
IELLIDPGTWDPMDEDMVSLDPIEFHSEEEPYKDRIDSYQRKTGLTEAVQTGIGQLNGIPVAIGVMDFQF
MGGSMGSVVGEKITRLIEYAANQILPLIIVCASGGARMQEGSLSLMQMAKISSALYDYQLNKKLFYVSIL
TSPTTGGVTASFGMLGDIIIAEPNAYIAFAGKRVIEQTLNKTVPEGSQAAEYLFQKGLFDLIVPRNLLKS
VLSELFKLHAFFPLNQKSSKIK

>AhFabD1-1 Malonyl-CoA:ACP transacylase 1-1 [*Arachis hypogaea*] ACJ07137.1
MQALLHHSPLIRTSHSSFPAMASSSLTLPSLSLNNFPSSPSNAHSRTFNLPLLRSRVFMSVASGSQVSVL
NDALFSDYKPSNAFLFPGQGAQAVGMGKEAQNVPAAAVLYKKANEILGYDLLDICINGPKDKLDSTVLSQ
PAIYVTSLAAVELLRARDGGQQIIDSVDVTCGLSLGEYTALAFAGAFSFEDGLKLVKLRGEAMQDAADAA
KSAMVSVIGLDSEKVQQLCDAANQEVPEAEKVQIANYLCPGNYAVSGGLKGVEVLESKAKSFKARMTVRL
AVAGAFHTSFMEPAVSRLEAALAATEIRTPRIPVISNVDAQPHADPDTIKKILARQVTSPVQWETTVKTL
LNKGLKKSYELGPGKVIAGIIKRVDKGADIENIGA
>AhFabD1-2 Malonyl-CoA:ACP transacylase 1-2 [*Arachis hypogaea*] ACJ07138.1
MQALLHHSPLIRTSHSSFPAMASSSLTLPSLSLNNFPSSPSNAHSRTFNLPLLRSRVFMSVASGSQVSVL
NDALFSDYKPSNAFLFPGQGAQAVGMGKEAQNVPAAAVLYKKANEILGYDLLDICINGPKDKLDSTVLSQ
PAIYVTSLAAVELLRARDGGQQIIDSVDVTCGLSLGEYTALAFAGAFSFEDGLKLVKLRGEAMQDAADAA
KSAMVSVIGLDSEKVQQLCDAANQEVPEAEKVQIANYLCPGNYAVSGGLKGVEVLESKAKSFKARMTVRL
AVAGAFHTSFMEPAVSRLEAALAATEIRTPRIPVISNVDAQPHADPDTIKKILARQVTSPVQWETTVKTL
LNKGLKKSYELGPGKVIAGIIKRVDKGADIENIGA
>GmFabD Malonyltransferase [*Glycine max*] NP_001238312.2
MSSSLALPSTPFFANGTRIFALKHSLPRSRVSMSVSAGSQASVHQALFADYKASTSFLFPGQGAQAVGMG
KEAQNVPAAAVLFNKANDILGFDLLDICINGPKEKLDSTVISQPAIYVTSLAAIELLRAREGGQQIIDSV
DVTCGLSLGEYTALAFAGAFSFEDGLKLVKLRGEAMQDAADAARSAMVSVIGLDSEKVQQLCDAANQEVP
EAEKVQIANYLCPGNYAVSGGLKGVEVLESKAKSFKARMTVRLAVAGAFHTSFMEPAVSRLEAALATTEI
RTPRIPVISNVNAQPHTDPNTIKKILARQVTSPVQWETTVKTLLTKGLKKSYELGPGKVIAGIVKRMDKG
ADIENIGA
>AtFabD Malonyltransferase [*Arabidopsis thaliana*] CAA0373481.1
MRSLLHRTILLTSPSHSLIRRTSLSAMATTASSSLLLPSISLNNLSSSKNASFGFAAKNLSRSRISMSVS
AGSQSTTVHDSLFADYKPTSAFLFPGQGAQAVGMGKEAQSVGAAGELYKKANDILGYDLLDICVNGPKEK
LDSTVISQPAIYVTSLAAVELLRVREGGEQIINSVDVTCGLSLGEYTALAFAGAFSFEDGLKLVKLRGEA
MQAAADAAKSAMVSIIGLDSEKVQQLCDAANQEVDEADKVQIANYLCPGNYAVSGGLKGIEVVEAKAKSF
KARMTVRLAVAGAFHTSFMEPAVSRLEAALAATEIRSPRIPVISNVDAQPHADPDTIKKILARQVTSPVQ
WETTVKTLLSKGLKSSYELGPGKVIAGIFKRVDKSASFENISA

>AhFabH 3-oxoacyl-[acyl-carrier-protein] synthase 3 A, [*Arachis hypogaea*] XP_025611200.1
MANASAFFTPSVPKFGETVPPLNLIAIHRFQRFSAKVVCFGTIEGAGKHASAASPSQSQLPRLVGKGCKL

VGCGSAVPTLQISNDDLSKMVDTSDEWISVRTGIRRRRVLSGRDNLIALGVDASRKALEMANVDPDDLDL
ILMCTSTPEDLFGSAPQIQKQLGCKANPLAYDITAACSGFVLGLISAACHIRGGGFRNVLVIGADALSRY
VDWTDRGSCILFGDAAGAVLVQACDTEEDGLFGFDLHSDGSGQRHLNASIKEHETNTALDSNGSVLDFPP
RKSSYSFIQMNGKEVFRFAVRCVPQSIESALQKAGLPASSIDWLLLHQANQRIIDAVAARLEVPSERVIS
NLANYGNTSAASIPLALDEAVRSGKVKAGQTIAAAGFGAGLTWGSAIIRWG
>GmFabH Beta-ketoacyl-acyl carrier protein synthase III [*Glycine max*] NP_001237735.1
MANASGFFSPSVPHFKVRVKPLTTAIGFSAKVVCVGNIEGAEKHASTVSPSSQSPIPRLVSKGCKLVGCG
SAVPALQISNDDLSKMVDTNDEWISVRTGIRRRRVLSGKDNLTGLAVEAARKALEMAKVDPDDLDLILMC
TSTPEDLFGSAPQIQKQLGCKANPLSYDITAACSGFVLGLISAACHIRGGGFNNVLVIGADALSRYVDWT
DRGTCILFGDAAGAVLVQACNSEEDGLFGFDLHSDGSGQRHLNASIKENESNNALDSNGSVFGFPPKQSS
YSCIQMNGKEVFRFAVRCVPQSIESALEKAGLPASSIDWLLLHQANQRIIDAVATRLELPSERVISNLAN
YGNTSAASIPLALDEAVRSGKVKAGQTIATAGFGAGLTWGSAIIRWG
>BnFabH 3-oxoacyl-[acyl-carrier-protein] synthase III,[*Brassica napus*] XP_013732147.1
MANASGFFAHPSISSMRSRTHLPIAVSGSGFCVSKRFSKRVLCSSLSSLDNNASPSPSQYRPPRLVPSGC
KLIGSGSAVPSLLISNDDLAKIVDTNDEWIATRTGIRNRRVVSGKDSLVGLAVEAATKALEMAEVAPEDI
DLVLMCTSTPDDLFGAAPQIQKALGCTKNPLAYDITAACSGFVLGLVSAACHIRGGGFKNVLVIGADSLS
RFVDWTDRGTCILFGDAAGAVVVQACDIEDDGLYSFDVHSDGDGRRHLNASVKESQTDGALSSNGSALGD
FPPKQSSYSCIQMNGQEVFRFAVRCVPQSLETALQKAGLPASSIDWLLLHQANQRILDSVATRLQFPPER
VISNLANYGNTSAASIPLALDEAVRSGKVKPGHTIATSGFGAGLTWGSAIVRWRTSAASIPLALDEAVRSG
KVKPGHTIATSGFGAGLTWGSAIMRWR
>AtFabH-1 3-ketoacyl-acyl carrier protein synthase III, [*Arabidopsis thaliana*] OAP12004.1
MANASGFFTHPSIPNLRSRIHVPVRVSGSGFCVSNRFSKRVLCSSVSSVDKDASSSPSQYQRPRLVPSGC
KLIGCGSAVPSLLISNDDLAKIVDTNDEWIATRTGIRNRRVVSGKDSLVGLAVEAATKALEMAEVVPEDI
DLVLMCTSTPDDLFGAAPQIQKALGCTKNPLAYDITAACSGFVLGLVSAACHIRGGGFKNVLVIGADSLS
RFVDWTDRGTCILFGDAAGAVVVQACDIEDDGLFSFDVHSDGDGRRHLNASVKESENDGESSSNGSVFGD
FPPKQSSYSCIQMNGKEVFRFAVKCVPQSIESALQKAGLPASAIDWLLLHQANQRIIDSVATRLHFPPER
VISNLANYGNTSAASIPLALDEAVRSGKVKPGHTIATSGFGAGLTWGSAIVRWR
>AtFabH-2 3-ketoacyl-acyl carrier protein synthase III [*Arabidopsis thaliana*] CAA72385.1
MANASGFFTHPSIPNLRSRIHVPVRVSGSGFCVSNRFSKRVLCSSVSSVDKDASSSPSQYQRPRLVPSGC
KLIGCGSAVPSLLISNDDLAKIVDTNDEWIATRTGIRNRRVVSGKDSLVGLAVEAATKALEMAEVVPEDI
DLVLMCTSTPDDLFGAAPQIQKALGCTKNPLAYDITAACSGFVLGLVSAACHIRGGGFKNVLVIGADSLS
RFVDWTDRGTCILFGDAAGAVVVQACDIEDDGLFSFDVHSDGDGRRHLNASVKESQNDGESSSNGSVFGD
FPPKQSSYSCIQMNGKEVFRFAVKCVPQSIESALQKAGLPASAIDWLLLHQANQRIIDSVATRLHFPPER
VISNLANYGNTSAASIPLALDEAVRSGKVKPGHTIATSGFGAGLTWGSAIMRWR

>Gm*FabB-1* 3-oxoacyl-[acyl-carrier-protein] synthase I, [*Glycine max*] XP_006584769.1
MAGIAGTCPLGALLRNSVSENNGKISVVHYEGLRLQQRMQMPSPSQYISASSSAAPRCRTIKAMASPTVG
APKREKDPKKRVVITGMGLVSVFGSDVDAFYNKLLEGESGISLIDRFDASNFSVRFGGQIRDFSSEGYID
GKNDRRLDNCWRYCIVAGKRALDDANLGKQVLDTQMDKTRIGVLVGSGMGGITAFSNGVEALVQKGYKKI
TPFFIPYSITNMGSALLAIDTGLMGPNYSISTACATANYCFCAAANHIRKGEADIMVVGGTEAAIMPSGL
GGFIACRALSHRNEDPKKASRPWDKDRDGFVMGEGSGVLVMESLESATKRGAKIIAEYLGGAITCDAHHM
TDPRADGLGVSSCISKSLEDAGVSPEEVNYVNAHATSTLAGDLAEVNAIKKVFKDTSELKMNATKSMIGH
GLGAAGGLEAIATIKAITTGWLHPSINLDNLEASVTIDTVPNVKKKHEVNVGISNSFGFGGHNSVVVFAP
FRP
>GmFabB-2 Beta-ketoacyl-ACP synthetase I [*Glycine max*] NP_001238610.1
MQALHSPTLRASPLDPLRGPNNAAANRRSSSAKRVFFVSATVAPKVSAPQRQKDPKKRVVITGMGLASVF
GNDVEGYYEKLLAGESGITAIDRFDASKFPTRFGGQIRGFSAEGYIDGKNDRRLDDCLRYCIVAGKKALE
NADLAPDNHSKIDKERAGVLVGSGMGGLTVFSDGVQALIEKGHRKITPFFIPYAITNMGSALLGIDLGFM
GPNYSISTACATSNYCFYAAANHIRRGEADLMIAGGTEAAIIPIGLGGFVACRALSQRNDDPKTASRPWD
KERDGFVMGEGAGVLVMESLEHAMKRGAPIIAEYLGGAVNCDAYHMTDPRSDGLGVSTCIQSSLEDAGVS

PEEVNYINAHATSTLAGDLAEINAIKKVFKDTSGIKINATKSMIGHCLGAAGGLEAIATVKAITTGWLHP
TINQFNPEPAVDFDTVANVKQQHEINVAISNSFGFGGHNSVVAFSAFKP
>BnFabB 3-oxoacyl-[acyl-carrier-protein] synthase I [*Brassica napus*] XP_013662893.1
MQALQSSSLRASPPNPLIKPSNRQSHQITNARQPTRRRTFISASAAVSAPKRETDPKKRVVITGMGLVSV
FGNDVDAYYEKLLSGESGISLIDRFDASKFPTRFGGQIRGFSSEGYIDGKNERRLDDCLKYCIVAGKKAL
ESANLGGDKLNTIDKQRAGVLVGTGMGGLTVFSDGVQALIEKGHRRISPFFIPYAITNMGSALLAIDLGL
MGPNYSISTACATSNYCFYAAANHIRRGEADMMIAGGTEAAIIPIGLGGFVACRALSQRNDDPKTASRPW
DKQRDGFVMGEGAGVLVMESLEHAMKRGAPIVAEYLGGAVNCDAHHMTDPRADGLGVSSCIESCLEDAGV
SPEEVNYINAHATSTLAGDLAEINAIKKVFKSTAGIKINATKSMIGHCLGAAGGLEAIATVKAINTGWLH
PSINQFNPEPAVDFDTVANEKKQHEVNVAISNSFGFGGHNSVVAFSAFTP
>HaFabB-1 3-oxoacyl-[acyl-carrier-protein] synthase I [*Helianthus annuus*] XP_022025436.1
MQAQALLQSPHLRVSPLDPLRTRPKPTNFKPRTTVIASATAPKREKDPKKRVVITGMGLVSVFGNDVDTY
YEKLLAGESGIGLIDRFDASKFPTRFGGQIRGFKSDGYIDGKNDRRLDDCLRYCIVAGKKALEDADLGGD
KLSKIDKERAGVLVGTGMGGLTVFSDGVQNLIEKGYRKITPFFIPYAITNMGSALLAIDVGFMGPNYSIS
TACATSNYCFYAAANHIRRGEADMMIAGGTEAAIIPIGLGGFVACRALSQRNDDPQTASRPWDKDRDGFV
MGEGAGVLVMESLEHAMKRGAPIIAEYLGGAVNCDAYHMTDPRSDGLGVSSCIQSSLEDAGVSPEEVNYI
NAHATSTLVGDLAEVNAVKKVFKSTDGIKMNSTKSMIGHCLGAAGGLEAIATVKAIQTGWLHPTINQFNP
EPAVEFDTVANQKQQHEVNVAISNSFGFGGHNSVVAFSAFKP
>HaFabB-2 3-keto-acyl-ACP synthase I [*Helianthus annuus*] ABM53471.1
MQAQALLQSPHLRVSPLDPLRTRPKPTNFKPRTTVIASATAPKREKDPKKRVVITGMGLVSVFGNDVDTY
YEKLLAGESGIGLIDRFDASKFPTRFGGQIRGFKSDGYIDGKNDRRLDDCLRYCIVAGKKALEDADLGGD
KLSKIDKERAGVLVGTGMGGLTVFSDGVQNLIEKGYRKITPFFIPYAITNMGSALLAIDVGFMGPNYSIS
TACATSNYCFYAAANHIRRGEADMMIAGGTEAAIIPIGLGGFVACRALSQRNDDPQTASRPWDKDRDGFV
MGEGAGVLVMESLEHAMKRGAPIIAEYLGGAVNCDAYHMTDPRSDGLGVSSCIQSSLEDAGVSPEEVNYI
NAHATSTLVGDLAEVNAVKKVFKSTDGIKMNSTKSMIGHCLGAAGGLEAIATVKAIQTGWLHPTINQFNP
EPAVEFDTVANQKQQHEVNVAISNSFGFGGHNSVVAFSAFKP
>AhFabB 3-oxoacyl-[acyl-carrier-protein] synthase I [*Arachis hypogaea*] XP_025639780.1
MQAIHTPTLRVSPLDPLRKSTNPPISTRRASKRVSFTVSAAAVSPKVSAPQRQKDPKKRVVITGMGLASV
FGNDVDTFYDRLLAGESGITTIDRFDASKFPTRFGGQIRGFSSEGYIDGKNDRRLDDCLRYCIVAGKKAL
ENADLGGEKLNKLDKEKAGVLVGSGMGGLTVFSDGVQALIEKGHRKITPFFIPYAITNMGSALLGIDLGF
MGPNYSISTACATSNYCFYAAANHIRRGEADLMIAGGTEAAIIPIGLGGFVACRALSQRNDDPKTASRPW
DKDRDGFVMGEGSGVLVMESLEHAMRRGAPIIAEYLGGAVNCDAYHMTDPRSDGLGVSSCIQSSLEDAGV
SPEEVNYINCHATSTLAGDLAEINAIKKIFKKTDGIKINATKSMIGHCLGAAGGLEAIATVKAITTGWLH
PSINQFNPEPAVDFDTVANVKKQHEINVGISNSFGFGGHNSVVAFSAFKP
>NtFabB 3-ketoacyl-acyl-carrier protein synthase I [*Nicotiana tabacum*] ANT48295.1
MQSIQSPSFRPSPLDPLKKTVPQLTNVRPLSHAKRVPFISASATTVSAPKREKDPKKRVVITGMGLVSVF
GNDVDTYYDRLLAGESGISLIDRFDASKFPTRFGGQIRGFTSDGYIDGKNDRRLDDCLRYAIVAGKKALE
NADLGGDRLGKIDKERAGVLVGTGMGGLTVFSDGVQNLIEKGHRKITPFFIPYAITNMGSALLGIDLGFM
GPNYSISTACATSNYCFYAAANHIRRGEADLMLAGGTEAAIIPIGLEVLWHVXLLSQRNDDPQTASRPWD
KERDGFVMGEGSGVLVMESLEHAMKRGAPIIAEYLGGAVNCDAHHMTDPRADGLGVSSCIQSSLEDAGVS
PEEVNYINAHATSTIVGDIAEVNAIKKVFKNTSGIKMNATKSMIGHCLGAAGGMEAIATVKAITTGWVHP
SINQFNPEPSVEFDTVPNKKQQHEVNVGISNSFGFGGHNSVVAFSAFKP
>AtFabB-I 3-ketoacyl-acyl carrier protein synthase I [*Arabidopsis thaliana*] NP_199441.1
MQALQSSSLRASPPNPLRLPSNRQSHQLITNARPLRRQQRSFISASASTVSAPKRETDPKKRVVITGMGL
VSVFGNDVDAYYEKLLSGESGISLIDRFDASKFPTRFGGQIRGFSSEGYIDGKNERRLDDCLKYCIVAGK
KALESANLGGDKLNTIDKRKAGVLVGTGMGGLTVFSEGVQNLIEKGHRRISPFFIPYAITNMGSALLAID
LGLMGPNYSISTACATSNYCFYAAANHIRRGEADMMIAGGTEAAIIPIGLGGFVACRALSQRNDDPQTAS
RPWDKARDGFVMGEGAGVLVMESLEHAMKRGAPIVAEYLGGAVNCDAHHMTDPRADGLGVSSCIERCLED
AGVSPEEVNYINAHATSTLAGDLAEINAIKKVFKSTSGIKINATKSMIGHCLGAAGGLEAIATVKAINTG
WLHPSINQFNPEQAVDFDTVPNEKKQHEVDVAISNSFGFGGHNSVVAFSAFKP

>Ha*FabF-1* 3-oxoacyl-[acyl-carrier-protein] synthase II [*Helianthus annuus*] XP_022013627.1
MVASAVATWLVAACMSFSGDTRLKPSPPAALPLPSKRLSRSARRKLQAMTKCNHFSAAGGLMSSIYGSGI
MNLLEPCEEYYKSKPLSSSSLSIFGYENRRVKQTGKAMAIAVQPAKEPTTKKKPVTKKRRVVVTGLGVVS
PVGDVVDVFYENLLDGVSGISEIESFDCQHFPTKIAGEIKSFSADGWIAPKLSKRVDKFMLYLLTAGKKA
LEDGGVTDEVMKELDITKCGVIIGSALGGMKIFQDAIEALRVSYKKMNPFCVPFATTNMGSAILAMDLGW
MGPNYSISTACATSNYCILNAANHIIRGETDMMLSGGSDSAIIPIGLGGFVACNSLSQRNDDPTKASRPW
DVSRDGFVMGEGAGVLLLEELEHAKARGAKIYAEFMGGSFTCDAYHMTEPHPDGTGIALCIEKALAQSGV
AREDVNYINAHATSTPSGDLNEYEAIIRCFGDNSQLKINSTKSMVGHLLGAAGAVEAVATVKAIQTGWLH
PNINLENPDVGVDKNVLVGAKKERFDVKVALSNSFGFGGHNSSILFAPYKDQ
>Ha*FabF-2* 3-keto-acyl-ACP synthase II [*Helianthus annuus*] ABI18155.1
MAASMVCTWIVAACMSVSSSSSSNSLFSSKRKRRLDRFSLSSRCRRGGRVAMAIAIQPSSIEMEEETTLT
KRKQPPTKQRRVVVTGMGVETPIGNNPDQFYNNLLQGVSGITQIEAFDCSSYPTRIAGEIKNFSTDGWVA
PKLSKRMDRFMLYMLTAGKKALADAGISPSDSDEIDKSRCGVLIGSAMGGMKVFNDAIEALRVSYRKMNP
FCVPFATTNMGSAMLAMDLGWMGPNYSISTACATSNFCILNAANHIIRGEADMMLCGGSDAVIIPIGLGG
FVACRALSERNTDPAKASRPWDSGRDGFVMGEGAGVLLLEELEHAKKRGAKIYAEFLGGSFTCDAYHMTE
PHPEGAGVILCIEKALSQAGVRREDVNYINAHATSTPAGDLKEYHALLHCFGNNQELRVNSTKSMIGHLL
GAAGAVEAVATVQAIRTGWIHPNINLENPDQGVDTKVLVGSKKERLNVKVGLSNSFGFGGHNSSILFAPF
Q
>Ah*FabF-1* 3-oxoacyl-[acyl-carrier-protein] synthase II [*Arachis hypogaea*] XP_025644592.1
MAFSSAFAASSPLCTWLVAACMSLTSHSDHSPSLTCSRRRTHVTRSSFTPRSTSAFNRTRTRTNRMPLSR
KIMAIALQPAQEATTVSKKPPTKERRVVVTGMGVVTPLGHDADVFYKNLLDGVSGISEINAFDCAEFPTR
IAGEIKSVSADGWVAPKLSKRLDKFMLYMLIAGKKALVDGGITEDIMNELNKQKCGVLIGSAMGGMKVFS
DAIEALRISYKKMNPFCVPFATTNMGSAILAMDLGWMGPNYSISTACATSNFCILNAANHIIRGEADMML
CGGSDSAIIPIGLGGFVACRALSQRNSDPTKASRPWDSNRDGFVMGEGAGVLLLEELEHAKQRGAEIYAE
FRGGSFTCDAYHMTEPHPDGAGVILCIEKALTHSGLSKEDVNYINAHATSTPAGDLKEYQALMHCFGRNP
ELKVNSTKSMIGHLLGAAGGVEAVATVQAIRTGWIHPNINLESPDKGVDANVLVGPKKERLEVKAALSNS
FGFGGHNSSIIFSPFK
>Ah*FabF*-2 3-oxoacyl-[acyl-carrier-protein] synthase II [*Arachis hypogaea*] XP_025645142.1
MIHSAASSMASPLCTWLVAACMSVTCHTDRAGTPKAALRSSKRSRRTKALPANTSQLNMRLISSLYGSSI
QGLISSFEPCDDYCNTHNAFSSLFRSTTPNRRHTRLNKLSHSGKTMAVAVEPAHEVTATKKPPTKQRRVV
VTGLGVVTPLGHEPDVYYNNLLDGVSGISEIETFDCAEYPTRIAGEIKSFSTDGWVAPKLSKRMDKFMLY
LLTAGKKALVDGGITEDVMDELNKEKCGVLIGSAMGGMKVFNDAIEALRVSYKKMNPFCVPFATTNMGSA
ILAMDLGWMGPNYSISTACATSNFCILNAANHIIRGEADVMLCGGSDAAIIPIGLGGFVACRALSQRNTD
PTKASRPWDINRDGFVMGEGAGVLLLEDLEHAKKRGATIYAEFLGGSFTCDAYHVTEPRPDGAGVILCIE
KALAQSGVSKEDVNYINAHATSTPAGDLKEFQALLHCFGQNPELRVNSTKSMIGHLLGAAGGVEAVATVQ

>Gm*FabF* Beta-ketoacyl-ACP synthetase 2 [*Glycine max*], AAF61737.1
MASTTTSSLCTWLVAACMSVTCHADRTKTPHAMFRSSKKSRYSQFNVCRSTHSGKTMAVALQPTQGITTI
KKPPTKQRRVVVTGLGVVTPLGHEPDIFYNNLLDGASGISEIETFDCADIPTRIAGEIKSFSTDGWVAPK
LSKRMDKFMLYMLTAGKKALVDGGITDDVMDELYKDKRGVLIGSAMGGMKVFNDAIEALRISYKKMNPFC
VPFATTHMGSAMLAMDLGWMGPNYSISTACATSNFCILNAANHIIRGEADVMLCGGSDAAIIPIGLGGFV
ACRALSQRNTDPTKASRPWDINRDGFVMGEGAGVLLLEELEHAKERGATILAEFLGGSFTCDAYDVTEPR
PDGAGVILCIEKALAQSGVSKEDVNYINAHATSTPAGDLKEYQALMHCFGQNPELRVNLTKSMIGHLLGA
AGGVEAVATIQAIRTGWVHPNINLENPDNGVDAKVLVGSKKERLDVKAALSNSFGFGGHNSSIIFAPY

>JrFabF Beta-ketoacyl-ACP synthase II [*Jatropha curcas*] ABJ90469.2
MMGSASFASPLCTWLVAACMSVTCAKENRTAPHAFHSSQPSNRLSRWARRRKTLHAQYNSDSSNSIAAGG
GGGGGGGYSTEFLSNSLVSTLCGSSFQGLMSSCLAFEPCSQYYSSNGLFRSRNLNRKQRRLNRLALSGEA
MAIAVQPEKEVATKKKPATKQRRVVVTGMGVVSPLGHEPDVFYNNLLEGVSGISQIEAFECAQFPTRIAG
EIKSFSTDGWIAPKLSKRMDKFMLYMLTAGKKALADGGITEDIMDELDKAKCGVLIGSAMGGMKVFNDAI
EALRVSYRKMNPFCVPFATTNMGSAMLAMDLGWMGPNYSISTACATSNFCILNAANHIIRGEADIMLCGG
SDAAIIPIGLGGFVACRALSQRNDDPAKASRPWDMNRDGFVMGEGAGVLLLEELEHAKKRGANIYAEFLG
GSFTCDAYHMTEPRPGGIGVILCVEKALAQSGVSREDVNYINAHATSTPAGDIKEFGALMHCFGQNPGLR
VNSTKSMIGHLLGAAGAVEAIAAIQAIRTGWVHPNINLENPDEGVDTNVLVGPKKERLDVKVALSNSFGF

GGHNSSIVFAPHK
>AhKASmt 3-oxoacyl-[acyl-carrier-protein] synthase mitochondrial [*Arachis hypogaea*] XP_025632626.1
MAMRASRRVFGSRGLGFYQSISSSSSVGRAIMDPPPVVSSRRVVVTGLGMVTPLGCGVDKTWRHLIDGKC
GVRSLCLEDLQMSSFDKETQLSTFDQLTSKVAAVVPTGTNLGEFNEEIWLNSKDHRSIARFIAYALCAAD
EALKDSNWFPTEQEHRERTGVSIGGGIGSISDVLDSAQLLCEKRLRRLSPFFIPRILVNMASGHVSMKYG
FQGPNHAAVTACATGAHSIGDAVRMIQFGDADVMVAGGTESSIDALSIAGFCRSRALTTKYNSSPQEASR
PFDSGRDGFVIGEGCGVLVLEEFNHAKNRGAKVYAEVRGYGMSGDAHHITQPPNDGRGAILAMTHALRQS
GLHPSEVDYINAHATSTPLGDVIEANAIKTIFSGHANSSALAFSSTKGAIGHLLGAAGAVEAIFSVLAIR
HGIAPLTLNLTKPDPVFADGFMPLTASKEMPIRVAMSNSFGFGGTNASLLFASVGSDR

>BrSAD1 Stearoyl-[acyl-carrier-protein] 9-desaturase [*Brassica rapa*] XP_009133630.1
MALKLNPLASQPYNFPSSARPPVSTFRSPKFLCLASSSPALSSKEVESLKKPFTPPKEVHVQVLHSMPPQ
KIEIFKSMEDWAEQNLLTQLKDVEKSWQPQDFLPDPASDGFEDQVRELRERARELPDDYFVVLVGDMITE
EALPTYQTMLNTLDGVRDETGASPTSWAIWTRAWTAEENRHGDLLNKYLYLSGRVDMRQIEKTIQYLIGS
GMDPRTENNPYLGFIYTSFQERATFISHGNTARQAKEHGDLKLAQICGTIAADEKRHETAYTKIVEKLFE
IDPDGTVMAFADMMRKKISMPAHLMYDGRDESLFDNFSSVAQRLGVYTAKDYADILEFLVGRWKIESLTG
LSGEGNKAQEYLCGLTPRIRRLDERAQARAKKGPKVPFSWIHDREVQL
>BrSAD2 stearoyl-[acyl-carrier-protein] 9-desaturase [*Brassica rapa*] XP_009142970.1
MALKLNPLASQPYKLHTSARPPISTFRSPKFLCLASSSSPALSSSTKEVESLKKPFTPPKEVHVQVLHSM
PPQKIEIFKSMEDWAEHNLLPHLKDVEKSWQPQDFLPDPASDGFEDQVKELRERARELPDDYFVVLVGDM
ITEEALPTYQTMLNTLDGVRDETGASPTSWAVWTRAWTAEENRHGDLLNKYLYLSGRVDMRQIEKTIQYL
IGSGMDPRTENNPYLGFIYTSFQERATFVSHGNTARQAKEHGDLKLAQICGTIAADEKRHETAYTKIVEK
LLEIDPDGTVVAFADMMRKKISMPAHLMYDGRDDKLFDNFSSVAQRLGVYTAKDYADILEFLVGRWKIES
LSGLSGEGNKAQEYLCGLTPRIRRLDERAQARAKKGPKIPFSWIHDREVQL
> BrSAD3A Stearoyl-[acyl-carrier-protein] 9-desaturase [*Brassica rapa*] XP_018513314.1
MAMSMDRIVVSPSSYVCRPSQARGSRSSVVSMASTIRSASTYREVTNGKKLYIPPREVHVQVKHSMPPQK
LEIFKSLEGWADETLLTYLKPVEKSWQPTDFLPEAESEGFYDQVKELRERCKELPDEYFVVLVGDMITEE
ALPTYQTMLNTLDGVRDETGASPTPWAVWTRAWTAEENRHGDLLNKYLYLSGRVDMRQIEKTIQYLIGSG
MDPKTENNPYLGFIYTSFQERATFISHGNTARLAKDRGDLKLAQICGTIAADEKRHETAYTKIVEKLFEI
DADGTILGLADMMKKKISMPAHLMYDGQDDNLFEHFSTVAQRLGVYTAKDYADILEFLVERWNVETLSGL
SSEGHMAQDFVCGLPARIRRIEERAQGRAKEAAKNVPFSWIFGREIRA
> BrSAD4 Stearoyl-[acyl-carrier-protein] 9-desaturase [*Brassica rapa*] XP_009118393.2
MAMAMAMNRIVVLPSSYAYRPHQARGSRSPIASSIRSATTEVTNGRKLYIPPREVHVQVKHSMPPQKLEI
FKSLEGWADETLLTYLKPVEKSWQPTDFLPEAESEGFYDQVKELRERCKELPDEYFVVLVGDMITEEALP
TYQTMLNTLDGVRDETGASPTPWAVWTRAWTAEENRHGDLLNKYLYLSGRVDMRQIEKTIQYLIGSGMDP
KTENNPYLGFIYTSFQERATFISHGNTARHAKDLGDLKLAQICGTIAADEKRHETAYTKIVEKLFEIDAD
GTILGLADMMKKKISMPAHLMYDGQDDNLFEHFSTVAQRLGVYTAKDYADILEFLVERWNVETLSGLSSE
GHKAQDFVCGLPARIRKIEERAQGRAKEAAKNVPFSWIFGREIRA
> BrSAD5 Stearoyl-[acyl-carrier-protein] 9-desaturase [*Brassica rapa*] XP_009121502.1
MVMAMDRIAFTSSPSAYQRSYHPHGYRSSRVFMSSTIRPASTEVTNGRKPYLPPREVQLQVKYSMPPQKL
EIFKSLEEWANDNLLPYLKPVEKSWQPTDFLPEPESEGFYDQVKELRERCKELSDDYFVVLIGDMITEEA
LPTYQTMINTLDGVRDETGASPTPWAVWTRAWTAEENRHGDLLNKYLYLSGRVDMRQIEKTIQYLIGSGM
DPKTENNPYLGFIYTSFQERATFISHGNTAGLAKKLGDSKLAQICGTIAADEKRHETAYTKIVEKLFEID
PDTTVVGFADMMKKKIAMPAHLMYDGCDDNLFEHFSSVAQRLGVYTARDYADILEFLVRRWNVENLVGLS
GDGHKAQDYLCKLPARIRKLEERAQGRNKDAARNIPFSWIFGREIRA
> BrSAD6 Stearoyl-[acyl-carrier-protein] 9-desaturase [*Brassica rapa*] XP_009147347.1
MTMTLPLNSTKTTPMKQNPVVAVSSSPSLFLGPSFPRRLRVSCVATSPRKTSEQTHKKTFRPIKEVPNQI

THTITQEKLEIFKSMENWAQENLLSYLKPVETSWQPQDLLPQTNDEDQFYEQVKELRDRTREIPDDYFVV
LVGDMITEEALPTYQTTLNTLDGVKDETGGSLTPWAVWVRAWTAEENRHGDLLNKYLYLSGRVDMQHVER
TIQYLIGSGMDSKFENNPYNGFIYTSFQERATFISHANTAKLATTYGDTTLAKICGTIAADEKRHETAYT
RIVEKLFEIDPDGTVQALASMMRKRITMPAHLMHDGRDDNLFDHYGAVAQRIGVYTAMDYAGILEFLLRR
WKVESLGVGLTGEGRRAQEYLCSLPQRITRLEERANDRVKLGSRPSVAFSWIYGREVGL
>BrSAD7 Stearoyl-[acyl-carrier-protein] 9-desaturase [*Brassica rapa*]
XP_009121503.1
MSIALLLPSPVMTQKPSSITSPPRGFGSSSSRLLQVSCVTKNPARNNNVTCNNFRPIKEVNNQITHTIPQ
EKLEIFKSMENWAETTLLPYLKPVEDSWQPQDFLPAPETDDAFYDQVREIRERTKEIPDDYFVVLVGDMI
TEEALPTYQTTLNTLDGVKDETGGSLSPWAVWIRAWTAEENRHGDLLNKYLYLCGRVDMRHVERTIQYLI
GSGMDSKFENNPYNGFIYTSFQERATFISHGNTARLATTYGDTTLAKICGTIAADEKRHETAYTKIVEKL
FELDPDGTVQALASMMKKRITMPAHLMHDGRDDQLFDHYAAVAQRIGVYTAADYAGILEFLLRRWKVESL
GSGLSGEGRRAQEYLCTLPQRIKRLEERANDRVKRQSKGSVSFSWVFGRDVEL
>RcSAD1 Stearoyl-[acyl-carrier-protein] 9-desaturase [*Ricinus communis]*
NP_001310659.1
MALKLNPFLSQTQKLPSFALPPMASTRSPKFYMASTLKSGSKEVENLKKPFMPPREVHVQVTHSMPPQKI
EIFKSLDNWAEENILVHLKPVEKCWQPQDFLPDPASDGFDEQVRELRERAKEIPDDYFVVLVGDMITEEA
LPTYQTMLNTLDGVRDETGASPTSWAIWTRAWTAEENRHGDLLNKYLYLSGRVDMRQIEKTIQYLIGSGM
DPRTENSPYLGFIYTSFQERATFISHGNTARQAKEHGDIKLAQICGTIAADEKRHETAYTKIVEKLFEID
PDGTVLAFADMMRKKISMPAHLMYDGRDDNLFDHFSAVAQRLGVYTAKDYADILEFLVGRWKVDKLTGLS
AEGQKAQDYVCRLPPRIRRLEERAQGRAKEAPTMPFSWIFDRQVKL
>AhSAD1 Stearoyl-[acyl-carrier-protein] 9-desaturase [*Arachis hypogaea*]
ACI45450.1
MALRLNPNPSQKLFLSPSSSSSSSSSFSLPQMASLRSPRFRMASTLRTGSKEVENLKKPFTPPREVHVQ
VTHSMPPQKIEIFKSLEGWAEENILTLLKPVGKCWQPQDYLPEPSEDGFEEQVRELRARAKELPDDYFVV
LVGDMITEEALPTYQTMLNTLDGVRDETGASLTSWAVWTRAWTAEENRHGDLLNKYLYLSGRVDLRQIEK
TIQYLIGSGMDPRTENSPYLGFIYTSFQERATFISHGNTARLAKEHGDMKLAQICGMIASDEKRHETAYT
KIVEKLFEIDPDGTVMAFADMMRKKIAMPAHLMYDGRDDNLFENYSAVAQRIGVYTAKDYADILEFLVGR
WKVADLTGLSGEGRKAQDYVCGLPPRIRRLEERAQGRAKESPRLKFSWIYDREVQL
>AhSAD2 Stearoyl-[acyl-carrier-protein] 9-desaturase [*Arachis hypogaea*]
AID51207.1
MQMQISSLRTQTFLHFSPAQKYGRHIMPLPRAAAIAAPPPLKSQKTHSMAPEKIEIFKSLENWASECVLP
MLKPVEQCWQPQNLVPDSSQPFDEFTDEVKNLRQRTAEIPDDYFVVLVGDMITEEALPTYQSMINNLDGV
GDEIGSSPNPWAVWTRAWTAEENRHGDLLRTYLYLSGRVDMLMIEKTVQYLIGAGMDPGTENNPYLGFVY
TSFQERATFVSHGNTARLAKEGGDPVLARICGTIAADEKRHENAYQKIVEKLLEVDPSDAVVAIGDMMQK
KITMPAHLMYDGSDPKLFEHFSAVAQRLGVYTANDYADILEFLVGRWRLEKLEGLTAEGKRAQDYVCGLS
QMIRRLQERADERARNMKKSHSVKFSWIFNKEVLL
>AhSAD3 Stearoyl-[acyl-carrier-protein] 9-desaturase [*Arachis hypogaea*]
AID51208.1
MILLGWFLPIPEEEMAMQLQTRMMFTPKRASLAHAWTPSGIHISMLHAHARSSSSSSTLPPEKIEIFKSL
EGWASQCILPLVKPVEKSWQPHDLLPDSSLSLDEFIYQVKALRDRTAELPDDYLVVLVGDMITEEALPSY
QTWFNQLDGVGDKLGSSPNPWAVWSRAWTSEENRHGDLLKTYLYLSGRVDMFMIEKTIHYLIGAGVDVKT
ENNPYMGFVYTSFQERATFISHGNTARLATKSGDPVLARICGTIAADEKRHENVYSKIVEKLLEVDPTGT
MIAISEMMRKKITMPAYLMHDGRDPHLFDHFSAVAQRLGVYTAADYADILEYLIGRWRLGELQGLTPEGR
HAQDFVCGLAPRIRRLQERADKQVCKMKSRSVKFSWIFSREVPII
>AtFAB2 Stearoyl-acyl-carrier-protein desaturase [*Arabidopsis thaliana*]
AT2G43710.1
MALKFNPLVASQPYKFPSSTRPPTPSFRSPKFLCLASSSPALSSGPKEVESLKKPFTPPR
EVHVQVLHSMPPQKIEIFKSMENWAEENLLIHLKDVEKSWQPQDFLPDPASDGFEDQVRE
LRERARELPDDYFVVLVGDMITEEALPTYQTMLNTLDGVRDETGASPTSWAIWTRAWTAE
ENRHGDLLNKYLYLSGRVDMRQIEKTIQYLIGSGMDPRTENNPYLGFIYTSFQERATFIS
HGNTARQAKEHGDIKLAQICGTIAADEKRHETAYTKIVEKLFEIDPDGTVMAFADMMRKK
ISMPAHLMYDGRNDNLFDNFSSVAQRLGVYTAKDYADILEFLVGRWKIQDLTGLSGEGNK
AQDYLCGLAPRIKRLDERAQARAKKGPKIPFSWIHDREVQL
>AtAAD2 Stearoyl-acyl-carrier-protein desaturase [*Arabidopsis thaliana*]
AT3G02610.1
MALLLNSTITVAMKQNPLVAVSFPRTTCLGSSFSPPRLLRVSCVATNPSKTSEETDKKKFRPIKEVPNQV

THTITQEKLEIFKSMENWAQENLLSYLKPVEASWQPQDFLPETNDEDRFYEQVKELRDRTKEIPDDYFVV
LVGDMITEEALPTYQTTLNTLDGVKDETGGSLTSWAVWVRAWTAEENRHGDLLNKYLYLSGRVDMRHVEK
TIQYLIGFGMDSKFENNPYNGFIYTSFQERATFISHGNTAKLATTYGDTTLAKICGTIAADEKRHETAYT
RIVEKLFEIDPDGTVQALASMMRKRITMPAHLMHDGRDDDLFDHYAAVAQRIGVYTATDYAGILEFLLRR
WEVEKLGMGLSGEGRRAQDYLCTLPQRIRRLEERANDRVKLASKSKPSVSFSWIYGREVEL
>AtAAD3 Stearoyl-acyl-carrier-protein desaturase [*Arabidopsis thaliana*]
AT5G16230.1
MSMALLLTSPAMKQKPAVITSPRRGSSPSRRLRVSCVTTNPARKKNETCNHFRPIKEVNNQLTHTIPQEK
LEIFKSMENWAEQKLLPYLKPVEDSWQPQDFLPAPENDDEFYDRVKEIRERTKEIPDDYFVVLVGDMITE
EALPTYQTTLNTLDGVKDETGGSLSPWAVWIRAWTAEENRHGDLLNKYLYLTGRVDMRHVEKTIQYLIGS
GMDSKFENNPYNGFIYTSFQERATFISHGNTARLATTYGDVTLAKICGTIAADEKRHETAYTKIVEKLFE
IDPDGSVQALASMMKKRITMPAHLMHDGRDNDLFDHYAAVAQRIGVYTAADYAGILEFLLRRWKVESLGL
GLSGEGRRAQEYLCTLPQRIKRLEERANDRVKLVSKPSVSFSWVFGRDVKL
>AtSAD4 Stearoyl-acyl-carrier-protein desaturase [*Arabidopsis thaliana*]
AT3G02620.1
MALLLNSTMTVAMKQNPATAVSFMQTTCLGSSFSPPRHLQVSCVATNPSKTFRPIKEVSNQVTHTITQEK
LEIFKSMENWAQENLLSYLKPVETSWQPQDFLPETKDEDRFYEQVKELRDRTKEIPDDYFVVLVGDMITE
EALPTYQTVMNTLDGAKDETGVSLTPWAVWLRAWTAEENRHGDLLNKYLYLSGRVDTRHVEKTIQYLIGS
GMDTKYENNPYNGYIYTSFQERATFISHANTAKLATTYGDTTLAKICGTIAADEKRHEMAYTRIVEKLFE
IDPDGTVQALASMMRKRITMPAQLMHDGRDDNLFDHYAAVAQRIGVYTATDYAGILEFLLRRWEVEKLGM
GLSGEGRRAQDYLCTLPQRIRRLEERADDRVKRASKSKPSVSFSWIYGREVEL
>AtSAD5 Stearoyl-acyl-carrier-protein desaturase [*Arabidopsis thaliana*]
AT3G02630.1
MAMAMDRIVFSPSSYVYRPCQARGSRSSRVSMASTIRSATTEVTNGRKLYIPPREVHVQVKHSMPPQKLE
IFKSLEGWADETLLTYLKPVEKSWQPTDFLPEPESEGFYDQVKELRERCKELPDDYFVVLVGDMITEEAL
PTYQTMLNTLDGVRDETGASPTPWAIWTRAWTAEENRHGDLLNKYLYLSGRVDMRQIEKTIQYLIGSGMD
PKTENNPYLGFIYTSFQERATFISHGNTARLAKDRGDLKLAQICGTIAADERRHETAYTKIVEKLFEIDP
DGTILGLADMMKKKISMPAHLMYDGQDDNLFEHFSTVAQRLGVYTAKDYADILEFLVERWNVETLTDLSS
EGHRAQDFVCGLPARIRKIEERAQGRAKEAAKNIPFSWIFGRNIRA
>AtSAD6 Stearoyl-acyl-carrier-protein desaturase [*Arabidopsis thaliana*]
AT5G16240.1
MVMAMDRIALFSSSSSVYHHGSSHSHGSKSSRVFTIRSDSTAVGRKLYIPPREVHLQVKYSMPPQKLEIF
KSLEGWANDNLLAYLKPVEKSWQPTDFLPEPESEGFYDQVKELRERCKELSDDYLIVLVGDMITEEALPT
YQTMINTLDGVRDETGASPTPWAVWTRAWTAEENRHGDLLNKYLYLSGRVDMRQIEKTIQYLIGSGMDPK
TENNPYLGFIYTSFQERATFISHGNTARLAKDLGDLTLGKICGTIAADERRHEHAYTKIVEKLFEIDPDT
TVVGFADMMRKKISMPAHLMYDGRDDNLFDHFSSVAQRLGVYTAKDYADILQHLVERWNVEKLSDLSSEG
NRAQDYLCGLPARIRKLEERAQGRTKEAAKNIPFSWIFGREVRA
>AtSAD7 Stearoyl-acyl-carrier-protein desaturase [*Arabidopsis thaliana*]
AT1G43800.1
MLAHKSLLSFTTQWATLMPSPSTFLASRPRGPAKISAVAAPVRPALKHQNKIHTMPPEKMEIFKSLDGWA
KDQILPLLKPVDQCWQPASFLPDPALPFSEFTDQVRELRERTASLPDEYFVVLVGDMITEDALPTYQTMI
NTLDGVRDETGASESAWASWTRAWTAEENRHGDLLRTYLYLSGRVDMLMVERTVQHLIGSGMDPGTENNP
YLGFVYTSFQERATFVSRGNTARLAKSAGDPVLARICGTIAADEKRHENAYVRIVEKLLEIDPNGAVSAV
ADMMRKKITMPAHLMTDGRDPMLFEHFSAVAQRLEVYTADDYADILEFLVGRWRLEKLEGLTGEGQRAQE
FVCGLAQRIRRLQERADERAKKLKKTHEVCFSWIFDKQISV
> Muc-PAD Acyl-ACP desaturase, [Macfadyena unguis-cati] AAC05293.1
MALKLNAINFQSPKCSSFGLPPVVSLRSPKLSVAATLRSGLRDVETVKKTFSPAREVHVQVTHSMAPQKI
EIFKAMEDWAENNILVHLKNVEKCPQPQDFLPDPASDEFHDQIKELRERAKEIPDDYFVVLVGDMITEEA
LPTYQTMLNTWDGVRDETGASPTSWAIWTRAWTAEENRHGDPLNKYLYLSGRVDMKQIEKTIQYLIGSGM
DPRTENSPYLGFIYTSFQERATFISHGNTARLARDHGDFKLAQICGTIASDEKRHETAYTKIVEKLFEID
PDGTVLAFGDMMKKKISMPDHFMYDGRDDNLFDHFSSVAQRLGVYTAKDYADILEHLVGRWKVEKLTGLS
AEGQKAQDYVCGLPPRIRRLEERAQIRAKQAPRLPFSWIYDREVQL

>AtFatA-1 Acyl-ACP thioesterase A, [*Arabidopsis thaliana], NP_189147.1*
MLKLSCNVTDSKLQRSLLFFSHSYRSDPVNFIRRRIVSCSQTKKTGLVPLRAVVSADQGSVVQGLATLAD
QLRLGSLTEDGLSYKEKFVVRSYEVGSNKTATVETIANLLQEVGCNHAQSVGFSTDGFATTTTMRKLHLI
WVTARMHIEIYKYPAWGDVVEIETWCQSEGRIGTRRDWILKDSVTGEVTGRATSKWVMMNQDTRRLQKVS

DDVRDEYLVFCPQEPRLAFPEENNRSLKKIPKLEDPAQYSMIGLKPRRADLDMNQHVNNVTYIGWVLESI
PQEIVDTHELQVITLDYRRECQQDDVVDSLTTTTSEIGGTNGSATSGTQGHNDSQFLHLLRLSGDGQEIN
RGTTLWRKKPSS
>AtFatA-2 Acyl-ACP thioesterase A [*Arabidopsis thaliana] NP_189147.1*
MLKLSCNVTDHIHNLFSNSRRIFVPVHRQTRPISCFQLKKEPLRAILSADHGNSSVRVADTVSGTSPADR
LRFGRLMEDGFSYKEKFIVRSYEVGINKTATIETIANLLQEVACNHVQNVGFSTDGFATTLTMRKLHLIW
VTARMHIEIYKYPAWSDVVEIETWCQSEGRIGTRRDWILKDCATGEVIGRATSKWVMMNQDTRRLQRVTD
EVRDEYLVFCPPEPRLAFPEENNSSLKKIPKLEDPAQYSMLGLKPRRADLDMNQHVNNVTYIGWVLESIP
QEIIDTHELKVITLDYRRECQQDDIVDSLTTSETPNEVVSKLTGTNGSTTSSKREHNESHFLHILRLSEN
GQEINRGRTQWRKKSSR
>CsFatA-1 Acyl-ACP thioesterase A [*Camelina sativa]* AFQ60948.1
MLKLSCNVTDSSKLQRSLLFFSHSHRSDPVNCIRRRIVSSSVSCSQTKKTGLVDPLRAVVSADQGSVIRA
EQGLGTLADQLRLGSLTEDGLSYKEKFVVRSYEVGSNKTATIETIANLLQEVGCNHAQSVGFSTDGFATT
PTMRKLHLIWVTARMHIEIYNYPAWGNVVEIETWCQSEGRIGTRRDWILKDCATGEVTGRATSKWVMMNQ
DTRRLQKVSDDVRDEYLVFCPQEPRLAFPEENNRSLKKIPKLEDPAQYSMIGLKPRRADLDMNQHVNNVT
YIGWVLESVPQEIVDTHELQVITLDYRRECQQDDVVDSLTTSEIGGTNGSATSGTQGHNDSQFLHLLRLS
GDGQEINRGTTLWRKKSSR
>CsFatA-2 Acyl-ACP thioesterase A [*Camelina sativa] AFQ60947.1*
MLKLSCNVTDSSKLQRSLLFFSHSHRSDPVNCIRRRIVASSVSCSQTKKTGLVDPLRAVVSADQGSVIRA
EQGLGTLADQLRLGSLTEDGLSYKEKFVVRSYEVGSNKTATIETIANLLQEVGCNHAQGVGFSTDGFATT
PTMRKLHLIWVTARMHIEIYNYPAWGDVVEIETWCQSEGRIGTRRDWILKDCATGEVTGRATSKWVMMNQ
DTRRLQKVSDDVRDEYLVFCPQEPRLAFPEENNRSLKKIPKLEDPAQYSMIGLKPRRADLDMNQHVNNVT
YIGWVLESVPQEIVHTHELQVITLDYRRECQQDDVVDSLTTSEIGGTNGSATSGTQGHNDSQFLHLLRLS
GDGQEINRGTTLWRKKSSR
>CsFatA-3 Acyl-ACP thioesterase A [*Camelina sativa], AFQ60948.1*
MLKLSCNVTDSSKLQRSLLFFSHSHRSDPVNCIRRRIVSSSVSCSQTKKTGLVDPLRAVVSADQGSVIRA
EQGLGTLADQLRLGSLTEDGLSYKEKFVVRSYEVGSNKTATIETIANLLQEVGCNHAQSVGFSTDGFATT
PTMRKLHLIWVTARMHIEIYNYPAWGNVVEIETWCQSEGRIGTRRDWILKDCATGEVTGRATSKWVMMNQ
DTRRLQKVSDDVRDEYLVFCPQEPRLAFPEENNRSLKKIPKLEDPAQYSMIGLKPRRADLDMNQHVNNVT
YIGWVLESVPQEIVDTHELQVITLDYRRECQQDDVVDSLTTSEIGGTNGSATSGTQGHNDSQFLHLLRLS
GDGQEINRGTTLWRKKSSR
>HaFatB Fatty acyl-ACP thioesterase B [*Helianthus annuus]* CAC80370.1
MVAMSATASLFPVSSPKPHSGAKTSDKLGGEPGSVAVRGIKTKSVNSGGMKVKANAQAPTEVNGSRSRIT
HGFKTDDYSTSPAPRTFINXLPDWXMLLAAITTIFLAAEKQWMMLEWKTKRPDMIADMDPFGLGRIVQDG
LVFRQNFSIRSYEIGADRTASIETLMNHLQETALNHVKSAGLLGDGFGSTPEMCKKNLFWVVTKMQVIVD
RYPTWGDVVQVDTWVAPNGKNGMRRDWLLRDYKTGEILTRASSNWVMMNKETRRLSKIPDEVRGEIEHYF
VDAPPVVEDDSRKLSKLDESTADYVRDGLIPRWSDLDVNQHVNNVKYIGWILESAPQVVEKYELARITLE
YRRECRKDSVVKSLTSVLGGGDDDNGGIGDSGRVDCQHVLLFAGGGDGTPGGEIVKGRTQWRPKYEKQDG
SVDHFSAGNV
>AhFatB Fatty acyl-ACP thioesterase B [*Arachis hypogaea*] ABO38555.1
MATAATASIFPVPSPSPDAGADGNKLVGGSVKLQGLKSKHASSGGLQVKAHAQAPPKINGSTVESLKHDD
DLPSPPPRTFINQLPDWSMLLAAITTIFLAAEKQWMMLDWKPRRSDMLIDPFGIGRIVQDGLVFRQNFSI
RSYEIGADRTASIETVMNHLQETALNHVKTAGLLGDGFGSTPEMCKKSLIWVVTRMQVVVDRYPTWGDVV
QVDTWVSASGKNGMRRDWLLRDCKTGEILTRASSVWVMMNKLTRRLSKIPEEVRAEIASYFVNSAPILEE
DNRKLSKLDDNTADYIRTGLSPRWNDLDVNQHVNNVKYIGWILESAPQPILESHELSAMTLEYRRECGRD
SVLQSLTAVSAADVGNLAHRGQLECKHLLRLEDGAEIVRGRTEWRPKPVSNFDIVNQVPAESI
>GmFatA acyl-ACP thioesterase A [*Glycine max*] XP_006602508.1
MLKLSCNGLDRAHSLAQCGFAGRPACAVPRRRRSGVSGFRLPEGRSIRVSAAVSAKDGAVATRVEADPGTL
ADRLRVGSLTEDGLSYKEKFIVRSYEVGINKTATVETIANLLQEVGCNHAQSVGYSTDGFATTPTMRKLRL
IWVTARMHIEIYKYPAWSDIVEIETWCQGEGRVGTRRDFILKDYATDEVIGRATSKWVMMNQDTRRLQKVS
DDVKEEYLVFCPREPRLAIPEADSNSLKKIPKLEDPAQYSRLGLVPRRADLDMNQHVNNVTYIGWVLESMP
QEIIDSHELQSITLDYRRECGQHDIVDSLTSVEAIQGGAEAVPELKGTNGSATAREDKHEHQQFLHLLRLS
TEGLEINRGRTEWRKKAPR
>RcFatA acyl-ACP thioesterase A [*Ricinus communis] NP_001310680.1*
MLKVPCCNATDPIQSLSSQCRFLTHFNNRPYFTRRPSIPTFFSSKNSSASLQAVVSDISS
VESAACDSLANRLRLGKLTEDGFSYKEKFIVRSYEVGINKTATVETIANLLQEVGCNHAQ
SVGFSTDGFATTTSMRKMHLIWVTARMHIEIYKYPAWSDVVEVETWCQSEGRIGTRRDWI

LTDYATGQIIGRATSKWVMMNQDTRRLQKVTDDVREEYLVFCPRELRLAFPEENNRSSKK
ISKLEDPAQYSKLGLVPRRADLDMNQHVNNVTYIGWVLESIPQEIIDTHELQTITLDYRR
ECQHDDIVDSLTSVEPSENLEAVSELRGTNGSATTTAGDEDCRNFLHLLRLSGDGLEINR
GRTEWRKKSAR

>HaFatB-1 Fatty acyl-ACP thioesterase B [*Helianthus annuus*] XP_022024086.1
MTTLSLSYVPSFRTTSFNDNDIRSMVWGYKIFGASNTMKMGRTTMYATPVTKGVNGKPAV
VERTMIPTQKQFVDPFRQGIIILDGVGYRQTVVVRSYEVGPDKAVTLQSILNLLQETALN
HVWMSGLLGDGFGATHGMMKNNLIWVVSRMQVQVDQYPIWGEILEIDTWVGASGKNGMRR
DWEIRSHTTGIVFARATSTWVMMNQKTRRLSKMPDEVRAEISPWFINKQAIKEESVEKID
KLDDNAKYINSGLQPKRSDLDMNHHVNNVKYVGWMLEAIPEECLENYQLSNIILEYRREC
GSSDVVESLCEPEQVGIIEDVNYMSRCPIGAGFLNCLVNQCIRFTHVLQVKEDSKCQEIV
RGKTTWKKRNNHYPYCG
>HaFatB-2 Fatty acyl-ACP thioesterase B [*Helianthus annuus*] XP_021989855.1
MVAMAASTYFFLNSSSYHDMTRNNLRRTPGPKDAHGIIKKNDGSSRSLLAKDLTKVNATE
VRANRASFLKPIMMLGQNKNYLDMIPDLGFGSMLSDGFVFRQNFQIRSYEVGSDQAVTTE
TLMNLLQETSINHLKAIGVWGDGLGLTREMCKKNLIWVMSKLQVAVDRYPIWDDVIQIDT
WKGAYGKIGMCSNWKFCDAKTGEILLTASCIWLMMNEETRRLSKFPDEVRSELDEHFTNA
TLPVIARKWSPHDEMSIVDHVRNGIMPRWSDLDINHHVNNVKYIGWILESVPHSIVENYE
IASLTLEYRQECKMESVVQSHTYVMVSNNGKKGDNDHVDCKHALQLETGGGEIMKGWTIW
RPKQYENRLGLE

>AtFAD2 Fatty acid desaturase 2 [*Arabidopsis thaliana*] NP_187819.1
MGAGGRMPVPTSSKKSETDTTKRVPCEKPPFSVGDLKKAIPPHCFKRSIPRSFSYLISDIIIASCFYYVA
TNYFSLLPQPLSYLAWPLYWACQGCVLTGIWVIAHECGHHAFSDYQWLDDTVGLIFHSFLLVPYFSWKYS
HRRHHSNTGSLERDEVFVPKQKSAIKWYGKYLNNPLGRIMMLTVQFVLGWPLYLAFNVSGRPYDGFACHF
FPNAPIYNDRERLQIYLSDAGILAVCFGLYRYAAAQGMASMICLYGVPLLIVNAFLVLITYLQHTHPSLP
HYDSSEWDWLRGALATVDRDYGILNKVFHNITDTHVAHHLFSTMPHYNAMEATKAIKPILGDYYQFDGTP
WYVAMYREAKECIYVEPDREGDKKGVYWYNNKL
>AtFAD3 Omega-3 fatty acid desaturase [*Arabidopsis thaliana*] AAA61778.1
MVVAMDQRTNVNGDPGAGDRKKEERFDPSAQPPFKIGDIRAAIPKHCWVKSPLRSMSYVVRDIIAVAALA
IAAVYVDSWFLWPLYWAAQGTLFWAIFVLGHDCGHGSFSDIPLLNSVVGHILHSFILVPYHGWRISHRTH
HQNHGHVENDESWVPLPERVYKKLPHSTRMLRYTVPLPMLAYPLYLCYRSPGKEGSHFNPYSSLFAPSER
KLIATSTTCWSIMFVSLIALSFVFGPLAVLKVYGVPYIIFVMWLDAVTYLHHHGHDEKLPWYRGKEWSYL
RGGLTTIDRDYGIFNNIHHDIGTHVIHHLFPQIPHYHLVDATKAAKHVLGRYYREPKTSGAIPIHLVESL
VASIKKDHYVSDTGDIVFYETDPDLYVYASDKSKIN
>AtFAD6 Fatty acid desaturase 6 [*Arabidopsis thaliana*] NP_194824.1
MASRIADSLFAFTGPQQCLPRVPKLAASSARVSPGVYAVKPIDLLLKGRTHRSRRCVAPVKRRIGCIKAV
AAPVAPPSADSAEDREQLAESYGFRQIGEDLPENVTLKDIMDTLPKEVFEIDDLKALKSVLISVTSYTLG
LFMIAKSPWYLLPLAWAWTGTAITGFFVIGHDCAHKSFSKNKLVEDIVGTLAFLPLVYPYEPWRFKHDRH
HAKTNMLVHDTAWQPVPPEEFESSPVMRKAIIFGYGPIRPWLSIAHWVNWHFNLKKFRASEVNRVKISLA
CVFAFMAVGWPLIVYKVGILGWVKFWLMPWLGYHFWMSTFTMVHHTAPHIPFKPADEWNAAQAQLNGTVH
CDYPSWIEILCHDINVHIPHHISPRIPSYNLRAAHESIQENWGKYTNLATWNWRLMKTIMTVCHVYDKEE
NYIPFDRLAPEESQPITFLKKAMPNYTA
>AtFAD7 Fatty acid desaturase 7 [*Arabidopsis thaliana*] NP_187727.1
MANLVLSECGIRPLPRIYTTPRSNFLSNNNKFRPSLSSSSYKTSSSPLSFGLNSRDGFTRNWALNVSTPL
TTPIFEESPLEEDNKQRFDPGAPPPFNLADIRAAIPKHCWVKNPWKSLSYVVRDVAIVFALAAGAAYLNN
WIVWPLYWLAQGTMFWALFVLGHDCGHGSFSNDPKLNSVVGHLLHSSILVPYHGWRISHRTHHQNHGHVE
NDESWHPMSEKIYNTLDKPTRFFRFTLPLVMLAYPFYLWARSPGKKGSHYHPDSDLFLPKERKDVLTSTA
CWTAMAALLVCLNFTIGPIQMLKLYGIPYWINVMWLDFVTYLHHHGHEDKLPWYRGKEWSYLRGGLTTLD
RDYGLINNIHHDIGTHVIHHLFPQIPHYHLVEATEAAKPVLGKYYREPDKSGPLPLHLLEILAKSIKEDH
YVSDEGEVVYYKADPNLYGEVKVRAD
>AtFAD8 Omega-3 fatty acid desaturase [*Arabidopsis thaliana*] AAA65621.1
MASSVLSECGFRPLPRFYPKHTTSFASNPKPTFKFNPPLKPPSSLLNSRYGFYSKTRNWALNVATPLTTL

QSPSEEDTERFDPGAPPPFNLADIRAAIPKHCWVKNPWMSMSYVVRDVAIVFGLAAVAAYFNNWLLWPLY
WFAQGTMFWALFVLGHDCGHGSFSNDPRLNSVAGHLLHSSILVPYHGWRISHRTHHQNHGHVENDESWHP
LPESIYKNLEKTTQMFRFTLPFPMLAYPFYLWNRSPGKQGSHYHPDSDLFLPKEKKDVLTSTACWTAMAA
LLVCLNFVMGPIQMLKLYGIPYWIFVMWLDFVTYLHHHGHEDKLPWYRGKEWSYLRGGLTTLDRDYGWIN
NIHHDIGTHVIHHLFPQIPHYHLVEATEAAKPVLGKYYREPKNSGPLPLHLLGSLIKSMKQDHFVSDTGD
VVYYEADPKLNGQRT
>AhFAD2-1A Omega-6 desaturase [*Arachis hypogaea*] AAB84262.1
MGAGGRVTKIEAQKKPLSRVPHSNPPFSVGQLKKAIPPHCFERSLFISFSYVVYDLLVAYLLFYIATTYF
HKLPYPFSFLAWPIYWAIQGCILTGVWVIAHECGHHAFSKYQLVDDMVGLTLHSCLLVPYFSWKISHRRH
HSNTGSLDRNEVFVPKPKSKVSWYNKYMNNPPGRAISLFITLTLGWPLYLAFNVSGRPYDRFASHYDPYA
PIYSNRERLLIYVSDSSVFAVTYLLYHIATLKGLGWVVCVYGVPLLIVNGFLVTITYLQHTHASLPHYDS
SEWDWLRGALATVDRDYGILNKAFHHITDTHVAHHLFSTMPHYHAMEATNAIKPILGDYYQFDGTPFYKA
LWREAKECLYVEPDDGASKKGVYWYKNKF
>AhFAD2-1B Oleate desaturase [*Arachis hypogaea*] AAF82293.1
MGAGGRVTKIEAQKKPLSRVPHSNPPFSVGQLKKAIPPHCFERSLFISFSYVVYDLLMAYLLFYIATTYF
HKLPYPFSFLAWPIYWAIQGCILTGVWVIAHECGHHAFSKYQLVDDMVGLTLHSCLLVPYFSWKISHRRH
HSNTGSLDRDEVFVPKPKSKVSWYNKYMNNPPGRAISLFITLTLGWPLYLAFNVSGRPYDRFASHYDPYA
PIYSNRERLLIYVSDSSVFAVTYLLYHIATLKGLGWVVCVYGVPLLIVNGFLVTITYLQHTHASLPHYDS
SEWDWLRGALATVDRDYGILNKAFHHITDTHVAHHLFSTMPHYHAMEATNAIKPILGDYYQFDGTPVYKA
LWREAKECLYVEPDDGASQKGVYWYKNKF
>AhFAD2-2 Omega-6 fatty acid desaturase [*Arachis hypogaea*] ACZ06072.1
MGAGGRTSVPPSNRTVEADYAKRVPHEKPPFSLSQVKKAIPPHCFQRSVIRSFSYVVYDLTIAFCLYYAA
TNYFHKLSSPLFYLAWLLYGAVQGCILTGVWVIAHECGHHAFSDYQWLDDTVGLVLHSGLLVPYFSWKYS
HRRHHSNTGSLDRDEVFVPKKKSSIAWWSKYLNNPLGRVLTLTITLTLGWPLYLAFNVSGRPYDRFACHY
DPYGPIYSDRERLQIYISDAGVLAVSYGLIRLVMAKGLAWVVAVYGVPLLVVNGFLVLITYLQHTHPALP
HYDSSEWDWLRGALATVDRDYGILNKVFHNITDTHVAHHLFSTMPHYHAMEATNAIKPILGEYYRFDGTP
FYKAMWREAKECLYVESDQGAQNKGVFWYNNKL
>AhFAD3-4 Omega-3 fatty acid desaturase [*Arachis hypogaea*] QFR98376.1
MAVSHVVYTNGHKKENDEFDASAPPPFKIADIRAAIPRHCWVKNPWRSLSYVLRDLVLVATLLASAIHFN
SWFFWPIYWPLQGTMFWALFVLGHDCGHGSFSNNAKLNSIVGHILHSSILVPYHGWRISHKTHHQNHGHV
ENDESWVPLPEKIYKSLDNVTKLMRFTVPFPIFAYPFYLWRRSPGKEGSHFNPYGKLFSPNERKDVMIST
MCWVAMFSLLLFLSFIVTPLSILKLYGIPYLIFIMWLDFVTYLHHHGYKQKLPWYRGKEWSYLRGGLTTV
DRDYGLVNNIHHDIGTHVIHHLFPQIPHYHLLEATEAAKPVLGKYYREPEKSGPLPSHLIKYLLQSIGQD
HYVSDNGDIVYYQSDPYLLNNNNKSN
>AhFAD6 Omega-6 fatty acid desaturase [*Arachis hypogaea*] ACZ06070.1
MACRLADSFILFKGSHQKPLQSKRAIVAQYSPRVCNLKECGLIQKGFRHRLNFVPRKKVTVIQAVAVPLE
PFPVESAEYRKQLAESYGFRQIGEPLPDDITLKDVIDSLPKKVFEIDDTKAWKTVLISVASYALGLFMIS
KAPWYLLPLAWAWTGTAVTGFFVIGHDCAHKSFSKNKLVEDIVGTLAFLPLIYPYEPWRFKHDRHHAKTN
MLYEDTAWHPVWKDEFDSSPALRNAIIYGYGPFRTWMSIAHWLMWHFDLKKFRTNEVKRVKISLACVFAF
MAIGWPLIIYKTGIMGWIKFWFMPWLGYHFWMSTFTMVHHTAPHIPFKYSENWNAAQAQLNGTVHCDYPQ
WIEILCHDINVHIPHHISPRIPSYNLRAAHKSLQENWGKYLNEASWNWRLMKTIMTVCHVYDKQQNYVAF
DELAPEDSRPITFLKEVMPDYA
>AhFAD7-1A Delta 15 desaturase [*Arachis hypogaea*] AID51460.1
MATWVLSECGLRPLPPMFPRPRTGSISCTKSSSINSTFLSSDLKSFQQQTRFQCCSFKERRRSWEIKVSV
PLRVGTIEEEEEEEEGIINGVVEKREVCEFDPGAPPPFKLSDIRAAIPKHCWVKDPWRSMSYVVRDVVVV
LGLAAAAAHLNNWIVWPLYWAAQGTMFWALFVLGHDCGHGSFSNNPKLNSVVGHLLHSSILVPYHGWRIS
HRTHHQNHGHVENDESWHPLPEKIFKSLDNVTRTLRFTIPFPMLAYPIYLWSRSPGKTGSHFHPDSDLFV
SNERKDVITSTICWAAMASLLVGLGFVMGPIQLLKLYGIPYVLFVMWLDFVTYLHHHGHEDKLPWYRGEE
WSYLRGGLTTLDRDYGWINNIHHDIGTHVIHHLFPQIPHYHLIEATEAAKPVLGKYYREPKKSLPLPFHL
IGDLMRSMKKDHYVSDTGDVVYYQTDPTLGGSSTSI
>AhFAD8 Omega-3 fatty acid desaturase [*Arachis hypogaea*] AFD93985.1
MATWVLSECGLRPLPPMFPRPRTGSISCTKSSSINSTFLSSDLKSFQQQTRFQCCSFKERRRSWEIKVSV
PLRVGTIEEEEEEEEGIINGVVEKREVCEFDPGAPPPFKLSDIRAAIPKHCWVKDPWRSMSYVVRDVVVV
LGLAAAAAHLNNWIVWPLYWAAQGTMFWALFVLGHDCGHGSFSNNPKLNSVVGHLLHSSILVPYHGWRIS
HRTHHQNHGHVENDESWHPLPEKIFKSLDNVTRTLRFTIPFPMLAYPIYLWSRSPGKTGSHFHPDSDLFV
SNERKDVITSTICWAAMASLLVGLGFVMGPIQLLKLYGIPYVLFVMWLDFVTYLHHHGHEDKLPWYRGEE
WSYLRGGLTTLDRDYGWINNIHHDIGTHVIHHLFPQIPHYHLIEATEAAKPVLGKYYREPKKSLPLPFHL

IGDLMRSMKKDHYVSDTGDVVYYQTDPTLGGSSTSI
>GmFAD2-1A Omega-6-desaturase [*Glycine max*] AAX29989.1
MGGRGRVAKVEVQRKKPLSRVPNTKPPFTVGQLKKAIPPHCFQRSLLTSFSYVVYDLSFAFIFYIATTYF
HLLPQPFSLIAWPIYWVLQGCLLTGVWVIAHECGHHAFSKYQWVDDVVGLTLHSTLLVPYFSWKISHRRH
HSNTGSLDRDEVFVPKPKSKVAWFSKYLNNPLGRAVSLLVTLTIGWPMYLAFNVSGRPYDSFASHYHPYA
PIYSNRERLLIYVSDVALFSVTYSLYRVATLKGLVWLLCVYGVPLLIVNGFLVTITYLQHTHFALPHYDS
SEWDWLKGALATMDRDYGILNKVFHHITDTHVAHHLFSTMPHYHAMEATNAIKPILGEYYQFDDTPFYKA
LWREARECLYVEPDEGTSEKGVYWYRNKY
>GmFAD2-2 Omega-6 fatty acid desaturase [*Glycine max*] BAD89862.1
MGAGGRTDVPPANRKSEVDPLKRVPFEKPPFSLSQIKKVIPPHCFQRSVFRSFSYVVYDLTIAFCLYYVA
THYFHLLPSPLSFLAWPIYWAVQGCILTGVWVIAHECGHHAFSDYQLLDDIVGLVLHSGLLVPYFSWKYS
HRRHHSNTGSLERDEVFVPKQKSCIKWYSKYLNNPPGRVLTLAVTLTLGWPLYLALNVSGRPYDRFACHY
DPYGPIYSDRERLQIYISDAGVLAVCYGLFRLAMAKGLAWVVCVYGVPLLVVNGFLVLITFLQHTHPALP
HYTSSEWDWLRGALATVDRDYGILDKVFHNITDTHVAHELFSTMPHYHAMEATKAIKPILGEYYRFDETP
FVKAMWREARECIYVEPDQSTESKGVFWYNNKL
>GmFAD3A Omega-3 fatty acid desaturase A [*Glycine max*] AMY60543.1
MVKDTKPLAYAANNGYQKEAFDPSAPPPFKIAEIRVAIPKHCWVKNPWRSLSYVLRDVLVIAALMAAASH
FNNWLLWLIYWPIQGTMFWALFVLGHDCGHGSFSDSPFLNSLVGHILHSSILVPYHGWRISHRTHHQNHG
HIEKDESWVPLTEKIYKNLDNMTRLVRFTVPFPLFVYPIYLFSRSPGKEGSHFNPYSNLFPPSERKGIAI
STLCWVTMFSMLIYLSFITSPVLLLKLYGIPYWIFVMWLDFVTYLHHHGHHQKLPWYRGKEWSYLRGGLT
TVDRDYGWINNIHHDIGTHVIHHLFPQIPHYHLVEATQAAKSVLGEYYREPERSAPLPFHLIKYLIQSMR
QDHFVSDTGDVVYYQTDSLHLHSHRD
>GmFAD6C Omega-6 fatty acid desaturase [*Glycine max*] P48628.1
MACTLADSLLLFKGSYQKPVLRRDIAARYSPGIFSLNSNGLIQKRFRRQRNFVTRNKVTVIHAVAIPVQP
APVESAEYRKQLAEDYGFRQVGEPLSDDVTLKDVINPLPKEVFEIDDVKAWKSVLISVTSYALGLFMISK
APWYLLPLAWVWTGTAITGFFVIGHDCAHRSFSSNKLVEDIVGTLAFMPLIYPYEPWRFKHDRHHAKTNM
LREDTAWHPVWKDEFESTPLLRKAIIYGYGPFRCWMSIAHWLMWHFDLKKFRPSEVPRVKISLACVFAFI
AIGWPLIIYKTGIMGWIKFWLMPWLGYHFWMSTFTMVHHTAPYIPFKYSEEWNRAQAQLNGTVHCDYPKW
IEILCHDINVHIPHHISPRIPSYNLRAAHKSLQENWGQYLNEASWNWRLMKTIMTVCQVYDKEKSLCCLR
RTCP
>GmFAD7 Omega 3 fatty acid desaturase [*Glycine max*] ACF19424.1
MATWVLSECSLKPLAPVIPRPRTGAVLSSTSKVGFLETNKVLEGSKFQPLRCNLRERNWGLKVSVPLRIA
SIEEEEQKSVDVINGSNGVEHEKLPEFDPGAPPPFNLADIRAAIPKHCWVKDPLKSMSYVVRDVIAVFGL
ASAAAYLNNWLVWPLYWAAQGTMFWALFVLGHDCGHGSFSNNPKLNSVVGHLLHSSILVPYHGWRISHRT
HHQHHGHVENDESWHPLPEKLFRSLDTVTRMLRFTAPFPLLAYPVYLWGRSPGKTGSHFDPSSDLFVPNE
RKDVITSTACWAAMLGLLVGLGFVMGPIQLLKLYGVPYVIFVMWLDLVTYLHHHGHEDKLPWYRGKEWSY
LRGGLTTLDRDYGLINNIHHDIGTHVIHHLFPQIPHYHLVEATEAAKPVFGKYYREPKKSSPLPFHLIGE
LIKSFKTDHFVSDKGDVVYYQTDSEINGSSKSE
>GmFAD8-2 Omega-3 fatty acid desaturase [*Glycine max*] NP_001239777.1
MATWVLSECGLRPLPPVFPRSTRPISCQKPSKSRFLSTNKGVPDLNLQARGLTCCSFRDRKWELGVSAPL
KFATNEGEEEERTNGANNGVGEEVSEFDPSAPPPFKLADIRAAIPKHCWVKDPWKSMSYVVRDVIVVFGL
AAAAAYLNNWVVWPLYWAAQGTMFWALFVLGHDCGHGSFSNNPKLNSVAGHLLHSSILVPYHGWRISHRT
HHQNHGHVENDESWHPLPEKIFKSLDNVTRILRFTLPFPLLAYPIYLWSRSPGKTGSHFNPDSDLFVPSE
RKDVITSTICWTAMAALLVGLGFVMGPVQLLKLYGIPYAIFVMWLDLVTYLHHHGHEDKLPWYRGEEWSY
LRGGLTTIDRDYGWINNIHHDIGTHVIHHLFPQIPHYHLIEATEAAKPVLGQYYREPKKSSPLPIYLIGE
LLRSMKKDHFVSDSGDIVYYQTDPTLSSSSTSQ
>CsFAD2 Omega-6 fatty acid desaturase [*Camelina sativa*] XP_019096117.1
MGAGGRMPVPSSSSKKSETDAIKRVPCEKPPFTLGELKKAIPPQCFKRSIPRSFSYLITDIIVASCFYYV
ATNYFSLLPQPLSYLAWPLYWACQGCVLTGVWVIAHECGHHAFSDYQWLDDTVGLIFHSFLLVPYFSWKY
SHRRHHSNTGSLERDEVFVPKQKSAIKWYGKYLNNPAGRIMMLTVQFVLGWPLYLAFNVSGRPYDGFACH
FFPNAPIYNDRERLQIYLSDAGILAVCFGLYRYAAAQGLASMICLYGVPLLIVNAFLVLITYLQHTHPAL
PHYDSSEWDWLRGALATVDRDYGILNKVFHNITDTHVAHHLFSTMPHYNAMEATKAIKPILGDYYQFDGT
PWYVAMYREAKECIYVEPDREGDKKGVYWYNNKL
>CsFAD2-2 Omega-6 fatty acid desaturase [*Camelina sativa*] NP_001291954.1
MGAGGRMPVPSSSSKKSETDAIKRVPCEKPPFTLGDLKKAIPPQCFKRSIPRSFSYLITDIIIASCFYYV
ATNYFSLLPQPLSYLAWPLYWACQGCVLTGVWVIAHECGHHAFSDYQWLDDTVGLIFHSFLLVPYFSWKY

SHRRHHSNTGSLERDEVFVPKQKSAIKWYGKYLNNPAGRIMMLTVQFVLGWPLYLAFNVSGRPYDGFACH
FFPNAPIYNDRERLQIYLSDAGILAVCFGLYRYAAAQGLASMICLYGVPLLIVNAFLVLITYLQHTHPAL
PHYDSSEWDWLRGALATVDRDYGILNKVFHNITDTHVAHHLFSTMPHYNAMEATKAIKPILGDYYQFDGT
PWYVAMYREAKECIYVEPDREGDKKGVYWYNNKL
>BnFAD2-4 Omega-6 fatty acid desaturase [*Brassica napus*] XP_022570693.1
MGAGGRMQVSPPSSSPETKTLKRVPCETPPFTLGDLKKAIPPHCFKRSIPRSFSYLLFDILVSSSLYHLS
TAYFPLLPHPLPYLAWPLYWACQGCVLTGLWVIAHECGHHAFSDHQWLDDAVGLVFHSFLLVPYFSWKYS
HRRHHSNTGSLERDEVFVPKKKSDIKWYGKYLNNPLGRTVMLTVQFTLGWPLYLAFNVSGRPYSDGFACH
FHPNAPIYNDRERLQIYISDAGVLSVCYGLYRYAGSRGVASMVCVYGVPLMIVNCFLVLITYLQHTHPSL
PHYDSSEWDWLRGALATVDRDYGILNKVFHNITDTHVAHHLFSTMPHYNAMEATKAIKPILGEYYQFDGT
PVVKAMWREAKECIYVEPDRQGEKKGVFWYNNKL
>BnFAD3 Omega-3 fatty acid desaturase [*Brassica napus*] NP_001302953.1
MVVAMDQRSNVNGDSGARKEEGFDPSEQPPFKIGDIRAAIPKHCWVKSPLRSMSYVARDIFAVAALAMAA
VYFDSWFLWPLYWVAQGTLFWAIFVLGHDCGHGSFSDIPLLNSVVGHILHSFILVPYHGWRISHRTHHQN
HGHVENDESWVPLPEKLYKNLPHSTRMLRYTVPLPMLAYPIYLWHRSPGKEGSHFNPYSSLFAPSERKLI
ATSTTCWSIMLATLVYLSFLVGPVTVLKVYGVPYIIFVMWLDAVTYLHHHGHDEKLPWYRGKEWSYLRGG
LTTIDRDYGIFNNIHHDIGTHVIHHLFPQIPHYHLVDATRAAKHVLGRYYRVPKTSGAIPIHLVESLVAS
IKKDHYVSDTGDIVFYETDPDLYVYASDKSKIN
>BnFAD6 Omega-6 desaturase [*Brassica napus*] AAA50157.1
MASRIADSLFAFTGPQQCLPRAPKLASARLSPGVYAVRPIDLLLKGTRRTFLVPAKKRIGCIKAVFVPVA
PPSADNAEDREQLAESYGFKQIGQDLPDNVTLKDIMDTLPKEVFEIDDVKAWKSVLISVTSYALGLFMIA
KAPWYLLPLAWAWTGTAVTGFFVIGHDCAHKSFSKNKLVEDIVGTLAFLPLVYPYEPWRFKHDRHHAKTN
MLVHDTAWQPVPPEEFDSSPVLRKAIIFGYGPIRPWLSIAHWVNWHFNLRKFRPSEVNRVKISLACVFAF
MAVGWPLIIYKVGVLGWVKFWLMPWLGYHFWMSTFTMVHHTAPHIPFKPADEWNAAQAQLNGTVHCDYPS
WIEILCHDINVHIPHHISPRIPSYNLRAAHQSIQENWGKYTNLATWNWRLMKTIMTVCHVYDKEENYIPF
DRLAPEESQPITFLKKAMPDYAA
>HaFAD2 Delta(12) fatty acid desaturase,[*Helianthus annuus*] XP_021972383.1
MGAGGRMSNPVNGEEKPNPDPLQRVPYQKPPFTVGDVKKAIPPHCFNRSVIRSFSYVVYDLTIASIFYYL
ANNYIALLPSPLAYVAWPVYWICQGCVLTGVWVIAHECGHHAFSDYQWLDDTVGLVLHSALLVPYFSWKY
SHRRHHSNTGSIEHDEVFVPKLKSGVRSTARLLNNPPGRILTLLVTLTMGWPLYLMFNVSGRYYDRFACH
FDPNSPIYSNRERAQIFISDAGILTVLFVLFRVAMTKGLTWVLTMYAGPLLVVNGFLVLITFLQHTHPSL
PHYDSTEWDWLRGALATIDRDYGVLNKVFHNITDTHVTHHLFSTMPHYHAMEATKAIKPILGEYYQFDGT
SVFKAMYRETKECIYVDKDEEVKDGVYWYRNKI

>*Bn*LACS1 Long-chain acyl-CoA synthetase 1 [*Brassica napus*] CDY27501.1
MKSFAAKVEDGVRGENGKPSVGPVYRNLLSEKGFPPIDSDITTAWDIFSKSVKKFPDNKMLGWRRIVDEK
VGPYMWKTYKEAYDEVLQIGSALRAIGAEPGCRVGIYGVNCPQWIITMEACAAHTLICVPLYDTLGSGAV
DYIVDQAEIDFVFVQETKIKGLLEPDCKCARRLKAIVSFTNVSEEHNLKGSEVGVKTYSWLDFLQMGCGK
PEETTPPKPFNICTIMYTSGTSGDPKGVVLTHEAVATYIVGMDLFMDQFEDKMTHEDVYLSFLPLAHILD
RMNEEYFFRKGASVGYYHGDLNVLRDDIQELKPTYLAGVPRVFERIHEGIQKALQELNPRRRFIFNALYK
HKLAWLNRGYSHSKASPMADFIAFGKIRDKLGGRIRLLVSGGAPLSTEIEEFLRVTCCCFVVQGYGLTET
LGGTAMGFPDEMCMLGTVGIPAVYNEIRLEEVAEMGYDPLGENPAGEICIRGKCLFSGYYKNPKLTQEVM
KDGWFHTGDIGEIQSNGVLKIIDRKKNLIKLSQGEYVALENLENIYGQNSLVQDIWVYGDSFKSMLVAVI
VPNQETIKRWAKELGFTKPFEELCSLSELQEHIISELKSTAEKNKLRKFEYIKAVTVETKPFDVARDLVT
ATLKNRRNNLLKYYQVQVDEMYRKLASKKI
>*Bn*LACS2 Long-chain acyl-CoA synthetase 2 [*Brassica napus*] CDY29350.1
MAAAADHVVIMEEGRQATAEHPSAGPVYRCKYAKDGLLDLPADLDSPWQLFSEAVKQYPNEQMLGRRVTV
DSKVGPYTWITYREAHDAALRIGSAIRSRGVNPGNCCGIYGSNCPEWIIAMEACMSQGITYVPLYDSLGV
NAVEFIINHAEVSLVFVQEKALSSILACRKGCSSNLKTIVSFGEVSITQKEEAENQCVSLFSWHEFLLMG
NSDETTLPRKQKTDICTIMYTSGTTGEPKGVILSNAAIMVEVLSIDKMLQVTDRSCDTSDLFFSYLPLAH
CYDQVMEIYFLSRGSSVGYWRGDIRYLMDDVHALKPTVFCGVPRVYDKLYAGVMQKISAGGLIRKKLFDL
AYNYKLGNMRKGLSQEEASPRLDRLMFDKIKDALGGRAHMLLSGAAPLPRHVEEFLRIIPASNLSQGYGL
TESCGGSFTTLAGVFSMVGTVGVPMPTVEARLVSVPEMGYEAFSGDVARGEICLRGNSMFSGYHKRQDLT
DQVVINGWFHTGDIGEWQEDGSMKIIDRKKNIFKLSQGEYVAVENLENTYSRCPLIAQIWIYGNSFESFL
VAVVVPERKAIEDWAKLNNQSSPNDFESLCQNLKAQKYFLDELNSTAKQYQLKGFEMLKAVHLEPNLFDI

ERDLITPTFKLKRPQLLKHYKSIIDQLYTEAKASRA
>*Bn*LACS3 Long chain acyl-CoA synthetase 3 [*Brassica napus*] XP_013664517.1
MATDRFIVEVEKGKEGVDGGSPSVGSVYRSIYAKDGFPEPADDLLSCWDIFRLSAEKSPDNPMLGRREIV
DGKAGKYVWQTYKEVYDIVIKLGNSIRTIGVGKGEKCGIYGANSPEWIISMEACNAHGLYCVPLYDTLGA
GAIEFIICHAKVSLAFSEEKKISELLKTAPNSTKYLKNIVSFGEVSNVQRAEAERHGLSIYSWDQFLKLG
EGKHYELPEKKRSDICTIMYTSGTTGDPKGVLLTNESIIYLLEGVKKLLKTINEELTSKDVYLSYLPLAH
IFDRVIEELFIYEAASIGFWRGDVKILVEDIAALKPTIFCAVPRVLERIYNGLQQKLSDGGFLKKTLFNF
AFNYKHNNMVKGKAHEQAAPIFDKIVFKKVKEGLGGRVRLILSGAAPLAAHIESFLRVVACAHVLQGYGL
TESCGGTFVSIPNELQMLGTVGPPVPNVDIRLESVPEMGYDALARKPRGEICIRGKTLFSGYYKREDLTQ
EVFIDGWLHTGDVGEWQPDGAMKIIDRKKNIFKLSQGEYVAVENLENIYSHVAAIESVWVYGNSYESYLV
AVVCPSKIQIEHWAKEHNVSGDFETIGQNQKTKEFILGEFNRVAKDKKLKGFELIKGVHLDTVPFDMERD
LITPSYKKKRPQLLKYYQKEIDEMYNKTKN
>*Bn*LACS4 Long chain acyl-CoA synthetase 4 [*Brassica napus*] NP_001302548.1
MTQQLEKFIFQVEEGREGSDGRPSVGPVYRSVFAKDGFPEPIEGMDSCWDVFRMSVEKYPNNPMLGRREI
VDGKPGKYVWQTYQEVYDIVIKLGNSLRSCGVEEQAKCGIYGANSPEWIISMEACNAHGLYCVPLYDTLG
AGAVEFIITHSEVSIVFVEEKKISELFKTCPKATEYMKTVVSFGGVTREQKEAAETFGLVIYAWDEFLKL
GEGKQFDLPIKKKSDICTIMYTSGTTGDPKGVMISNESIVTLIAGVIRLLKSANEALTVKDVYLSYLPLA
HIFDRVIEECFIQHGAAIGFWRGDVKLLIEDLGELKPTIFCAVPRVLDRVYSGLQQKLAAGGFFKKFMFD
SAFSFKYGHMKKGQSHVEASPLCDKLVFSKVKQGLGGNVRIILSGAAPLASHVESFLRVVACCHVLQGYG
LTESCAGTFVSLPDRLDMLGTVGPPVPNVDIRLESVPEMEYDALASTPRGEICIRGKTLFSGYYKREDLS
KEVLIDGWLHTGDVGEWQPNGSMKIIDRKKNIFKLSQGEYVAVENLENIYGEVQAVDSVWVYGNSFESFL
IAVANPNQHILEKWAAENGVSGDYNALCQNAKAKEFILGELVKMGKEKKMKGFEIIKAVHLDPVPFDMER
DLLTPTYKKKRPQLLKHYQCVIDEMYKTTNESLASRK
>*Bn*LACS5 Long chain acyl-CoA synthetase 5 [*Brassica napus*] XP_013669542.1
MTSQKRFIIEVEAAKEATDGKPSIGPVYRSTFAKDGFPNPIDGIDSCWDIFRTAVEKYPNNRMLGRREIV
NNKAGKYEWKTYKEVYDIVLKLGNSLRSCGIEEGGKCGIYGANCPEWIISMEACNAHGLYCVPFYDTLGS
GAVEFIISHAEVSIAFVEEKKIPELFKTCPNSTKYMKTVVSFGGVKSEQKEEAEKLGLVIHAWDEFLKLG
EGKQYDLPVKKKSDICTIMYTSGTTGDPKGVLMSNESIVTITAGVRHFLAIFNESLTEKDVYISYLPLAH
VFDRAVEECIIQVGGSIGFWRGDVKLLIEDLGELKPSIFCAVPRVLERVYTGLQQKLSVGGFFKKKVFNV
AFSYKFGNMKKGQSHVEAAPFCDKLVFNKVKQGLGGNVRIILSGAAPLASHIESFLRVVACCHVLQGYGL
TESCAGTFVTFPDELDMLGTVGPPVPNVDIRLESVPDMEYDALGSIPRGEICIRGKTLFSGYHKREDLTK
EVLIDGWFHTGDIGEWQPDGSMKIIDRKKNIFKLAQGEYVAVENLENVYGQVEAIDSIWVYGNSFESFLI
AVANPSQQTLERWAAENGVNGDFDSICQNTKARAFLLGELVKTAKERKLKGFEIIRAVHLEPVAFDIERD
LLTPTYKKKRPQLLKYYQNVIDDMYKTAKEGQASVQ
>*Bn*LACS9 Long chain acyl-CoA synthetase 9 [*Brassica napus*] NP_001303207.1
MIPYAAGVIVPLALTLLVRNAKKDKKRGVVVDDVGGEPGHTVRNHRFKDPVSSHWEDISTLPELFEISCK
SHSDRFFLGTRRLIAREVETSEDGKVFEKLHLGDYEWKTFGETLEAVCSFASGLVQIGHKSEERVAIFAD
TREEWFIALQGCFRRNVTVVTIYSSLGEEALCHSLNETEVTTVICGNKELKKLMDISQQLETVKRVICMD
DEFPSEASSTWTTTSLADVQKLGRESPVDPSFPLSADVAVIMYTSGSTGLPKGVMMTHGNVLATVSAVMT
IVPDLGKRDTYMAYLPLAHILELAAESVMATIGSAIGYGSPLTLTDTSNKIKKGTKGDVTALKPTIMTAV
PAILDRVRDGVRKKVDAKGGAAKKLFDFAYARRLSAINGSWFGAWGLEKLLWDVLVFGKIRAVLGGQLRY
LLSGGAPLSGDTQRFINICVGAPIGQGYGLTETCAGGTFSEFDDTSVGRVGAPLPCSFVKLIDWPEGGYL
ISDKPMPRGEIVIGGSNITLGYFKNEEKTKEVYKVDEKGMRWFYTGDIGQFHPDGCLEIIDRKKDIVKLQ
HGEYVSLGKVEAALSISPYVENIMVHADPFYSYCVALVVAAQQTLEGWASKQGIEFTNFEELCAKEQPVK
EVYASLVKAAKQSRLEKFEIPAKIKVLAAPWTPESGLVTAALKLKRDVIRKEFSEDLTKLYAS
>AtLACS1 Long chain acyl-CoA synthetase 1 [*Arabidopsis thaliana*]
AT2G47240
MKSFAAKVEEGVKGIDGKPSVGPVYRNLLSEKGFPPIDSEITTAWDIFSKSVEKFPDNNMLGWRRIVDEK
VGPYMWKTYKEVYEEVLQIGSALRAAGAEPGSRVGIYGVNCPQWIIAMEACAAHTLICVPLYDTLGSGAV
DYIVEHAEIDFVFVQDTKIKGLLEPDCKCAKRLKAIVSFTNVSDELSHKASEIGVKTYSWIDFLHMGREK
PEDTNPPKAFNICTIMYTSGTSGDPKGVVLTHQAVATFVVGMDLYMDQFEDKMTHDDVYLSFLPLAHILD
RMNEEYFFRKGASVGYYHGNLNVLRDDIQELKPTYLAGVPRVFERIHEGIQKALQELNPRRRFIFNALYK
HKLAWLNRGYSHSKASPMADFIAFRKIRDKLGGRIRLLVSGGAPLSPEIEEFLRVTCCCFVVQGYGLTET
LGGTALGFPDEMCMLGTVGIPAVYNEIRLEEVSEMGYDPLGENPAGEICIRGQCMFSGYYKNPELTEEVM
KDGWFHTGDIGEILPNGVLKIIDRKKNLIKLSQGEYVALEHLENIFGQNSVVQDIWVYGDSFKSMLVAVV
VPNPETVNRWAKDLGFTKPFEELCSFPELKEHIISELKSTAEKNKLRKFEYIKAVTVETKPFDVERDLVT
ATLKNRRNNLLKYYQVQIDEMYRKLASKKI

>AtLACS2 Long chain acyl-CoA synthetase 2 [*Arabidopsis thaliana*] AT1G49430
MSLAADNVLLVEEGRPATAEHPSAGPVYRCKYAKDGLLDLPTDIDSPWQFFSEAVKKYPNEQMLGQRVTT
DSKVGPYTWITYKEAHDAAIRIGSAIRSRGVDPGHCCGIYGANCPEWIIAMEACMSQGITYVPLYDSLGV
NAVEFIINHAEVSLVFVQEKTVSSILSCQKGCSSNLKTIVSFGEVSSTQKEEAKNQCVSLFSWNEFSLMG
NLDEANLPRKRKTDICTIMYTSGTTGEPKGVILNNAAISVQVLSIDKMLEVTDRSCDTSDVFFSYLPLAH
CYDQVMEIYFLSRGSSVGYWRGDIRYLMDDVQALKPTVFCGVPRVYDKLYAGIMQKISASGLIRKKLFDF
AYNYKLGNMRKGFSQEEASPRLDRLMFDKIKEALGGRAHMLLSGAAPLPRHVEEFLRIIPASNLSQGYGL
TESCGGSFTTLAGVFSMVGTVGVPMPTVEARLVSVPEMGYDAFSADVPRGEICLRGNSMFSGYHKRQDLT
DQVLIDGWFHTGDIGEWQEDGSMKIIDRKKNIFKLSQGEYVAVENLENTYSRCPLIAQIWVYGNSFESFL
VGVVVPDRKAIEDWAKLNYQSPNDFESLCQNLKAQKYFLDELNSTAKQYQLKGFEMLKAIHLEPNPFDIE
RDLITPTFKLKRPQLLQHYKGIVDQLYSEAKRSMA
>AtLACS3 Long chain acyl-CoA synthetase 3 [*Arabidopsis thaliana*] AT1G64400
MATGRYIVEVEKGKQGVDGGSPSVGPVYRSIYAKDGFPEPPDDLVSAWDIFRLSVEKSPNNPMLGRREIV
DGKAGKYVWQTYKEVHNVVIKLGNSIRTIGVGKGDKCGIYGANSPEWIISMEACNAHGLYCVPLYDTLGA
GAIEFIICHAEVSLAFAEENKISELLKTAPKSTKYLKYIVSFGEVTNNQRVEAERHRLTIYSWDQFLKLG
EGKHYELPEKRRSDVCTIMYTSGTTGDPKGVLLTNESIIHLLEGVKKLLKTIDEELTSKDVYLSYLPLAH
IFDRVIEELCIYEAASIGFWRGDVKILIEDIAALKPTVFCAVPRVLERIYTGLQQKLSDGGFVKKKLFNF
AFKYKHKNMEKGQPHEQASPIADKIVFKKVKEGLGGNVRLILSGAAPLAAHIESFLRVVACAHVLQGYGL
TESCGGTFVSIPNELSMLGTVGPPVPNVDIRLESVPEMGYDALASNPRGEICIRGKTLFSGYYKREDLTQ
EVFIDGWLHTGDVGEWQPDGAMKIIDRKKNIFKLSQGEYVAVENLENIYSHVAAIESIWVYGNSYESYLV
AVVCPSKIQIEHWAKEHKVSGDFESICRNQKTKEFVLGEFNRVAKDKKLKGFELIKGVHLDTVPFDMERD
LITPSYKMKRPQLLKYYQKEIDEMYKKNREVQLRV
>AtLACS4 Long chain acyl-CoA synthetase 4 [*Arabidopsis thaliana*] AT4G23850
MSQQKKYIFQVEEGKEGSDGRPSVGPVYRSIFAKDGFPDPIEGMDSCWDVFRMSVEKYPNNPMLGRREIV
DGKPGKYVWQTYQEVYDIVMKLGNSLRSVGVKDEAKCGIYGANSPEWIISMEACNAHGLYCVPLYDTLGA
DAVEFIISHSEVSIVFVEEKKISELFKTCPNSTEYMKTVVSFGGVSREQKEEAETFGLVIYAWDEFLKLG
EGKQYDLPIKKKSDICTIMYTSGTTGDPKGVMISNESIVTLIAGVIRLLKSANEALTVKDVYLSYLPLAH
IFDRVIEECFIQHGAAIGFWRGDVKLLIEDLAELKPTIFCAVPRVLDRVYSGLQKKLSDGGFLKKFIFDS
AFSYKFGYMKKGQSHVEASPLFDKLVFSKVKQGLGGNVRIILSGAAPLASHVESFLRVVACCHVLQGYGL
TESCAGTFVSLPDELGMLGTVGPPVPNVDIRLESVPEMEYDALASTARGEICIRGKTLFSGYYKREDLTK
EVLIDGWLHTGDVGEWQPDGSMKIIDRKKNIFKLSQGEYVAVENIENIYGEVQAVDSVWVYGNSFESFLI
AIANPNQHILERWAAENGVSGDYDALCQNEKAKEFILGELVKMAKEKKMKGFEIIKAIHLDPVPFDMERD
LLTPTFKKKRPQLLKYYQSVIDEMYKTINAKFASRG
>AtLACS5 Long chain acyl-CoA synthetase 5 [*Arabidopsis thaliana*] AAM28872.1
MTSQKRFIFEVEAAKEATDGNPSVGPVYRSTFAQNGFPNPIDGIQSCWDIFRTAVEKYPNNRMLGRREIS
NGKAGKYVWKTYKEVYDIVIKLGNSLRSCGIKEGEKCGIYGINCCEWIISMEACNAHGLYCVPLYDTLGA
GAVEFIISHAEVSIAFVEEKKIPELFKTCPNSTKYMKTVVSFGGVKPEQKEEAEKLGLVIHSWDEFLKLG
EGKQYELPIKKPSDICTIMYTSGTTGDPKGVMISNESIVTITTGVMHFLGNVNASLSEKDVYISYLPLAH
VFDRAIEECIIQVGGSIGFWRGDVKLLIEDLGELKPSIFCAVPRVLDRVYTGLQQKLSGGGFFKKKVFDV
AFSYKFGNMKKGQSHVAASPFCDKLVFNKVKQGLGGNVRIILSGAAPLASHIESFLRVVACCNVLQGYGL
TESCAGTFATFPDELDMLGTVGPPVPNVDIRLESVPEMNYDALGSTPRGEICIRGKTLFSGYYKREDLTK
EVFIDGWLHTGDVGEWQPNGSMKIIDRKKNIFKLAQGEYVAVENLENVYSQVEVIESIWVYGNSFESFLV
AIANPAQQTLERWAVENGVNGDFNSICQNAKAKAFILGELVKTAKENKLKGFEIIKDVHLEPVAFDMERD
LLTPTYKKKRPQLLKYYQNVIHEMYKTTKETLASGQ
>AtLACS6 Long-chain acyl-CoA synthetase 6 [*Arabidopsis thaliana*] NP_566265.1
MDSSSSSSSAAARRRINAIHSHLVTSSRSSPLLRSNPTAGEFCLDNGYSVVLPEKLNTGSWNVYRSAKSP
FKLVSRFPDHPDIATLHDNFEHAVHDFRDYKYLGTRVRVDGTVGDYKWMTYGEAGTARTALGSGLVHHGI
PMGSSVGIYFINRPEWLIVDHACSSYSYVSVPLYDTLGPDAVKFIVNHATVQAIFCVAETLNSLLSCLSE
MPSVRLVVVVGGLIESLPSLPSSSGVKVVSYSVLLNQGRSNPQRFFPPKPDDVATICYTSGTTGTPKGVV
LTHANLIANVAGSSFSVKFFSSDVYISYLPLAHIYERANQILTVYFGVAVGFYQGDNMKLLDDLAALRPT
VFSSVPRLYNRIYAGIINAVKTSGGLKERLFNAAYNAKKQALLNGKSASPIWDRLVFNKIKDRLGGRVRF
MTSGASPLSPEVMEFLKVCFGGRVTEGYGMTETSCVISGMDEGDNLTGHVGSPNPACEVKLVDVPEMNYT

SADQPHPRGEICVRGPIIFTGYYKDEIQTKEVIDEDGWLHTGDIGLWLPGGRLKIIDRKKNIFKLAQGEY
IAPEKIENVYAKCKFVGQCFIYGDSFNSSLVAVVSVDPDVLKSWAASEGIKGGDLRELCNNPRVKAAVLS
DMDTVGREAQLRGFEFAKAVTLVLEPFTLENGLLTPTFKIKRPQAKEYFAEAITNMYKELGASDPSANRG
L
>AtLACS7 Long-chain acyl-CoA synthetase 7 [*Arabidopsis thaliana*] NP_198112.2
MEFASPEQRRLETIRSHIDTSPTNDQSSSLFLNATASSASPFFKEDSYSVVLPEKLDTGKWNVYRSKRSP
TKLVSRFPDHPEIGTLHDNFVHAVETYAENKYLGTRVRSDGTIGEYSWMTYGEAASERQAIGSGLLFHGV
NQGDCVGLYFINRPEWLVVDHACAAYSFVSVPLYDTLGPDAVKFVVNHANLQAIFCVPQTLNILLSFLAE
IPSIRLIVVVGGADEHLPSLPRGTGVTIVSYQKLLSQGRSSLHPFSPPKPEDIATICYTSGTTGTPKGVV
LTHGNLIANVAGSSVEAEFFPSDVYISYLPLAHIYERANQIMGVYGGVAVGFYQGDVFKLMDDFAVLRPT
IFCSVPRLYNRIYDGITSAVKSSGVVKKRLFEIAYNSKKQAIINGRTPSAFWDKLVFNKIKEKLGGRVRF
MGSGASPLSPDVMDFLRICFGCSVREGYGMTETSCVISAMDDGDNLSGHVGSPNPACEVKLVDVPEMNYT
SDDQPYPRGEICVRGPIIFKGYYKDEEQTREILDGDGWLHTGDIGLWLPGGRLKIIDRKKNIFKLAQGEY
IAPEKIENVYTKCRFVSQCFIHGDSFNSSLVAIVSVDPEVMKDWAASEGIKYEHLGQLCNDPRVRKTVLA
EMDDLGREAQLRGFEFAKAVTLVPEPFTLENGLLTPTFKIKRPQAKAYFAEAISKMYAEIAASNPIPSKL
>AtLACS8 Long-chain acyl-CoA synthetase 8 [*Arabidopsis thaliana*] AT2G04350
MEDSGVNPMDSPSKGSDFGVYGIIGGGIVALLVPVLLSVVLNGTKKGKKRGVPIKVGGEEGYTMRHARAP
ELVDVPWEGAATMPALFEQSCKKYSKDRLLGTREFIDKEFITASDGRKFEKLHLGEYKWQSYGEVFERVC
NFASGLVNVGHNVDDRVAIFSDTRAEWFIAFQGCFRQSITVVTIYASLGEEALIYSLNETRVSTLICDSK
QLKKLSAIQSSLKTVKNIIYIEEDGVDVASSDVNSMGDITVSSISEVEKLGQKNAVQPILPSKNGVAVIM
FTSGSTGLPKGVMITHGNLVATAAGVMKVVPKLDKNDTYIAYLPLAHVFELEAEIVVFTSGSAIGYGSAM
TLTDTSNKVKKGTKGDVSALKPTIMTAVPAILDRVREGVLKKVEEKGGMAKTLFDFAYKRRLAAVDGSWF
GAWGLEKMLWDALVFKKIRAVLGGHIRFMLVGGAPLSPDSQRFINICMGSPIGQGYGLTETCAGATFSEW
DDPAVGRVGPPLPCGYVKLVSWEEGGYRISDKPMPRGEIVVGGNSVTAGYFNNQEKTDEVYKVDEKGTRW
FYTGDIGRFHPDGCLEVIDRKKDIVKLQHGEYVSLGKVEAALGSSNYVDNIMVHADPINSYCVALVVPSR
GALEKWAEEAGVKHSEFAELCEKGEAVKEVQQSLTKAGKAAKLEKFELPAKIKLLSEPWTPESGLVTAAL
KIKREQIKSKFKDELSKLYA
>AtLACS9 Long-chain acyl-CoA synthetase 9 [*Arabidopsis thaliana*] AT1G77590
MIPYAAGVIVPLALTFLVQKSKKEKKRGVVVDVGGEPGYAIRNHRFTEPVSSHWEHISTLPELFEISCNA
HSDRVFLGTRKLISREIETSEDGKTFEKLHLGDYEWLTFGKTLEAVCDFASGLVQIGHKTEERVAIFADT
REEWFISLQGCFRRNVTVVTIYSSLGEEALCHSLNETEVTTVICGSKELKKLMDISQQLETVKRVICMDD
EFPSDVNSNWMATSFTDVQKLGRENPVDPNFPLSADVAVIMYTSGSTGLPKGVMMTHGNVLATVSAVMTI
VPDLGKRDIYMAYLPLAHILELAAESVMATIGSAIGYGSPLTLTDTSNKIKKGTKGDVTALKPTIMTAVP
AILDRVRDGVRKKVDAKGGLSKKLFDFAYARRLSAINGSWFGAWGLEKLLWDVLVFRKIRAVLGGQIRYL
LSGGAPLSGDTQRFINICVGAPIGQGYGLTETCAGGTFSEFEDTSVGRVGAPLPCSFVKLVDWAEGGYLT
SDKPMPRGEIVIGGSNITLGYFKNEEKTKEVYKVDEKGMRWFYTGDIGRFHPDGCLEIIDRKKDIVKLQH
GEYVSLGKVEAALSISPYVENIMVHADSFYSYCVALVVASQHTVEGWASKQGIDFANFEELCTKEQAVKE
VYASLVKAAKQSRLEKFEIPAKIKLLASPWTPESGLVTAALKLKRDVIRREFSEDLTKLYA

>BnGPAT1 Glycerol-3-phosphate acyltransferase 1 [*Brassica napus*] XP_013696751.1
MVFPELLVILAEWVLYRLLANSCYRAARKLRGYGFQLKNFLNLSKSQSQQNTSHLLNNQQQQQNQDSLN
PLFPSITKYEDTGCSVSPDDTLVCDIDGVLLRQLSSKHFHAFFPYFMLVAFEGGSIIRAIILLFSCSFLW
ALPQETKLKVLTFITFSGLKVKDMDNVSRSVLSKFFLESLNFQVYNVWARTEYSKVVFTSLPQVMVERFL
REHLNADDVIGTKLQQIEVMGRKVYTGLTSGSLVKHRAAKDYFDNNNKKKPVLGIGSSSSVQDHTLISIC
KEVYYVCNEEDSMNTLPRERYPKPLIFHDGRLAFLPTPSATLAMFTWLPIGFFLAVFRILIGVLLPYHVAN
FLAAISGVRITFKTHNLYNGPPEKGKSGVLYVCNHRTLLDPVFLTTSLGKPLTAVTYSLSKFSELIAPLK
TVSLKRDRKKDGEAMQRLLSKGDLVVCPEGTTCREPYLLRFSPLFAELTEDIVPVAVDARVSMFYGTTAS
GLKCLDPIFFLMNPRPVYCIEVLKKLPKEMTCAGGKSSFEVANFIQGELARVLGFECTNLTRKDKYLVLA
GNEGIVK
>BnGPAT2 Glycerol-3-phosphate acyltransferase 2 [*Brassica napus*] CDY15240.1

MLVAFETGGIIRSFLLFILYPFISLMSYELGMKTMVMVSFFGIKKEKFLLGTSVLPKYFLEDVGLEMFEV
LKRGGKRVGVSDLPQVMIDGFLEDYLGIEVVVGREMKMIGGYYSGILEDKKKHEFYLGELVQEEKLSSGH
VIGITSYNLPSHRSLFSQFCQEIYFVRNSDKKSWQTLPRNQYPRPLIFHDGRLAIKPTPTNTLALFMWAP
FAAVLATARTVAGLNLPYSLAIPFLAFSGFRLTLTVNNDLISPDQKEGCLFVCNHRTLLDPLYISYALRK
TNIKAVTYSLSRLSELLAPIKTVRLTRDRVQDGQAMKRLLRQGDLVVCPEGTTCREPYLLRFSPLFAEVS
DAIVPVAIDSHVTFFHGTTASGLKAFDPIFFLLNPYPTYTVRLLDPVFGGGSSTCRDPDDGKSKFEVANH
VQHEIGKALGFECTNLTRRDKYLILAGNNGVVDKK
>BnGPAT3 Glycerol-3-phosphate acyltransferase 3 [*Brassica napus*] CDY22016.1
MLVAFEAGGVIRSFILFILYPLITLMLSHEMGVKVMVMVSFFGIKKDGFRAGGAVLPKHFLEDVGLEMFK
VLRKGGKRIVVSYDLPQVMIEGFLRDYLEVEVVVGREMKVVGGYYLGIMEDKTKHDLVFDELVRKERLNT
GRVIGITSYNTSLHRYLFSQFCQEIYFVKRSDKRTWQTLPKSQYPKPLIFHDGRLAIRPTLMNTLALFMW
GPFAVLAAAARLFVSLCIPYTLSIPILSFFGCRLTVKIDDVSSQKLNSSERKGCLFACNHRTLLDPLYVG
FALKKKNIKTVTYSLSRVSEILAPIKTVRLTRDRVSDGQAMKKLLAEGDLVVCPEGTTCREPYLLRFSPL
FAEISDVIVPVAVTSPATFFYGTTASGLKAFDPLFFLMDPYPTYTVQFLDPVPGVTCQDPEGKLKFEVAN
HVQSAIGKALDFECTYLTRKDKYLILAGNNGVVKKN
>AhGPAT1 Glycerol-3-phosphate acyltransferase 1 [*Arachis hypogaea*] AER51964.1
MVFPMVLLRLLDWVLYQLLANSFYRAARKMKSYWFNFEYNIISSKPQHLQQPCLSFPSVTKCDLEALRAS
QPTLAFCDVHKVLLNTHSFFPFFMLVAFEGGSILRALLLLLSCPLLLILNHDMNLKLMTFITFCGLRIKD
MECVSRAVLPKFYLENLNLHAYEVMASSSSKVVFTSVPRVMVEGFLKEYLSVCNVIGTELQTFGTYFTGF
VSDSGLLVKHRALKEYFGDRKPDIGIGSSSFDDHIFMSLCKEGYVVNNPSPMLGREKYPKPLIFHDGRLA
FLPTPSATLLMFMWLPIGILLAIYRILVGILLPYKWAMILGVWSGINLNVKGIMRSQSSEQKKGVLYVCT
HRTLLDPVFLSTSLAKPLTAVTYSLSKVSEFIAPIRTVRLTRDRKQDAQTMQRLLSEGDLVVCPEGTTCR
EPYLLRFSSLFAELADEIVPVAMNAHVSMFYGTTAAGLKCLDPIFFFMNPRPCYHIHILGKVPKELTCAG
GRSSCEVANYIQSQLALALGFQCTTLTRRDKYLMLAGNEGIVNQQRNTKINHHCTNNNKGETLLS
>AhGPAT2 Glycerol-3-phosphate acyltransferase 2 [*Arachis hypogaea*] AER42344.1
MAKMFRAFFFKSLFFFWYRFLFRQLKSLTGLRRNILSTQFKYQKFSSFLHQHHRSSELGDHTLVFDVENA
LLKSSSLFPYFMLVAFEAGGLIRAIVLVLLYPVACAPDIVGQELGLKMMVMICFFGIKVDSFRVGRSVLP
KFLLEDVGKEMFDVLKRSSGKKVGVTNMPRIMVESFLREYLEIDVVVGRELKVFCGYFVGLMEEKKSALH
ALEQVQEGKGCSDMIGITRFNKVVDHQLFSHCKEVYAVSEAEKRSWQRLSKDKYPKPLIFHDGRLALRPT
LFDSIAILMWLPYALILAIIRISLALSLPYNFSTPLLVFTGIHLTTSEIPKMPPNNNNKTGTLYVCNHRT
LLDPLYISFTLQRNLIAVTYSLSRMSEILAPIKTVRLTRNRDQDANMMKHLLAQGDLVVCPEGTTCREPY
LLRFSPLFSEMCDDIAPVAVNSHVTMFHGTTAGGLKCLDPVFFLMNPSPVYTVDLLQHVQRSRCVDRAVV
TEKEHQARFKVANHVQSQIGSALGFECTKLTRKDKYLILAGNEGVVINRACGKS
>AhGPAT6 Glycerol-3-phosphate acyltransferase 6 [*Arachis hypogaea*] AER42345.1
MVMGAFSHFEQVSKCNTEGRSNQTVASDLDGTLLVSRSAFPYYMLVAIEAGSLLRALILLISVPFVYFTY
IFISETIAIKTLIFVTFAGLKIRDVEMVARSVLPKFYAEDVHPVTWKVFNSFGKRYIVTASPRLMVEPFV
KTLLGADKVLGTELEATKSGRLTGFVKEPGVLVGEHKKTVLVKEFQSNLPDLGLGDSETDHDFMSICKEG
YMVPRTKCEALARNKLISPIIFHEGRFVQRPTPLASLLTFLWLPIGIILSILRVYLNIPLPERIAWYNYK
LLGIRVTVKGTPPPPPKKGQSGVLFVCNHRTVLDPVVTAVALGRKISCVTYSISKFTEIISPIKAVALSR
EREKDAENIKRLLEEGDLVICPEGTTCREPFLLRFSALFAELTDRIVPVAINTKQSVFYGTTVRGHKLLD
PYFVFMNPMPTYEITFLNQLPKELTCSGGKSAIEVANYIQRVLAGTLGFECTNLTRKDKYAMLAGTDGRV
ASSNKENKA
>AhGPAT4 Glycerol-3-phosphate acyltransferase 4 [*Arachis hypogaea*] AGR66149.1
MAAPKPKRTFPPVTECNGNSPSSPRESVAADLDGTLLISRSSFPYFMLVAVEAGSLLRGFILLLSVPFVI
IAYLFISEAIGIQILIFISFAGLKIRDIELASRAVLPRFYSADVRKESFEVFDSCKRKVVVTANPTVMVE
PFVKDFLGGDKVLGTEIEVNPKTKKATGFVKKPGVLVGKLKRLAILKEFGETTSPDIGLGDRESDHDFMS
ACKEGYMVPPSKSAKPVPRDRLKSRLIFHDGRFVQRPDPLNALFTFLWIPFGFALSIFRVYFNLPLPERI
VRYTYELLGIHLVISGHRPPPPSPGTPGNLYVCNHRTALDPIVIAIALGRKVSCVTYSVSKLSRFLSPIP
AVALTRDRAADAARIKDLLQKGDLVVCPEGTTCREPFLLRFNALFAELSDRIVPVAVDCKQSMFFGTTVR
GVKFWDPYFFFMNPRPVYEIRFLDRLPEEMSCKAGGKSSIEVANHVQKVLGDVLGFECAGLTRKDKYMLL
GGNDGKVESMYSSKK

>AhGPAT9 Glycerol-3-phosphate acyltransferase 9 [*Arachis hypogaea*] AGR66148.1
MMRKTNPKSQSTELELDQPNIEDYLPSGHTIHQEPHGKLRLCDLLDISPTLSEAAGAIVDDSFLRCFKSI
RSEPWNWNVYLFPLWCFGVVIRYLILFPARVLLLSFGWMIFLSAFIPVHLLLKRDNSVRKNIERCLVEMI
CGFFVASWTGVVKYHGPRPSMRPKQVFVANHTSMIDFIILEQMTAFAVIMQKHPGWVGLLQNTILESVGC
IWFNRTEAKDREIVARKLREHVQGADNNPLLIFPEGTCVNNHYSVMFKKGAFELGCTVCPVAIKYNKIFV
DAFWNSRKQSFTKHLLQLMTSWAVVCDVWYLEPQNQKPGETSIEFAERVREIISQRAGLKMVPWDGYLKY
SRPSPKLREGKQRNFVESVLRRLEEK
>AtATS1 Phospholipid/glycerol acyltransferase family protein [*Arabidopsis thaliana*] AT1G32200
MTLTFSSSAATVAVAAATVTSSARVPVYPLASSTLRGLVSFRLTAKKLFLPPLRSRGGVSVRAMSELVQD
KESSVAASIAFNEAAGETPSELSHSRTFLDARSEQDLLSGIKKEAEAGRLPANVAAGMEELYWNYKNAVL
SSGASRADETVVSNMSVAFDRMLLGVEDPYTFNPYHKAVREPFDYYMFVHTYIRPLIDFKNSYVGNASIF
SELEDKIRQGHNIVLISNHQSEADPAVISLLLEAQSPFIGENIKCVAGDRVITDPLCKPFSMGRNLICVY
SKKHMNDDPELVDMKRKANTRSLKEMATMLRSGGQLIWIAPSGGRDRPNPSTGEWFPAPFDASSVDNMRR
LVEHSGAPGHIYPMSLLCYDIMPPPPQVEKEIGEKRLVGFHGTGLSIAPEINFSDVTADCESPNEAKEAY
SQALYKSVNEQYEILNSAIKHRRGVEASTSRVSLSQPWN
>AtGPAT1 Glycerol-3-phosphate acyltransferase 1 [*Arabidopsis thaliana*] NP_563768.1
MVLPELLVILAEWVLYRLLAKSCYRAARKLRGYGFQLKNLLSLSKTQSLHNNSQHHLHNHHQQNHPNQTL
QDSLDPLFPSLTKYQELLLDKNRACSVSSDHYRDTFFCDIDGVLLRQHSSKHFHTFFPYFMLVAFEGGSI
IRAILLLLLSCSFLWTLQQETKLRVLSFITFSGLRVKDMDNVSRSVLPKFFLENLNIQVYDIWARTEYSKV
VFTSLPQVLVERFLREHLNADDVIGTKLQEIKVMGRKFYTGLASGSGFVLKHKSAEDYFFDSKKKPALGI
GSSSSPQDHIFISICKEAYFWNEEESMSKNNALPRERYPKPLIFHDGRLAFLPTPLATLAMFIWLPIGFL
LAVFRISVGVFLPYHVANFLASMSGVRITFKTHNLNNGRPEKGNSGVLYVCNHRTLLDPVFLTTSLGKPL
TAVTYSLSKFSEFIAPLKTVSLKRDRKKDGEAMQRLLSKGDLVVCPEGTTCREPYLLRFSPLFAELTEDI
VPVAVDARVSMFYGTTASGLKCLDPIFFLMNPRPVYCLEILKKLPKEMTCAGGKSSFEVANFIQGELARV
LGFECTNLTRRDKYLVLAGNEGIVR
>AtGPAT2 Glycerol-3-phosphate acyltransferase 2 [*Arabidopsis thaliana*] AT1G02390
MSGNKISTLQALVFFLYRFFILRRWCHRSPKQKYQKCPSHGLHQYQDLSNHTLIFNVEGALLKSNSLFPY
FMVVAFEAGGVIRSLFLLVLYPFISLMSYEMGLKTMVMLSFFGVKKESFRVGKSVLPKYFLEDVGLEMFQ
VLKRGGKRVAVSDLPQVMIDVFLRDYLEIEVVVGRDMKMVGGYYLGIVEDKKNLEIAFDKVVQEERLGSG
RRLIGITSFNSPSHRSLFSQFCQEIYFVRNSDKKSWQTLPQDQYPKPLIFHDGRLAVKPTPLNTLVLFMW
APFAAVLAAARLVFGLNLPYSLANPFLAFSGIHLTLTVNNHNDLISADRKRGCLFVCNHRTLLDPLYISY
ALRKKNMKAVTYSLSRLSELLAPIKTVRLTRDRVKDGQAMEKLLSQGDLVVCPEGTTCREPYLLRFSPLF
SEVCDVIVPVAIDSHVTFFYGTTASGLKAFDPIFFLLNPFPSYTVKLLDPVSGSSSSTCRGVPDNGKVNF
EVANHVQHEIGNALGFECTNLTRRDKYLILAGNNGVVKKK
>AtGPAT3 Glycerol-3-phosphate acyltransferase 3 [*Arabidopsis thaliana*] AT4G01950
MHCLKPNKENTRRKEKKSKKKSFLQNPNMSAKISIFQALVFLFYRFILRRYRNSKPKYQNGPSSLLQSDL
SRHTLIFNVEGALLKSDSLFPYFMLVAFEAGGVIRSFLLFILYPLISLMSHEMGVKVMVMVSFFGIKKEG
FRAGRAVLPKYFLEDVGLEMFEVLKRGGKKIGVSDDLPQVMIEGFLRDYLEIDVVVGREMKVVGGYYLGI
MEDKTKHDLVFDELVRKERLNTGRVIGITSFNTSLHRYLFSQFCQEIYFVKKSDKRSWQTLPRSQYPKPL
IFHDGRLAIKPTLMNTLVLFMWGPFAAAAAAARLFVSLCIPYSLSIPILAFSGCRLTVTNDYVSSQKQKP
SQRKGCLFVCNHRTLLDPLYVAFALRKKNIKTVTYSLSRVSEILAPIKTVRLTRDRVSDGQAMEKLLTEG
DLVVCPEGTTCREPYLLRFSPLFTEVSDVIVPVAVTVHVTFFYGTTASGLKALDPLFFLLDPYPTYTIQF
LDPVSGATCQDPDGKLKFEVANNVQSDIGKALDFECTSLTRKDKYLILAGNNGVVKKN
>AtGPAT4 Glycerol-3-phosphate acyltransferase 4 [*Arabidopsis thaliana*] AT1G01610
MSPAKKSRSFPPISECKSREYDSIAADLDGTLLLSRSSFPYFMLVAIEAGSLFRGLILLLSLPIVIIAYL
FVSESLGIQILIFISFAGIKIKNIELVSRAVLTRFYAADVRKDSFEVFDKCKKRKVVVTANPIVMVEPFV
KDYLGGDKVLGTEIEVNPKTMKATGFVKKPGVLVGDLKRLAILKEFGDDSPDLGLGDRTSDHDFMSICKE
GYMVHETKSATTVPIESLKNRIIFHDGRLVQRPTPLNALIIYLWLPFGFMLSVFRVYFNLPLPERFVRYT
YEILGIHLTIRGHRPPPPSPGKPGNLYVLNHRTALDPIIIAIALGRKITCVTYSVSRLSLMLSPIPAVAL
TRDRVADAARMRQLLEKGDLVICPEGTTCREPYLLRFSALFAELSDRIVPVAMNCKQGMFNGTTVRGVKF
WDPYFFFMNPRPSYEATFLDRLPEEMTVNGGGKTPFEVANYVQKVIGGVLGFECTELTRKDKYLLLGGND

GKVESINKTKSME
>AtGPAT5 Glycerol-3-phosphate acyltransferase 5 [*Arabidopsis thaliana*] NP_187750.1
MVMEQAGTTSYSVVSEFEGTILKNADSFSYFMLVAFEAAGLIRFAILLFLWPVITLLDVFSYKNAALKLK
IFVATVGLREPEIESVARAVLPKFYMDDVSMDTWRVFSSCKKRVVVTRMPRVMVERFAKEHLRADEVIGT
ELIVNRFGFVTGLIRETDVDQSALNRVANLFVGRRPQLGLGKPALTASTNFLSLCEEHIHAPIPENYNHG
DQQLQLRPLPVIFHDGRLVKRPTPATALIILLWIPFGIILAVIRIFLGAVLPLWATPYVSQIFGGHIIVK
GKPPQPPAAGKSGVLFVCTHRTLMDPVVLSYVLGRSIPAVTYSISRLSEILSPIPTVRLTRIRDVDAAKI
KQQLSKGDLVVCPEGTTCREPFLLRFSALFAELTDRIVPVAMNYRVGFFHATTARGWKGLDPIFFFMNPR
PVYEITFLNQLPMEATCSSGKSPHDVANYVQRILAATLGFECTNFTRKDKYRVLAGNDGTVSYLSLLDQL
KKVVSTFEPCLH
>AtGPAT6 Glycerol-3-phosphate acyltransferase 6 [*Arabidopsis thaliana*] NP_181346.1
MGAQEKRRRFEQISKCDVKDRSNHTVAADLDGTLLISRSAFPYYFLVALEAGSLLRALILLVSVPFVYLT
YLTISETLAINVFVFITFAGLKIRDVELVVRSVLPRFYAEDVRPDTWRIFNTFGKRYIITASPRIMVEPF
VKTFLGVDKVLGTELEVSKSGRATGFTRKPGILVGQYKRDVVLREFGGLASDLPDLGLGDSKTDHDFMSI
CKEGYMVPRTKCEPLPRNKLLSPIIFHEGRLVQRPTPLVALLTFLWLPVGFVLSIIRVYTNIPLPERIAR
YNYKLTGIKLVVNGHPPPPPKPGQPGHLLVCNHRTVLDPVVTAVALGRKISCVTYSISKFSELISPIKAV
ALTRQREKDAANIKRLLEEGDLVICPEGTTCREPFLLRFSALFAELTDRIVPVAINTKQSMFNGTTTRGY
KLLDPYFAFMNPRPTYEITFLKQIPAELTCKGGKSPIEVANYIQRVLGGTLGFECTNFTRKDKYAMLAGT
DGRVPVKKEKT
>AtGPAT7 Glycerol-3-phosphate acyltransferase 7 [*Arabidopsis thaliana*] NP_196227.1
MESSTTTSYSVVSELEGTLLKNPKPFAYFMLVAFEASGLIRFATLLFLWPIIALLDVLGYRNGSLKLMIF
VATAGLHESEIESVARAVLPKFFMDDISMDAWRAFGSCDKRVVVTRMPRVMVERFAKDHLSADEVIGTEI
VVNRFGYATGLIQETNVDQSVFNSVANLFVDRRPQLGLGRHIISDSPTFLSLCEEQVHAPVPSNYNGHNQ
RLHVQPLPVIFHDGRLVKLPTPATALIILLWIPFGIILAMIRIFVGFLLPLWAIPYVSRIFNTRFIVKGK
PPAQATTGNPGVLFVCTHRTLMDPVVLSYVLGRSIPAVTYSISRLSEILSPIPTFRLTRIRDVDAEMIKK
ELSNGDLVVYPEGTTCREPFLLRFSALFAELTDNIVPVAMNYRVGFFHATTARGWKGLDPIFFFMNPRPV
YEVTFLNQLEVEATCSSGKSPYDVANYVQRILAATLGFECTNFTRKDKYRVLAGNDGTVSYLSFLDQVKK
VVTTFKPFLH
>AtGPAT8 Glycerol-3-phosphate acyltransferase 8 [*Arabidopsis thaliana*] NP_191950.2
MSPEKKSQNFPPITECRDGEYDSIAADLDGTLLLSRSSFPYFMLVAVEAGSLLRGLILLLSLPFVIISYL
FVSESLGIQILIFISFAGLKIRDIELVSRAVLPRFYAADVRKDSFEVFDKCKRKVVVTANPIVMVEAFVK
DYLGGDKVLGTEIEVNPKTNRATGFVKKPGVLVGDLKRLAILKEFGNESPDLGLGDRTSDHDFMSLCKKG
YMVHATKSATTIPKERLKNRIVFHDGRLAQRPTPLNAIITYLWLPFGFILSIIRVYFNLPLPERFVRYTY
EMLGIHLTIRGHRPPPPSPGTLGNLYVLNHRTALDPIIVAIALGRKICCVTYSVSRLSLMLSPIPAVALT
RDRATDAANMRKLLEKGDLVICPEGTTCREEYLLRFSALFAELSDRIVPVAMNCKQGMFNGTTVRGVKFW
DPYFFFMNPRPSYEATFLDRLPEEMTVNGGGKTPIEVANYVQKVIGAVLGFECTELTRKDKYLLLGGNDG
KVESINNTKK
>AtGPAT9 Glycerol-3-phosphate acyltransferase 9 [*Arabidopsis thaliana*] At5g60620
MSSTAGRLVTSKSELDLDHPNIEDYLPSGSSINEPRGKLSLRDLLDISPTLTEAAGAIVDDSFTRCFKSN
PPEPWNWNIYLFPLYCFGVVVRYCILFPLRCFTLAFGWIIFLSLFIPVNALLKGQDRLRKKIERVLVEMI
CSFFVASWTGVVKYHGPRPSIRPKQVYVANHTSMIDFIVLEQMTAFAVIMQKHPGWVGLLQSTILESVGC
IWFNRSEAKDREIVAKKLRDHVQGADSNPLLIFPEGTCVNNNYTVMFKKGAFELDCTVCPIAIKYNKIFV
DAFWNSRKQSFTMHLLQLMTSWAVVCEVWYLEPQTIRPGETGIEFAERVRDMISLRAGLKKVPWDGYLKY
SRPSPKHSERKQQSFAESILARLEEK

>BnLPAAT1 1-acyl-sn-glycerol-3-phosphate acyltransferase 1 [*Brassica napus*] Q9LLY4.2
MDVASAPGVSSHPPYYSKPICSSQSSLIRIPINKGCCFARSSNLITSLHAASRGVTRRTSGVQWCYRSIR
FDPFKVNDKNSRTVTVRSDLSGAATPESTYPEPEIKLSSRLRGICFCLVAGVSAIVLIVLMITGHPFVLL
FDRYRRKFHHFIAKLWASISIYPFYKTDIQGLENLPSSDTPCVYVSNHQSFLDIYTLLSLGQSYKFISKT
GIFVIPVIGWAMSMMGVVPLKRMDPRSQVDCLKRCMELVKKGASVFFFPEGTRSKDGRLGPFKKGAFTIA

AKTGVPVVPITLMGTGKIMPTGSEGILNHGDVRVIIHKPIYGSKADLLCDEARNKIAESMNLVS
>BnLPAAT2-1 1-acyl-sn-glycerol-3-phosphate acyltransferase 2 [*Brassica napus*] Q9XFW4.1
MAMAAAVIVPLGILFFISGLVVNLLQAVCYVLVRPMSKNTYRKINRVVAETLWLELVWIVDWWAGVKIQV
FADDETFNRMGKEHALVVCNHRSDIDWLVGWILAQRSGCLGSALAVMKKSSKFLPVIGWSMWFSEYLFLE
RNWAKDESTLQSGLQRLNDFPRPFWLALFVEGTRFTEAKLKAAQEYAASSELPVPRNVLIPRTKGFVSAV
SNMRSFVPAIYDMTVAIPKTSPPPTMLRLFKGQPSVVHVHIKCHSMKDLPEPEDEIAQWCRDQFVAKDAL
LDKHIAADTFPGQKEQNIGRPIKSLAVVVSWACLLTLGAMKFLHWSNLFSSWKGIALSAFGLGIITLCMQ
ILIRSSQSERSTPAKVAPAKPKDNHQSGPSSQTEVEEKQK
>BnLPAAT2-2 1-acyl-sn-glycerol-3-phosphate acyltransferase 2-like [*Brassica napus*] NP_001302981.1
MAMAAAVIVPLGILFFISGLVVNLLQAICYVLIRPLSKNTYRKINRVVAETLWLELVWIVDWWAGVKIQV
FADNETFNRMGKEHALVVCNHRSDIDWLVGWILAQRSGCLGSALAVMKKSSKFLPVIGWSMWFSEYLFLE
RNWAKDESTLKSGLQRLNDFPRPFWLALFVEGTRFTEAKLKAAQEYAASSELPVPRNVLIPRTKGFVSAV
SNMRSFVPAIYDMTVAIPKTSPPPTMLRLFKGQPSVVHVHIKCHSMKDLPESDDAIAQWCRDQFVAKDAL
LDKHIAADTFPGQQEQNIGRPIKSLAVVLSWSCLLILGAMKFLHWSNLFSSWKGIAFSALGLGIITLCMQ
ILIRSSQSERSTPAKVVPAKPKDNHNDSGSSSQTEVEKQK
>BnLPAAT2-3 1-acyl-sn-glycerol-3-phosphate acyltransferase 2 [*Brassica napus*] AXX75484.1
MAMAAAVIVPLGILFFISGLVVNLLQAVCYVLVRPMSKNTYRKINRVVAETLWLELVWIVDWWAGVKIQV
FADDETLNRMGKEHALVVCNHRSDIDWLVGWILAQRSGCLGSALAVMKKSSKFLPVIGWSMWFSEYLFLE
RNWAKDESTLQSGLQRLNDFPRPFWLALFAEGTRFTEAKLKAAQEYAASSELPVPRNVLIPRTKGFVSAV
SNMRSFVPAIYDMTVAIPKTSPPPTMLRLFKGQPSVVHVHIKCHSMKDLPEPEDEIAQWCRDQFVAKDAL
LDKHIAADTFPGQKEQNIGRPIKSLAVVVSWACLLTLGAMKFLHWSNLFSSWKGIALSAFGLGIITLCMQ
ILIRSSQSERSTPAKVAPAKPKDNHQSGPSSQTEVEEKQK
>BnLPAAT2-4 NP_001302955.1 1-acyl-sn-glycerol-3-phosphate acyltransferase 2 [*Brassica napus*]
MAMAAAAVIVPLGILFFISGLVVNLLQAVCYVLIRPLSKNTYRKINRVVAETLWLELVWIVDWWAGVKIQ
VFADDETFNRMGKEHALVVCNHRSDIDWLVGWILAQRSGCLGSALAVMKKSSKFLPVIGWSMWFSEYLFL
ERNWAKDESTLKSGLQRLNDFPRPFWLALFVEGTRFTEAKLKAAQEYAASSQLPVPRNVLIPRTKGFVSA
VSNMRSFVPAIYDMTVAIPKTSPPPTMLRLFKGQPSVVHVHIKCHSMKDLPESDDAIAQWCRDQFVAKDA
LLDKHIAADTFPGQKEHNIGRPIKSLAVVVSWACLLTLGAMKFLHWSNLFSSLKGIALSALGLGIITLCM
QILIRSSQSERSTPAKVAPAKPKDKHQSGSSSQTEVEEKQK
>BnLPAAT4-2 Lysophosphatidyl acyltransferase 4-2 [*Brassica napus*] AHV90569.1
MEVCGDSDNLKNRHLTPLRTLRGLIILLIFLSTAFMFLIYFAPPFALALRLLSVHQSRKSISFIFGHWLA
LWPYLFEKVNGTTVIFSGDTLPVEKRVLLIANHRTEVDWMYLWNIALRKGCLGYIKYVLKSSLMRLPIFG
WGFHVLEFIPVERKREVDEPVMLQMLSTFKDPREPLWLALFPEGTDFTEEKCKRSQKFAAEAGLPTLSNV
LLPKTRGFSVCLEALHNSLDAVYDLTIAYKPRCPSFMDNVFGTDPSEVHIHVRRVLAKEIPASEAGSSAW
LMDSFKSKDKLLSDFNAQGQFPNQRPEEELSILKCIATFGVIVSLTVLFLYLTLYSHSCFKVYVGLSFTY
LSFATYYKFRPSPSVGSCKGGSCKESKTH
>BnLPAAT4-1 Lysophosphatidyl acyltransferase 4-1 [*Brassica napus*] AHV90568.1
MEVTGDSDNLKNRPLTPLRTLRGLIILLIFLSTAFMFLIYFAPPFALALRLLSVHQSRKSISFIFGHWLA
LWPYLFETINGTTVIFSGDTLPVEKRVLLIANHRTEVDWMYLWNIALRKGCLGYIKYVLKSNLMRLPIFG
WGFHVLEFIPVERKREVDEPVMLQMLSSFKDPREPLWLALFPEGTDFTEEKCKRSQKFAAEAGLPTLSNV
LLPKTRGFSVCLDALHNSLDAVYDLTIAYKPRCPSFMDNVFGTDPSEVHIHVKRVLTKEIPASEAESSAW
LMDSFKSKDRLLSDFNAQGQFPNQRPEEELSILKCIATFGVIVSLTVLFLYLTLYSHSCFKVYVGLSFTY
LSFATYYKFRPSPSVGCCKGGSSCKEAKTH
>BnLPAAT5 Probable 1-acyl-sn-glycerol-3-phosphate acyltransferase 5 [*Brassica napus*] XP_013749364.1
MEKKLKNSNNLSLIRMLRGIICLMVLVSTAFMMLIFWGFLSAVVMRLFSLHYSRKCVYFFFGSWLALWPF
LFEKINRTKVIFSGDKVPCESRVLLIANHRTEVDWMYFWDLALRKGQIGNMKYVLKSSLMKLPLFGWAFH
LFEFIPVERKWKVDEANLRQMVSSFKDPRDGLWLGLFPEGTDYTEAKCERSKKFAAENGLPELNNVLLPK
TKGFVSCLEELDSSLDAVYDVTIGYKTRCPSFLDNVYGTEPSEVHIHIRRISQNQIPNEEKEINAWLMNT
FQIKDQLLSEFYSRGHFPNEGTEKEFSTIKQLINCLAVIVFTIICTHLTFFSSMIWFKIYVSLVCAYLTY
ATHFNLRPAPLVETAKKSFYSKKLNF

>RcLPAATB B-class lysophosphatidate acyltransferase [*Ricinus communis*] AFR42414.1
MESTGGGSFMRNRRLESFLNTDSSTNVNETSRKLVRKEVEQRPNNDVYVDDDGWISGLISWVRIVVCFVS
MMITTFIWASLMLLLLPWPYERIRQGNIYGHVTGRILMWILGNPLKIEGVEFSNEKAIYISNHSSPIDIF
LIMWLTPTGTVGVAKKEIIWYPLFGQLYVLANHLRIDRSNPAAAIQSMKEVACAVIKNNLSLIIFPEGTR
SKSGRLLPFKKGFVHLALQTRLPIVPIVLTGTHRAWRKDSLHVRPAPINVKYLRPIKTDNWTDDKVDDYV
KLLHDMYVENLPESQRPLS
>AhLPAATB Lysophosphatidyl acyltransferase 6 [*Arachis hypogaea*] AGT57763.1
MENSGGGALLRNRRLESFLDTSSGLRVEEAPKILVKEKPELWLKKDVYMDDNGWVSSIISWIRIVTCFVS
MMVTTFIWALIMVVLIPWPYERIRQGNIYGHVTGRMLMWILGNPIKIEGAEYNNERAIYISNHASPIDIF
LIMWLTPTGTVGIAKKEIVWYPLFGQLYVLANHLRIDRSNPTSAIESMNEVARAVVRNNLSLIIFPEGTR
SKNGRLLPFKKGFVHLALQTRLPIVPMVLTGTHQAWRKGSLHVRPTPLAVKYLPPISTENWKAEKIDDYV
KMVHDLYAAQLPETQRPLA
>GmLPAAT3 1-acyl-sn-glycerol-3-phosphate acyltransferase 3 [*Glycine max*] XP_025981491.1
MGAKHFLEKIIGYVGGEIADEDGVFQGGIGPALADAEGGPIEPEGLVGGVVGLENPFRCGVGDELDEAVA
LGLARELVADDFDGDHLAGGGEAVAQAVFFVLLRPLSKNCYSRINKLLTESLWLELIWLIDWWAGIKIEL
YTDSETLQLMGKKILGKENALVICNHRSDIDWLIGWVLAQRSGCLGSTVAIMKKEVKFLPVLGWSMWFAE
YIFLERDWAKDETSLKSGFRHLEHMPFPFWLALFVEGTRFTQTKLLQAQEFAASKGLPIPRNVLIPRTKG
FVTAVQSLRPFVPAIYDCTYAVPKSEASPTLVRIFKGISCPVKVQIKRHKMEELPETDDGIGQWCKDAFV
AKDALLEKYSTTEIFSEQDLQQIRRHKTSILVVVCWLCLLGFLVYEFFQWTSLLSSWEGILFTVLFLLLV
TVIMEIFIHSSQSERSKPPMVLPTQDPMKQKLLQT
>BrLPAAT3 1-acyl-sn-glycerol-3-phosphate acyltransferase 3 [*Brassica rapa*] XP_009144912.1
MAIPAALVFVPVGVLFLVSGLIVNLIQLVFFIIVRPFSKSLYRRINKNVVELLWLQLIWLIDWWACIKVN
IYADAETLQLLGKEHALVLSNHRSDIDWLIGWVMAQRAGCLGSSLAIMKKEAKYLPIIGWSMWFSDYIFL
ERSWDKDEKTLTAGFKRFEDFPMTFWLALFVEGTRFTQEKLEAAQEYASIRSLPSPRNVLIPRTKGFVSA
VSHIRSFVPAVYDCTLTVRNNQPKPTLLRMFSGQSSELNLQLRRHKMSDLPETDDGIAQWCQDLFITKDA
QLETYFTKDVFSDLDVHQINRPIKPLIVVIVWVCLLMYGGFKLLQWLSMVASWEIICLFVVILVIATITM
QVLIQSSESHRSTPAKRPLQEQLISA
>AhLPAAT1 1-acyl-sn-glycerol-3-phosphate acyltransferase 1, [*Arachis hypogaea*] XP_025672028.1
MSSVHALLSTSLSQSPLSSMFFNSSPSMEMFQLCTYKRPTYDHRVLRTSLSYAQRRLTGIPRKHRNDLWV
SFCPNEELCRPRYSCNLRYQNKHARDTFARCELAAAGDSHSLSADLKMESKVRGACFYAVTALSAVFLFV
LMLVGHPFVLLFDRQRRKFHHYVAKVWASLTVAPFYRIKFEGLENLPPPDTPAVYVSNHQSFLDIYTLLT
LGRSFKFISKTGIFLFPIIGWAMFLLGTIPLKRMDSRSQMDCLKRCMDLIRKGASVFVFPEGTRSKDGKL
GAFKKGAFSIAAKTNAPVVPITLIGTGQIMPAGREGIVNIGWVKVVIHKPLRGKDAEMLCKEARTTIAGV
L
>AhLPAAT2 1-acyl-sn-glycerol-3-phosphate acyltransferase 2 [*Arachis hypogaea*] XP_025604428.1
MGIAAAAVVVPFGLLFFTSGLAVNLIQAVSYVLVRPLSKNLHRRINRVVAELLWLELVWLIDWWAGVKVE
VFTDHETFQLMGKEHALVICNHRSDIDWLVGWVLAQRSGCLGSTLAVMKKSSKFLPVIGWSMWFSEYLFL
ERSWAKDESTLKSGIQRLSDFPLPFWLALFVEGTRFTQAKLLAAQEYAASAGLPIPRNVLIPRTKGFVSA
VSHMRSFVPAIYDVTVAIPKCSPTPTMLRLLKGQPSVVQVHIKRHLMNELPETDEAVAQWCRDVFVAKDA
LLDKHIAEDTFSDKELQDTGRPKKSLLVTISWSGLIVTGAVKFLRWTSLLSSWKGIAISTASLAVVTGLM
QILIMFSQSERSTPAKVLPAKPKSTGEQVEDKNVKQQ
>AhLPAAT3 1-acyl-sn-glycerol-3-phosphate acyltransferase 3 [*Arachis hypogaea*] XP_025613857.1
MAFPAAVFILPAGTLFILSGLIVNAIQIVVFILFRPISRNLFRRINNMLTQSLWLELIFLIDWWGAIKIE
LHMDSESRQLMGKENALLLCNHRSDIDWLIGFALAQRSGCLGSSLAIMKKELKYLPVIGWSMWFCDYIFL
EREWAKDERTLSSGLKQLENSPLPFWLALFVEGTRFTPDKLLAAQAFAASRGLPIPKNVLIPRTKGIVCV
VENIRSFTPAIYDCTYSVKKGDTPPTMLRLFKGQSSSVKLQIQRLKMEDLPHTPNEIAEWCKDLFISKDA
MLERYNTTDVFSDAELQDLGRSKKSIFVVLCWTCLLGFLLYEFFKSTKLLSTWEGILCTVLFFVVVTLVM
ELLIHATQSERSTRMNLPTQDPLRQKLLQ
>RcLPAAT1 1-acyl-sn-glycerol-3-phosphate acyltransferase 1, [*Ricinus communis*] XP_015580987.1

MELAFHPTHSNPLRLCYKRSSVHALSSSSLLSSVHKGLHVGSSPVKRAIMRSTNNSAFSGSLCIPRKLVA
VPWAEELNWPHFDSKLHKRNKLPRKTVVRSELVGNANPESAHPLSDIKLGAKVRGICFYAVTAIAAIFLF
VLMLVQHPFVLLLDRYRRKTQFFIAKIWAMITVAPFFKIEFEGLENLPSPDAPAVYVSNHQSFLDIYTLL
TLGRSFKFISKTGIFLFPIIGWAMFMLGVIPLKRMDSRSQLECLKRCIELVKKGTSVFFFPEGTRSKDGK
LGIFKKGAFSVAAKTKVPVVPITLMGTGKIMPAGMESILNLGSVKVVIHKPVEGSNPEVLCNEVRNTIAS
VLNQQS
>RcLPAAT2 A-class lysophosphatidate acyltransferase-2 [*Ricinus communis*] AFR42413.1
MAVAAVAVILPLGVLFFLSGLVVNLFQAICFVLIRPISKSSYRMINRALAELLWLELVWLVDWWAKIKIQ
VYTDRETLCLMGKEHALILANHRSDIDWLVGWMLAQRAGCLGSALAVMKKSSKFLPVIGWSMWFSEYLFL
ERSWAKDESTLKSGIQRLKDFPRPFWLGLFVEGTRFTKAKLLAAQQYAASQGLPIPRNVLIPRTKGFVSA
VSNMRSFVPAIYDVTVAIPKNSPQPTMLRLFKGQSSVVHVHIKRHLMKDLPETDDAVAQWCKDLFVAKDA
LLDKHIAEDTFSEQELQDIGRPKKSLVVVTLWSCLLIFGTLKFLQWSSLLSSWKGIALSMSALAIVTVLM
HILIRFSQSEHSTPAQVAPANHAQVAPENPQNGGEPSEKAENKQD
>RcLPAAT3 Lysophosphatidic acid acyltransferase 3 [*Ricinus communis*] AJP36329.1
MAIPAALAIVPIGILFFVSGFVVNLIQAVLFILVRPVSKSLHRRINKIVAELLWLELIWLVDWWAHLKIE
VYADDETFELMGKEHALLICNHRSDIDWLVGWILAQRSGCLGSALAVMKKQAKVLPIIGWSMWFSDYLFL
ERSWAKDETTLKSGFKRLEDFPMPFWLALFVEGTRFTEAKLQAAQEYAVTRGLPIPRNVLLPRTKGFVSA
VVHMRSFVPAIYDCTVDVDKSQPAPTMLRIFRRQSSVINLQIKRHSMQEVPETADGISQWCKDVFVTKDA
MLEKYHTKGTFSDQKHQHIGRPKKSLFVVIFWSNLLIFGIVMSFQFLSFLNSWQVLSFSVTLLVLVTIVM
QILIHSSESEHSTPPPKLTFSDQAKERLLQK
>RcLPAAT4 Lysophosphatidic acid acyltransferase 4 [*Ricinus communis*] AJP36330.1
MEVCKPPNHTYRQKNRPLTLFRLLRGLICLMVFVSTAFMFLVYFAPLAFLTRFASVHFRRRASSFIFSIW
LSMWPFLFEKINGTKVVFSGDDIPAEERVLIIANHRTEVDWMYLWDLALRKGFLGSIKYILKSSLMKLPV
FGWGFHILEFISVDRKWEVDEPVMRIMLSTFKGPQDPLWLALFPEGTDFTEKKCLGSQKFAAEVGLPVLK
NVLLPKTRGFCVCLEVLRGSLDAVYDVSIAYKHQCPSFLDNVFGLDPAEVHIHIRRIPVNDIPVSDSEAA
TWLMNTFQIKDELLSGFKTRGHFPNEGTEGELSTLRCLVNITIVISLTAIFTYLTLFSSVWFKIYVSLAC
VFLSLVTYFNFLPLPVVDSFNSMFSYKKI
>RcLPAAT5 Lysophosphatidic acid acyltransferase [*Ricinus communis*] EEF43306.1
MEVRGAVFSPDGTGGRALTPLRMIRGIMCLLMLLVSAFMMLVYFGFASSVIMRLFSIRYSRKATSFFFGA
WLALWPSLFEKINKTKVVFSGETVPAKECVLLIANHRTEVDWMYLWDLAWRKGCLGFIKYVLKSSLMKLP
IFGWGFHILEFIPVERKWEVDGSNMRRVLSSFKDPKDPLWLALFPEGTDFTDQKCERSKKYAAENGLPIL
NNVLLPKTKGFYACLECLRGSMDAVYDVTIGYKPRCPSLLDNVFGVNPSEVHIHVRRIALDGIPKSEEEV
AAWLMNTFQLKDQLLSNFYSQGHFPHQGTEGGLSTLKCFVNFLALILLISILCCYLASATHFDVRPMPLL
SFLSCQ
>GmLPAAT2 1-acyl-sn-glycerol-3-phosphate acyltransferase 2 [*Glycine max*] XP_003535857.1
MTAVVVVPLGLLFFASGLIVNLIQAICYVVVRPVSKNLYRRMNRVVAELLWLELVWIIDWWAGVKVQVFT
DPETFRSMGKEHALVISNHRSDIDWLVGWVLAQRSGCLGSTLAVMKKSSKFLPVIGWSMWFSEYLFLERS
WAKDERTLKSGLQQLRDFPLPFWLALFVEGTRFTQAKLLAAQEYAASAGLPVPRNVLIPRTKGFVSAVNH
MRSFVPAIYDVTVAIPKSSPAPTMLRLFRGKSSVVHVHIKRHAMKDLPEEDEAVAQWCRDMFVAKDTLLD
KHIAEDTFSDQELQDTGRPIKSLVVVISWACVVVMGVVKFLQWSSLLSSWKGVAFSAFGLGVVTLLMHIL
IMFSQSERSTPTKVAPAKSKNSEQLEARDNKQD
>CsLPAAT3 PREDICTED: 1-acyl-sn-glycerol-3-phosphate acyltransferase 3 [*Camelina sativa*] XP_010479592.1
MTIPVALVFIPVGVLFLISGLIVNIIQLVFFITVRPFSRSLYRRINKTVAELLWLQLIWLFDWWACIKIN
LYADAETLELIGKEHALVLSNHRSDIDWLIGWVMAQRAGCLGSSLAIMKKEAKYLPIIGWSMWFSDYIFL
ERSWAKDENNLKAGFKRLEDFPMTFWLALFVEGTRFTQEKLEAAQEYASIRSIPSPRNVLIPRTKGFVSA
VSHIRSFVPAIYDCTVTVQNIQPTPTLLRMFSGQSSEVNLQMRRHKMSDLPETDDGIAQWCQDLFITKDA
QLEKYFTKDVFSDLDVHQINRPIKPLIVVIIWLCLLIYGGFKLFQWLSVVASWKIIFLLVFFLVIATITM
QVLIQSSESQRSTPAKRPLQEQLITA
>HnLPAAT3 1-acyl-sn-glycerol-3-phosphate acyltransferase 3 [*Helianthus annuus*] XP_021973179.1
MALAAVVVLPIGLLFLLSGLVINFLQALFFVIVRPCSKNVYRKMNAHLAQLLWLEVVWLFDWWAHIKVDL

YIDSETLELMGKEHALLICNHKSDIDWLIGWVLAQRAGCLGSTLSLIKKSLKLLPVIGWSMWFTEYIFLE
RSWAKDEHTLKAGFENLTDFPMPFWLAVFVEGTRFTQAKLIAAQEYAIAMKLPIPRNLLIPRTKGFVAAV
NHLRSFVPAIYNCTVAIPKDEPLPTILRMFRGRSSTVHIHIKRHLICELPETDSGIKQWCRDRFVEKDAS
LELHKSKNSFDSSECHSIGRPKKSLFVVIFWSWLLLYGAIKFFEWCSFSWGTIAFCALLLLFVVILMQIL
ILFSQSEKSDSLRSQTINYKEDTLNQKLIP

>HnLPAAT4 probable 1-acyl-sn-glycerol-3-phosphate acyltransferase 4 [*Helianthus annuus*] XP_021983651.1
MDDCQALNSDTNSELKHYALTPLRLLRGLACLVVYLSTAFMFLVYFSPVAAVFLRLFSVHYSRKACSFLF
GLWLALWPFLFEKINKTKVVFSGETVPEKERVLLIANHRTEVDWMYLWDLALRKGCLGCIKYVLKSSLMK
LPIFGWGFHIFEFISVERKWEVDEPIMRKMLSTFTNPLDPLWLAVFPEGTDFTEQKCIKSQKYAAENGLP
ILKNVLLPKTRGFHACVEILRGSLDAVYDVTIAYKNRCPTFMDNVFGVEPSEVHVHVRRISLEEIPVSED
ECNTWLLNTFHQKDQLLSDFIAQGYFPNEGTEGDLSTFKCLVNCTIVLVITSVFAYLAVFSSVWFKVYVA
LSCAYLACATAYNFWPSPILDYVKGLLSGKKSS

>AtLPAAT1 Phospholipid/glycerol acyltransferase family protein [*Arabidopsis thaliana*] NP_194787.2
MDVASARSISSHPSYYGKPICSSQSSLIRISRDKVCCFGRISNGMTSFTTSLHAVPSEKFMGETRRTGIQ
WSNRSLRHDPYRFLDKKSPRSSQLARDITVRADLSGAATPDSSFPEPEIKLSSRLRGIFFCVVAGISATF
LIVLMIIGHPFVLLFDPYRRKFHHFIAKLWASISIYPFYKINIEGLENLPSSDTPAVYVSNHQSFLDIYT
LLSLGKSFKFISKTGIFVIPIIGWAMSMMGVVPLKRMDPRSQVDCLKRCMELLKKGASVFFFPEGTRSKD
GRLGSFKKGAFTVAAKTGVAVVPITLMGTGKIMPTGSEGILNHGNVRVIIHKPIHGSKADVLCNEARSKI
AESMDL

>AtLPAAT2 Lysophosphatidyl acyltransferase 2 [*Arabidopsis thaliana*] ABG48392.1
MVIAAAVIVPLGLLFFISGLAVNLFQAVCYVLIRPLSKNTYRKINRVVAETLWLELVWIVDWWAGVKIQV
FADNETFNRMGKEHALVVCNHRSDIDWLVGWILAQRSGCLGSALAVMKKSSKFLPVIGWSMWFSEYLFLE
RNWAKDESTLKSGLQRLSDFPRPFWLALFVEGTRFTEAKLKAAQEYAASSELPIPRNVLIPRTKGFVSAV
SNMRSFVPAIYDMTVTIPKTSPPPTMLRLFKGQPSVVHVHIKCHSMKDLPESDDAIAQWCRDQFVAKDAL
LDKHIAADTFPGQQEQNIGRPIKSLAVVLSWACVLTLGAIKFLHWAQLFSSWKGITISALGLGIITLCMQ
ILIRSSQSERSTPAKVVPAKPKDNHHPESSSQTETEKEK

>AtLPAAT3 Lysophosphatidyl acyltransferase 3 [*Arabidopsis thaliana*] AT1G51260
MKIPAALVFIPVGVLFLISGLIVNIIQLVFFIIVRPFSRSLYRRINKNVAELLWLQLIWLFDWWACIKIN
LYVDAETLELIGKEHALVLSNHRSDIDWLIGWVMAQRVGCLGSSLAIMKKEAKYLPIIGWSMWFSDYIFL
ERSWAKDENTLKAGFKRLEDFPMTFWLALFVEGTRFTQEKLEAAQEYASIRSLPSPRNVLIPRTKGFVSA
VSEIRSFVPAIYDCTLTVHNNQPTPTLLRMFSGQSSEINLQMRRHKMSELPETDDGIAQWCQDLFITKDA
QLEKYFTKDVFSDLEVHQINRPIKPLIVVIIWLGFLVFGGFKLLQWLSIVASWKIILLFVFFLVIATITM
QILIQSSESQRSTPAKRPLQEQLISA

>AtLPAAT4 Lysophosphatidyl acyltransferase 2 [*Arabidopsis thaliana*] AT1G75020
MEVCGDLKSDNLKNRPLTPLRILRGLMILLVFLSTAFMFLLYFAPIAALGLRLLSVQQSRKVVSLIFGLW
LALWPYLFETVNGTTVVFSGDIIPVEKRVLLIANHRTEVDWMYLWNIALRKGCLGYIKYVLKSSLMKLPI
FGWGFHVLEFIPVERKREVDEPVLLQMLSSFKDPQEPLWLALFPEGTDFTEEKCKRSQKFAAEVGLPALS
NVLLPKTRGFGVCLEVLHNSLDAVYDLTIAYKPRCPSFMDNVFGTDPSEVHIHVRRVLLKEIPANEAESS
AWLMDSFKLKDKLLSDFNAQGKFPNQRPEEELSVLKCIATFAGVISLTVVFIYLTLYSHSCFKVYACLSG
TYLTFATYYKFQPSPGCFREDSCKVKNH

>AtLPAAT5 Lysophosphatidyl acyltransferase 5 [*Arabidopsis thaliana*] [*Arabidopsis thaliana*] At3g18850
MEKKSVPNSDKLSLIRVLRGIICLMVLVSTAFMMLIFWGFLSAVVLRLFSIRYSRKCVSFFFGSWLALWP
FLFEKINKTKVIFSGDKVPCEDRVLLIANHRTEVDWMYFWDLALRKGQIGNIKYVLKSSLMKLPLFGWAF
HLFEFIPVERRWEVDEANLRQIVSSFKDPRDALWLALFPEGTDYTEAKCQRSKKFAAENGLPILNNVLLP
RTKGFVSCLQELSCSLDAVYDVTIGYKTRCPSFLDNVYGIEPSEVHIHIRRINLTQIPNQEKDINAWLMN
TFQLKDQLLNDFYSNGHFPNEGTEKEFNTKKYLINCLAVIAFTTICTHLTFFSSMIWFRIYVSLACVYLT
SATHFNLRSVPLVETAKNSLKLVNK

>BnPAH1-1 Phosphatidate phosphatase 1 [*Brassica napus*] XP_013750374.1
MSLVGRFGSLISQGVYSVATPFHPFGGAIDAIVVQQEDGSFRSTPWYVRFGKFQGVLKGAEKFVKISVNG
TEADFHMYLDNSGEAYFIREVDPAAANDDTENNNGNENNGLRLEHSLSDAGAEREGFNSLSRLDRTESDC
NRRYYDFQDDDDDDPPSPTSEYGEGQGSDSEVVLVSVDGRILTAPVSAAEQEAENLRLNTPQFHLAPGDG
TEFCEGNTEFASSETSWDTEFISKVESSEKVDSRSRDITVKDSHYTELSQPGENVKSEEPAQNLKEGELL
STAVTENARGEEEVAVETVNTLIDGSESSTTQLTTEEVRITEESVDANTESECEDEQTNTSAETAILIES
EASERVSIDSTREEDEQLTPSKPNKDSENRNTIDSVAATSSVDIELKYELSLCKDELRQGMGLTAAAEVF
NTHIISIEEYKNSAASILESENLVVRIRETYMPWKKAARIILGKVAFDLDLDIQPEDVISVEENESSKPK
DDDEAAATTPSSSGRRWRLWPIPFRRVKTIEHTSSNSSSEEDLFVDSEPGLQNSPEVLSAAESRHESPRR
QLVRTNVPTNEQIASLNLKDGQNMITFSFSTRVLGTQQVDAHIYRWRWDTKIVISDVDGTITKSDVLGQF
MPLVGKDWTQSGVAKLFSAIKENGYQLLFLSARAIVQAYLTRSFLNNLKQDGKALPNGPVVISPDGLFPA
LYREVIRRAPHEFKIACLEDIRKLFPKDYNPFYAGFGNRDTDELSYRKLGIPKGKIFIINPKGEVATGHR
IDVKKSYTSLHTLVNDMFPPTSLVEQEDYNPWNFWKLPVEEVD
>BnPAH2 Phosphatidate phosphatase 2 [*Brassica napus*] XP_013644973.1
MNAVGRIGSYIYRGVGTVSGPFHPFGGAIDIIVVEQPDGTFKSSPWYVRFGKFQGVLKNRRNLIKIEVNG
VDSGFTMYLAHTGQAYFLRQVEDVLGEVYTLSSGDEAETSRRKSSSSSSDGVDKKIKIPLKSKSCNYDGV
VGGKPGILGFVFGGRSVKVDDGVTSMERAEIAADLLDVKWSTNIDTRKCVKGKASKSFDSILETPLVASP
TLQYLDEKEQDCFRESSNNVVEDDDDYSLLLVENGSSTVFSVTTSQGSGNVEPPPLAQDSVNLDSKQQVL
GVPEYQADKTSLQEGSSTGSLVQDDFEKTQPTNGATVQQPEVEEEQFSFSDIGDCKSAETSSQESSFLHT
VKVEIYDENEISPENGVEVLSEPIEIERKKDISGEEMERLAESLPIMRLHNNNDIDAGPCRQPMSQSFDP
SSNTLKPSSRGSPSLKAFEHVITNPEVVELSLCKHLLREGMGAEAASQAFNSEKLDMEKFASLGPSVLEN
DMLIVKIGGCYFPWDAAAPIILGAVSFGTSQVFEPKGMIVVDRNEKPDDAVLAPSGGSWNLWPFSQRRSR
NDSEASSKDAAEREEKKQEKSSPRPVKKTVRALTPTSEQLASLNLKEGMNTVNFTFSTNIVGTQQVDARI
YLWKWDARIVVSDVDGTITRSDVLGQFMPLVGIDWSQTGVTHLFSAVKENGYQLMFLSARAISQASVTRQ
FLVNLKQDGKALPDGPVVISPDGLFPSLFREVIRRAPHEFKISCLEEIRALFPPEHNPFYAGFGNRDTDE
ISYLKVGIPRGKIFTINPKGEVAVNRRVDTRSYTNLHALVNGMFPATTTSSEPEDFNTWNFWKLPPPPLM
>BnPAH1-2 Phosphatidate phosphatase 1 [*Brassica napus*] CDY41298.1
MSLVGRVGSLISQGVYSVATPFHPFGGAIDVIVVQQEDGSFRSTPWYVRFGKFQGVLKGAEKFVKISVNG
DEADFHMYLDNSGEAYFIREVDPETTNDLISSSEDSNGSERVLTLEHSSSDVGAGELREGLSSLNRLDRT
ESDSNGRYYDFQDDPPSPTSEYGSARFDNLNVESYGDSSPGLDSEVVLVSVDGHILTAPVSAEEQEAENL
RLNTPQFHLAPGDGTEFCEGSTEFASSETSWDTEFISKVESSNAVVNIKSKKVDIGCVECNDENAKSEEA
ASTLEVQNLKEGELVPTTIKESVRGEDEVAIVGSCLEKSEPASIFEIQNLKEGELVPIETVTTLVDGSES
STTQLTIEEVRTSEESNTDPSAETAILIENKEGEIIESEERVSVDSTREEDEQLTPSKEPEEDNNTT
ESVAETSNIARSETELKYELSLCKDELRQGMGLTAAAEVFNTHLISTEEYKNSATSILESENLVVRIRET
YMPWKKAARIVLGKVVFDLDLEIQPEDVISVEETKPKDDETAAVTTTPSSSGRRWRLWPIPFRRVKTVEH
TTSSNSSSEEDLFVDSEPGLQNSPEVLSTNESPRRQLVRTNVPTNEQIASLNLKDGQNMITFSFSTRVLG
TQQVDAHIYRWRWDTKIVISDVDGTITKSDVLGQFMPLVGRDWTQSGVAKLFSAIKENGYQLLFLSARAI
VQAYLTRSFLNNLKQDGKALPNGPVVISPDGLFPALYREVIRRAPHEFKIACLEDIKKLFPEHYNPFYAG
FGNRDTDELSYSKLGIPKGKIFIINPKGEVATGHRVDVKKSYTSLHTLVNDMFPPTSLVEQEDYNPWNFW
KLPIEEVD
>AhPAH1-1 Phosphatidate phosphatase 1 [*Arachis hypogaea*] XP_025605008.1
MNVVGKVGSLITQGVYSVATPFHPFGGAVDVIVVQQQDGTFRSTPWYVRFGKFQGVLKGAEKFVRINVNG
VEANFHMYLDNSGEAYFIKEVDDDKDVESSVADEDAGQSDFTHVHRLDHSISDSGVLRLKANDRPQIQRV
ESDLDRRYYELQDDEPGVDGSFELSEYGSNRYDNPNGENGLDSHGSHPEVVLVSVDGHMMTAPICRSEQN
DEENVQLKNPQFHLGPAEGTEFCEGNEFTSDENAWAADYIRKLDDSTDDVPSCSYNRNGGDTTSEIVAKV
HQREEVHVRYAEATLVIKDQENYVQSDTEEVASGLRRETVFKSCLALDQFGQQDENADSEDEGSPAKVQK
SADEYNANCSVGNENAQENIKSPCSIDELSPLSRPTYSDENGSTKVELGGVQEVDDKASVHVDTDSGSHS
VTNDNETEGNDELIENSVLTDGVDDSKQIPALDDANNLSEEIEPQPTTSNDEDQSYYGLGFEISLCGHEL
KVGMGSVAAAETFDAHRISAEEFRASAPLIIKNENLVVKFRERYLLWEKAAPLILGMAVFGLDSPVNPDD
TIPVEQGDALSRDGDAGSASSGRRWRLWPIPFRRVKTLEHSTSNASSDEIFVDTESELQSSLLDPSPKSY
NPGSPHKQFVRTNVPGNEQIASLNLKEGQNLVTFSFSTRVLGTQQVDAHIYLWKWDAKIVISDVDGTITK
SDVLGQFMPLVGKDWSQSGVARLFSAIKENGYQLLFLSARAIVQAYLTRNFLLNLKQDGNTLPNGPVIS
PDGLFPSLYREVIRRAPHEFKIACLEDIKRLFPADYNPFYAGFGNRDTDELSYRKIGIPKGKIFIINPKG
EVAISQRIDSKSYTSLHTLVNDMFPPTSLVEHEDFNSWNYWRVPIPDVNDN
>AhPAH2-1 Phosphatidate phosphatase 2 [*Arachis hypogaea*] QHN88458.1
MQAVERLGSFITRGVSTVSMPFIPYGGAVDIVVVQQKDGSFKSSPWFVRFGKLHRVLKADEKKVSISVNG
SEAGFHMHMNHKGEAFFLRDTHCEQQGDEDSGSSESSSSGEDADVVLPWGRKRNFKSKSCKFDPDGSFVA

DMNALNNDKIVDRTNSRGSRLFRLVLGQRSFNGEVDEDAEDLLERAEIAANLLDLKWSTNLKFDQLPRRE
RKNTRGGTLENGLHSSKVDDLHAQQRDCSHSVLDMFDVADFEKLPKFQKSRTISVYRPHRKANKVRADTP
TSEQLASLNLQEGRNLVTFRYSTAMLGTQKIDARIYLWKWNTRIVISDVDGTITRSDVLGQVMPLVGIDW
SQTGVAHLFSAIQDNGYQLLFLSARAISQACHTRRFLFNLKQDGKVLPDGPVVISPDGLFPALYREVIRR
APHEFKIACLEEIRALFPPDCNPFYAGFGNRDTDELSYLKVGIPKGKIFIINPRGEIAVNCFDAQSYPSL
HAVVDGIFPPTDSSEQVYL
>AhPAH2-2 Phosphatidate phosphatase 2 [*Arachis hypogaea*] QHO29627.1
MRRIGSYITQGVYTVSGPFHPFGGAVDIVVVEQPDGTFKSSPWYVRFGKFQGVLKAREKIVDINVNGVDA
DFHMHLDHKGEAYFFREVDAEYGDAVIYPSSGDEYDDNRSRNLNDMQIQERLRSKSCNYDSENSNGNAGA
RSRGSLLGFVFGRKSIEEGEECGDKYGVEQMAPAEIAADLLELKWSTNIKSDRRPPRFVDKRKIAKSSSD
GDVLQEALPPLEVKEEEAASFSNSNSEHGHDKTMIKIDVAHEVECDSNGKQGGLAHECADFPVELVEVEA
EGGFERKLSEGELAPVSAFASPTGVASVDDNISADEVAQSVVFFETSEKATVDCANESSATSHDVSSTFS
PPKELLGVQATTKSPAAGLVEEENFLFSDLDESKINDQLDRPVSPESVDKEEEEEEEHHSCDDGDVKDDN
LVSEKGDLHSSSPMAIPRTEAAGEDIGHTGSLPNITTRSVSLGQPEASYPLSQSLDTRSKSLPWLFPENE
DSSEANGNQLSNAKSGVGSPPSGWRLWPFSRSVSQTYVPPMPSDTKDTTFEQSSENTISTDPNKNELKTD
PKKKHVRVKVPTSEQIASLHLKEGGNTVMFTFSTTMLGRQQVDCRIYLWKWNTRIVISDVDGTITKSDVL
GQFMPLVGIDWSQTGVAHLFSAIKENGYQLLFLSARSISQAYLTRQFLFNLKQDGKALPDGPVVISPDGL
FPSLYREGQNLFFLLLFLITSL
>GmPAH1 Phosphatidate phosphatase 1 [*Glycine max*] XP_003541397.1
MNVVGKVGSLITQGVYSVATPFHPFGGAVDVIVVQQQDGTFRSTPWYVRFGKFQGVLKGAEKFVRINVNG
VEANFHMYLDNSGEAYFLKEVDDDKGVDSIEAVQDSIDKKNGYLINVHRLDHSISDSGVLRLKDESDSLV
VSQLQRAESDIDRRFYEFPDDRSSLEDSVELSEYESNSYESLEGDNFGESQGSHPEMVLVSVDGHILTAP
ISESEQAEENVQLKTPQFHLGPGEETDLCEGNGEFSTGENAWAAGYINQLGAQTTNVQPRLCDTNGDDNT
SKLLLKLCQGEEAHICEAQDTLEIKNQDHIKTDSKGAASGIKRENVFKSYLELQDFGQQAGNADLQDIGS
SLEIQNSAEESNASCPVVDENEQESIAISKNGDELSPHSGSTSSNGHRSLKSELEIQEVEKNASGKIETA
SGSHSVTTDTEQNDEHVDKSVSNDELDESQQTPALKDVRATSEVVEPQTETSNKGDQSHLGLGFEMSLCG
HELKVGMGSIAAAEVFEAHRISVVDFTSSAPSIIKNQNLVIKFKERYMTWEKAAPLVLGMAVYGLDLPVE
SKDTIPVEQDHALKSRDDDLGSSSSGRRWRLWPIPFRKVKTFEHTNSNSSNEEVFLDSESGSLIEPTPTS
STQGSSHKQFLRTNVPTTEQIASLNLKEGQNLVTFSFSTRVLGTQQVDAHIYLWKWNARIVISDVDGTIT
KSDVLGQFMPLVGKDWTQSGVARLFCAIKENGYQLLFLSARAIVQAYLTRNFLLNLKQDGKTLPNGPVVI
SPDGLFPSLYREVIRRAPHEFKIACLEDIKRLFPSDYNPFYAGFGNRDTDELSYRKIGVPKGKIFIINPK
GEVAISHRIGAKSYTSLHTLVNDMFPPTSLVEQEDYNSWNYWKTPLPDID
>GmPAH2 Phosphatidate phosphatase 2 [*Glycine max*] XP_006578018.1
MQAVGRIISQGVYTFSGPFHPFGGAVDIVVVEQQDGTFKSSPWYVRFGKFQGVLKAREKVVDICVNGVQA
GFQMHLDHKGEAFFLREIDAQEEEAILMFPSSSGDDADDQTRSHSLRSESLNYDAAAEVVGRTTSSRRSR
ILGLVFGRRSLKREDGAGVGDGDGVGDGIGNRVGSLERAEIAANLLDINWSTNPSAEQGQDVIANPSGDG
EVVVENGELNEEACFGGECDLNWKEIVYDIAESDVQVACVEVEVEACVEKELNGEEVSIDAPGENSNESS
SETSKLGVDCSSEQAHEVMYLAGPECEEVHVHVHDEVLHGATVLLAEDTEAEEVIENADLESHSRIQQTD
SSDSDDIRYNEVVVEEQPTSPKSQTVKMGLGHYSNEKVEPNCIIKPSSYSILADQALDDNNMKDKDVSST
LSSPLDSVDDSLPRKASRRSSSPSSEDENFLFSGHDKSVINDRFERSFSPEHVDKEDHVSYGNDSEKLTA
ISNPIDIPMNKAAGEEVVQFSGSLPNISSGSDIMVEHNVRYPLCQSLDSKSTSLPWAFPGKDDLECLKSD
EDKGNQLSHEGQGAKDYNDSGELKGDPSTRNPSAGGSWRIWPFSLRREGSRKSMLPPSPSDSKNTTFVNS
PENTISTDMNKNELKPNLMKKKVKEMTPTSEQLASLNLKDGMNTVTFTFSTAVLGKQQVDCRIYLWKWNA
RIVISDVDGTITRSDVLGQFMPLVGIDWSQTGVAHLFSAIKENGYQLLFLSARSISQAYITRQFLLNLKQ
DGKVLPDGPVVISPDGLFPSLYREVIRRVPHEFKIACLEDIKALFPSDSSPFYAGFGNRDTDEISYLKVG
IPLGKIFIINPRGEVVINRRVDAKSYTSLHALVNGMFPPTSSSEQEDFNSWNFWKLPPPAIDI
>AtPAH1 Phosphatidic acid phosphohydrolase 1 [*Arabidopsis thaliana*]
AT3G09560
MSLVGRVGSLISQGVYSVATPFHPFGGAIDVIVVQQQDGSFRSTPWYVRFGKFQGVLKGAEKFVRISVNG
TEADFHMYLDNSGEAYFIREVDPAANDTNNLISGSENNNGNQNNGVTYRLEHSLSDSGTGELREGFDPLS
RLERTESDCNRRFYDFQDDPPSPTSEYGSARFDNLNVESYGDSQGSDSEVVLVSIDGHILTAPVSVAEQE
AENLRLNTPQFHLAPGDGTEFCEGNTEFASSETPWDTEYIDKVEESSDTANIASDKVDAINDERNDLDSH
SRDNAEKDSHDAERDLLGSCLEQSELTKTSENVKSEEPGPTFEDRNLKEGEFPLRTITENDRSEDEVTIE
SIDTLVDSFESSTTQITIEEVKTTEGSRISVDSNADSECKDEQTSAETAILINNQESSISVDSNPDSECK
DEQPRISAETAILINNQEGGIIESEDQDSERVSIDSTREEVEQLTPSKPTDKDNEDRKTVVSVGVTSSVD
EGEPDTDQRYELSLCKDELRQGMGLSAAAEVFDAHMISKEEYINSATSILESENLVVRIRETYMPWTKAA
RIVLGKAVFDLDLDIQPDDVISVEENESPKPKDDETTITPSSSGTRWKLWPIPFRRVKTVEHTGSNSSSE

EDLFVDSEPGLQNSPETQSTTESRHESPRRQLVRTNVPTNEQIASLNLKDGQNMITFSFSTRVLGTQQVD
AHIYRWRWDTKIVISDVDGTITKSDVLGQFMPFIGKDWTQSGVAKLFSAIKENGYQLLFLSARAIVQAYL
TRNFLNNLKQDGKALPTGPVVISPDGLFPALYREVIRRAPHEFKIACLEDIRKLFPTDYNPFYAGFGNRD
TDELSYRKLGIPKGKIFIINPKGEVATGHRIDVKKSYTSLHTLVNDMFPPTSLVEQEDYNPWNFWKLPIE
EVE
>AtPAH2 Phosphatidic acid phosphohydrolase 2 [*Arabidopsis thaliana*] AT5G42870
MNAVGRIGSYIYRGVGTVSGPFHPFGGAIDIIVVEQPDGTFKSSPWYVRFGKFQGVLKNGRNLIRIDVNG
VDSGFNMYLAHTGQAYFLREVEDVVGESESGEVYTLSSGDEAETTSRDDVVDKVKIPLKSRSCNYDSPSP
RTGNGKIVGKPGILGYVFGGRSVRESQDCGVERAEIAADLLEVKWSTNIDTRKRGKGMSSESLDGKDYGE
STSTSGKSCVEGSSEMIVDSDSILETPLVASPTLRFLDEKEQDFRESTNVEDYCEEDGSSGVVVENGLCE
ASNMVFSVTSEGSGNVEIFVEPRTEALAEDAVSGSDLDSKQELLRAPESVEIATLGSADQADMGSIGTSQ
EGSSTGSPVQDENKITIKDMHISAGDFEKSQSASGESILQPEIEEEQFSFSDLDECKPGGNSSVGSSSSD
TVKVDGKESYDETKTSPENGVDNTMALSEPINIERKKDIFTDEMERLVGSLPIMRLQNIDDMDASPSQPL
SQSFDPCFNTSKLDLREDESSSGGLDAESVAESSPKLKAFKHVIANPEVVELSLCKHLLSEGMGAEAASQ
AFNSEKLDMEKFASLGPSILENDKLVVKIGGCYFPWDAAAPIILGVVSFGTAQVFEPKGMIAVDRNEKPG
DVLAQGSGSWKLWPFSLRRSTKEAEASPSGDTAEPEEKQEKSSPRPMKKTVRALTPTSEQLASLDLKDGM
NSVTFTFSTNIVGTQQVDARIYLWKWNSRIVVSDVDGTITRSDVLGQFMPLVGIDWSQTGVTHLFSAVKE
NGYQLIFLSARAISQASVTRQFLVNLKQDGKALPDGPVVISPDGLFPSLFREVIRRAPHEFKIACLEEIR
GLFPPEHNPFYAGFGNRDTDEISYLKVGIPRGKIFIINPKGEVAVNRRIDTRSYTNLHTLVNRMFPATSS
SEPEDFNTWNFWKLPPPSLM

>AhDGAT1C Diacylglycerol O-acyltransferase 1C [*Arachis hypogaea*] XP_025688510.1
MAISDVHETSVAGDGANHSSLRQRHSRVASSGGNMFDEAEASAEAVMIASSGSDDSLNEKIGAAREEKVK
ERQKQKEEDRKPPDHASRNEVQDGERAAAGDNFTYRASVPVHRRIKDSPLSSRNIFKQSHAGLFNLCVVV
LIAVNSRLIIENIMKYGWLINSGFWFSSKSLRDWPLLMCCISLNLFPLAAFMVEKLAQKKRISEPVIFLL
HTIIMTGEILFPVLVILSCDSTFLSGLTLMMVACIIWLKLVSYAHTSHDLRSLSLSIEKGETLPNNLNME
HPYRVSFRSMAYFMVAPTLCYQPSYPRTPSVRKGWVFRQLIKLVIFTGLMGFIIEQYMHPIVQNSQHPFK
GNLLYGFERTLKLSVPNVYVWLCMFYCFFHLWLNILAELVQFGDREFYKDWWNAKTVDEYWRNWNMPVHK
WMVRHIYFPCIRHGMSKLCIAVPCHKFKLWAFIGIMFQVPLSIVTNFLHEKCKSSMVGNMVFWFTFCILG
QPMCVLLYYHDWMNRHREHN
>AhDGAT1-3 Diacylglycerol acyltransferase 1 [*Arachis hypogaea*] ATG83402.1
MAIYQSVGATATVQGDSGANSSTVRRRRSGGATSATPLEKVELDAFDGESSSEELAKDSSSDDNNNGNGV
DDASKGNRQQNAADFSAVNFDYRPSFPAHRRIRDSPLSSGNIFKQSHAGLFNLCIVVLVAVNSRLIIENL
MKYGWLIKTDFWFSSTSLSDWPLFMCCLTLLLFPVASFIVEKLAQHKYIPEPVVVILHIIITSTSLLYPI
FVILRSDSAFLSGVTLMLFACIVWLKLVSYGHTNYDLRALAISNEKVEGLPSTLSMDYPYDVSIRRLAYF
MLAPTLCYQPSYPRTPSIRKGWVFRQLVTLIIFTGLMGFIIEQYINPIVQNSQHPLKGNLLYAIERVLKL
SVPNLYVWLCMFYCFFHLWLNILAELLRFGDREFYKDWWNAKTVDEYWRLWNMPVHKWMIRHLYFPCIRH
GVPKGVALLIAFLVSALFHELCIAVPCHIFKLWAFIGIMMQVPLVLITNYLQNKFRSSMVGNMIFWFIFC
ILGQPMCVLLYYHDLMSRKVKTE
>GmDGAT1A Diacylglycerol O-acyltransferase 1A [*Glycine max*] NP_001237289.1
MAISDEPETVATALNHSSLRRRPTAAGLFNSPETTTDSSGDDLAKDSGSDDSISSDAANSQPQQKQDTDF
SVLKFAYRPSVPAHRKVKESPLSSDTIFRQSHAGLFNLCIVVLVAVNSRLIIENLMKYGWLIKSGFWFSS
KSLRDWPLFMCCLSLVVFPFAAFIVEKLAQQKCIPEPVVVVLHIIITSASLFYPVLVILRCDSAFLSGVT
LMLFACVVWLKLVSYAHTNYDMRALTKSVEKGEALPDTLNMDYPYNVSFKSLAYFLVAPTLCYQPSYPRT
PYIRKGWLFRQLVKLIIFTGVMGFIIEQYINPIVQNSQHPLKGNLLYAIERVLKLSVPNLYVWLCMFYCF
FHLWLNILAELLRFGDREFYQDWWNAKTVEDYWRMWNMPVHKWMIRHLYFPCLRHGIPKAVALLIAFLVS
ALFHELCIAVPCHIFKLWAFGGIMFQVPLVFITNYLQNKFRNSMVGNMIFWFIFSILGQPMCVLLYYHDL
MNRKGKLD
>OeDGAT1 Acyl-CoA:diacylglycerol acyltransferase 1 [*Olea europaea*] AAS01606.1
MTIPELPESLETTTLNSHHSRAASTVRRRSIDVAVLESDSNSLEAVNDSDSDVNNTNEMGNLRGGVVESA
LEEPSELGTEGLRNGKEENEHVRTGESNQEMEVLASAKFAHRPSAPVHRRIKESPLSSDAIFKQSHAGLF

NLCIVVLVAVNSRLIIENLMKYGWLINSGFWFSSTSLKDWPLLMCCLSLPIFPLAAFFVEKLVLLKYISE
CVAVFLHILITTAAILYPVLVILRCDSAVLSGVTLMLFACIVWLKLVSYAHASHDMRALAKSLDKGETLS
GYWNSDDSYGASFQSLAYFMVAPTLCYQPSYPRTSCIRKGWVVRQLIKLIIFTGFMGFIVEQYINPIVRN
SQHPLKGNLLYAIERVLKLSVPNLYVWLCMFYCFFHLWLNILAELLCFGDREFYKDWWNAKTVEEYWRMW
NMPVHKWMVRHIYFPCLRNGMPRGGAILIAFLISAIFHELCIAVPCHIFKFWAFIGIMFQVPLVILTNYL
QDKFQNSMVGNMIFWCFFSILGQPMCLLLYYHDLMNRKASAK

>BnDGAT1 Diacylglycerol acyltransferase [*Brassica napus*] AAD45536.1
MAILDSGGVAVPPTENGVADLDRLHRRKSSSDSSNGLLSDTSPSDDVGAAAAERDRVDSAAEEEAQGTAN
LAGGDAETRESAGGDVRFTYRPSVPAHRRTRESPLSSDAIFKQSHAGLFNLCVVVLVAVNSRLIIENLMK
YGWLIRTDFWFSSTSLRDWPLFMCCLSLSVFPLAAFTVEKMVLQKFISEPVAIILHVIITMTEVLYPVYV
TLRCDSAFLSGVTLMLLTCIVWLKLVSYAHTSYDIRTLANSADKVDPEISYYVSLKSLAYFMVAPTLCYQ
PSYPRSPCIRKGWVARQLAKLVIFTGLMGFIIEQYINPIVRNSKHPLKGDLLYAIERVLKLSVPNLYVWL
CMFYCFFHLWLNILAELLCFGDREFYKDWWNAKSVGDYWRMWNMPVHKWMVRHVYFPCLRIKIPKVPAII
IAFLVSAVFHELCIAVPCRLFNLWAFMGIMFQVPLVFITNFLQERFGSMVGNMIFGSASCIFGQPMCGLL
YYHDLMNRKGSMS

>CsDGAT1B Diacylglycerol O-acyltransferase 1B [*Camelina sativa*] AQU71886.1
MAILDSGGGGVSTATATENGGGEFVDLRRRKSRSDSNGVLCGSDNPPSDDVGAPADVRDRIDSVVNDDAQ
GTTANLAGDNEIRETGGGGRGGGGEGGRGNAETTYTYRPSVPAHRRARESPLSSDAIFKQSHAGLFNLCV
VVLIAVNSRLIIENLMKYGWLIRTDFWFSSRSLRDWPLFMCCLSLSFFPLAAFTVEKLVLQKCISEPVVI
FLHIIITMTEVLYPVYVTLSCDSAFLSGVTLMLLTCIVWLKLVSYAHTNYDIRTLANSADKANPEVSYYV
SLKSLAYFMVAPTLCYQPSYPRSPCIRKGWVARQFAKLVIFTGFMGFIIEQYINPIVRNSKHPLKGDLLY
AIERVLKLSVPNLYVWLCMFYCFFHLWLNILAELLCFGDREFYRDWWNAKSVGDYWRMWNMPVHKWMVRH
IYFPCLRSKIPKTLAIIIAFLVSAVFHELCIAVPCRLFKLWAFIGIMFQVPLVFITNYLQERFGSTVGNM
IFWFIFCIFGQPMCVLLYYHDLMNRKGSMS

>AhDGAT2 Diacylglycerol acyltransferase type 2 [*Arachis hypogaea*] AEO11788.1
MEDRGNVTAAPPAEEKVFRSTEVFAAESSSKSKGFKTTLALALWLGAIHFNGALMLFALLFLPLSKALLV
FALLFVFMVIPIDEKSKFGRKLSRYICKNACSYFPITLHVEDIKAFNSNRAYVFGFEPHSVLPIGVVALA
DNTGFMPLPKIKVLASSAVFYTPFLRHIWTWLGLTPATKKNFLSLLDNGYSCILIPGGVQETFLMEHGTE
TAYLKARKGFIRIAMQKGQPLVPVFCFGQSDIYKWWKPGGKLILNFARAIKFTPIYFWGIFGSPIPFKHP
MYVVVGRPIELDKNPEPTTEEVATVHSQFVASLQDLFERYKARAGYPNLELRIV

>AhDGAT2D Diacylglycerol acyltransferase type 2D [*Arachis hypogaea*] XP_025682671.1
MEVRGNVTVAPPAEEKVFRSTEVFAPESSSKSKGFKTTLALALWLGAIHFNGALMLFALLFLPLSKALLV
FALLFVFMVIPIDEKSKFGRKLSRYICKNACSYFPITLHVEDIKAFNSNRAYVFGFEPHSVLPIGVVALA
DNTGFMPLPKIKVLASSAVFYTPFLRHIWTWLGLTPATKKNFLSLLDNGYSCILIPGGVQETFLMEHGTE
TAYLKARKGFIRIAMQKGQPLVPVFCFGQSDIYKWWKPGGKLILNFARAIKFTPIYFWGIFGSPIPFKHP
MYVVVGRPIELDKNPEPTTEEVATVHSQFVASLQDLFERYKARAGYPNLELRIV

>AhDGAT2-1 Diacylglycerol acyltransferase type 2 [*Arachis hypogaea*] AEO11791.1
MEDRGNVTAAPPAEEKVFRSTEVFAPESSSKSKGFKTTLALALWLGAIHFNGALMLFALLFLPLSKALLV
FALLFVFMVIPIDEKSKFGRKLSRYICKNACSYFPITLHVEDIKAFNSNRAYVFGFEPHSVLPIGVVALA
DNTGFMPLPKIKVLASSAVFYTPFLRHIWTWLGLTPATKKNFLSLLDNGYSCILIPGGVQETFLMEHGTE
TAYLKARKGFIRIAMQKGQPLVPVFCFGQSDIYKWWKPGGKLILNFARAIKFTPIYFWGIFGSPIPFKHP
MYVVVGRPIELDKNPEPTTEEVATVHSQFVASLQDLFERYKARAGYPNLELRIV

>OeDGAT2 Diacylglycerol acyltransferase type 2 [*Olea europaea*] ADG22608.1
MTSEGNGDMRRRQLLVSESAQQPTPAEFKGTQGSIFHTILALVLWLGSVHFNIIVVLASFVFLSFTKALM
VIGLLVILMVAPIDDRNKWGRKLARYICKHAVGYFPVNLYVEDIKAFDPNESYVFGYEPHSVWPIGVVSL
ADLTGFMPLQKIKVLASTAVFYTPFLRHIWSWLGLTPATRKNFTSLLASGYSCIIVPGGVKEALYMEHGS
EIAYLKTRRGFVRIAMEMGKPLVPVFCFGQTNVYKWWKPSGKLYLEFSRAIKFAPIFFWGVLGSPLPLRH
PMHVVVGRPIVPKRNPQPTVEEVAEVHGQFVEALQELFERHKARVGYTDMQLKIL

>GmDGAT2D Diacylglycerol O-acyltransferase 2D [*Glycine max*] BCB92186.1
MAAEPVSDGGAAAEKLISGREEFGDSSNLLSAILAMVLWLGAIHFNIALILLAVFFLPLSKSLLVFGFLF
GFMVLPINEKSRFGRRLSRFICKHACNYFPITLHVEDMKAFDPNRAYVFGYEPHSVLPIGIVALADHTGF
MPLPKVKVLASSTVFYTPFLRHLWTWLGLTPATKKNFISLLASGHSCILIPGGVQEAFHMQHGTEIAFLK
ARRGFVRVAMVKGKPLVPVFCFGQSNVYKWWKPGGKLFLKFARAIKFTPICFWGIFGSPLPFRHPMHVVV

GRPIEVDKNREPTTEEVAKIHGLFVEALQDLFERHKARAGYPNLELRIV
>EgDGAT2D Diacylglycerol O-acyltransferase 2D [*Elaeis guineensis*] XP_010932136.1
MPRVTHLGDIYPAAHRNTRRILSLSLTRSHEASWRMDHGNGDRQAAVLDDSAGAGEAAVYRGTEYSVVRT
TVALALWLGLIHFNVALVLTALLLLPARLAAAVFALLLVFMAIPLDENSKWGRKLSRWICKYAVGYFPVT
LYVEDIKVFDPNQAYVFAFEPHSVLPIGVVALADLTGFMPLTKVKVLASSAVFYTPFLRHLWTWLGLVSA
SRKNFYAYLEAGYSCIVVPGGVQEMLHMDHDSEVAFLKARKGFVRIAMETGRPIVPVFCFGQSYVYKWWK
PSGKLFVWIARAIKFTPIVFWGRFGSPIPFRHPMHVVVGKPIGLKRNPKPTIEEINEVHAQFVAAFQELF
EKHKTRAGYPDLRLRVL
>AhDGAT3-1 Diacylglycerol acyltransferase [*Arachis hypogaea*] ABW34442.1
MEVSGAVLRNVTCPSFSVHVSSRRRGGDSCVTVPVRMRKKAVVRCCCGFSDSGHVQYYGDEKKKENGTAM
LSTKKKLKMLKKRVLFDDLQGNLTWDAAMVLMKQLEQVRAEEKELKKKRKQEKKEAKLKASKMNTNPDCE
SSSSSSSSESESESSESECDNEVVDMKKNIKVGVAVAVADSPRKAETMILYTSLVARDVSANHHHHNAVE
LFSRNNDISVGSINGGLKNENTAVITTEAIPQKRIEVCMGNKCKKSGSIALLQEFERVVGAEGGAAAAVV
GCKCMGKCKSAPNVRIQNSTADKIAEGFNDSVKVPANPLCIGVAWRMLKPLWLRFLGENQESTNE
>GmDGAT3 Diacylglycerol O-acyltransferase 3 [*Glycine max*] XP_003542403.1
MEISGSVLRQLSYVSGYGTPTRSRGVASRVGLRMGTGSGFCDEGHLQYYQDTKKILTPKKLKLLKGFSKL
GLASDPEKLAMFHDLQQNLTSDAGEVLLRELEAARAKEKEMKKKRKQEIKAKLKASKMNCESSSSSSSES
SDSDGDCDQVVDMNCFRAGAGVVVPAPVEESPLPKTPIVEDTNAKAHRDAMALCSKNDISVSSVRDCIKS
ESAVVTAAPQKRIEVCMGTKCKRSGAAALMQEFERVVGVEGGAVVSCKCMGKCKTAPNVKVQNSVDHSLA
RGLDDSVNIPANPLCIGVGLGDVDAIVARFLGESHTDIGMIGAATAT
>AhDGAT3-2 Diacylglycerol acyltransferase [*Arachis hypogaea*] ABW34442.1
MEVSGAVLRNVTCPSFSVHVSSRRRGGDGCFTVPVRMRKKAVVRCCCGFSDSGHVQYYGDEKKREKGTDM
LSTKKKLKMLKKRVLFDDLQGNLTSDAAAEVLMKQLEQVRAEEKELKKKRKQEKKEAKLKASKMNTNPDC
ESSLSSSESESSESECDNEVVDMKKNIKVGVATADSPRKAETMIYTPPLLPEDVSANHHHHNAVELFSRN
NDISVGSINGGLKNENAAVITTEAIPQKRIEVCMGNKCKKSGSIALLQELERVIGAEGGAAAAVVGCKCM
GKCKSAPNVRIQNSTADKIAEGFNDSVKVPANPLCIGVALEDVETIVARFLGENQESTNE
>AhDGAT3-3 Soluble diacylglycerol acyltransferase [*Arachis hypogaea*] AAX62735.1
MEVSGAVLRNVTCPSFSVHVSSRRRGGDSCVTVPVRMRKKAVVRCCCGFSDSGHVQYYGDEKKKENGTAM
LSTKKKLKMLKKRVLFDDLQGNLTWDAAMVLMKQLEQVRAEEKELKKKRKQEKKEAKLKASKMNTNPDCE
SSSSSSSSESESESSESECDNEVVDMKKNIKVGVAVAVADSPRKAETMILYTSLVARDVSANHHHHNAVE
LFSRNNDISVGSINGGLKNENTAVITTEAIPQKRIEVCMGNKCKKSGSIALLQEFERVVGAEGGAAAAVV
GCKCMGKCKSAPNVRIQNSTADKIAEGFNDSVKVPANPLCIGVAWRMLKPLWLRFLGENQESTNE
>CsDGAT3-1 Diacylglycerol acyltransferase 3[*Camelina sativa*] XP_010479249.2
MEVSGVVLRQVPCVSGSVADGRYSGLRLVSDXGXTRTVSFRARKLRGVVCNNEFADKGHVNYYIEPTRCG
EAKEKVKVMEKEKKALKKKAKILKSLSKNLDMFSSIGFGLVPEAGLVGEIQTKTISEATEILVKQLEQLK
AEEKLLKKQRKEEKAKAKALKKMTEMDSESSSSSESSDSDCDKGKVVDMSSLRNKAKPILEPLQPEATLV
TTLPRIQEDAQSCKDTSEALQIALQTSTVFPSMANPGQTMKKVEAVPVVGLPLKRVEVCMGGKCKKLGGA
VLLDEFQKAMTGFQGSAVACKCMGKCRDGPNVRVVNETDAVMTDSVMTPSKTVCLGVGLQDVETIVTSFF
DEECSRENSVVSF
>CsDGAT3-2 Diacylglycerol acyltransferase 3[*Camelina sativa*] XP_010461643.1
MEVSGVVLRQVPCVSGSVANGRYSGLRLVSNFSGNTRTVSFRTRKLRGVVCNNEFAEKGHVNYYIEPTRC
GEAKDKVKVMEKEKKALKKKAKILKSLSKNLDMFSCIGFGLDPEAGLVGEVQTKTISEATEILVKQLEQL
KAEEKLLKKQRKEEKAKAKALKKMTEMDSESSSSSESSDSDCDKGKVVDMSSFRNKAQPILEPLQPEATL
VTTLPRIQEDAQSFKDTSEALQIALQRSTVFPSMANPGQTMKKVEAVPVVGLPLKRVEVCMGGKCKKLGG
AVLLDEFQKAMTGFEGSAVACKCMGKCRDGPNVRVVNETDAVMTDSVMTPSKTVCVGVGLQDVETIVTSF
FDEECSRENSVVSF
>CsDGAT3-3 Diacylglycerol acyltransferase 3[*Camelina sativa*] XP_010500353.1
MEVSGVVLRQVPCVSGSVADGRYSGLRLVSDISGNTRTVSFRTRKLRGVVCNNEFAEKGHVNYYIEPTRC
GEAKEKEMIKVMEKEKKALKKKAKILKSLSKNLDMFSSIGFGLDPEAGLVGEIQTKTISEATEILVKQLE
QLKAEEKLLKKQRKEEKAKAKALKKMTEMDSESSSSSESSDSDCDKGKVVDMSSFRNKAKPILEPLQPEA
TLTTLPKILEDAQSFKDTSEALQIALQTSTVFPSMANPGQTLKKVDAVSVVGLPLKRVEVCMGGKCKKMG
GAVLLDEFQKAMTGFEGSAVACKCMVKCRDGPNVRVVNETVAVMTDSVMTPSKTVCVGVGLQDVETIVTS

FFDEECSRENSVVSF
>AtDGAT1 Diacylglycerol acyltransferase 1 [*Arabidopsis thaliana*] AAF19262.1
MAILDSAGVTTVTENGGGEFVDLDRLRRRKSRSDSSNGLLLSGSDNNSPSDDVGAPADVRDRIDSVVNDD
AQGTANLAGDNNGGGDNNGGGRGGGEGRGNADATFTYRPSVPAHRRARESPLSSDAIFKQSHAGLFNLCV
VVLIAVNSRLIIENLMKYGWLIRTDFWFSSRSLRDWPLFMCCISLSIFPLAAFTVEKLVLQKYISEPVVI
FLHIIITMTEVLYPVYVTLRCDSAFLSGVTLMLLTCIVWLKLVSYAHTSYDIRSLANAADKANPEVSYYV
SLKSLAYFMVAPTLCYQPSYPRSACIRKGWVARQFAKLVIFTGFMGFIIEQYINPIVRNSKHPLKGDLLY
AIERVLKLSVPNLYVWLCMFYCFFHLWLNILAELLCFGDREFYKDWWNAKSVGDYWRMWNMPVHKWMVRH
IYFPCLRSKIPKTLAIIIAFLVSAVFHELCIAVPCRLFKLWAFLGIMFQVPLVFITNYLQERFGSTVGNM
IFWFIFCIFGQPMCVLLYYHDLMNRKGSMS
>AtDGAT2 Diacylglycerol acyltransferase 2 [*Arabidopsis thaliana*] NP_566952.1
MGGSREFRAEEHSNQFHSIIAMAIWLGAIHFNVALVLCSLIFLPPSLSLMVLGLLSLFIFIPIDHRSKYG
RKLARYICKHACNYFPVSLYVEDYEAFQPNRAYVFGYEPHSVLPIGVVALCDLTGFMPIPNIKVLASSAI
FYTPFLRHIWTWLGLTAASRKNFTSLLDSGYSCVLVPGGVQETFHMQHDAENVFLSRRRGFVRIAMEQGS
PLVPVFCFGQARVYKWWKPDCDLYLKLSRAIRFTPICFWGVFGSPLPCRQPMHVVVGKPIEVTKTLKPTD
EEIAKFHGQYVEALRDLFERHKSRVGYDLELKIL
>AtDGAT3 Diacylglycerol acyltransferase 3 [*Arabidopsis thaliana*] OAP16619.1
MEVSGVVLRQIPCVSSGSVAGLRLVSEFSGNTRTVGFRTRRFRGIVCNNEFADKGHVNYYIEPTRCGEEK
EKVKVMEKEKKALKKKAKVLKSLSKNLDMFSSIGFGLDPEAGLVGEIQTKTISEATEILVKQLEQLKAEE
KILKKQRKEEKAKAKAMKKMTEMDSESSSSSESSDSDCDKGKVVDMSSLRNKAKPVLEPLQPEATVATLP
RIQEDAISCKNTSEALQIALQTSTIFPSMANPGQTLKTVEAVSVVGLPLNRVEVCMGGKCKKSGGALLLD
EFQRAMTGFEGSAVACKCMGKCRDGPNVRVVKETDAVMTDSVRTPSKTLCVGVGLQDVETIVTSFFDEEC
SREGLGSFSY
>TmDGAT1 Putative diacylglycerol acyltransferase [*Tropaeolum majus*] AAM03340.2
MAVAESSQNTTTMSGHGDSDLNNFRRRKPSSSVIEPSSSGFTSTNGVPATGHVAENRDQDRVGAMENATG
SVNLIGNGGGVVIGNEEKQVGETDIRFTYRPSFPAHRRVRESPLSSDAIFKQSHAGLFNLCIVVLIAVNS
RLIIENLMKYGWLIDTGFWFSSRSLGDWSIFMCCLTLPIFPLAAFIVEKLVQRNHISELVAVLLHVIVST
AAVLYPVIVILTCDSVYMSGVVLMLFGCIMWLKLVSYAHTSSDIRTLAKSGYKGDAHPNSTIVSCSYDVS
LKSLAYFMVAPTLCYQPSYPRSSCIRKGWVVRQFVKLIVFIGLMGFIIEQYINPIVRNSKHPLKGDFLYA
IERVLKLSVPNLYVWLCMFYSFFHLWLNILAELLRFGDREFYKDWWNAKTVAEYWKMWNMPVHRWMVRHL
YFPCLRNGIPKEGAIIIAFLVSGAFHELCIAVPCHVFKLWAFIGIMFQVPLVLITNYLQEKFSNSMVGNM
IFWFIFCILGQPMCVLLYYHDLINLKEK
>JrDGAT3 Diacylglycerol O-acyltransferase 3 [*Juglans regia*] XP_018840223.2
MEASGFSFRRIPCLSGAKADTRSSKPSISCVSVGSRKLRVLGFQQSRARVRPRKAYGAMNMIMNSGFCDN
GHKRYYYVSPMCSGKKEKEKEKSVNSCSSLTAKKKLKLFKGLAEDLAKFSDMGFGLDAYDGLAADVQQKA
IADAVEVLMSQLQHLRAAEELKKRRKQENAKLKDRQMNIMLDCESSSSSSESSDSECEDVIDMSFLRSEP
LTQATLPVLDEVQLVSQENVTTLTLPSRLTQEGSAVLSDGLGDLKSENEVERCTGIGTSSSSNSFGHNDR
CSSVTGSSAKRIEVCMGGKCKKSGAATLLEEFGKVTGVEGAVVGCKCLGKCRDGPNVRILNSVDGSEAEG
ADVSVRSPLNPLCIGVGLDDVGVIVAKFFGDEPKDSGLAAASLQNFL
>PfDGAT1 Diacylglycerol acyltransferase [*Perilla frutescens*] AAG23696.1
MAILDSPEILDTTSSSADNGAAHHTTLRRRQSARSVPPLLDSDSNSLEAESAINDSENVRNDANLIENLR
GGAVESENEKQESYGKEEGAKVKENGETSNGNGTDVMAVKFTFRPAAPAHRKNKESPLSSDAIFKQSHAG
LFNLCIVVLVAVNSRLIIENLMKYGWLIKSGFWFSSTSLRDWPLLMCCLSLPVFALASFLVEKLVKLNYI
PEWVAVFLHVTITTVEILFPVVVILRCDSAVLSGVTLMLFACTVWLKLVSYAHTNYDLRVLAKSLDKWEA
MSRYWNLDYAYDVSFKSLAYFMVAPTLCYQPSYPRTACIRKGWVVRQLIKLVIFTGLMGFIIEQYINPIV
QNSQHPLKGNLLYAIERVLKLSVPNLYVWLCMFYCFFHLWLNILAELLCFGDREFYKDWWNARTVEEYWR
MWNMPVHKWMVRHIYCPCLQNGIPKIVAVLIAFLVSAIFHELCVAVPCQIFKFWAFSGIMLQVPLVIVTN
YLQEKFKNSMVGNMMFWCFFCIFGQPMCVLLYYHDLMNRKASAR
>SaDGAT1 O-acyltransferase [*Striga asiatica*] GER56917.1
MAILESPESLDTTSSSAENGHGRDHATTLRRRPSARSVEPLLESDSSSLDGETAVNDGERYQSDANSAAK
LCGGAIESNEEREAATGSEGFTCGKGEAEKVKEGDGKGVDALAVKFAYRPSAPAHRRIKESPLSSDAIFK
QSHAGLFNLCIVVLVAVNGRLIIENLMKYGWLIKAGFWFSSKSLRDWPLFIVSLPVFPLAAFLVEKLVKQ

KYIHEWVAVFLHVVITTTEILYPVVVILRCDSAVLSGVTLMLFACIVWLKLVSYAHTNYDLRVLAKSLDK
WEALSSYWNMDYSYDISFKSLVYFIVAPTLCYQPSYPRTSCIRKGWVVRQLIKLVIFTGFMGFIVEQYIN
PIVQNSQHPLKGNLLYAVERVLKLSVPNLYVWLCMLNILAELLRFGDREFYKDWWNARTVEEPVHKWMVR
HIYFPCLRNGIPKVVAVLIAFLVSAIFHELCIAVPCHMFKLWAFIGQLVATVKRELFCGVKLKVGNMMFW
CFFCIVGQPMCVLLYYHDLMNRKASAR

>ShDGAT2 Diacylglycerol acyltransferase 2 [*Salvia hispanica*] AXN76729.1

MSSESNGDVRRRRSPSSEAESDAPPTAAEFKGTRGSLMNSIIAIVLWLGSVHLIVSIVLASFFFLPFPKS
LGGIVLLFVFMVIPINERSRWGRNLARYICKHAVGYFPVALHVENIKAFDPNEAYVFGYEPHSVWPIGVI
AIADLTGFMPLPKIKVLASSAVFYTPFMRHLWTWLGLSAATRKNFTALLSSGYSCIIIPGGVQEACYMEH
GSEVAFLQSRKGFVRIAIETGKPLVPVFCFGQTDVYKWWRPGGKLFREFSRAIKFTPIVFWGVLGSPLPF
RQPLHVVVGEPILVKKNSQPTKEEVMEVHARFVEALQDLFQRHKARVGHPDLQLRIL

>HiDGAT2 Acyl-CoA:diacylglycerol acyltransferase [*Handroanthus impetiginosus*] PIN08318.1

MAAEANGDVKQRKSPSPEAAEPPTPAEFKGTQGPLLHTILALVLWLGSVHLNFLVVLFAFVFLPFPKALG
VIGLLLIFMVIPIDERSKWGRRLSRYICKHAVGYFPVTLYVEDIKAFDPNEAYVFGYEPHSVWPIGVVAL
ADLTGFMPLPRIKVLASTAVFYTPFLRHVWSWLGLSPATRENFSSLLTSGYSCIIVPGGVQEALYMEKGS
EVAFIQKRRGFVRIAMETGKPLVPVFSFGQSDVYRWWKPRGKLYREFSRAIKFAPIIFWGVLGSPLPLRH
PMHVVVGRPIMLKKNPQPTAEEVAEVHAQFIEALKDLFERHKERVGYGDLHLRIL
