# Supplementary material for: Longer Duration of Active Oil Biosynthesis during Seed Development Is Crucial for High Oil Yield—Lessons from Genome-Wide In Silico Mining and RNA-Seq Validation in Sesame
Source: Plants (Basel). 2022 Nov 4;11(21):2980. doi: 10.3390/plants11212980 (PMC9657858; doi:10.3390/plants11212980)
Supplement: Supplementary file 1 [file plants-11-02980-s001.zip › Table_S1.pdf]

Table S1. List of gene products used in this study for in *silico* analysis

Subfamily	Gene ID	NCBI Accession Number	Botanical name
**ACCase**	*Si*accB1	XP_011077154.1	*Sesamum indicum* (2x)
	*Si*accB2	XP_011072841.1	*Sesamum indicum* (2x)
	*Si*accC	XP_011070473.1	*Sesamum indicum* (2x)
	*Si*accA-1	XP_011077598.1	*Sesamum indicum* (2x)
	*Si*accA-2	XP_020551382.1	*Sesamum indicum* (2x)
	*Si*accA-3	XP_011094964.1	*Sesamum indicum* (2x)
	*Si*accD	YP_004935675.1	*Sesamum indicum* (2x)
	*Bn*accB1	XP_013649844.1	*Brassica napus* (4x)
	*Bn*accB2	XP_013728269.1	*Brassica napus* (4x)
	*Gh*accB	ABU41516.1	*Gossypium hirsutum* (4x)
	*Bn*accC	ADI79335.1	*Brassica napus* (4x)
	*Bn*accA	ACN65504.1	*Brassica napus* (4x)
	*Bn*accA2	ACT83681.1	*Brassica napus* (4x)
	*Bn*accD	CAA90747.1	*Brassica napus* (4x)
	*Gma*accB2	AAG44776.1	*Glycine max* (2x)
	*Gma*accB1	NP_001238375.1	*Glycine max* (2x)
	*Gm*accA-1	AAF80496.1	*Glycine max* (2x)
	*Gm*accA-3	AAF89549.1	*Glycine max* (2x)
	*Gm*accD	AAA80643.1	*Glycine max* (2x)
	*Ah*accB1	XP_025649626.1	*Arachis hypogaea* (4x)
	*Ah*accB2	XP_025681786.1	*Arachis hypogaea* (4x)
	*Ah*accB1-1	ACO53609.1	*Arachis hypogaea* (4x)
	*Ah*accB2-1	ACO53611.1	*Arachis hypogaea* (4x)
	*Ah*accC2	XP_025636875.1	*Arachis hypogaea* (4x)
	*Ah*accC1	XP_025700754.1	*Arachis hypogaea* (4x)
	*Ah*accC4	ACO53619.1	*Arachis hypogaea* (4x)
	*Ah*accC3	ACO53618.1	*Arachis hypogaea* (4x)
	*Ah*accA-1	XP_025695444.1	*Arachis hypogaea* (4x)
	*Ah*accA-2	XP_025649321.1	*Arachis hypogaea* (4x)
	*Ah*accD	ACO53622.1	*Arachis hypogaea* (4x)
	*Ha*accC	XP_021986207.1	*Helianthus annuus* (2x)
	*Ha*accA-1	QGZ98789.1	*Helianthus annuus* (2x)
	*Ha*accA-2	XP_022010319.1	*Helianthus annuus* (2x)
	*Co*accC	ACS14663.1	*Camellia oleifera* (4x)
	*At*accB1	*AAC49114.1*	*Arabidopsis thaliana (2x)*
	*At*accB2	*NP_568316.1*	*Arabidopsis thaliana (2x)*
	*At*accC	NP_198386.1	*Arabidopsis thaliana* (2x)
	*At*accA	BAD93727.1	*Arabidopsis thaliana* (2x)
	*At*accD	NP_051068.1	*Arabidopsis thaliana* (2x)
	*Nt*accD	*CAA77362.1*	*Nicotiana tabacum (2x)*
**MCAT**	*Si*FabD	XP_011080770.1	*Sesamum indicum* (2x)
	*Ah*FabD1-1	ACJ07137.1	*Arachis hypogaea* (4x)
	*Ah*FabD1-2	ACJ07138.1	*Arachis hypogaea* (4x)
	*Gm*FabD	NP_001238312.2	*Glycine max* (2x)

	*At*FabD	CAA0373481.1	*Arabidopsis thaliana* (2x)
**KASIII**	*Si*FabH-1	XP_011069912	*Sesamum indicum* (2x)
	*Si*FabH-2	XP_011079297	*Sesamum indicum* (2x)
	*Ah*FabH	XP_025611200.1	*Arachis hypogaea* (4x)
	*Gm*FabH	NP_001237735.1	*Glycine max* (2x)
	*Bn*FabH	XP_013732147.1	*Brassica napus* (4x)
	*At*FabH-1	OAP12004.1	*Arabidopsis thaliana* (2x)
	*At*FabH-2	CAA72385.1	*Arabidopsis thaliana* (2x)
**KASI**	*Si*FabB-1	XP_011085258	*Sesamum indicum* (2x)
	*Si*FabB-2	XP_011096538	*Sesamum indicum* (2x)
	*Si*FabB-3	XP_011082304	*Sesamum indicum* (2x)
	*Gm*FabB-1	XP_006584769.1	*Glycine max* (2x)
	*Gm*FabB-2	NP_001238610.1	*Glycine max* (2x)
	*Bn*FabB	XP_013662893.1	*Brassica napus* (2x)
	*Ha*FabB-1	XP_022025436.1	*Helianthus annuus* (2x)
	*Ha*FabB-2	ABM53471.1	*Helianthus annuus* (2x)
	*Ah*FabB	XP_025639780.1	*Arachis hypogaea* (4x)
	*Nt*FabB	KX033513	*Nicotiana tabacum (2x)*
	*At*FabB-1	NP_199441.1	*Arabidopsis thaliana* (2x)
**KASII**	*Si*FabF-1	XP_011093876	*Sesamum indicum* (2x)
	*Si*FabF-2	XP_011078136	*Sesamum indicum* (2x)
	*Si*FabF-3	XP_011072620	*Sesamum indicum* (2x)
	*Ha*FabF-1	XP_022013627.1	*Helianthus annuus* (2x)
	*Ha*FabF-2	ABI18155.1	*Helianthus annuus* (2x)
	*Ah*FabF-1	XP_025644592.1	*Arachis hypogaea* (4x)
	*Ah*FabF-2	XP_025645142.1	*Arachis hypogaea* (4x)
	*Ji*FabF	DQ987700	*Jatropha curcas* (2x)
	*Gm*FabF	AAF61737.1	*Glycine max* (2x)
**KAS Mitochandrion**	*Si*KASmt	XP_011077001	*Sesamum indicum* (2x)
	*Ah*KASmt	XP_025632626.1	*Arachis hypogaea* (4x)
**SAD**	*Si*SAD1	NP_001291335.1	*Sesamum indicum* (2x)
	*Si*SAD2	XP_011091536.1	*Sesamum indicum* (2x)
	*Si*SAD3	XP_011084689.1	*Sesamum indicum* (2x)
	*Si*SAD4	XP_011077116.1	*Sesamum indicum* (2x)
	*Si*SAD5	XP_011074783.1	*Sesamum indicum* (2x)
	*Si*SAD6	XP_011100275.1	*Sesamum indicum* (2x)
	*Si*SAD7	XP_011100276.1	*Sesamum indicum* (2x)
	*Br*SAD1	XP_009133630.1	*Brassica rapa* (2x)
	*Br*SAD2	XP_009142970.1	*Brassica rapa* (2x)
	*Br*SAD3A	XP_018513314.1	*Brassica rapa* (2x)
	*Br*SAD4	XP_009118393.2	*Brassica rapa* (2x)
	*Br*SAD5	XP_009121502.1	*Brassica rapa* (2x)
	*Br*SAD6	XP_009147347.1	*Brassica rapa* (2x)
	*Br*SAD7	XP_009121503.1	*Brassica rapa* (2x)
	*Rc*SAD1	NP_001310659.1	*Ricinus communis* (2x)
	*Ah*SAD1	ACI45450.1	*Arachis hypogaea* (4x)
	*Ah*SAD2	AID51207.1	*Arachis hypogaea* (4x)
	*Ah*SAD3	AID51208.1	*Arachis hypogaea* (4x)

	*At*FAB2	AT2G43710.1	*Arabidopsis thaliana* (2x)
	*At*AAD2	AT3G02610.1	*Arabidopsis thaliana* (2x)
	*At*AAD3	AT5G16230.1	*Arabidopsis thaliana* (2x)
	*At*SAD4	AT3G02620.1	*Arabidopsis thaliana* (2x)
	*At*SAD5	AT3G02630.1	*Arabidopsis thaliana* (2x)
	*At*SAD6	AT5G16240.1	*Arabidopsis thaliana* (2x)
	*At*SAD7	AT1G43800.1	*Arabidopsis thaliana* (2x)
	Muc-PAD	AAC05293.1	*Macfadyena unguis-cati* (2x)
**FatB**	*Si*FatB1-1	XP_011073093.1	*Sesamum indicum* (2x)
	*Si*FatB1-2	XP_011078334.1	*Sesamum indicum* (2x)
	*Ha*FatB	CAC80370.1	*Helianthus annuus* (2x)
	*Ah*FatB	ABO38555.1	*Arachis hypogaea* (4x)
	*Ha*FatB-1	XP_022024086.1	*Helianthus annuus* (2x)
	*Ha*FatB-2	XP_021989855.1	*Helianthus annuus* (2x)
**FatA**	*Si*FatA	XP_011089673.1	*Sesamum indicum* (2x)
	*Cs*FatA-1	AFQ60948.1	*Camelina sativa* (6x)
	*Cs*FatA-2	AFQ60947.1	*Camelina sativa* (6x)
	*Cs*FatA-3	AFQ60948.1	*Camelina sativa* (6x)
	*Gm*FatA	XP_006602508.1	*Glycine max* (2x)
	*Rc*FatA	NP_001310680.1	*Ricinus communis* (2x)
	*At*FatA-1	NP_189147.1	*Arabidopsis thaliana* (2x)
	*At*FatA-2	NP_193041.1	*Arabidopsis thaliana* (2x)
**FAD**	*Si*FAD2-1	AAX11454.1	*Sesamum indicum* (2x)
	*Si*FAD2-2	XP_011075145.1	*Sesamum indicum* (2x)
	*Si*FAD3	XP_011080789.1	*Sesamum indicum* (2x)
	*Si*FAD6	XP_011079423.1	*Sesamum indicum* (2x)
	*Si*FAD7	NP_001306619.1	*Sesamum indicum* (2x)
	*Si*FAD8	XP_011076709.1	*Sesamum indicum* (2x)
	*Ah*FAD2-1A	AAB84262.1	*Arachis hypogaea* (4x)
	*Ah*FAD2-1B	AAF82293.1	*Arachis hypogaea* (4x)
	*Ah*FAD2-2	ACZ06072.1	*Arachis hypogaea* (4x)
	*Ah*FAD3-4	QFR98376.1	*Arachis hypogaea* (4x)
	*Ah*FAD6	ACZ06070.1	*Arachis hypogaea* (4x)
	*Ah*FAD7-1A	AID51460.1	*Arachis hypogaea* (4x)
	*Ah*FAD8	AFD93985.1	*Arachis hypogaea* (4x)
	*Gm*FAD2-1A	AAX29989.1	*Glycine max* (2x)
	*Gm*FAD2-2	BAD89862.1	*Glycine max* (2x)
	*Gm*FAD3A	AMY60543.1	*Glycine max* (2x)
	*Gm*FAD6	P48628.1	*Glycine max* (2x)
	*Gm*FAD7	ACF19424.1	*Glycine max* (2x)
	*Gm*FAD8-2	NP_001239777.1	*Glycine max* (2x)
	*Cs*FAD2	XP_019096117.1	*Camelina sativa* (6x)
	*Cs*FAD2-2	NP_001291954.1	*Camelina sativa* (6x)
	*Bn*FAD2-4	XP_022570693.1	*Brassica napus* (4x)
	*Bn*FAD3	NP_001302953.1	*Brassica napus* (4x)
	*Bn*FAD6	AAA50157.1	*Brassica napus* (4x)
	*Ha*FAD2	XP_021972383.1	*Helianthus annuus (2x)*
	*At*FAD2	NP_187819.1	*Arabidopsis thaliana* (2x)

	*At*FAD3	AAA61778.1	*Arabidopsis thaliana* (2x)
	*At*FAD6	NP_194824.1	*Arabidopsis thaliana* (2x)
	*At*FAD8	AAA65621.1	*Arabidopsis thaliana* (2x)
	*At*FAD7	NP_187727.1	*Arabidopsis thaliana* (2x)
LACS	*Si*LACS1	XP_011079524.1	*Sesamum indicum* (2x)
	*Si*LACS2	XP_011088325.1	*Sesamum indicum* (2x)
	*Si*LACS4	XP_011101042.1	*Sesamum indicum* (2x)
	*Si*LACS6	XP_011090746.1	*Sesamum indicum* (2x)
	*Si*LACS8	XP_011085413.1	*Sesamum indicum* (2x)
	*Si*LACS9	XP_011069534.1	*Sesamum indicum* (2x)
	*Bn*LACS1	CDY27501.1	*Brassica napus* (4x)
	*Bn*LACS2	CDY29350.1	*Brassica napus* (4x)
	*Bn*LACS3	XP_013664517.1	*Brassica napus* (4x)
	*Bn*LACS4	NP_001302548.1	*Brassica napus* (4x)
	*Bn*LACS5	XP_013669542.1	*Brassica napus* (4x)
	*Bn*LACS9	NP_001303207.1	*Brassica napus* (4x)
	*At*LACS1	BAH19991.1	*Arabidopsis thaliana* (2x)
	*At*LACS2	AT1G49430	*Arabidopsis thaliana* (2x)
	*At*LACS3	AT1G64400	*Arabidopsis thaliana* (2x)
	*At*LACS4	AT4G23850	*Arabidopsis thaliana* (2x)
	*At*LACS5	AAM28872.1	*Arabidopsis thaliana* (2x)
	*At*LACS6	NP_566265.1	*Arabidopsis thaliana* (2x)
	*At*LACS7	NP_198112.2	*Arabidopsis thaliana* (2x)
	*At*LACS8	AT2G04350	*Arabidopsis thaliana* (2x)
	*At*LACS9	AT1G77590	*Arabidopsis thaliana* (2x)
**GPAT**	*Si*GPAT1	XP_011069706.1	*Sesamum indicum* (2x)
	*Si*GPAT2	XP_011082911.1	*Sesamum indicum* (2x)
	*Si*GPAT3	XP_011094070.1	*Sesamum indicum* (2x)
	*Si*GPAT4	XP_011077903.1	*Sesamum indicum* (2x)
	*Si*GPAT5	XP_011088689.1	*Sesamum indicum* (2x)
	*Si*GPAT6	XP_011092934.1	*Sesamum indicum* (2x)
	*Si*GPAT7	XP_011094147.2	*Sesamum indicum* (2x)
	*Si*GPAT8	XP_011085137.1	*Sesamum indicum* (2x)
	*Si*GPAT9	XP_011076807.1	*Sesamum indicum* (2x)
	*Si*GPAT10	XP_011073520.1	*Sesamum indicum* (2x)
	*Si*GPAT11	XP_011086432.1	*Sesamum indicum* (2x)
	*Si*GPAT12	XP_011091226.1	*Sesamum indicum* (2x)
	*Si*GPAT13	XP_011092551.1	*Sesamum indicum* (2x)
	*Si*GPAT14	XP_011093132.1	*Sesamum indicum* (2x)
	*Si*GPAT15	XP_011077573.2	*Sesamum indicum* (2x)
	*Si*GPAT16	XP_011094988.1	*Sesamum indicum* (2x)
	*Si*GPAT17	XP_011098557.1	*Sesamum indicum* (2x)
	*Bn*GPAT1	XP_013696751.1	*Brassica napus* (4x)
	*Bn*GPAT2	CDY15240.1	*Brassica napus* (4x)
	*Bn*GPAT3	CDY22016.1	*Brassica napus* (4x)
	*Ah*GPAT1	AER51964.1	*Arachis hypogaea* (4x)
	*Ah*GPAT2	AER42344.1	*Arachis hypogaea* (4x)
	*Ah*GPAT6	AER42345.1	*Arachis hypogaea* (4x)

	*Ah*GPAT4	AGR66149.1	*Arachis hypogaea* (4x)
	*Ah*GPAT9	AGR66148.1	*Arachis hypogaea* (4x)
	*At*ATS1	AT1G32200	*Arabidopsis thaliana* (2x)
	*At*GPAT1	NP_563768.1	*Arabidopsis thaliana* (2x)
	*At*GPAT2	AT1G02390	*Arabidopsis thaliana* (2x)
	*At*GPAT3	AT4G01950	*Arabidopsis thaliana* (2x)
	*At*GPAT4	AT1G01610	*Arabidopsis thaliana* (2x)
	*At*GPAT5	NP_187750.1	*Arabidopsis thaliana* (2x)
	*At*GPAT6	NP_181346.1	*Arabidopsis thaliana* (2x)
	*At*GPAT7	NP_196227.1	*Arabidopsis thaliana* (2x)
	*At*GPAT8	NP_191950.2	*Arabidopsis thaliana* (2x)
	*At*GPAT9	AT5G60620	*Arabidopsis thaliana* (2x)
**LPAAT**	*Si*LPAAT1	XP_011084281.2	*Sesamum indicum* (2x)
	*Si*LPAAT2-1	XP_011075205.1	*Sesamum indicum* (2x)
	*Si*LPAAT2-2	XP_011078878.1	*Sesamum indicum* (2x)
	*Si*LPAAT3	XP_011080283.1	*Sesamum indicum* (2x)
	*Si*LPAAT4	XP_011074489.1	*Sesamum indicum* (2x)
	*Si*LPAAT5	XP_011088344.1	*Sesamum indicum* (2x)
	*Si*LPAATB	XP_020554192.1	*Sesamum indicum* (2x)
	*Bn*LPAAT1	Q9LLY4.2	*Brassica napus* (4x)
	*Bn*LPAAT2-1	Q9XFW4.1	*Brassica napus* (4x)
	*Bn*LPAAT2-2	NP_001302981.1	*Brassica napus* (4x)
	*Bn*LPAAT2-3	AXX75484.1	*Brassica napus* (4x)
	*Bn*LPAAT2-4	NP_001302955.1	*Brassica napus* (4x)
	*Ah*LPAAT2	XP_025604428.1	*Arachis hypogaea* (4x)
	*Bn*LPAAT3	XP_013749364.1	*Brassica napus* (4x)
	*Bn*LPAAT4-1	AHV90568.1	*Brassica napus* (4x)
	*Bn*LPAAT4-2	AHV90569.1	*Brassica napus* (4x)
	*Rc*LPAATB	AFR42414.1	*Ricinus communis* (2x)
	*Ah*LPAATB	AGT57763.1	*Arachis hypogaea* (4x)
	*At*LPAAT1	NP_194787.2	*Arabidopsis thaliana* (2x)
	*At*LPAAT2	ABG48392.1	*Arabidopsis thaliana* (2x)
	*At*LPAAT3	AT1G51260	*Arabidopsis thaliana* (2x)
	*At*LPAAT4	AT1G75020	*Arabidopsis thaliana* (2x)
	*At*LPAAT5	AT3G18850	*Arabidopsis thaliana* (2x)
**PAH**	*Si*PAH1-1	XP_011077414.2	*Sesamum indicum* (2x)
	*Si*PAH1-2	XP_011094787.1	*Sesamum indicum* (2x)
	*Si*PAH2	XP_020550995.1	*Sesamum indicum* (2x)
	*Bn*PAH1-1	XP_013750374.1	*Brassica napus* (4x)
	*Bn*PAH2	XP_013644973.1	*Brassica napus* (4x)
	*Bn*PAH1-2	CDY41298.1	*Brassica napus* (4x)
	*Ah*PAH1-1	XP_025605008.1	*Arachis hypogaea* (4x)
	*Ah*PAH2-1	QHN88458.1	*Arachis hypogaea* (4x)
	*Ah*PAH2-2	QHO29627.1	*Arachis hypogaea* (4x)
	*Gm*PAH1	XP_003541397.1	*Glycine max* (2x)
	*Gm*PAH2	XP_006578018.1	*Glycine max* (2x)
	*At*PAH1	AT3G09560	*Arabidopsis thaliana* (2x)
	*At*PAH2	AT5G42870	*Arabidopsis thaliana* (2x)

**DGAT**	*Si*DGAT1-1	NP_001291334.1	*Sesamum indicum* (2x)
	*Si*DGAT2D	XP_011098009.1	*Sesamum indicum* (2x)
	*Si*DGAT3	XP_011086024.1	*Sesamum indicum* (2x)
	*Ah*DGAT1c	XP_025688510.1	*Arachis hypogaea* (4x)
	*Ah*DGAT1-3	ATG83402.1	*Arachis hypogaea* (4x)
	*Gm*DGAT1A	NP_001237289.1	*Glycine max* (2x)
	*Oe*DGAT1	AAS01606.1	*Olea europaea* (2x)
	*Cs*DGAT1B	AQU71886.1	*Camelina sativa* (6x)
	*Ah*DGAT2	AEO11788.1	*Arachis hypogaea* (4x)
	*Ah*DGAT2D	XP_025682671.1	*Arachis hypogaea* (4x)
	*Ah*DGAT2-1	AEO11791.1	*Arachis hypogaea* (4x)
	*Gm*DGAT2D	BCB92186.1	*Glycine max* (2x)
	*Oe*DGAT2	ADG22608.1	*Olea europaea* (2x)
	*Eg*DGAT2	XP_010932136.1	*Elaeis guineensis* (2x)
	*Bn*DGAT2D	AKR16145.1	*Brassica napus* (4x)
	*Ah*DGAT3-3	AAX62735.1	*Arachis hypogaea* (4x)
	*Ah*DGAT3-1	ABW34442.1	*Arachis hypogaea* (4x)
	*Ah*DGAT3-3	AAX62735.1	*Arachis hypogaea* (4x)
	*Gm*DGAT3	XP_003542403.1	*Glycine max* (2x)
	*Cs*DGAT3-1	XP_010479249.2	*Camelina sativa* (6x)
	*Cs*DGAT3-2	XP_010461643.1	*Camelina sativa* (6x)
	*Cs*DGAT3-3	XP_010500353.1	*Camelina sativa* (6x)
	*Bn*DGAT1	AAD45536.1	*Brassica napus* (4x)
	*At*DGAT1	AAF19262.1	*Arabidopsis thaliana* (2x)
	*At*DGAT2	NP_566952.1	*Arabidopsis thaliana* (2x)
	*At*DGAT3	OAP16619.1	*Arabidopsis thaliana* (2x)
	*Tm*DGAT1	AAM03340.2	Tropaeolum majus (2x)
	*Jr*DGAT3	XP_018840223.2	*Juglans regia* (2x)
	*Pf*DGAT1	AAG23696.1	*Perilla frutescens* (4x)
	*Sa*DGAT1	GER56917.1	*Striga asiatica* (2x)
	*Sh*DGAT2	AXN76729.1	*Salvia hispanica* (2x)
	*Hi*DGAT2	PIN08318.1	*Handroanthus impetiginosus* (2x)
