# Supplementary material for: Longer Duration of Active Oil Biosynthesis during Seed Development Is Crucial for High Oil Yield—Lessons from Genome-Wide In Silico Mining and RNA-Seq Validation in Sesame
Source: Plants (Basel). 2022 Nov 4;11(21):2980. doi: 10.3390/plants11212980 (PMC9657858; doi:10.3390/plants11212980)
Supplement: Supplementary file 1 [file plants-11-02980-s001.zip › Table_S2.pdf]

Table S2. Computed physico-chemical properties for gene products associated with oil biosynthetic pathway in sesame (*Sesamum indicum*).

Protein family	Protein name	Linkage group	Length (aa)	Mol. Wt. (kDa)	Theoretical pI	Grand average of hydropathicity (GRAVY)	Instability index (II)	Stable yes/no	Aliphatic index	Subcellular location	Transit peptide length	Subunit
ACCase	*Si*accB-1	LG4	280	29.49	8.86	-0.14	60.71	No	84.75	plastid, soluble	65	Heteromeric
	*Si*accB-2	LG3	271	29.06	6.89	-0.19	65.69	No	82.44	plastid, soluble	63	
	*Si*accC	LG2	535	58.53	6.88	-0.22	35.65	Yes	83.16	plastid, soluble	48	
	*Si*accA-1	LG5	758	84.42	8.28	-0.54	37.88	Yes	82.90	plastid, membrane	57	
	*Si*accA-2	LG1	735	82.60	9.25	-0.55	33.98	Yes	87.25	plastid, membrane	-	
	*Si*accA-3	LG11	757	83.81	8.93	-0.46	39.54	Yes	87.65	plastid, membrane	55	
	*Si*accD	cp	509	57.64	5.04	-0.49	51.03	No	72.20	cytoplasm, soluble	-	
MCAT	*Si*FabD	LG6	408	43.36	8.88	0.11	36.94	Yes	99.02	plastid, soluble	83	Monomer
KASIII	*Si*FabH-1	LG2	401	42.42	6.54	0.01	38.62	Yes	96.86	plastid, membrane	72	Homodimer
	*Si*FabH-2	LG5	400	42.28	6.54	-0.02	37.40	Yes	90.53	plastid, membrane	62	
KASI	*Si*FabB-1	LG8	471	50.28	8.03	-0.11	36.44	Yes	84.56	plastid, soluble	43	Homodimer
	*Si*FabB-2	LG12	472	50.14	7.57	-0.11	34.45	Yes	83.77	plastid, soluble	45	
	*Si*FabB-3	LG6	493	52.19	8.37	-0.05	27.79	Yes	84.12	plastid, soluble	38	
KASII	*Si*FabF-1	LG11	547	59.00	8.26	-0.08	47.28	No	76.97	plastid, soluble	49	Homodimer
	*Si*FabF-2	LG5	546	58.74	7.48	-0.02	47.13	No	79.05	plastid, soluble	51	
	*Si*FabF -3	LG3	567	60.54	7.96	0.02	42.75	No	84.53	plastid, soluble	54	
mtKAS	*Si*mtKAS	LG4	470	49.96	7.20	-0.003	36.61	Yes	83.55	mitochondrion, soluble	22	Homodimer
SAD	*Si*SAD-1	LG3	397	45.0	6.43	-0.48	38.50	Yes	77.85	plastid, soluble	33	Homodimer

	*Si*SAD-2	LG10	399	45.5	6.04	-0.47	35.82	Yes	77.96	plastid, soluble	33	
	*Si*SAD-3	LG7	391	44.6	6.14	-0.43	41.35	No	81.05	plastid, soluble	24	
	*Si*SAD-4	LG4	391	44.4	6.38	-0.45	34.49	Yes	81.31	plastid, soluble	24	
	*Si*SAD-5	LG3	384	43.0	6.18	-0.40	32.51	Yes	78.22	plastid, soluble	31	
	*Si*SAD-6	LG16	383	43.7	5.98	-0.33	44.51	No	83.22	plastid, soluble	28	
	*Si*SAD-7	LG16	392	44.5	6.19	-0.35	42.77	No	81.33	plastid, soluble	37	
FatB	*Si*FatB 1-1	LG3	386	44.18	6.20	-0.55	37.65	Yes	80.54	plastid, soluble	-	Homodimer
	*Si*FatB 1-2	LG5	424	46.60	6.28	-0.30	34.05	Yes	85.28	plastid, membrane	56	
FatA	*Si*FatA	LG9	376	42.65	8.33	-0.53	50.05	No	79.34	plastid, soluble	52	Homodimer
FAD	*Si*FAD2-1	LG6	384	44.2	8.56	-0.04	37.32	Yes	89.79	ER, membrane	-	Monomer
	*Si*FAD2-2	LG4	384	43.9	8.73	-0.05	41.76	No	90.37	ER, membrane	-	
	*Si*FAD3	LG6	410	46.6	8.48	-0.18	30.00	Yes	81.52	ER, membrane	-	
	*Si*FAD6	LG5	444	51.3	9.01	-0.17	56.33	No	84.76	plastid, membrane	30	
	*Si*FAD8	LG7	448	51.1	8.16	-0.31	37.91	Yes	87.83	plastid, membrane	72	
	*Si*FAD7	LG4	437	49.9	7.44	-0.26	36.50	Yes	84.68	plastid, membrane	75	
LACS	*Si*LACS1	LG6	661	74.48	6.47	-0.26	32.38	Yes	88.61	ER, membrane	-	Monomer
	*Si*LACS2	LG8	659	73.43	6.15	-0.12	36.86	Yes	86.24	ER, membrane	-	
	*Si*LACS4	LG1	658	73.21	6.39	-0.14	32.81	Yes	88.95	ER, membrane	-	
	*Si*LACS6	LG10	697	77.05	8.05	-0.05	33.63	Yes	91.32	peroxisome, membrane	-	
	*Si*LACS8	LG8	732	80.16	8.55	-0.06	27.05	Yes	92.73	ER, membrane	-	
	*Si*LACS9	LG2	695	75.64	6.81	0.01	28.37	Yes	97.37	ER, membrane	24	
GPAT	*Si*GPAT1	LG2	455	50.23	6.09	-0.22	51.22	No	83.21	plastid, soluble	52	Monomer
	*Si*GPAT2	LG6	547	61.21	9.26	0.18	35.62	Yes	97.28	mitochondrion, membrane		

	*Si*GPAT3	LG11	534	61.18	8.86	0.07	42.70	No	94.34	mitochondrion, membrane	26	
	*Si*GPAT4	LG5	521	59.81	9.63	0.06	43.66	No	98.02	mitochondrion, membrane	-	
	*Si*GPAT5	LG8	371	42.27	9.20	-0.10	48.74	No	94.53	ER, membrane	-	
	*Si*GPAT6	LG10	371	42.94	9.05	-0.12	48.73	No	93.75	ER, membrane	-	
	*Si*GPAT7	LG11	447	50.80	8.86	0.14	33.99	Yes	101.59	ER, membrane	-	
	*Si*GPAT8	LG7	501	55.68	9.10	0.20	42.10	No	101.96	ER, membrane	-	
	*Si*GPAT9	LG4	493	54.66	9.50	0.20	37.81	Yes	98.84	ER, membrane	-	
	*Si*GPAT10	LG3	511	56.12	9.69	0.20	36.50	Yes	98.04	ER, membrane*	-	
	*Si*GPAT11	LG8	500	54.78	9.07	0.24	40.82	No	96.14	peroxisome, membrane*	-	
	*Si*GPAT12	LG10	509	56.40	9.08	0.17	42.24	No	99.96	plastid, membrane*	-	
	*Si*GPAT13	LG1	502	55.80	9.17	0.08	37.77	Yes	100.98	ER, membrane	-	
	*Si*GPAT14	LG11	502	55.93	9.38	0.09	38.22	Yes	100.80	ER, membrane	-	
	*Si*GPAT15	LG5	501	56.03	9.36	0.05	38.35	Yes	98.78	ER, membrane	24	
	*Si*GPAT16	LG11	501	55.78	9.45	0.13	31.74	Yes	98.78	ER, membrane	-	
	*Si*GPAT17	LG15	510	56.16	9.42	0.14	36.14	Yes	95.43	peroxisome, membrane	-	
LPAAT	*Si*LPAAT1	LG7	541	59.53	9.27	-0.05	48.68	No	86.69	plastid, membrane	72	
	*Si*LPAAT2-1	LG4	381	42.64	9.63	0.25	46.15	No	110.55	ER, membrane	-	
	*Si*LPAAT2-2	LG5	386	43.61	9.69	0.23	42.72	No	107.10	ER, membrane	-	
	*Si*LPAAT3	LG6	377	42.80	9.04	0.42	45.18	No	121.27	ER, membrane	-	Monomer
	*Si*LPAAT4	LG3	350	40.52	8.86	0.24	43.08	No	103.49	ER, membrane	-	
	*Si*LPAAT5	LG8	369	42.74	8.64	0.15	42.18	No	94.23	ER, membrane	-	
	*Si*LPAAT6	LG12	316	35.62	9.69	-0.14	38.72	Yes	91.30	ER, membrane	-	
PAH	*Si*PAH1-1	LG5	910	100.66	4.66	-0.52	43.35	No	76.87	nucleus, membrane*	-	Monomer

	*Si*PAH1-2	LG11	857	94.97	4.60	-0.57	41.10	No	73.09	nucleus, membrane*	-	
	*Si*PAH2	LG7	1004	110.47	5.58	-0.43	46.46	No	77.65	golgi apparatus, membrane*	-	
DGAT	*Si*DGAT1	LG11	543	61.94	8.06	0.20	42.38	No	99.04	ER, membrane	48	Homodimer
	*Si*DGAT2	LG14	335	37.48	9.42	0.26	45.99	No	104.69	ER, membrane	-	Monomer
	*Si*DGAT3	LG8	392	42.16	8.59	-0.39	65.78	No	73.88	plastid, soluble	91	Homodimer

*Likelihood for Localization is lower than 0.5; ER: Endoplasmic reticulum.
